# Spring‐Like Behavior in [8]Helicene Diimides: How Helical Pitch Governs Optical Anisotropy and Electronic Conjugation

**DOI:** 10.1002/anie.202508779

**Published:** 2025-07-07

**Authors:** Fridolin Saal, Vincenzo Brancaccio, Krzysztof Radacki, Holger Braunschweig, Prince Ravat

**Affiliations:** ^1^ Institut für Organische Chemie Julius‐Maximilians‐Universität Würzburg Am Hubland D‐97074 Würzburg Germany; ^2^ Institut für Anorganische Chemie and Institute for Sustainable Chemistry & Catalysis with Boron Julius‐Maximilians‐Universität Würzburg Am Hubland D‐97074 Würzburg Germany

**Keywords:** Chiroptical spectroscopy, Helical structures, Helicenes, Molecular spring, Optical anisotropy

## Abstract

Helicenes, with their corkscrew‐shaped geometry, have emerged as prototypical molecular springs for engineering chiral functional materials through precise structural modulation. Here we introduce the design and synthesis of [8]helicene diimides ([8]HDIs) and demonstrate that the helical pitch of their backbone can be precisely tuned by bridging the imide nitrogen atoms with alkyl chains of varying lengths (C_3_–C_6_). This approach constrains the molecular geometry to systematically control optical anisotropy, chiroptical response, and electronic communication. Remarkably, modulation of the helical pitch leads to high optical dissymmetry factors (up to 6.0 × 10^−2^) and enhanced through‐space conjugation. Furthermore, we investigate how variations in the helical pitch affect crystal packing in both enantiopure and racemic samples. Complementary quantum chemical calculations provide insights into the origins of these properties, highlighting the potential of this strategy for designing advanced chiral materials.

## Graphical abstract

Bridging the imide nitrogen atoms of [8]helicene diimides with different alkyl chains allows for precise control over the helical pitch of their backbones. This has a profound impact on the molecular packing, chiroptical properties, and through‐space electronic conjugation of the molecules. As a result, this approach enables fine‐tuning of the properties of this new class of materials with enhanced optical anisotropy and electronic communication.

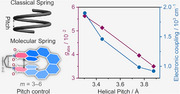

## Introduction

**Figure 1:**
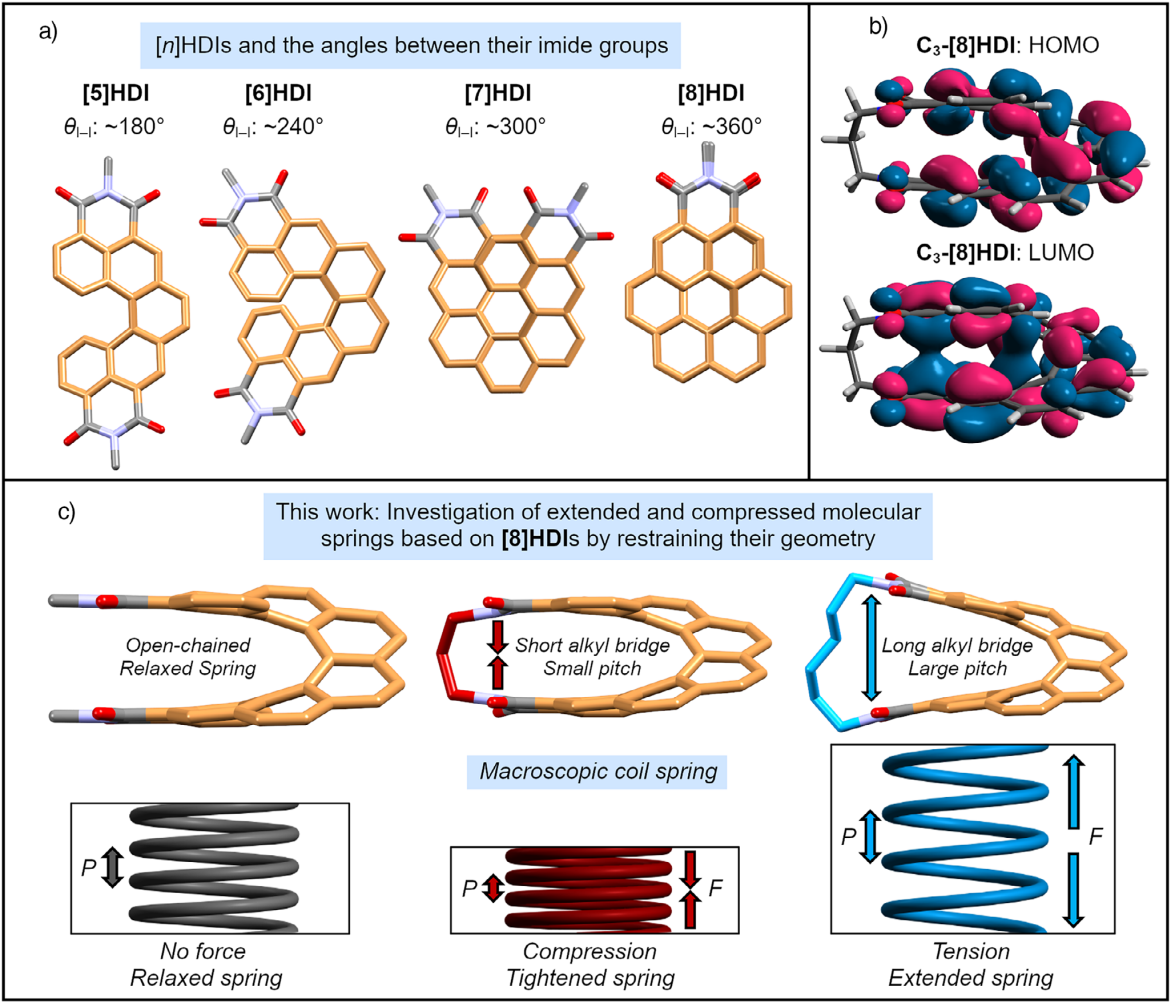
a) Comparison of the members of the **[*n*]HDI** family^[^ ^34^ ^]^ viewed from the top, and the approximate angle between their imide groups (*θ*_I–I_). b) Calculated frontier molecular orbitals of **C_3_‐[8]HDI**. A partial through‐space overlap of the orbital lobes of the LUMO between the imide groups becomes apparent. c) Analogy of the open‐chained and alkyl bridged **[8]HDI**s discussed in this work with the action on a macroscopic coil spring under the influence of a force *F*. The helical pitch *P* shrinks under compression and increases under tension.

Due to their inherently chiral, fully conjugated backbone, helicene‐like compounds^[^ ^1^, ^2^, ^3^, ^4^, ^5^, ^6^, ^7^, ^8^ ^]^ have emerged as promising functional molecules, driven by their pronounced circular dichroism (CD) and circularly polarized luminescence (CPL),^[^ ^8^, ^9^, ^10^, ^11^, ^12^, ^13^, ^14^ ^]^ as well as their potential as emitters for chiral organic light‐emitting diodes (OLEDs),^[^ ^15^, ^16^, ^17^, ^18^ ^]^ in asymmetric catalysis,^[^ ^19^, ^20^, ^21^, ^22^ ^]^ and in emerging topics such as the chirality induced spin selectivity (CISS) effect.^[^ ^23^, ^24^ ^]^ In their simplest form carbo[*n*]helicenes consist of *n* ≥ 5 *ortho*‐fused aromatic rings,^[^ ^25^ ^]^ which forces them to adopt a spiral, or corkscrew shape.^[^ ^26^ ^]^ As such, they are one of the most straightforward molecular analogs for a classical coil spring (Figure 1c), making it possible to employ them as a model for the study of spring‐like behavior on the molecular level.^[^ ^27^, ^28^ ^]^ Similar systems, often referred to as molecular springs,^[^ ^29^ ^]^ have recently attracted interest, particularly in the emerging field of molecular machines,^[^ ^30^, ^31^, ^32^ ^]^ where helical structures have been used to generate a structural response to external stimuli.^[^ ^33^ ^]^

While the conception and synthesis of spring‐like compounds is relatively straightforward, probing their elastic function and the influence of spring tension on the macroscopic properties remains challenging, though various methods to induce structural changes or strain into spring‐like molecules have been developed for specialized systems. For example, in 2021, Petrukhina and co‐workers were able to compress the helical pitch of a double [7]helicene from 2.09  to 1.42 Å by chemical reduction with potassium or rubidium,^[^ ^35^ ^]^ and Choudhury and co‐workers achieved similar results by using acid/base stimuli as well as a ring‐closing photoswitch attached to the helical backbone.^[^ ^36^ ^]^ Apart from chemical stimuli, mechanical compression can also be used to contract molecular springs. Müllen and co‐workers recently investigated the fluorescence of two π‐extended [*n*]helicenes when exposed to pressures of up to 6 GPa in a diamond anvil cell.^[^ ^37^ ^]^ It was found that higher pressures lead to fluorescence quenching and a significant redshift of the residual signal, likely due to compression of the helical spring. However, since these high‐pressure experiments were conducted only with racemic samples, the impact on the chiroptical properties could not be examined.

While responsiveness to external stimuli is an essential property in the quest for molecular machines, such structural changes are reversible and influence the electronic properties and redox behavior of the compounds. This makes it challenging to quantify the influence of stimuli‐induced compression or extension of the spring on the molecular properties. One way to overcome this hurdle is by controlling the helical pitch permanently by structural modification, either through the introduction of sterically demanding substituents, e.g., at the inner rim of π‐extended helicenes^[^ ^38^ ^]^ or by the construction of bridges or tethers within the molecule to force the π‐conjugated system into a strained shape. The latter strategy has been employed successfully to distort the geometry of molecules such as pyrenes,^[^ ^39^ ^]^ teropyrenes,^[^ ^40^ ^]^ perylene diimides,^[^ ^41^ ^]^ and other π‐conjugated compounds.^[^ ^42^, ^43^, ^44^, ^45^, ^46^ ^]^ In 2002, Tanaka and co‐workers employed a bridging strategy to control the helical pitch of [7]thiaheterohelicenes and found that a larger helical pitch led to more intense CD signals.^[^ ^47^ ^]^ In a series of publications, Cuerva and co‐workers explored how *o*‐phenylene ethynylene oligomers can be fixed in helically chiral conformers by stapling their ends together with ether bridges.^[^ ^48^, ^49^, ^50^, ^51^ ^]^ Employing this strategy, they achieved compounds with remarkable luminescence dissymmetry factors (*g*_lum_) of up to 5.5 × 10^−2^.^[^ ^49^ ^]^ They were also able to demonstrate reversible modulation of the CPL emission in response to complexation by Ag(I) cations.^[^ ^48^, ^50^ ^]^ More recently, Gidron and co‐workers^[^ ^52^ ^]^ demonstrated close control over the pitch angle of an S‐shaped double [4]helicene by connecting the two ends of the molecule with alkyl chains of different lengths. In that case, a larger helical pitch angle led to lower fluorescence quantum yields (FQYs) and shorter fluorescence lifetimes, while the molar ellipticity of the lowest‐energy transitions increased. Moya and coworkers studied tuning of helical pitch of chiral BODIPY dimers by sterically demanding substituents, which likewise led to a strong increase in *g*_lum_ with larger pitch.^[^ ^53^ ^]^ These findings highlight that even small structural changes can have a profound impact on the chiroptical properties of the compounds.

Recent work by Crassous, Zysman‐Colman and coworkers highlights a similar observation: Unforeseen distortion in the structure of their multi‐resonance triple helicene led to an observed *g*_lum_ of 6.8 × 10^−4^ as opposed to a calculated value of 2.1 × 10^−2^ for the ideal *C*_3_‐symmetric structure.^[^ ^54^ ^]^ Accordingly, theoretical studies have indicated that the chiroptical properties^[^ ^55^ ^]^ and electronic transport^[^ ^56^ ^]^ of helicenes can be tuned by controlling their helical pitch. Despite the insights achieved in the cited works, a comprehensive study of the influence of precise control over the helical pitch of helicene‐based molecular springs on their crystal packing, optical, chiroptical and electrochemical properties has remained elusive.

**Scheme 1:**
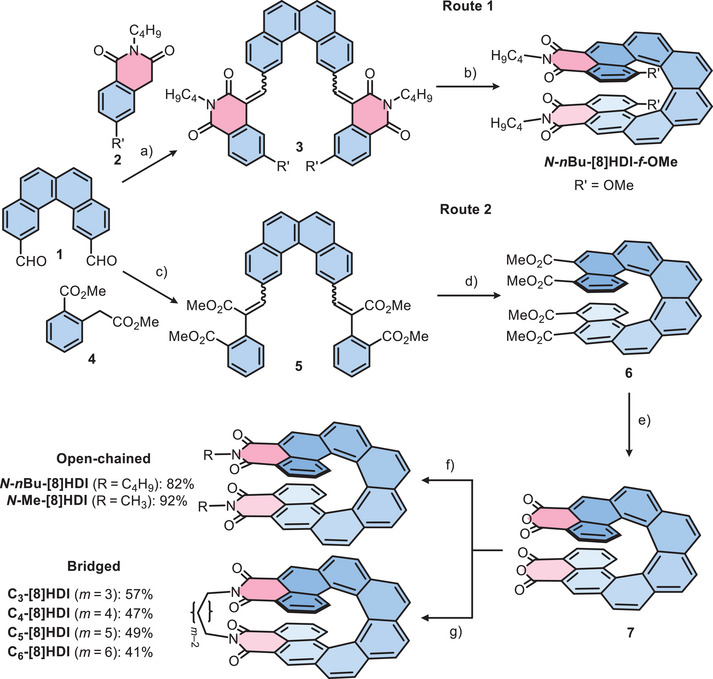
Route 1: Synthesis of ***N*‐*n*Bu‐[8]HDI‐*f*‐OMe** via Knoevenagel condensation with the homophthalimide building block **2** and subsequent photocyclodehydrogenation. Route 2: Substitution of **2** with the diester **4** led to more efficient synthesis of **[8]HDI** derivatives via the intermediate [8]helicene tetracarboxylic ester **6** and [8]helicene tetracarboxylic dianhydride **7**. Reaction conditions and yields: a) Piperidine, CHCl_3_, reflux, 44%. b) I_2_, THF, *hν*, toluene, rt, 8%. c) (i) NaOMe, MeOH, reflux; (ii) SOCl_2_, MeOH, reflux, 82%. d) I_2_, THF, *hν*, toluene, rt, 50%. e) TsOH·H_2_O, toluene, reflux, 94%. f) C_4_H_9_‐NH_2_ or MeNH_3_Cl, propionic acid, reflux. g) H_2_N(CH_2_)_m_NH_2_ (*m* = 3–6), toluene, reflux.

Recently, we have introduced a new class of doubly imide‐substituted^[^ ^7^, ^57^, ^58^, ^59^, ^60^, ^61^, ^62^, ^63^, ^64^ ^]^ carbo[*n*]helicenes ([*n*]helicene diimides, **[*n*]HDI**s, *n*  =  5–7, Figure 1a), and demonstrated that their chiroptical and electrochemical properties can be controlled by variation of the backbone length^[^ ^34^ ^]^ and modification of the chromophore by introduction of functional groups on the outer helix to achieve a push‐pull electronic system.^[^ ^65^ ^]^ This work focuses on the higher homologue of this series, **[8]HDI**, in which the aromatic system bearing the two imide groups completes a full revolution, leading to the interesting case of the two functional groups being stacked directly on top of each other (Figure 1a). This proximity led us to anticipate unusual properties caused by the strong through‐space interaction^[^ ^66^, ^67^ ^]^ between the opposite ends of the molecule (Figure 1b). The adequate proximity of the two imide nitrogen atoms also makes it possible to connect them with alkyl chains of different length, leading to four bridged **[8]HDI**s, **C_m_‐[8]HDI** (*m* = 3–6, Scheme 1), to demonstrate the impact of compressing and expanding the molecular backbone (Figure 1c). For reference and to ease comparison with our previously synthesized compounds, three non‐bridged **[8]HDI**s (***N*‐*n*Bu‐[8]HDI‐*f*‐OMe**, ***N*‐*n*Bu‐[8]HDI**, and ***N*‐Me‐[8]HDI**) were prepared as well. Through experimental results and theoretical calculations, we establish that the helical pitch (*P*) governs the crystal packing, optical and chiroptical properties, as well as the redox chemistry of these compounds. Notably, the chiroptical properties, in particular the absorption and emission dissymmetry factors (*g*_abs_ and *g*_lum_), show a large enhancement upon decrease in helical pitch.

## Results and Discussion

### Synthesis and Characterization

We previously established the two‐step synthesis of *fjord*‐methoxy substituted **[*n*]HDI**s (*n* = 5–7) employing a building‐block based strategy: 1) The coupling of homophthalimide derivative **2** with aromatic dialdehydes in a Knoevenagel reaction to form stilbene‐like precursors, followed by 2) Mallory photocyclodehydrogenation (PCD) to yield **[*n*]HDI**s (*n* = 5–7).^[^ ^34^, ^65^ ^]^ This approach was used as the starting point for the synthesis of the **[8]HDI**s (Route 1, Scheme 1). The first challenge was obtaining the [4]helicene dialdehyde **1**. While this compound is known, the only reference mentioning it did not provide synthetic details.^[^ ^68^ ^]^ We therefore developed a synthetic strategy starting from 2,7‐dibromonaphthalene to yield **1** over four steps in 56% overall yield (Section S2). However, we encountered problems in the PCD step upon attempting to prepare ***N*‐*n*Bu‐[8]HDI‐*f*‐OMe**. While the precursor **3** was obtained in 44% yield, in the PCD reaction even after several days only 8% yield could be achieved.

We therefore developed an alternative synthetic pathway (Route 2, Scheme 1) with the main difference being the use of homophthalic acid dimethyl ester (**4**) in the Knoevenagel reaction in place of **2**, with consequent conversion of the [8]helicene tetracarboxylic ester **6** resulting from the PCD reaction into the [8]helicene dianhydride **7** with an excess of *p*‐toluenesulfonic acid monohydrate, and subsequently reacting **7** with the appropriate amine to obtain the target **[8]HDI**s. Crucially, the PCD reaction from **5** to **6** is possible in significantly larger quantity (on the order of a few hundred milligrams), shorter reaction time (24 h) and with much higher yields (−50%) compared with the earlier procedure, likely due to the electron‐rich nature of **6** compared with **3**.^[^ ^57^, ^69^ ^]^ Even though this pathway requires more steps than route 1, it is more versatile: The enantiomeric separation via HPLC employing a chiral stationary phase (CSP) column can take place with **6** and does not have to be optimized for each new final compound. Additionally, the dianhydride **7** can serve as a starting point for a variety of target molecules, like the extensive chemistry that has been developed for rylene diimides starting from their respective anhydrides.^[^ ^70^, ^71^, ^72^ ^]^

While the open dialkyl‐**[8]HDI**s, (***N*‐*n*Bu‐[8]HDI** and ***N*‐Me‐[8]HDI**), which serve as reference compounds, could be obtained in good yields by straightforward reaction with an excess of amine in propionic acid, synthesizing the alkyl‐bridged **[8]HDI**s (**C_m_‐[8]HDI**, *m* = 3–6) required careful tuning of the reaction conditions. Although the ring closure is kinetically favored, the numerous possible side reactions, including oligomer formation or reaction with two equivalents of diamine, made optimizing the reaction conditions challenging. Through the high dilution principle of Ziegler and Ruggli,^[^ ^73^ ^]^ achieved by slow addition of one equivalent of the respective diamine, and a concentration of the anhydride of − 2 × 10^−4^ M in toluene with imidazole and pyridine as co‐solvents, it was however possible to obtain **C_m_‐[8]HDI** (*m* = 3–6), with yields between 41% and 57%. **6** was resolved into its enantiomers by HPLC on a semi‐preparative CSP column (Figure S19 and Table S6). The reactions with enantiomerically pure **6** gave similar yields for imidization as with ***rac*‐6**. The enantiopurity of the products was ensured by analytical CSP–HPLC. Owing to the high kinetic stability of [8]helicenes,^[^ ^6^, ^74^ ^]^ no racemization took place during the reaction. The structures of the obtained compounds were unambiguously confirmed by 1D and 2D ^1^H and ^13^C NMR measurements (Section S8), high‐resolution mass spectrometry (Section S9), and single‐crystal X‐ray analysis (Section S7).

### Effect of the Helical Pitch on the Solid‐State Structures and Molecular Packing

The effect of the different alkyl bridges on the helical pitch, and consequently the molecular packing, was investigated by X‐ray crystallography. Single crystals were grown by various diffusion and evaporation methods for all racemic **[8]HDI**s, as well as enantiopure **C_3_‐[8]HDI** and **C_4_‐[8]HDI** and ***N*‐Me‐[8]HDI** (Section S7). The expected structures were confirmed, and the strong influence of the length of the alkyl bridge on their helical backbone was revealed.

**Figure 2:**
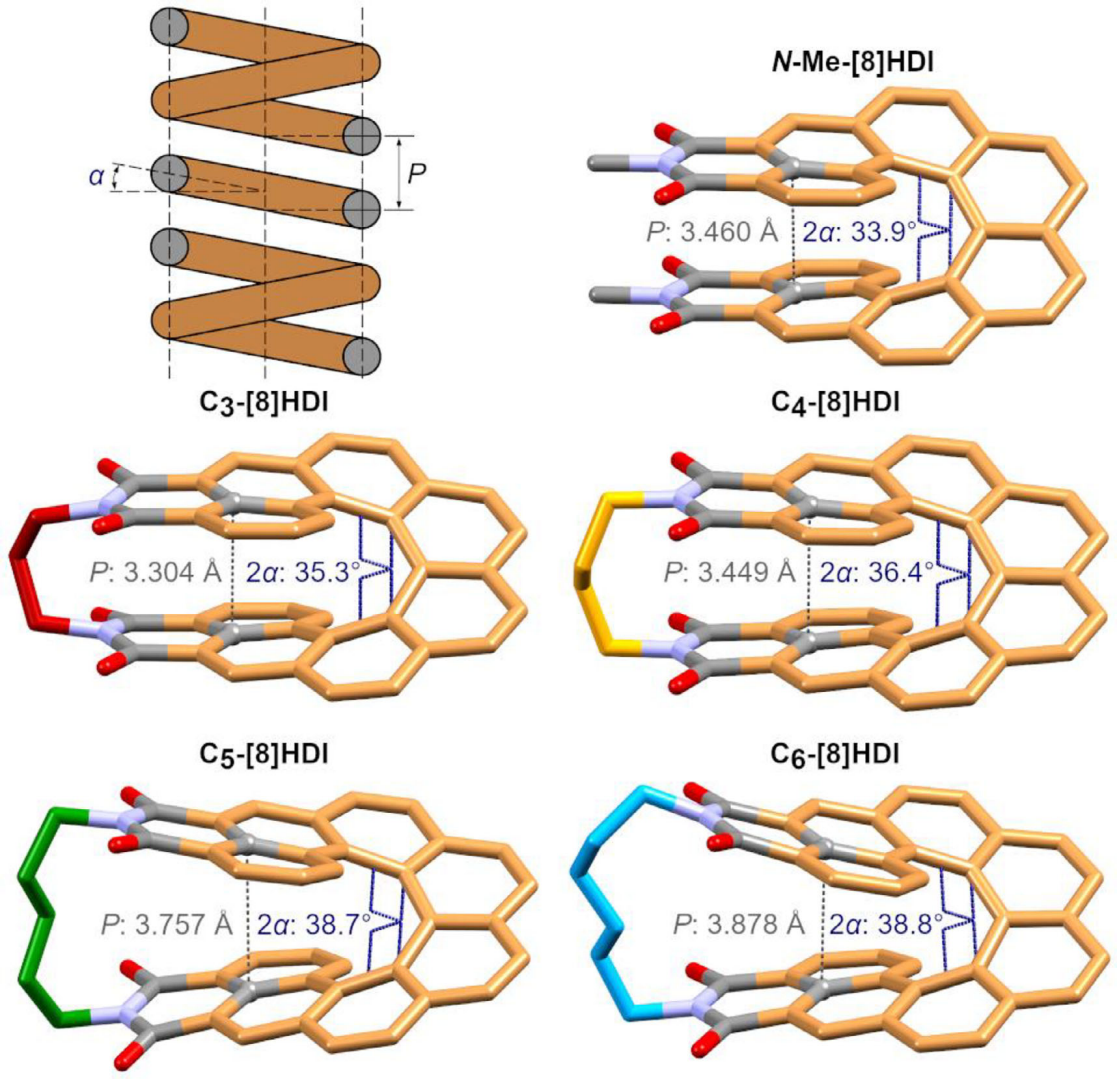
Top left: Characteristics of a classical coil spring with its coil angle *α* and helical pitch *P*. In the discussed molecular springs, 2*α* can be approximated by the torsion angle between the 3^rd^ and 6^th^ carbon‐carbon bonds within the inner helix of the backbone and *P* by the distance between the carbon atoms at the intersection between the [8]helicene and the imide ring (gray dotted line). The values for these characteristics were taken from the crystal structures. A comparison with DFT‐calculated values can be found in Table S17.

The key parameters defining the geometry of a classical spring—its helical pitch *P* and coil angle *α*—have direct equivalents that can be measured in the respective crystal structures (Figure 2). The helical pitch can be approximated by the distance between the carbon atoms at the intersection between the [8]helicene and the imide ring (*d*_C–C_), and 2*α* by the torsion angle between the 3^rd^ and 6^th^ carbon‐carbon bonds within the inner helix of the backbone. *d*_C–C_ increases from 3.304 Å in **C_3_‐[8]HDI** to 3.878 Å in **C_6_‐[8]HDI**, and 2α increases from 35.3° to 38.8°. Notably, the 2*α* value of 33.9° for ***N*‐Me‐[8]HDI** is lower than that of all bridged **[8]HDI**s, which is likely due to strong intermolecular dipole–dipole interactions between the imide groups facilitated by the crystal packing—the DFT‐optimized structure shows the expected intermediate value with 38.2° (Table S17). Hence, as we hypothesised, the helical pitch of **[8]HDI** can be restrained in different compressed or extended states by variation of the bridge length. The experimental values for *d*_C–C_ correlate well with the ones obtained from DFT calculations. A third characteristic, exclusive to the **[8]HDI**s, is the dihedral angle between the planes defined by the imide rings, as a measure of the distortion of the structure by the bridging alkyl chain (Figure S32). While the planes of the imide rings are pushed apart with a significant angle of 26.2° in **C_6_‐[8]HDI**, in **C_3_‐[8]HDI** they are squeezed together, pointing towards each other with an angle of −4.1°. These changes in overall geometry have a considerable effect on the crystal packing patterns (Figure S34).

The crystal packing of ***rac*‐C_3_‐** and ***rac*‐C_4_‐[8]HDI** consists of alternating homochiral columnar stacks, with partially interlocking backbones of (*P*) and (*M*) enantiomers in their respective columns, held together mainly by π–π‐interactions. A comparable arrangement can be found in two polymorphs of ***rac*‐*N*‐Me‐[8]HDI**. In ***rac*‐C_5_‐[8]HDI** crystals, this arrangement changes to stacks of alternating (*P*) and (*M*) enantiomers. ***rac*‐C_6_‐[8]HDI** forms homochiral columns similar to ***rac*‐C_3_‐** and ***rac*‐C_4_‐[8]HDI**, however here the individual molecules in the stacks are offset by 180° with respect to each other. The longer‐chained ***rac*‐*N*‐*n*Bu‐[8]HDI** shows a complicated arrangement due to the larger volume taken up by the alkyl chains. The enantiomerically pure compounds exhibit different crystal packing compared with the racemic compounds. For **(*M*)‐C_3_‐[8]HDI**, the crystal consists of homochiral stacks in which each stacked molecule is turned by 90° in the opposite direction as the helical backbone, while for **(*M*)‐C_4_‐[8]HDI**, the individual molecules within the columnar structures are all oriented parallelly, but each column is turned by 90° with respect to its neighbors. **(*P*)‐*N*‐Me‐[8]HDI** exhibits a herringbone‐like packing consisting of sheets of aligned molecules, but in contrast to a true herringbone pattern there are not two, but four distinct orientations which repeat in the crystal.

### Effect of the Helical Pitch on the Optical Properties

**Figure 3:**
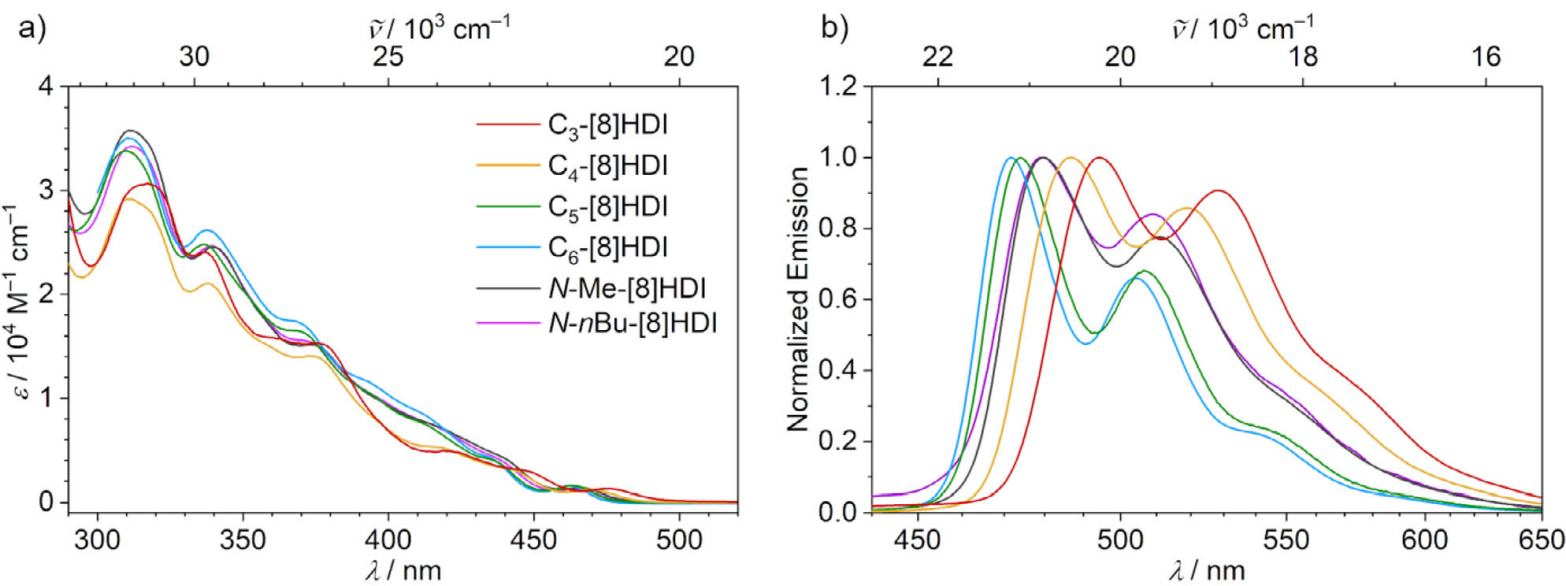
a) Absorption and b) emission (excitation at 350 nm) spectra of the **[8]HDI**s in toluene (c ∼ 10^−5^ m) at 298 K. The absorption and emission spectra follow the same color code for the respective compounds.

The target compounds were all obtained as crystalline, bright yellow solids exhibiting greenish fluorescence in solution. Their absorption spectra are only weakly influenced by the helical pitch—except for a shift of the weak first absorption peak towards longer wavelengths with lower pitch (Figure 3a). The first absorption maximum, around 460 to 475 nm, is of small magnitude, with extinction coefficients of between 1200 m ^−1^ cm^−1^ for **C_3_**‐ and **C_4_‐[8]HDI** to 1600 m ^−1^ cm^−1^ for **C_5_**‐ and **C_6_‐[8]HDI** (in toluene), with the non‐bridged analogs taking up intermediate values (Tables 1 and S1).

**Table 1:** Optical, chiroptical (in toluene) and electrochemical (in THF) properties of the **C_m_‐[8]HDIs** and the non‐bridged reference compounds (for further details and the data for related compounds and different solvents, see SI Section S3).

Compound	*P*/*Å*	*λ*_abs_/nm^a)^	*λ*_em_/nm^b)^	*ϕ*_FL_/%	*τ*_FL_/ns	*E*_g_/eV^c)^	|*g*_abs_| ×10^2^ ^d)^	|*g*_lum_| ×10^2^ ^e)^	LUMO^e)^/eV	HOMO^f)^/eV	*V*_12_ ^g)^ cm^−1^	Δ*E*_red_ ^h)^/eV
**C_3_‐[8]HDI**	3.304	477	495	5.8	6.96	2.56	5.6	6.0	−3.52	−6.06	1902	0.30
**C_4_‐[8]HDI**	3.449	471	487	5.7	6.52	2.60	5.1	5.3	−3.50	−6.08	1458	0.27
**C_5_‐[8]HDI**	3.757	464	474	5.7	6.11	2.65	4.0	4.0	−3.39	−6.03	985	0.27
**C_6_‐[8]HDI**	3.878	463	472	6.5	6.20	2.66	3.5	3.3	−3.36	−6.00	922	0.26
***N*‐Me‐[8]HDI**	3.460	468	480	4.3	5.72	2.63	3.1	3.4	−3.47	−6.09	658	0.23
***N*‐*n*Bu‐[8]HDI**	3.529	466	479	5.6	5.97	2.63	3.1	–	−3.39	−6.03	749	0.23

^a)^ Due to low extinction coefficients for the lowest‐energy transitions, their wavelengths were determined by the first negative peak in the 2^nd^ derivative of the absorption spectrum.

^b)^ Emission maximum upon excitation at 350 nm.

^c)^ Optical energy gaps were calculated using the intersection of the normalized absorption and emission spectra.

^d)^ The maximum dissymmetry factors are given.

^e)^ LUMO = −(5.1 + *E*_red1_).^[^ ^75^ ^]^

^f)^ HOMO = LUMO – *E*_g_.

^g)^ Calculated electronic coupling between the redox centers (Table S10).

^h)^ Difference between the reduction potentials of the two imide moieties. –: Not measured.

In contrast, the helical pitch of the backbone has a large influence on the emission spectra. While all compounds show a similar emission profile (Figure 3b), there is a notable bathochromic shift with decreasing helical pitch. This can be explained by selective stabilization of the LUMO by through‐space interactions between the imide groups, and consequently a smaller optical energy gap (Tables 1 and S5). The non‐bridged reference compounds (***N*‐Me‐[8]HDI** and ***N*‐*n*Bu‐[8]HDI**) show similar absorption and emission profiles with emission maxima at 480 and 479 nm, respectively, indicating a negligible effect of the length of the alkyl chains on the optical spectra. Therefore, the bathochromic shift of **C_3_**‐ and **C_4_‐[8]HDI** and the hypsochromic shift of **C_5_**‐ and **C_6_‐[8]HDI** with respect to these reference compounds can be solely attributed to the compression or extension of the molecular spring imposed by the alkyl bridges. The similar vibronic structures of the emission spectra of all compounds indicate that, despite the large structural changes, the rigidity of the fluorophore is only weakly dependent on the helical pitch.

While there are no pronounced solvent effects in the absorption spectra, a distinct bathochromic shift of the fluorescence can be observed with increasing solvent polarity in all compounds in the series toluene, chloroform, THF, and DMSO (Section S3.2). In toluene, there is a clear vibronic progression in the emission spectra, while in polar DMSO, the features of the spectra are mostly obscured by line broadening.

The photoluminescence quantum yields of all compounds are around 0.05, and the fluorescence lifetimes range between 5 and 7 ns. Therefore, the rate constants for both the radiative and non‐radiative processes are similar in all compounds (Tables 1 and S1–S4). In contrast to earlier studies on the effects of strain on spring‐like helicenes,^[^ ^52^, ^76^, ^77^ ^]^ in the **[8]HDI**s, the changes in the helical pitch of the molecular backbone do not lead to significant fluorescence quenching or acceleration of non‐radiative decay modes.

TD–DFT calculations (B3LYP/6–311G(2d,p)//ωB97XD/6–31g(d,p)) provide further insights into the electronic transitions responsible for the absorption spectra. For the **C_3_‐**, **C_4_‐**, and **C_5_‐**bridged **[8]HDIs**, the lowest‐energy electronic transition consists mostly of the HOMO → LUMO transition with relatively low oscillator strengths of 0.015 to 0.018, in line with the low extinction coefficients of their observed absorption peaks (Table S9). In contrast, the same transition for **C_6_‐[8]HDI,** as well as for the non‐bridged **[8]HDI**s, shows a significant contribution from the HOMO–1 → LUMO transition, with similar oscillator strengths (except for **C_6_‐[8]HDI** which shows *f* = 0.029). In this context it is notable that the HOMO and the HOMO + 1 are almost degenerate in all compounds, while the energetic difference between the LUMO and the LUMO + 1 decreases with the length of the bridging alkyl chain.

### Effect of the Helical Pitch on the Chiroptical Properties

**Figure 4:**
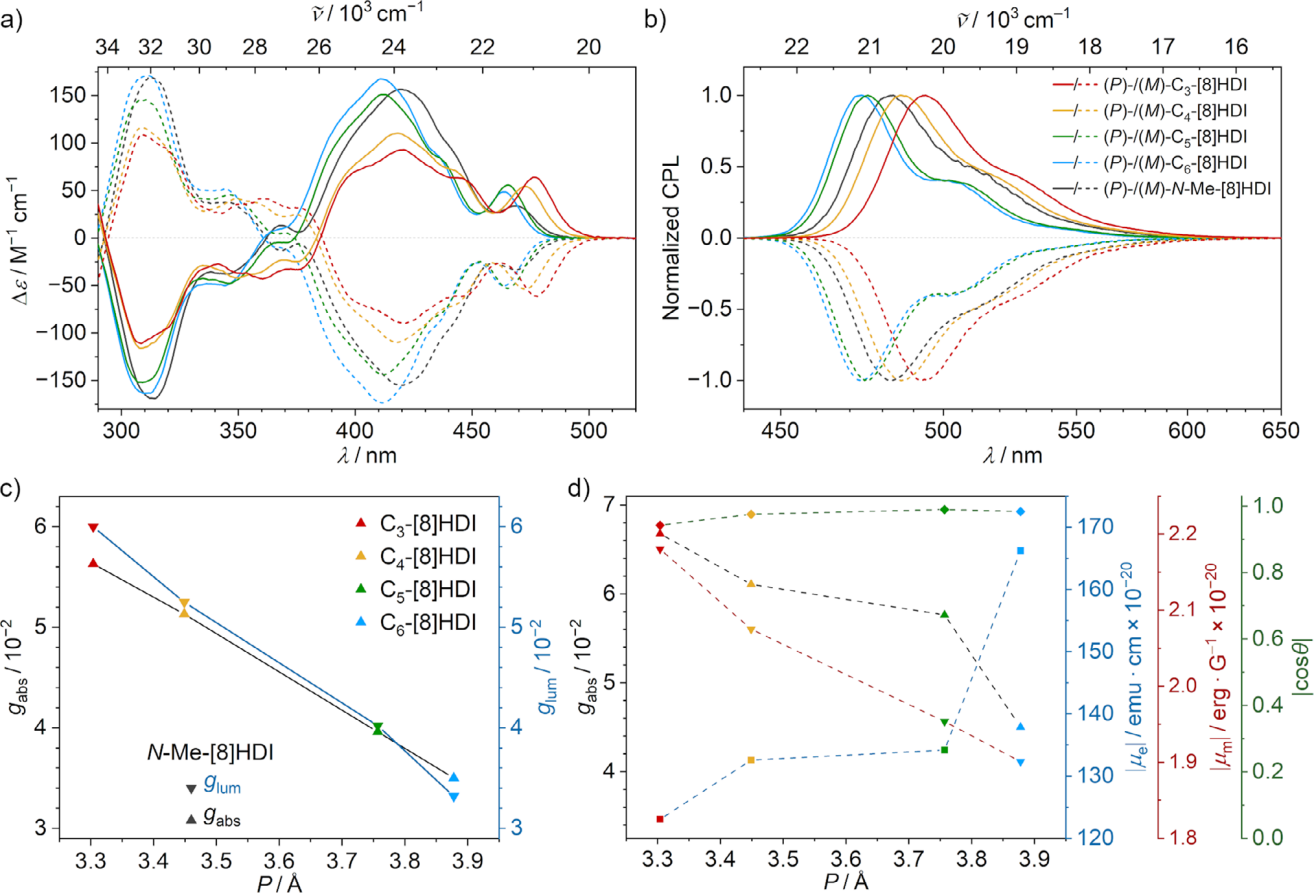
a) Circular dichroism and b) normalized CPL of the **[8]HDI**s in toluene (*c* ∼ 10^−5^ m) at 293 K. c) Influence of the helical pitch (*P*) of the **[8]HDI**s on *g*_abs_ and *g*_lum_. d) Calculated *g*_abs_, the electric transition dipole moment (*µ*_e_), the magnetic transition dipole moment (*µ*_m_), and the cosine of the angle *θ* between *µ*_e_ and *µ*_m_ for the S_0_ → S_1_ transition. The CD and CPL spectra follow the same color code for the respective compounds.

The enantiomerically pure **[8]HDI**s exhibit a pronounced chiroptical response (Figure 4). Their absolute configurations were assigned by comparing the experimental and calculated CD spectra. Additionally, the absolute configurations of **(*M*)‐C_3_‐[8]HDI**, **(*M*)‐C_4_‐[8]HDI**, and **(*P*)‐*N*‐Me‐[8]HDI** could be confirmed by the Flack parameter in their crystal structures. The experimental and the calculated spectra are in good agreement (Figures S21–S22). The CD spectra show three major peaks in the visible and near ultraviolet regions of the spectral range. The second peak (380–450 nm) reaches substantial Δ*ε* values ranging from 90 m ^−1^ cm^−1^ for **C_3_‐[8]HDI** to 170 m ^−1^ cm^−1^ for **C_6_‐[8]HDI**, while the first peak (450–500 nm) coincides with the lowest‐energy absorption peak and exhibits a somewhat smaller Δ*ε*. With decreasing helical pitch, the rotary strength of the first peak increases, while that of the second and third peaks decreases. Concomitantly, the low‐energy transitions in the absorption spectra exhibit much lower extinction coefficients, indicating a relatively high *g*_abs_ factor. Indeed, all **[8]HDI**s show their maximum *g*_abs_ value in the vicinity of the first peak in the CD spectrum. These values range from 3.1 × 10^−2^ for non‐bridged ***N*‐Me‐** and ***N*‐*n*Bu‐[8]HDI**—similar to pristine [8]helicene^[^ ^78^, ^79^ ^]^—up to 5.6 × 10^−2^ in **C_3_‐[8]HDI** and are similar across solvents of varying polarity (Tables S1–S4). The correlation between helical pitch and *g*_abs_ shows a perfectly linear relationship for the bridged compounds, while the open‐chained **[8]HDI**s form outliers on the low side (Figure 4c). Remarkably, the curve shapes and *g*_abs_ values for wavelengths below 450 nm are similar in all tested solvents for all compounds, indicating that the lowest‐energy transition is the only one affected by the geometry changes (Figures S11 and S13–S15).

The CPL spectra of the **[8]HDI**s show similar features as their fluorescence profiles, albeit with a less pronounced vibronic structure, as the lower‐energy bands show a proportionally smaller CPL signal compared with the fluorescence spectra (Figure 4b). Accordingly, the *g*_lum_ plots reach their respective maximums around the emission peak for all compounds. The *g*_lum_ values range from 3.1 × 10^−2^ for **C_6_‐[8]HDI** to 6.0 × 10^−2^ for **C_3_‐[8]HDI**, like the *g*_abs_ values exhibiting a linear relationship with helical pitch. A similar linear relationship between helical pitch and dissymmetry factors has previously been observed in helical BODIPY dimers,^[^ ^53^ ^]^ however in that case better performance was correlated with an increase in helical pitch, while in the **C_m_‐[8]HDI**s, it is instead associated with a decrease in pitch. Again, the open‐chained ***N*‐Me‐[8]HDI** is in an outlier position with a significantly lower value (Figure 4c). The *g*_lum_ plots show a clear peak structure (Figures S11 and S13–S15), which is unusual, as most compounds exhibit a nearly constant *g*_lum_ over the whole width of the emission spectrum.^[^ ^80^ ^]^ Together with the *g*_abs_ of similar magnitude, those are among the largest values recorded for helicene‐type molecules,^[^ ^8^, ^10^, ^11^, ^81^, ^82^, ^83^, ^84^, ^85^, ^86^, ^87^, ^88^, ^89^ ^]^ only exceeded among purely organic compounds by some chiral carbon nanotube wall segments^[^ ^90^ ^]^ and similar molecules featuring cylinder chirality,^[^ ^91^, ^92^ ^]^ as well as some self‐assembled macrocycles.^[^ ^93^ ^]^ The similar values of *g*_abs_ and *g*_lum_ indicate no excimer formation despite the large overlap of the termini of the helicene backbone and the imide groups.^[^ ^94^ ^]^ While helicene‐derived molecules often show significantly lower *g*_lum_ compared to the *g*_abs_ values,^[^ ^10^, ^80^ ^]^ the discussed compounds exhibit similar values, implying minute structural reorganization in the excited state.

To elucidate the influence of the helical pitch on the chiroptical properties, supporting TD–DFT calculations were performed to obtain the values for the electric (*µ*_e_) and magnetic (*µ*_m_) transition dipole moments for the S_0_ → S_1_ and S_1_ → S_0_  transitions (SI Tables S8.1 and S8.2), since the *g* values arise from the interplay of these factors as derived in the following equation:^[^ ^95^ ^]^ $\left|g\right|=4\frac{\mu_{e}\mu_{m}\left|\cos\theta \right|}{\mu_{e}^{2}+\mu_{m}^{2}}$, with *θ* being the angle between the vectors of the two dipole moments. The value for *g* is maximized for *µ*_e_ and *µ*_m_ that are equal in magnitude and either parallel or antiparallel. Therefore, while the low *µ*_e_ of the S_0_ → S_1_ transition (vide supra) impedes its absorptivity, it has the advantageous effect of leading to a relatively high chiroptical response, as the low value for *µ*_e_ makes it closer in value to *µ*_m_, which is usually much lower for small organic molecules. In agreement with the experimental *g* values, going from larger to smaller helical pitch, *µ*_e_ (in 10^−20^ esu cm) decreases from 166 for **C_6_‐[8]HDI** to 123 for **C_3_‐[8]HDI**, while *µ*_m_ (in 10^−20^ erg G^−1^) increases from 1.90 to 2.18. Crucially, this means that the high *g* values observed for the shorter‐bridged compounds are not merely a result of the small *µ*_e_ in their lowest‐energy transitions, but can concomitantly be attributed to an increase in their *µ*_m_. Compared with their bridged analogs, the open‐chained compounds ***N*‐Me‐[8]HDI** and ***N*‐*n*Bu‐[8]HDI** show similar *µ*_e_ to **C_3_‐[8]HDI**, but their *µ*_m_ are notably lower. From the calculated values for *µ*_e_ and *µ*_m_, as well as the angle *θ* between them, *g*_abs_ values were obtained and compared to the observed experimental values (Tables S8.1 and S8.2).^[^ ^96^ ^]^ The calculated values are in good agreement with the experimental ones, albeit slightly overestimating them, in particular for the longer‐chained compounds. Plotting *µ*_e_, *µ*_m_, and cos *θ *for the S_0_ → S_1_ transition against the helical pitch provides an overview of how these values are affected by the helical pitch (Figure 4d). The observed increase in *g* values with decreasing helical pitch therefore results from a concurrent decrease in *µ*_e_ and an increase in *µ*_m_, while cos *θ* remains nearly constant. A direct relationship between the rotational strength R of electronic transitions in helical molecules and the radius *r* and pitch *P* was first shown by Tinoco and Woody in 1964 using the simple free electron on a helix model.^[^ ^97^ ^]^ A recent theoretical study further demonstrated a linear relationship between *µ*_m_ and the area enclosed by the backbone of helicene‐like molecules.^[^ ^98^ ^]^ A similar mechanism might be responsible for the increase in *µ*_m_ and dissymmetric factors in the shorter‐bridged **[8]HDI**s.

### Effect of the Helical Pitch on the Electrochemical Properties

**Figure 5:**
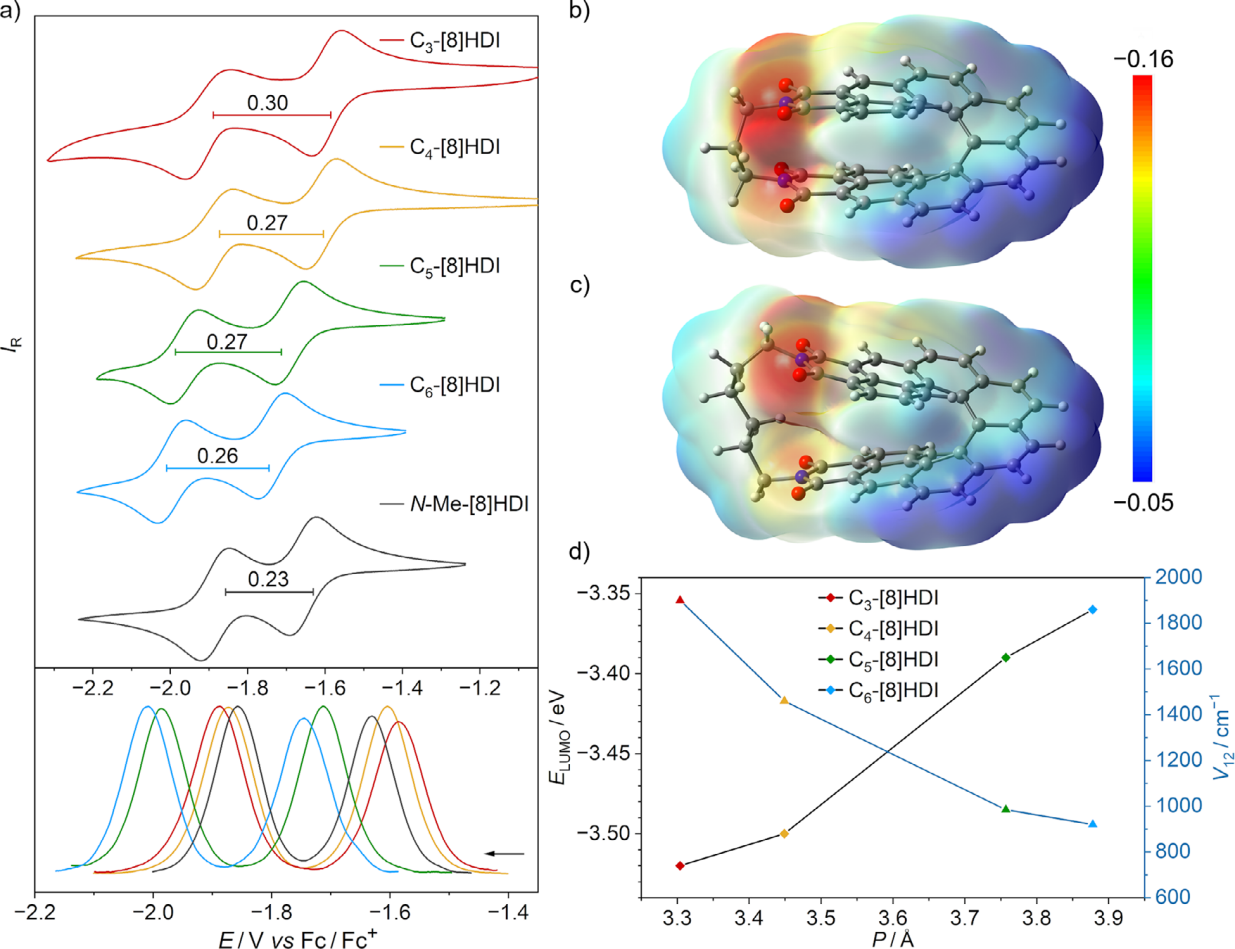
a) Influence of the helical pitch on the redox properties of the **[8]HDI**s. CV (top) and DPV (bottom) plots of **C_m_‐[8]HDI** (*m* = 3–6), and ***N*‐Me‐[8]HDI**, in THF with [Bu_4_N][PF_6_] (0.2 m) as supporting analyte, at a scan rate of 200 mV s^−1^ for CV and 25 mV s^−1^ for DPV. In the CV plots, the values for the difference between the first and second reduction peaks (Δ*E*_red_) for the respective compounds are given in V. b) ,  c) Calculated molecular electrostatic potential surface (MEP) for the radical anions of **C_3_‐[8]HDI** / **C_6_‐[8]HDI**. d) Relationship between the helical pitch, the LUMO level and the calculated electronic coupling (*V*_12_) between the two redox centers.

In our previous work, we demonstrated how the interplay of through‐space and through‐bond interactions between the imide groups on the smaller **[*n*]HDI**s (*n* = 5–7) influences their redox behavior.^[^ ^34^ ^]^ We established that bringing the imide groups—and thus, the redox centers—closer together in space can compensate the decreasing conjugation through the twisted backbone of the molecules with increasing length of the helicenes. The maximum through‐space conjugation was observed in **[7]HDI**, where two terminal benzene rings of the [7]helicene backbone are overlapping. For the **[8]HDI**s, we anticipated an enhanced through‐space conjugation due to the position of the terminal naphthalimide moieties directly on top of each other (Figure 1a). The electrochemical properties of the bridged **[8]HDI**s and the reference compounds ***N*‐Me‐[8]HDI** and ***N*‐*n*Bu‐[8]HDI**, as well as *fjord*‐methoxy substituted ***N*‐*n*Bu‐[8]HDI‐*f*‐OMe** (Figures 5, S16–S18 and Table S5) were probed by cyclic voltammetry (CV) and differential pulse voltammetry (DPV) measurements. As expected, all **[8]HDI**s show two distinct, reversible reduction waves in the CV and two symmetrical peaks in the DPV plots, corresponding to sequential one‐electron reduction of the two imide units. In contrast, no oxidation could be probed within the electrochemical window of THF.

Several trends became apparent: 1) The value of Δ*E*_red_ for ***N*‐*n*Bu‐[8]HDI‐*f*‐OMe** (0.25 V) is indeed the largest among the homologous series of *fjord*‐methoxy substituted **[*n*]HDI**s (*n* = 5–8), confirming that the through‐space conjugation of the imide groups reaches a maximum for this compound. The other two non‐bridged **[8]HDI**s show slightly lower values of Δ*E*_red_ (−0.23 V)—for both ***N*‐Me‐[8]HDI** and ***N‐n*Bu‐[8]HDI**—due to the absent methoxy group. 2) All bridged compounds exhibit a larger value for Δ*E*_red_ compared with the non‐bridged reference compounds. 3) Δ*E*_red_ increases with decreased helical pitch from 0.26 V for **C_6_‐[8]HDI** to 0.30 V for **C_3_‐[8]HDI**, although the value for **C_4_‐[8]HDI** is similar to that for **C_5_‐[8]HDI** despite the increase in helical pitch in the latter. This indicates that other effects, such as electronic communication through the alkyl bridge, may play a role as well.

The redox potential of the first reduction steadily decreases with the helical pitch, corresponding to easier reduction and consequently a lower LUMO level, while the HOMO level stays nearly constant for all investigated compounds (Figure 5 and Table S5). This can be rationalized by the greater stabilization of negative charges by two spatially close imides, which is further supported by the calculated molecular electrostatic potential surfaces (MEPs) of the radical anions (Figures 5b,c and S23), where it becomes apparent that the negative charge is more delocalized between the two imide units in the molecules bearing a shorter bridging chain. Likewise, the calculated LUMO of the neutral molecule shows significantly increased through‐space orbital overlap in **C_3_‐[8]HDI** compared with the higher homologs (Figures 1b and S20).

The strong electronic coupling between the two redox centers is further supported by the (TD)–DFT calculated intervalence charge transfer (IV–CT) parameters (Table S10) of their radical anions. The calculated electronic coupling (*V*_12_) values can serve as a quantitative measure of electronic communication between the two redox centers.^[^ ^99^, ^100^ ^]^ As anticipated the calculated values for *V*_12_ show an inverse relationship with the helical pitch (Figure 5d)—the *V*_12_ value for **C_3_‐[8]HDI** (1902 cm^−1^) is nearly double that for **C_6_‐[8]HDI** (922 cm^−1^), which demonstrates that the electronic coupling between the redox centers is strongly dependent on their through‐space distance, further supporting the CV and DPV data (Table S10).^[^ ^101^, ^102^, ^103^ ^]^ Similar to their smaller homologues (**[*n*]HDI**, *n* = 5–7), the *V*_12_ values for the radical anions of all **[8]HDIs** are smaller than half of their reorganization energy (*V*_12_ < *λ* / 2), which classifies them as Robin−Day class II mixed‐valence compounds.^[^ ^104^ ^]^

## Conclusion

In summary, by incorporating alkyl bridges into [8]helicene diimide, we have effectively locked **[8]HDI**‐based molecular springs into well‐defined compressed and extended conformations. X‐ray structural analysis revealed that increasing or reducing the alkyl chain length induces a systematic increase or decrease in helical pitch, which in turn significantly influences both the redox and chiroptical properties. Specifically, a reduced helical pitch stabilizes the LUMO through enhanced through‐space interactions between the imide groups, resulting in lower reduction potentials and a greater disparity between the reduction potentials of the two imide units. Concomitantly, the optical properties are markedly affected—lower helical pitch causes a bathochromic shift in the lowest‐energy absorption and emission bands, and both the absorption (*g*_abs_) and luminescence (*g*_lum_) dissymmetry factors increase linearly, reaching values up to −6 × 10^−2^ for **C_3_‐[8]HDI**. Our findings underscore that precise conformational control can be employed to tune the properties of helically chiral small molecules. Our work establishes that bridging functional groups with alkyl chains is an effective strategy for achieving such control, and ongoing studies in our group are aimed at further elucidating the structure‐property relationships of **[*n*]HDI**s to optimize their performance as chiral organic functional materials.

## Supporting Information

The authors have cited additional references within the Supporting Information.^[^ ^105^, ^106^, ^107^, ^108^, ^109^, ^110^, ^111^, ^112^, ^113^, ^114^, ^115^, ^116^, ^117^, ^118^ ^]^ Supporting Information contains the following: Experimental and calculation details, materials, synthesis and characterization, additional spectroscopic data, HPLC plots; Tables S1–S17, and Figures S1–S103. Deposition Numbers 2434331 (for ***rac*‐C_3_‐[8]HDI**), 2434333 (for ***rac*‐C_4_‐[8]HDI**), 2434334 (for ***rac*‐C_5_‐[8]HDI**), 2434335 (for ***rac*‐C_6_‐[8]HDI**), 2434337 (for ***rac*‐*N*‐Me‐[8]HDI** Polymorph 1), 2434338 (for ***rac*‐*N*‐Me‐[8]HDI** Polymorph 2), 2434339 (for ***rac*‐*N*‐nBu‐[8]HDI**), 2434330 (for **(*M*)‐C_3_‐[8]HDI**), 2434332 (for **(*M*)‐C_3_‐[8]HDI**), 2434336 (for **(*P*)‐*N*‐Me‐[8]HDI**) contain the supplementary crystallographic data for this paper. These data are provided free of charge by the joint Cambridge Crystallographic Data Centre and Fachinformationszentrum Karlsruhe. Access structures service.

## Conflict of Interests

The authors declare no conflict of interest.

## Supporting information



Supporting Information

Supporting information

Supporting information

## Data Availability

Research data are not shared.
