# Supplementary material for: Spring‐Like Behavior in [8]Helicene Diimides: How Helical Pitch Governs Optical Anisotropy and Electronic Conjugation
Source: Angew Chem Int Ed Engl. 2025 Jul 7;64(34):e202508779. doi: 10.1002/anie.202508779 (PMC12363639; doi:10.1002/anie.202508779)
Supplement: Supplementary file 1 — Supporting Information [file ANIE-64-e202508779-s003.pdf]

Supporting Information for

Spring-Like Behavior in [8]Helicene Diimides: How Helical Pitch Governs Optical Anisotropy and Electronic Conjugation

Fridolin Saal[a], Vincenzo Brancaccio[a], Krzysztof Radacki[b], Holger Braunschweig[b], and Prince Ravat*[a]

[a] F. Saal, V. Brancaccio, Dr. Prince Ravat
Institut für Organische Chemie
Julius-Maximilians-Universität Würzburg
Am Hubland, D-97074 Würzburg, Germany
E-mail: princekumar.ravat@uni-wuerzburg.de

[b] Dr. K. Radacki, Prof. Dr. H. Braunschweig
Institut für Anorganische Chemie and Institute for Sustainable Chemistry & Catalysis with Boron
Julius-Maximilians-Universität Würzburg
Am Hubland, D-97074 Würzburg, Germany

Table of Contents

S1. Experimental Details S2

S2. Synthesis S4

S3. Spectroscopy S14

S3.1. Fluorescence Lifetime Spectroscopy S15

S3.2. Optical and Chiroptical Spectroscopy S19

S4. Electrochemistry S24

S5. Chiral Stationary Phase HPLC S26

S6. Quantum Chemical Calculations S28

S7. Single Crystal Data S36

S8. NMR Spectroscopy S50

S9. High-Resolution Mass Spectrometry (HRMS) S90

S10. Cartesian Coordinates S94

S11. References S95

S1. Experimental Details

Synthesis and Materials

All chemicals and solvents were purchased from commercial sources and were used without further purification unless stated otherwise. Reactions and experiments sensitive to dioxygen were performed using Schlenk techniques and nitrogen-saturated solvents. Compound **2** was prepared as in our previous work.[1]

Chromatography:

Open-column chromatography and thin-layer chromatography (TLC) were performed on silica gel (Merck silica gel 60M, 40–63 µm).

NMR Spectroscopy:

The NMR experiments were performed at 298 K on NMR spectrometers operating at 400 MHz ^{1}H and 101 MHz ^{13}C frequencies. Standard pulse sequences were used, and the data was processed using 2-fold zero-filling in the indirect dimension for all 2D experiments. Chemical shifts (δ) are reported in parts per million (ppm) relative to the solvent residual peak (^{1}H and ^{13}C NMR, respectively): $CDCl_3$ (δ = 7.26 and 77.16 ppm), CD_2Cl_2 (δ = 5.32 and 54.00 ppm), DMSO-d_6 (δ = 2.50 and 39.52 ppm), and J values are given in Hz. Structural assignment was made with additional information from *g*COSY, *g*NOESY, HSQC and HMBC experiments.

HRMS:

MALDI–TOF–HRMS were measured on a Bruker ultrafleXtreme mass spectrometer. *Trans*-2-[3-(4-*tert*-butylphenyl)-2-methyl-2-propenylidene]malononitrile (DCTB) dissolved in chloroform (30 mg mL^{-1}) was used as supporting matrix in the MALDI–TOF–HRMS measurement. Reference spectra were simulated using the mMass software.[2-4]

Melting Points:

Melting points were measured using an OptiMelt Automated Melting Point System from Stanford Research Systems.

UV–Vis Absorption Spectroscopy:

UV–Vis spectra were measured on a JASCO V–670 spectrophotometer.

Emission Spectroscopy:

Room temperature fluorescence emission spectra were measured on an Edinburgh FLS 980 photoluminescence spectrometer. A rectangular 10 mm quartz glass cuvette with a Teflon screw cap was used for the measurements.

Fluorescence Lifetime and Quantum Yield:

The fluorescence lifetimes were measured on an Edinburgh FLS 980 photoluminescence spectrometer using the Time Correlated Single Photon Counting (TCSPC) technique for the acquisition of single photons. The samples were dissolved in the respective solvent and either a 418.6 nm or 378.2 nm pulsed laser diode with a pulse interval of 100 ns (10,000,000 Hz) was used for excitation. Decays were recorded to 10,000 counts in the peak channel with a record length of 8192 channels. The band pass of the monochromator was adjusted to give a signal count rate of <100 kHz. The instrument response function (IRF) was recorded by measuring the excitation signal using a cuvette filled with LUDOX® colloidal silica suspension to scatter

the laser light. The resulting spectra were analyzed with the FAST (Fluorescence Analysis Software Technology) software by Edinburgh Instruments, where the fluorescence lifetimes were calculated by monoexponential iterative reconvolution fits employing non-linear least-squares analysis. The quality of the fits was judged by the calculated value of the reduced $\chi 2$ and visual inspection of the weighted residuals.

The absolute fluorescence quantum yields (FQY) were measured on the same spectrometer with an Integrating Sphere Assembly calibrated integrating sphere (F-M01) installed. A 450 W xenon arc lamp was used as a light source. The raw quantum yields were calculated within the F980 software by Edinburgh Instruments. Due to the low extinction coefficients of the compounds in the overlap region of the absorption and emission spectra, self-absorption correction was not performed.

Electronic Circular Dichroism and Circularly Polarized Luminescence Spectroscopy:

The electronic circular dichroism (ECD) spectra were recorded on either a Jasco J–810 CD spectropolarimeter, or a customized JASCO CPL–300 / J–1500 hybrid spectrometer, at 293 K.

The circularly polarized luminescence (CPL) spectra were recorded with a customized JASCO CPL–300 / J–1500 hybrid spectrometer at 293 K. Excitation / Emission band widths of 26 nm / 13 nm were used for the CPL measurement.

Cyclic Voltammetry and Differential Pulse Voltammetry:

Cyclic voltammetry (CV) and differential pulse voltammetry (DPV) experiments were performed in THF with 0.2 M $[Bu_4N][PF_6]$ as supporting electrolyte, using a Gamry Instruments Reference 600 potentiostat. A standard three-electrode cell configuration was employed, using a platinum disk working electrode, a platinum wire counter electrode, and a platinum wire serving as reference electrode. The redox potentials were referenced to the ferrocene (Fc) / ferrocenium (Fc^+) redox couple used as an internal standard.

S2. Synthesis

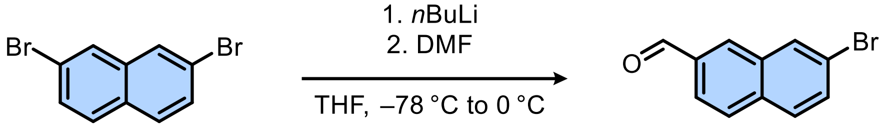

7-Bromo-2-naphthaldehyde. Under an inert atmosphere, 2,7-dibromonaphthalene (15.0 g, 52.5 mmol, 1.00 eq.) was dissolved in dry THF (250 mL). The solution was cooled to −78 °C and a 2.31 M solution of *n*-butyllithium in hexane (22.4 mL, 51.7 mmol, 0.99 eq., exact concentration determined by titration with diphenylacetic acid immediately before use) was steadily added to the reaction mixture over a period of 20 minutes using a syringe pump. The resulting cloudy suspension was stirred for 10 min at −78 °C. Dry *N*,*N*-dimethylformamide (5.21 mL, 5.52 g 75.5 mmol, 1.44 eq.) was added and the suspension was stirred for an additional 1 h at −78 °C, 1 h at −15 °C and 1 h at 0 °C. The reaction mixture was quenched with 30 mL of saturated aqueous ammonium chloride solution and stirred for 1 h at room temperature. The majority of the THF was evaporated, during which the crude product largely precipitated. The crude mixture was transferred into a separating funnel and EtOAc was added until the precipitate dissolved. The aqueous phase was then extracted twice with EtOAc. The combined organic phases were brought to dryness *in vacuo* and the obtained off-white crude product was purified by column chromatography on silica gel (petroleum ether / EtOAc 3:1) to deliver the target compound as an off-white powder (11.0 g, 46.8 mmol, 89%).

^{1}H NMR (400 MHz, $CDCl_3$, rt): δ [ppm] = 10.16 (d, J = 0.6 Hz, 1H), 8.26 (s, 1H), 8.17 (s, 1H), 7.98 (dd, J = 8.6, 1.6 Hz, 1H), 7.92 (d, J = 8.6 Hz, 1H), 7.79 (d, J = 8.8 Hz, 1H), 7.72 (dd, J = 8.8, 2.0 Hz, 1H).

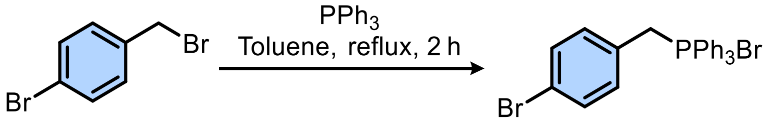

(4-Bromobenzyl)triphenylphosphonium bromide.[5] Triphenylphosphane (37.8 g, 144 mmol, 1.20 eq.) and 4-bromobenzyl bromide (30.0 g, 120 mmol, 1.00 eq.) were dissolved in toluene (650 mL) and heated to reflux for 2 h. The mixture was cooled to room temperature and the voluminous, white precipitate (55.6 g, 109 mmol, 90%) was filtered and dried under high vacuum.

^{1}H NMR (400 MHz, DMSO–d_6, rt): δ [ppm] = 7.92 (m, 3H), 7.76 (m, 6H), 7.69 (m, 6H), 7.46 (dd, J = 8.5, 0.9 Hz, 2H), 6.91 (dd, J = 8.5, 2.5 Hz, 2H), 5.18 (d, J = 15.8 Hz, 2H).

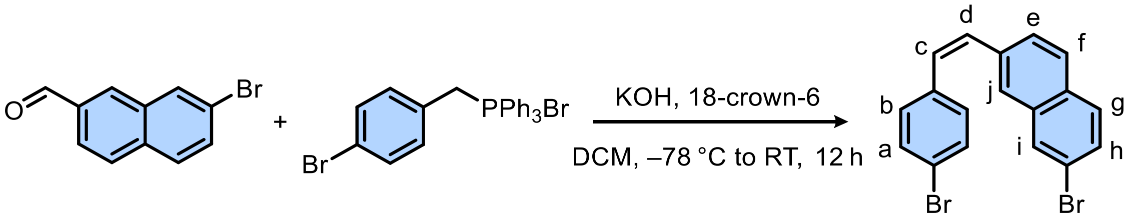

(*Z*)-2-bromo-7-(4-bromostyryl)naphthalene.[6] (4-Bromobenzyl)triphenylphosphonium bromide (17.8 g, 34.7 mmol, 1.20 eq.), 7-bromo-2-naphthaldehyde (6.80 g, 28.9 mmol, 1.00 eq.) and 18-crown-6 (917 mg, 3.47 mmol, 0.120 eq.) were suspended in DCM (250 mL) and the mixture was cooled to −78 °C. Freshly ground KOH (4.0 g, 71 mmol, 2.4 eq.) was added to the reaction mixture and the resulting suspension was stirred for 2 h at −78 °C. The

suspension was then stirred overnight while being allowed to warm to room temperature. The resulting solution was filtered, the filtrate was washed with ammonium chloride solution and extracted with DCM. The organic phase was dried over $MgSO_4$ and the volatiles were removed by evaporation under reduced pressure. The off-white residue was purified by column chromatography on silica gel (petroleum ether) to yield the title compound (7.93 g, 20.4 mmol, 71%) as a white crystalline solid.

Melting point: 118 °C.

^{1}H NMR (400 MHz, $CDCl_3$, rt): δ [ppm] = 7.88 (d, J = 1.9 Hz, 1H, H_i), 7.70 – 7.58 (–, 3H, H_f, H_g, H_j), 7.52 (dd, J = 8.7, 1.9 Hz, 1H, H_h), 7.40 – 7.30 (–, 3H, H_a, H_e), 7.12 (d, J = 8.5 Hz, 2H, H_b), 6.77 (d, J = 12.2 Hz, 1H, H_d), 6.62 (d, J = 12.2 Hz, 1H, H_c).

^{13}C NMR (101 MHz, $CDCl_3$, rt): δ [ppm] = 135.9 (C_q), 135.7 (C_q), 134.7 (C_q), 131.6 (CH), 131.1 (C_q), 130.7 (CH), 130.6 (CH), 130.1 (CH), 130.0 (CH), 129.5 (CH), 129.4 (CH), 127.8 (CH), 127.3 (CH), 127.1 (CH), 121.4 (C_q), 120.3 (C_q).

HRMS (MALDI): m / z: calculated $[C_{18}H_{12}Br_2{-}e^-]^+$ 385.93003; found 385.93542 ($|\Delta|$ = 14.0 ppm).

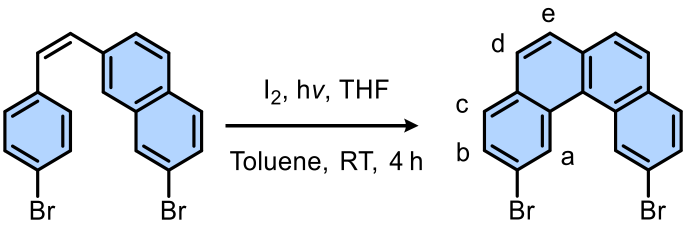

2,11-Dibromobenzo[c]phenanthrene. The reaction was performed in a Peschl Ultraviolet GmbH photoreactor with a Heraeus 150 W medium pressure mercury lamp at room temperature. (*Z*)-2-bromo-7-(4-bromostyryl)naphthalene (500 mg, 1.29 mmol, 1.00 eq.) was dissolved in toluene (600 mL) and the solution was deoxygenated by vigorous bubbling with N_2 for 1 h. I_2 (359 mg, 1.42 mmol, 1.10 eq) and THF (6 mL) were added, and the mixture was irradiated for 4 h. The residual iodine was quenched with saturated $Na_2S_2O_3$ solution (10 mL), and the volatiles were evaporated under reduced pressure. The crude product was purified by column chromatography on silica gel (petroleum ether) to deliver the title compound as a white crystalline solid (478 mg, 1.24 mmol, 96%).

Melting point: 157 °C.

^{1}H NMR (400 MHz, $CDCl_3$, rt): δ [ppm] = 9.15 (d, J = 1.9 Hz, 2H, H_a), 7.87 (d, J = 8.6 Hz, 2H, H_c), 7.85 (d, J = 8.6 Hz, 2H, H_e), 7.80 (d, J = 8.6 Hz, 2H, H_d), 7.72 (dd, J = 8.6, 1.7 Hz, 2H, H_b).

^{13}C NMR (101 MHz, $CDCl_3$, rt): δ [ppm] = 132.0 (C_q), 131.9 (C_q), 131.3 (C_q), 130.3 (CH), 129.8 (CH), 129.5 (CH), 127.8 (CH), 127.3 (CH), 125.3 (C_q), 121.2 (C_q).

HRMS (MALDI): m / z: calculated $[C_{18}H_{10}Br_2{-}e^-]^+$ 383.91438; found 383.9113 ($|\Delta|$ = 8.02 ppm).

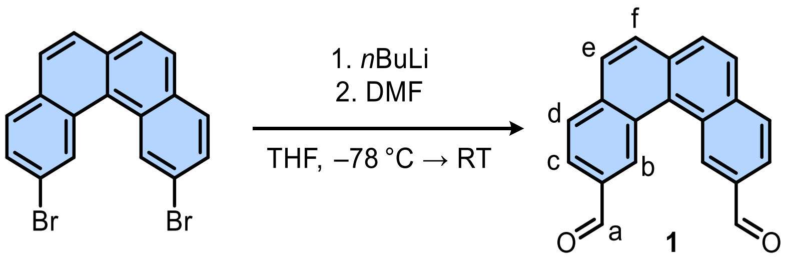

Benzo[c]phenanthrene-2,11-dicarbaldehyde (**1**). Under an inert atmosphere, 2,11-dibromobenzo[c]phenanthrene (4.15 g, 10.7 mmol, 1.00 eq.) was dissolved in dry THF (50 mL) in a Schlenk tube. The solution was cooled to −89 °C by an isopropanol / liquid nitrogen bath and a 2.25 M solution of *n*-butyllithium in hexane (15.3 mL, 34.4 mmol, 3.20 eq.) was added to the reaction mixture dropwise over a period of 20 min by syringe pump. The resulting cloudy suspension was stirred for 5 min at −89 °C. Dry *N*,*N*-dimethylformamide (1.44 mL, 18.6 mmol, 6.00 eq.) was added over 5 min and the suspension was stirred for an additional 45 min. The temperature was then increased to −15 °C and stirring continued for one more hour at −15 °C and 45 min at room temperature. The reaction mixture was quenched with 30 mL of saturated aqueous ammonium chloride solution and stirred at room temperature for 1 h. The resulting suspension was poured onto ice water in a separation funnel and extracted with DCM (2 × 50 mL). The combined organic phases were dried over $MgSO_4$, brought to dryness *in vacuo* and the obtained yellow oily crude product was purified by column chromatography on silica gel (DCM) to yield **1** as an off-white powder (2.87 g, 10.1 mmol, 94%).

Melting point: 190 °C.

^{1}H NMR (400 MHz, DMSO–d$_6$, rt): δ [ppm] = 10.36 (d, J = 0.6 Hz, 2H, H$_a$), 9.69 (s, 2H, H$_b$), 8.35 (d, J = 8.2 Hz, 2H, H$_d$), 8.28 – 8.22 (–, 4H H$_{e/f}$), 8.14 (dd, J = 8.3, 1.1 Hz, 2H, H$_c$).

^{13}C NMR (101 MHz, DMSO–d$_6$, rt): δ [ppm] = 194.0 (CH), 136.6 (C$_q$), 134.5 (C$_q$), 134.1 (CH), 131.6 (C$_q$), 130.0 (CH), 129.9 (CH), 128.8 (C$_q$), 128.2 (CH), 127.7 (C$_q$), 123.2 (CH).

HRMS (ESI): m / z: calculated $[C_{20}H_{12}O_2Na]^+$ 307.0730; found 307.0729 (|Δ| = 0.3 ppm).

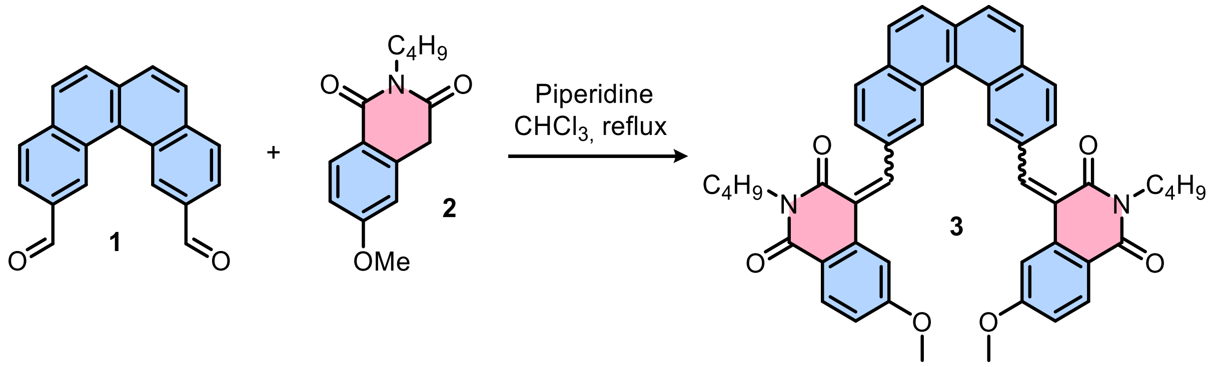

Compound **3**. 2-butyl-6-methoxyisoquinoline-1,3(2H,4H)-dione (**2**, 109 mg, 439 µmol, 2.50 eq.)[1] and **1** (50.0 mg, 176 µmol, 1.00 eq.) were dissolved in chloroform (20 mL) under N_2 atmosphere. Piperidine (0.10 mL) was added, and the mixture was heated to reflux for 6 h. The solvent was evaporated under reduced pressure and the residue was purified by column chromatography on silica gel (DCM / 2% EtOAc). The target compound was obtained as a mixture of three isomers, *E,E*- *E,Z*- and *Z,Z* as an orange amorphous solid (56.0 mg, 75.8 µmol, 44%).

Note: A useful NMR spectrum could not be recorded as the three isomers could not be separated by column chromatography and it was impossible to unambiguously assign the peaks in the NMR spectrum of the isomeric mixture.

HRMS (MALDI): m / z: calculated $[C_{48}H_{42}N_2O_6+H]^+$ 743.31156; found 743.31280 ($|\Delta|$ = 1.67 ppm).

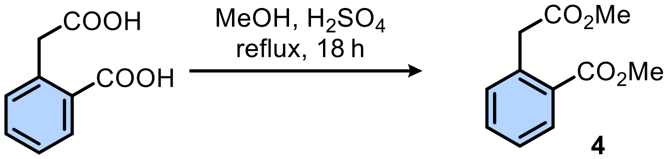

Dimethyl homophthalate (**4**). Homophthalic acid (10.0 g, 55.5 mmol, 1.00 eq.) was dissolved in methanol (50 mL) and concentrated sulfuric acid (3.0 mL) was added. The mixture was heated to reflux for 18 h and cooled to room temperature. The solution was quenched with saturated aqueous $NaHCO_3$, and the aqueous phase was extracted with DCM (3 × 100 mL). The combined organic phases were washed with a concentrated solution of $NaHCO_3$ (10 mL) and brine (2 × 20 mL). After drying over Na_2SO_4, the solvent was evaporated under reduced pressure to yield **4** as an oily, colorless liquid (11.4 g, 54.8 mmol, 99%).

^{1}H NMR (400 MHz, $CDCl_3$, rt): δ [ppm] = 8.01 (dd, J = 7.9, 1.7 Hz, 1H), 7.47 (td, J = 7.5, 1.5 Hz, 1H), 7.35 (td, J = 7.7, 1.4 Hz, 1H), 7.25 (ddd, J = 7.7, 1.3, 0.7 Hz, 1H), 4.00 (s, 2H), 3.85 (s, 3H), 3.68 (s, 3H).

The NMR spectrum agrees with the one from the literature.[7]

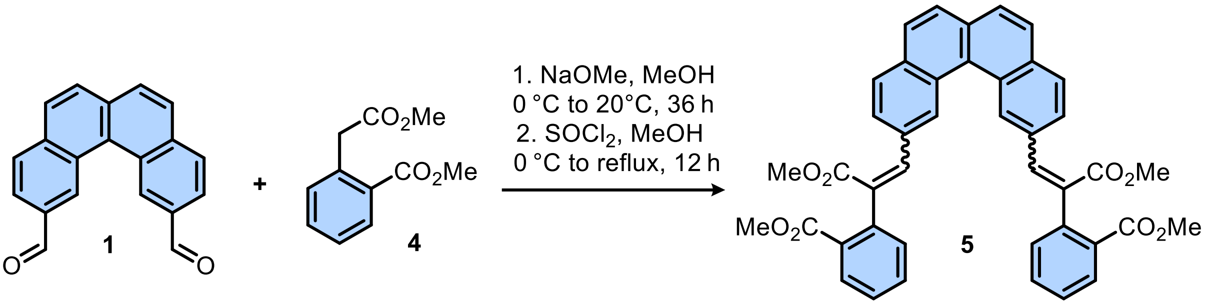

Compound **5**.[8] Under an inert atmosphere, dimethyl homophthalate (**4**) (5.26 g, 25.3 mmol, 2.50 eq.) was dissolved in a freshly prepared solution of NaOMe (5.5 g, 0.10 mol, 10 eq.) in absolute methanol (150 mL). A solution of **1** (2.87 g, 10.1 mmol, 1.00 eq.) in THF (130 mL) was added over 5 min at 0 °C. The solution was then allowed to warm to room temperature and stirred for 36 h, after which a colorless precipitate emerged. The reaction mixture was acidified with 2 M HCl, the suspension was poured onto ice water in a separation funnel and extracted with DCM (3 × 50 mL). The combined organic phases were dried over $MgSO_4$ and the solvent was evaporated. The off-white crude product was then dissolved in methanol (100 mL) and $SOCl_2$ (7.00 mL, 11.5 g, 96.5 mmol, 9.55 eq.) was added dropwise at 0 °C. The mixture was then heated to reflux overnight. The resulting solution was diluted with DCM (200 mL) and washed with water (2 × 100 mL). The organic phase was dried over $MgSO_4$, the solvent was evaporated, and the resulting crude product was purified by column chromatography on silica gel (DCM / 2% EtOAc) to yield **5** as an off-white powder (5.46 g, 8.22 mmol, 82%).

Melting point: 119–122 °C.

^{1}H NMR (400 MHz, $CDCl_3$, rt: δ [ppm] = 8.91 (s, 2H), 8.18 – 8.12 (–, 2H), 8.10 (d, J = 11.9 Hz, 2H), 7.75 (m, 4H), 7.62 (d, J = 8.5 Hz, 2H), 7.51 – 7.39 (–, 4H), 7.36 (d, J = 7.4 Hz, 1H), 7.21 (m, 1H), 6.89 (d, J = 8.3 Hz, 2H), 3.89 (s, 3H), 3.84 (s, 3H), 3.81 (s, 6H).

^{13}C NMR (101 MHz, $CDCl_3$, rt): δ [ppm] = 168.2 (C_q), 168.1 (C_q), 167.3 (C_q), 167.2 (C_q), 138.9 (CH), 138.1 (C_q), 138.0 (C_q), 133.9 (C_q), 133.59 (C_q), 133.56 (C_q), 133.53 (C_q), 133.12 (C_q), 133.07 (CH), 133.0 (C_q), 132.90 (CH), 132.86 (CH), 131.9 (CH), 131.7 (CH), 131.5 (C_q), 131.1 (CH), 130.7 (C_q), 130.5 (C_q), 130.08 (C_q), 130.05 (C_q), 128.4 (CH), 128.0 (CH), 127.9 (CH), 127.6 (C_q), 127.3 (CH), 126.6 (CH), 52.5 (CH_3), 52.34 (CH_3), 52.28 (CH_3).

Note: Due to the asymmetric geometry of the compound, some ^{13}C signals are so close that they could not be resolved with a 101 MHz frequency NMR spectrometer. Therefore, the number of observed signals is different from the number of expected signals.

HRMS (MALDI): m / z: calculated $[C_{42}H_{32}O_8{-}e^-]^+$ 664.20917; found 664.21005 (|Δ| = 1.32 ppm).

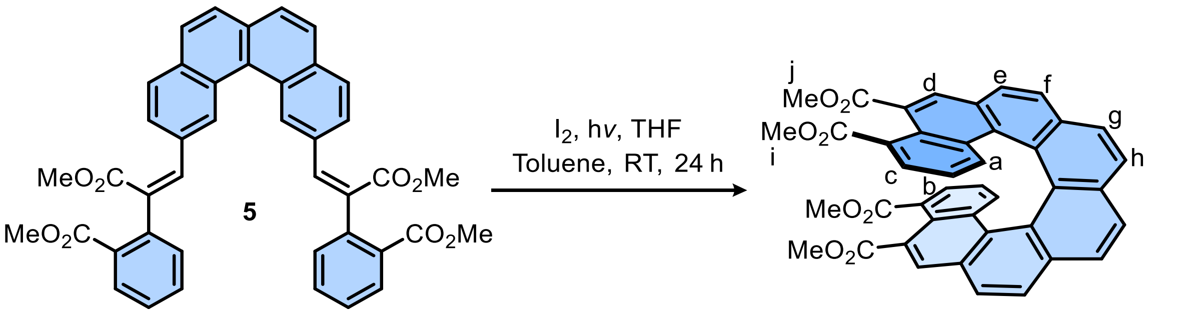

6

Compound **6**. The reaction was performed in a Peschl Ultraviolet GmbH photoreactor with a Heraeus 150 W medium pressure mercury lamp at room temperature. **5** (400 mg, 602 µmol, 1.00 eq.) was dissolved in toluene (1.9 L) and the solution was deoxygenated by vigorous bubbling with N_2 for 1 h. I_2 (335 mg, 1.22 mmol, 2.20 eq) and THF (12 mL) were added, and the reaction mixture was irradiated for 24 h. The residual iodine was quenched with saturated $Na_2S_2O_3$ solution (10 mL), and the volatiles were evaporated under reduced pressure. Purification was performed by column chromatography on silica gel (Cyclohexane / EtOAc 2:1) to yield **6** as a light-yellow powder (197 mg, 298 µmol, 50%).

Melting point: >300 °C (slow decomposition).

^{1}H NMR (400 MHz, $CDCl_3$, rt): δ [ppm] = 8.09 (d, J = 8.2 Hz, 2H, H_h), 8.02 (d, J = 8.2 Hz, 2H, H_g), 7.86 (d, J = 8.2 Hz, 2H, H_f), 7.76 (s, 2H, H_d), 7.49 d, J = 8.4 Hz, 2H, H_e), 7.47 dd, J = 7.2, 1.3 Hz, 2H, H_c), 7.10 (dd, J = 8.5, 1.3 Hz, 2H, H_a), 6.45 (dd, J = 8.5, 7.2 Hz, 2H, H_b), 3.86 (s, 6H, H_j), 3.85 (s, 6H, H_i).

^{13}C NMR (101 MHz, $CDCl_3$, rt) δ [ppm] = 169.1 (C_q), 168.3 (C_q), 133.2 (C_q), 131.0 (C_q), 130.3 (CH), 129.21 (C_q), 129.20 (C_q), 129.1 (C_q), 129.0 (C_q), 128.8 (CH), 128.2 (CH), 127.9 (C_q), 127.7 (CH), 127.5 (CH), 127.4 (CH), 127.3 (CH), 126.8 (C_q), 125.4 (C_q), 125.1 (C_q), 123.4 (CH), 52.0 (CH_3), 51.9 (CH_3).

HRMS (MALDI): m / z: calculated $[C_{42}H_{28}O_8{-}e^-]^+$ 660.17787; found 660.17877 (|Δ| = 1.36 ppm).

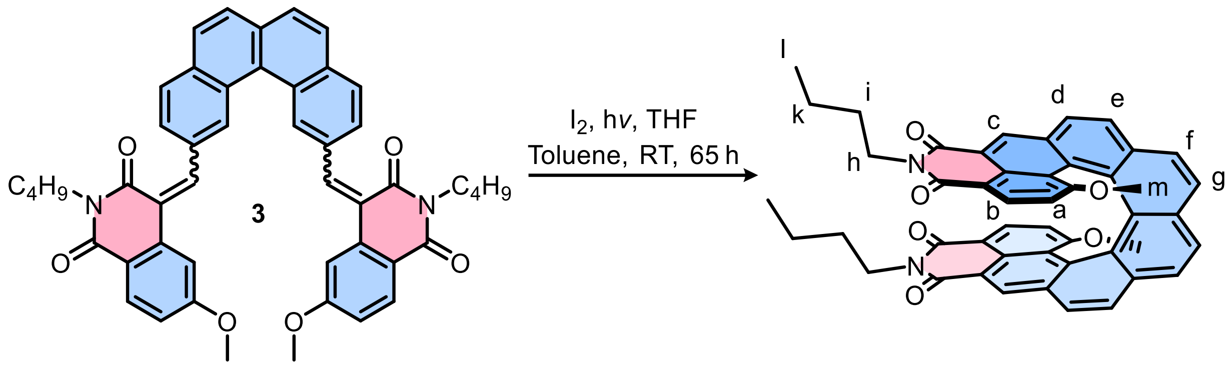

***N-n*Bu[8]HDI-*f*-OMe**. The reaction was performed in a Peschl Ultraviolet GmbH photoreactor with a Heraeus 150 W medium pressure mercury lamp at room temperature. **3** (25 mg, 34 µmol, 1.0 eq.) was dissolved in toluene (250 mL) and the solution was deoxygenated by vigorous bubbling with N_2 for 1 h. I_2 (188 mg, 74.3 µmol, 2.20 eq.) and THF (3 mL) were added, and the reaction mixture was irradiated for 65 h. The residual iodine was quenched with saturated $Na_2S_2O_3$ solution (5 mL), and the volatiles were evaporated under reduced pressure. The orange crude product was purified by column chromatography on silica gel (petroleum ether / EtOAc 2:1) to deliver the title compound as orange crystals (2.1 mg, 2.8 µmol, 8%).

Melting point: >300 °C (slow decomposition).

^{1}H NMR (400 MHz, ($CDCl_3$, rt): δ [ppm] = 8.28 (s, 2H, H_c), 8.18 (d, J = 8.4 Hz, 2H, H_b), 8.08 (d, J = 8.2 Hz, 2H, H_g), 7.95 (d, J = 8.3 Hz, 2H, H_f), 7.89 (d, J = 8.2 Hz, 2H, H_e), 7.56 (d, J = 8.4 Hz, 2H, H_d), 6.19 (d, J = 8.5 Hz, 2H, H_a), 4.15 (m, 4H, H_h), 2.77 (s, 6H, H_m), 1.61 (m, 4H, H_i), 1.43 (m, 4H, H_k), 0.95 (t, J = 7.4 Hz, 6H, H_l).

^{13}C NMR (101 MHz, $CDCl_3$, rt) δ [ppm] = 163.7 (C_q), 163.3 (C_q), 158.6 (C_q), 133.0 (CH), 132.5 (C_q), 131.8 (C_q), 130.8 (CH), 130.2 (C_q), 128.5 (C_q), 128.3 (CH), 127.9 (C_q), 127.7 (CH), 127.0 (CH), 126.9 (C_q), 126.0 (CH), 125.0 (C_q), 120.2 (C_q), 117.3 (C_q), 114.3 (C_q), 105.7 (CH), 55.0 (CH_3), 40.4 (CH_2), 30.4 (CH_2), 20.6 (CH_2), 14.0 (CH_3).

HRMS (MALDI): m / z: calculated $[C_{48}H_{38}N_2O_6{-}e]^+$ 738.27244; found 738.27460 ($|\Delta|$ = 2.93 ppm).

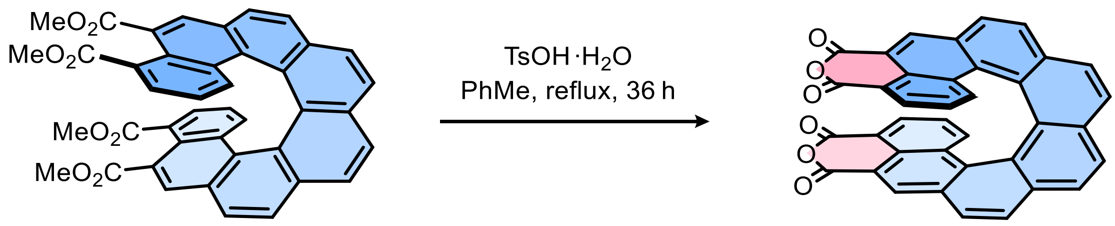

6 **7**

[8]helicene-4,5,16,17-tetracarboxylic dianhydride (**7**). **6** (78.0 mg, 118 µmol, 1.00 eq.) and *p*-toluenesulfonic acid monohydrate (134 mg, 708 µmol, 6.00 eq.) were dissolved in toluene (15 mL) and heated to reflux for 36 h. The bright yellow precipitate was filtered, washed with cold petroleum ether and methanol, and dried under high vacuum to yield pure **7** (63.1 mg, 111 µmol, 94%).

Melting point: >350 °C

^{1}H NMR (400 MHz, $CDCl_3$) δ [ppm] = 8.34 (s, 2H), 8.24 (d, J = 8.3 Hz, 2H), 8.17 – 8.11 (–, 4H), 8.02 (d, J = 8.2 Hz, 2H), 7.65 (d, J = 8.3 Hz, 2H), 7.44 (dd, J = 8.5, 1.1 Hz, 2H), 6.77 (dd, J = 8.5, 7.3 Hz, 2H).

^{13}C NMR (101 MHz, $CDCl_3$, rt) δ [ppm] = Not possible to record due to low solubility.

HRMS (MALDI): m / z: calculated $[C_{38}H_{16}O_6+e^-]^-$ 568.0952; found 568.09550 (|Δ| = 0.53 ppm).

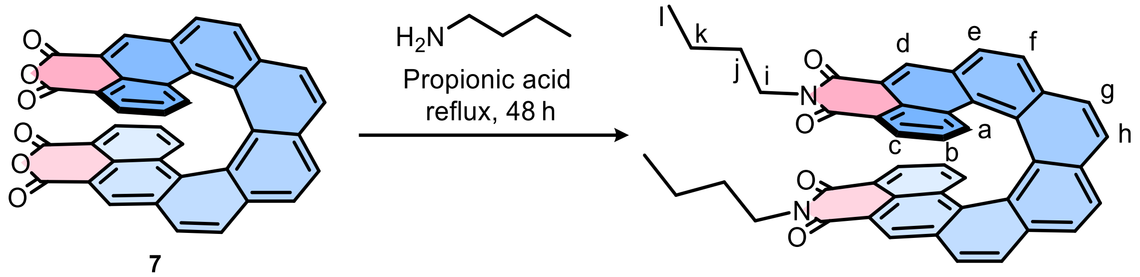

7

***N*-*n*-Bu-[8]HDI**. **7** (10.0 mg, 17.6 µmol, 1.00 eq) and *n*-butylamine (40.0 mg, 547 µmol, 31.1 eq) were dissolved in propionic acid (10 mL) and the mixture was heated to reflux for 48 h. The resulting yellow solution was diluted with DCM (50 mL) and neutralized with saturated $NaHCO_3$ solution in a separation funnel. The aqueous phase was extracted with DCM (2 × 20 mL). The combined organic phases were dried over $MgSO_4$, filtered, and the volatiles were evaporated. The bright yellow crude product was purified by column chromatography on silica gel (DCM / EtOAc 9:1) and reprecipitated from DCM / MeOH to yield the title compound as bright yellow crystals (9.81 mg, 14.4 µmol, 82%).

Melting point: >300 °C (slow decomposition).

^{1}H NMR (400 MHz, CD_2Cl_2, rt): δ [ppm] = 8.18 (d, J = 8.2 Hz, 2H, H_h), 8.17 (s, 2H, H_d), 8.08 (d, J = 8.2 Hz, 2H, H_g), 8.06 (dd, J = 7.3, 1.1 Hz, 2H, H_c), 7.94 (d, J = 8.3 Hz, 2H, H_f), 7.60 (d, J = 8.3 Hz, 2H, H_e), 7.34 (dd, J = 8.4, 1.2 Hz, 2H, H_a), 6.69 (dd, J = 8.4, 7.3 Hz, 2H, H_b), 4.05 (m, 4H, H_i), 1.60 (m, 4H, H_j), 1.43 (m, 4H, H_k), 0.98 (t, J = 7.3 Hz, 6H, H_l).

^{13}C NMR (101 MHz, CD_2Cl_2, rt) δ [ppm] = 164.2 (C_q), 163.4 (C_q), 134.6 (C_q), 132.6 (CH), 132.3 (C_q), 130.7 (C_q), 130.5 (C_q), 129.9 (CH), 129.2 (CH), 129.0 (CH), 128.7 (CH), 128.5 (CH), 128.1 (CH), 126.6 (C_q), 126.5 (C_q), 126.2 (C_q), 125.5 (CH), 124.6 (C_q), 122.0 (C_q), 120.4 (C_q), 40.7 (CH_2), 30.7 (CH_2), 21.0 (CH_2), 14.2 (CH_3).

HRMS (MALDI): m / z: calculated $[C_{46}H_{34}N_2O_4+e^-]^-$ 678.25241; found 678.2530 (|Δ| = 0.87 ppm).

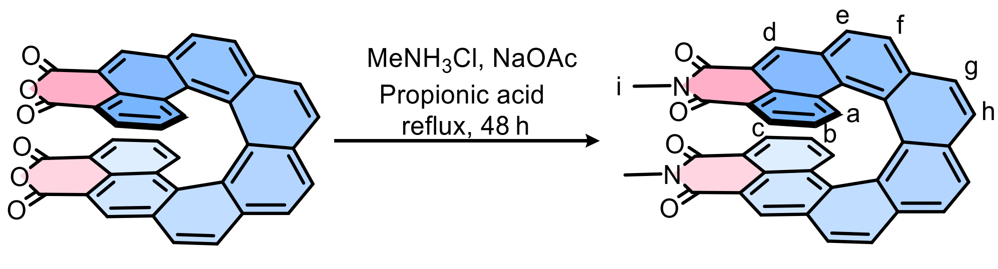

7

***N*-Me-[8]HDI**. **7** (136 mg, 239 µmol, 1.00 eq), methylammonium chloride (323 mg, 4.78 mmol, 20.0 eq), and sodium acetate (400 mg, 4.88 mmol, 20.4 eq.) were dissolved in propionic acid (100 mL) and the mixture was heated to reflux for 48 h. The resulting yellow solution was poured on ice water and neutralized with Na_2CO_3 solution in a separation funnel.

The aqueous phase was extracted with DCM (3 × 50 mL). The combined organic phases were dried over $MgSO_4$, filtered, and the volatiles were evaporated. The bright yellow crude product was reprecipitated from DCM / MeOH to yield the title compound as bright yellow crystals (131 mg, 220 µmol, 92%). The reaction was performed in the same way for both racemic and enantiomerically pure starting materials to racemic and enantiopure ***N*-Me-[8]HDI**, respectively.

Melting point: >300 °C (slow decomposition).

^{1}H NMR (400 MHz, CD_2Cl_2, rt): δ [ppm] = 8.17 (d, J = 8.3 Hz, 2H, H_h), 8.15 (s, 2H, H_d), 8.10 – 8.04 (–, 4H, H_c, H_g), 7.92 (d, J = 8.5 Hz, 2H, H_f), 7.57 (d, J = 8.6 Hz, 2H, H_e), 7.34 (dd, J = 8.4, 1.1 Hz, 2H, H_b), 6.71 (dd, J = 8.4, 7.3 Hz, 2H, H_a), 3.45 (s, 6H, H_i).

^{13}C NMR (101 MHz, CD_2Cl_2, rt) δ [ppm] =164.50 (C_q), 163.78 (C_q), 134.66 (C_q), 132.54 (CH), 132.17 (C_q), 130.71 (C_q), 130.39 (C_q), 130.05 (CH), 129.23 (CH), 128.90 (CH), 128.63 (CH), 128.38 (CH), 127.95 (CH), 126.58 (C_q), 126.41 (C_q), 126.07 (C_q), 125.63 (CH), 124.41 (C_q), 121.73 (C_q), 120.17 (C_q), 27.22 (CH_3).

HRMS (MALDI): m / z: calculated $[C_{40}H_{22}N_2O_4+e^-]^-$ 594.15851; found 594.15738 ($|\Delta|$ = 1.90 ppm).

General procedure for the synthesis of bridge-[8]HDIs

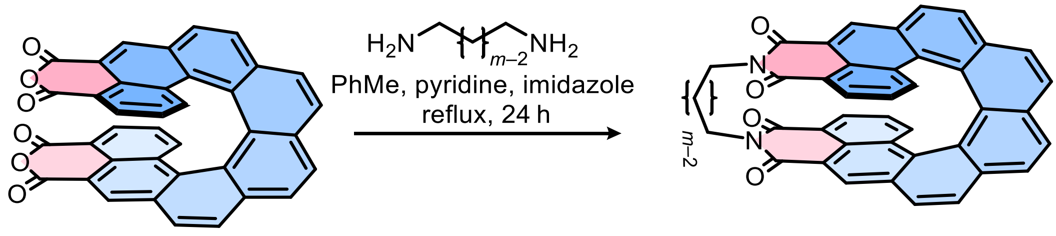

7 **C*m*-[8]HDI**

7 was dissolved in toluene together with pyridine (2 % per volume) and imidazole (2 mg ml^{-1}), and the mixture was heated to its boiling point. The appropriate diamine was added as a solution in toluene (1 mg ml^{-1}) over a period of four hours by a syringe pump. The mixture was then stirred to reflux for a further 20 h. The resulting suspension was diluted with DCM until the suspended solid had dissolved and was then washed with 2 N hydrochloric acid. The organic phase was dried over $MgSO_4$ and the volatiles were evaporated. The bright yellow crude product was purified by column chromatography on silica gel (DCM / EtOAc 9:1) and reprecipitated from $CHCl_3$ / MeOH to yield the title compounds as bright yellow crystals.

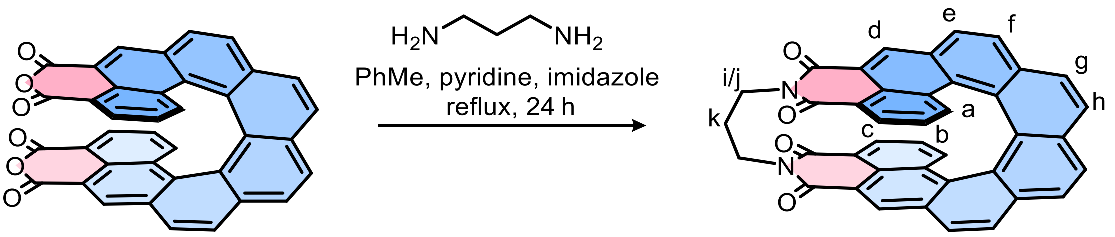

7

C3-[8]HDI. The reaction was performed according to the general procedure for the synthesis of bridge-**[8]HDI**s. **7** (10.0 mg, 17.6 µmol, 1.00 eq), toluene (100 mL), pyridine (2.0 mL), imidazole (200 mg), 1,3-propanediamine (1.30 mg, 17.6 µmol, 1.00 eq). Yield: 6.10 mg, 10.0 µmol, 57%. The reaction was performed in the same way for both racemic and enantiomerically pure starting materials to obtain racemic and enantiopure **C3-[8]HDI**, respectively.

Melting point: >300 °C (slow decomposition).

^{1}H NMR (400 MHz, CD_2Cl_2, rt): δ [ppm] = 8.19 (s, 2H, H_d), 8.16 (d, J = 8.3 Hz, 2H, H_h), 8.12 – 8.02 (–, 4H, H_c, H_g), 8.06 (d, J = 8.3 Hz, 2H, H_c), 7.98 (d, J = 8.5 Hz, 2H, H_f), 7.63 (d, J = 8.3 Hz, 2H, H_e), 7.50 (dd, J = 8.3, 1.2 Hz, 2H, H_a), 6.68 (dd, J = 8.3, 7.4 Hz, 2H, H_b), 4.51 (m, 2H, $H_{i/j}$), 4.40 (m, 2H, $H_{i/j}$), 2.61 (bs, 2H, H_k)

^{13}C NMR (101 MHz, CD_2Cl_2, rt) δ [ppm] = 164.3 (C_q), 164.0 (C_q), 135.1 (C_q), 133.2 (CH), 132.9 (C_q), 131.1 (C_q), 131.0 (C_q), 129.7 (CH), 129.4 (CH), 128.8 (CH), 128.5 (CH), 128.4 (CH), 128.3 (CH), 127.0 (C_q), 126.8 (C_q), 126.4 (C_q), 125.1 (CH), 124.4 (C_q), 122.5 (C_q), 121.0 (C_q), 40.1 (CH_2), 21.3 (CH_2).

HRMS (MALDI): m / z: calculated $[C_{41}H_{22}N_2O_4+e^-]^-$ 606.15850; found 606.15774 ($|\Delta|$ = 1.25 ppm).

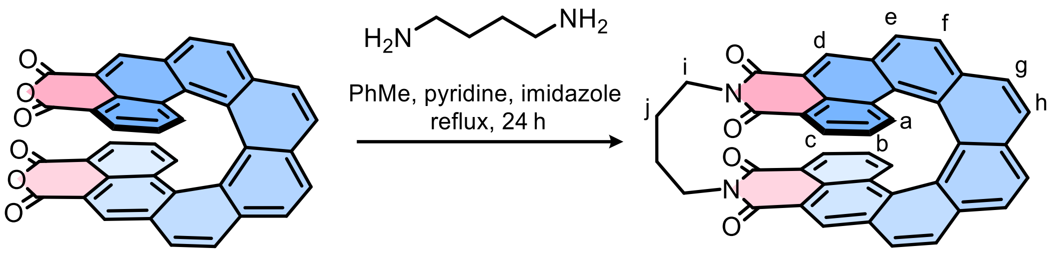

7

C4-[8]HDI. The reaction was performed according to the general procedure for the synthesis of bridge-**[8]HDI**s. **7** (10.0 mg, 17.6 µmol, 1.00 eq), toluene (100 mL), pyridine (2.0 mL), imidazole (200 mg), 1,4-butanediamine (1.55 mg, 17.6 µmol, 1.00 eq). Yield: 5.13 mg (8.27 mmol, 47%). The reaction was performed in the same way for both racemic and enantiomerically pure starting materials to obtain racemic and enantiopure **C4-[8]HDI**, respectively.

Melting point: >300 °C (slow decomposition).

^{1}H NMR (400 MHz, $CDCl_3$, rt): δ [ppm] = 8.28 (s, 2H, H_d), 8.14 (d, J = 8.3 Hz, 2H, H_h), 8.11 (dd, J = 7.3, 1.2 Hz, 2H, H_c), 8.07 (d, J = 8.3 Hz, 2H, H_g), 7.96 (d, J = 8.2 Hz, 2H, H_f), 7.64 (d, J = 8.2 Hz, 2H, H_e), 7.49 (dd, J = 8.4, 1.1 Hz, 2H, H_a), 6.68 (dd, J = 8.4, 7.3 Hz, 2H, H_b), 4.29 (m, 2H, H_i), 4.17 (m, 2H, H_i), 2.26 (m, 4H, H_j).

^{13}C NMR (101 MHz, $CDCl_3$, rt) δ [ppm] = 164.1 (C_q), 163.9 (C_q), 134.4 (C_q), 133.0 (CH), 132.2 (C_q), 130.54 (C_q), 130.53 (C_q), 129.2 (CH), 128.8 (CH), 128.71 (CH), 128.69 (CH), 128.2 (CH), 128.0 (CH), 126.3 (C_q), 126.22 (C_q), 126.15 (C_q), 124.6 (CH), 124.2 (C_q), 121.8 (C_q), 120.2 (C_q), 41.1 (CH_2), 26.7 (CH_2).

HRMS (MALDI): m / z: calculated $[C_{42}H_{24}N_2O_4-e^-]^+$ 620.173059; found 620.17325 ($|\Delta|$ = 0.3 ppm).

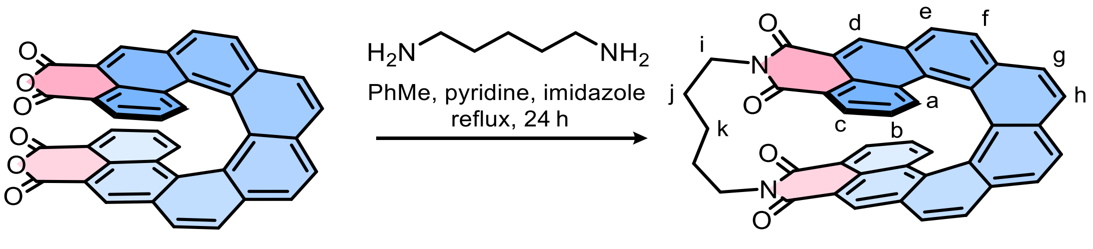

7

C_5-[8]HDI. The reaction was performed according to the general procedure for the synthesis of bridge-**[8]HDI**s. **7** (10.0 mg, 17.6 µmol, 1.00 eq.), toluene (100 mL), pyridine (2.0 mL), imidazole (200 mg), 1,5-pentanediamine (1.80 mg, 17.6 µmol, 1.00 eq). Yield: 5.51 mg (8.68 µmol, 49%). The reaction was performed in the same way for both racemic and enantiomerically pure starting materials to obtain racemic and enantiopure **C_5-[8]HDI**, respectively.

Melting point: >300 °C (slow decomposition).

^{1}H NMR (400 MHz, CD_2Cl_2, rt): δ [ppm] = 8.32 (s, 2H, H_d), 8.21 (d, J = 8.3 Hz, 2H, H_h), 8.12 (d, J = 8.2 Hz, 2H, H_g), 8.04 (d, J = 8.5 Hz, 2H, H_f), 8.02 (dd, J = 7.3, 1.1 Hz, 2H, H_c), 7.73 (dd, J = 8.2, 0.5 Hz, 2H, H_e), 7.43 (dd, J = 8.5, 1.1 Hz, 2H, H_a), 6.68 (dd, J = 8.5, 7.3 Hz, 2H, H_b), 4.24 (ddd, J = 13.3, 8.7, 4.8 Hz, 2H, H_i), 4.16 (ddd, J = 13.3, 5.9, 5.0 Hz, 2H, H_i), 1.90 (m, 2H, H_j), 1.82 (m, 2H, H_j), 1.14 (dt, J = 16.1, 8.4 Hz, 2H, H_k).

^{13}C NMR (101 MHz, CD_2Cl_2, rt): δ [ppm] = 164.5 (C_q), 164.1 (C_q), 134.8 (C_q), 133.2 (CH), 132.81 (C_q), 130.76 (C_q), 130.73 (C_q), 129.9 (CH), 129.5 (CH), 129.09 (CH), 129.08 (CH), 129.0 (CH), 128.5 (CH), 126.9 (C_q), 126.6 (C_q), 126.3 (C_q), 125.2 (CH), 125.0 (C_q), 122.0 (C_q), 120.5 (C_q), 39.2 (CH_2), 27.0 (CH_2), 22.0 (CH_2).

HRMS (MALDI: m / z: calculated $[C_{43}H_{26}N_2O_4{-}e^-]^+$ 634.18871; found 634.18960 (|Δ| = 1.40 ppm).

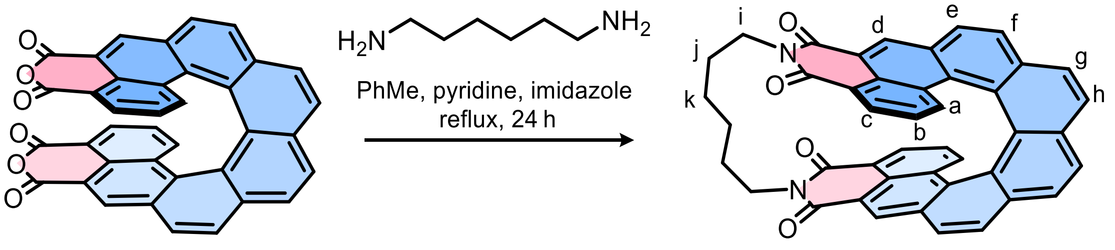

7

C_6-[8]HDI. The reaction was performed according to the general procedure for the synthesis of bridge-**[8]HDI**s. **7** (10.0 mg, 17.6 µmol, 1.00 eq), toluene (100 mL) together with pyridine (2.0 mL) and imidazole (200 mg), 1,6-hexanediamine (2.04 mg, 17.6 µmol, 1.00 eq). Yield: 4.65 mg (7.17 µmol, 41%). The reaction was performed in the same way for both racemic and enantiomerically pure starting materials to obtain racemic and enantiopure **C_6-[8]HDI**, respectively.

Melting point: >300 °C (slow decomposition).

^{1}H NMR (400 MHz, $CDCl_3$, rt): δ [ppm] = 8.34 (s, 2H, H_d), 8.19 (d, J = 8.2 Hz, 2H, H_h), 8.11 (d, J = 8.2 Hz, 2H, H_g), 8.07 (dd, J = 7.3, 1.2 Hz, 2H, H_c), 8.00 (d, J = 8.3 Hz, 2H, H_f), 7.71 (d, J = 8.3 Hz, 2H, H_e), 7.39 (dd, J = 8.5, 1.2 Hz, 2H, H_a), 6.69 (dd, J = 8.5, 7.3 Hz, 2H, H_b), 4.31 (ddd, J = 13.6, 10.9, 3.2 Hz, 2H, H_i), 4.03 (dt, J = 13.1, 4.2 Hz, 2H, H_i), 1.94 (m, 2H, H_j), 1.82 (m, 2H, H_j), 1.48 (m, 4H, H_k).

^{13}C NMR (101 MHz, $CDCl_3$, rt) δ [ppm] = 164.2 (C_q), 163.9 (C_q), 134.1 (C_q), 132.9 (CH), 132.0 (C_q), 130.5 (C_q), 130.2 (C_q), 129.9 (CH), 129.6 (C_q), 129.0 (CH), 128.9 (CH), 128.7 (CH), 128.3 (CH), 128.0 (CH), 126.5 (C_q), 126.0 (C_q), 124.9 (CH), 124.8 (C_q), 121.6 (C_q), 120.2 (C_q), 41.6 (CH_2), 28.6 (CH_2), 27.9 (CH_2).

HRMS (MALDI: m / z: calculated $[C_{44}H_{28}N_2O_4{-}e^-]^+$ 648.20436; found 648.20423 (|Δ| = 0.20 ppm).

S3. Spectroscopy

a) b)

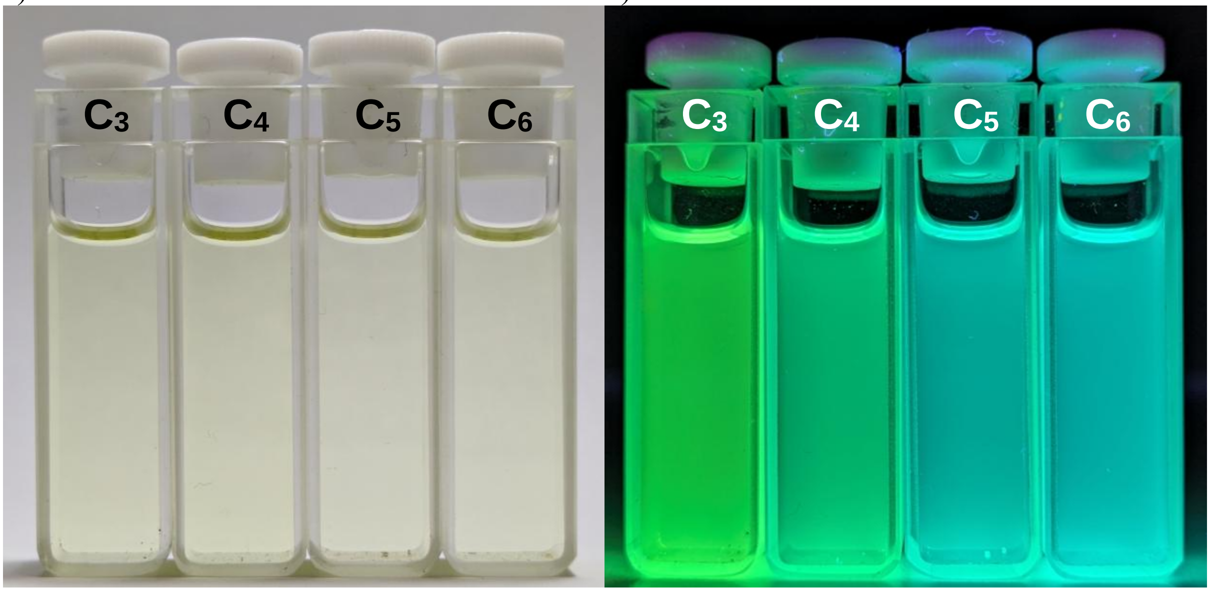

Figure S1. Photographs of **C*m*-[8]HDI** (*m* = 3–6) under (a) ambient light and (b) under 365 nm UV light. The compounds were dissolved in toluene ($c \sim 10^{-4}$ M).

a) b)

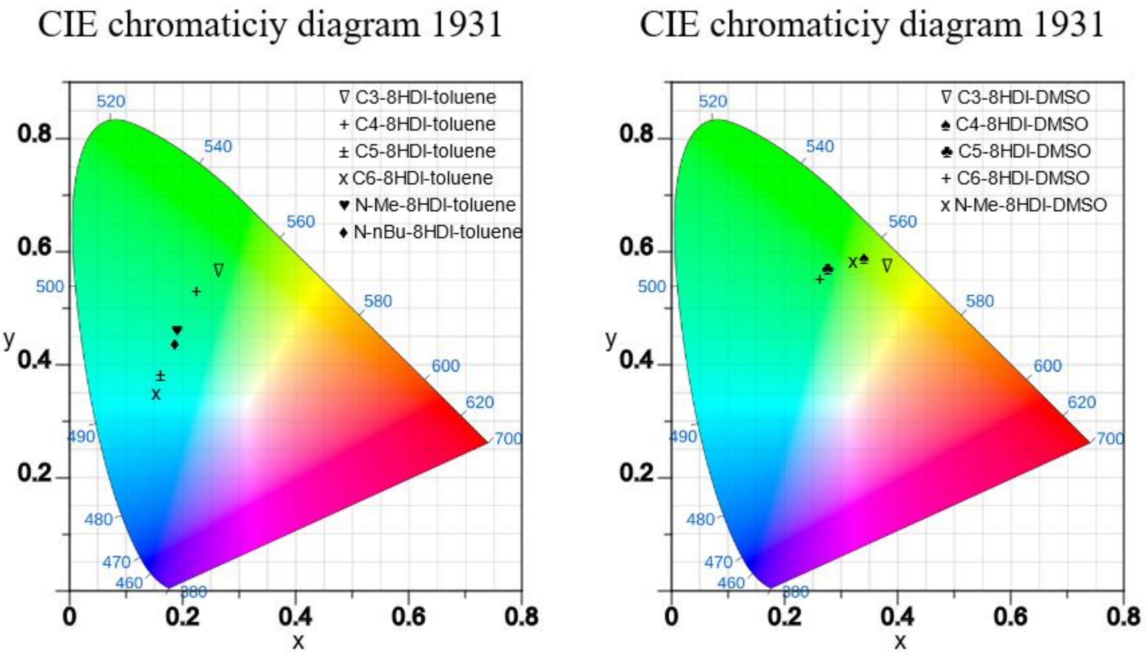

Figure S2. Photoluminescence color coordinates of **C*m*-[8]HDIs** and the non-bridged reference compounds in (a) toluene and (b) DMSO in the CIE 1931 chromaticity diagram.[9] The diagrams were made with the CIE 1931 web-based app by E. H. H. Hasabeldaim (https://sciapps.sci-sim.com/CIE1931.html).

S3.1. Fluorescence Lifetime Spectroscopy

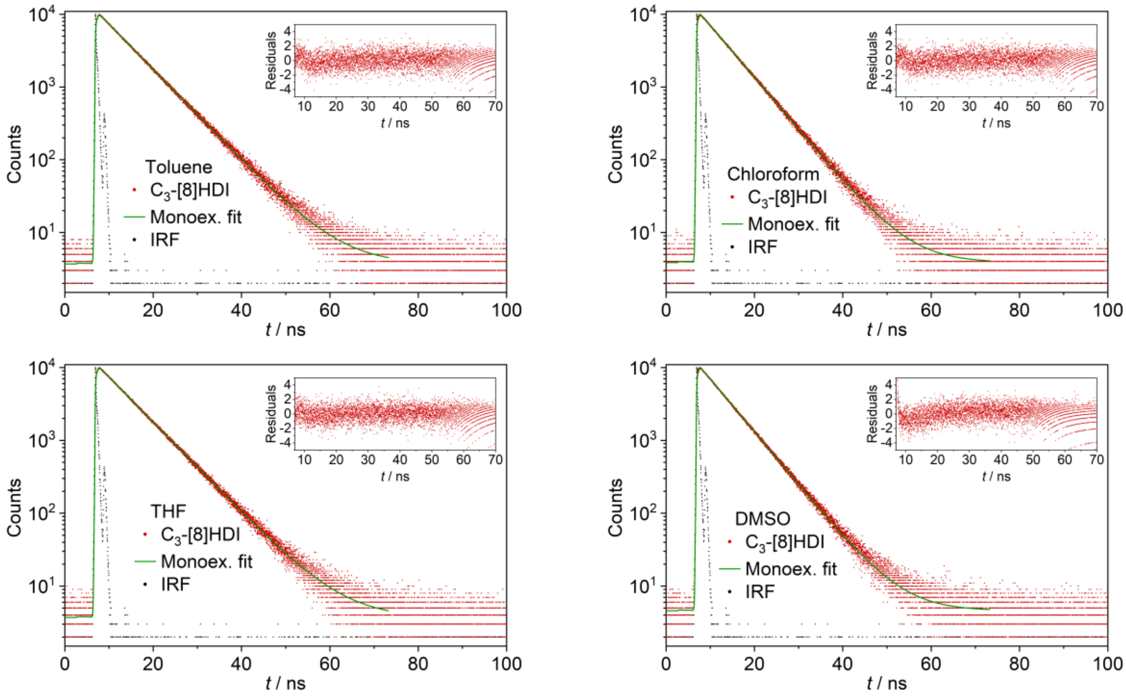

Figure S3. Time-resolved fluorescence decay of **C3-[8]HDI** in different solvents ($c \sim 10^{-6}$ M).

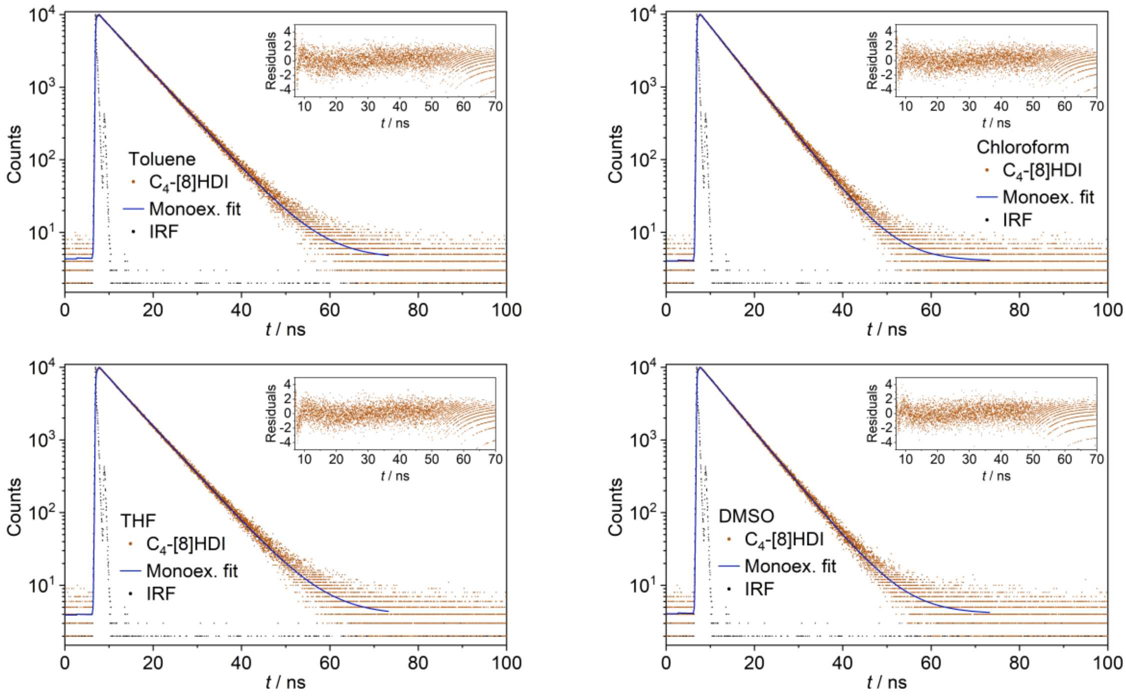

Figure S4. Time-resolved fluorescence decay of **C4-[8]HDI** in different solvents ($c \sim 10^{-6}$ M).

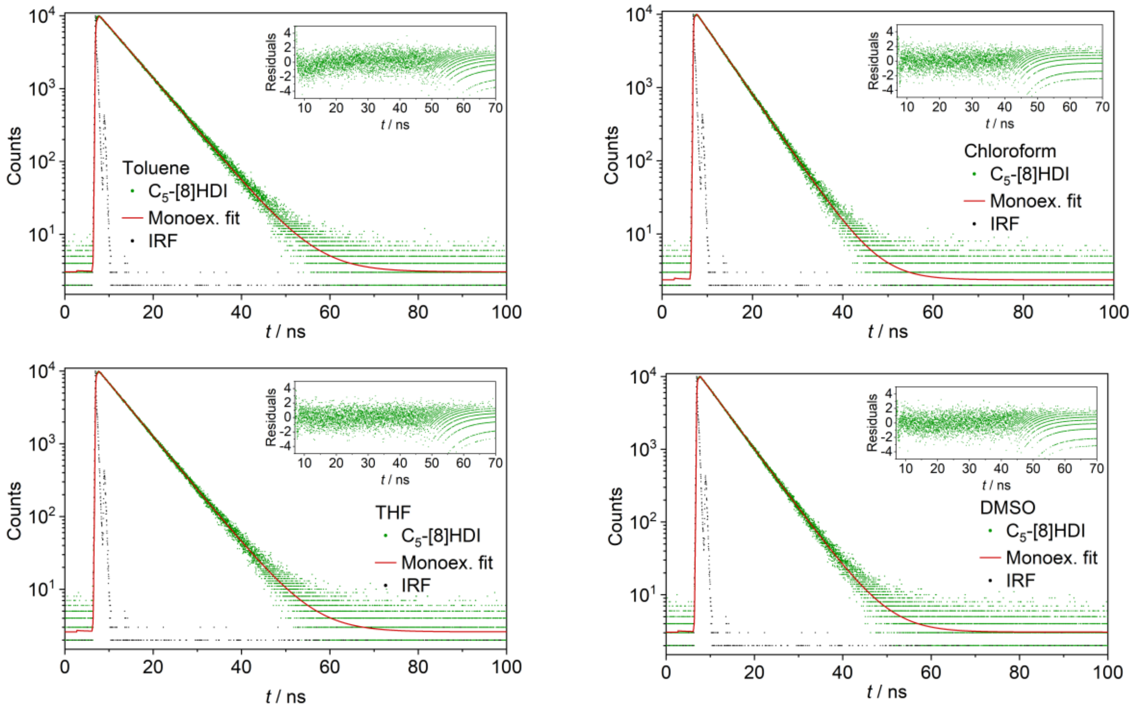

Figure S5. Time-resolved fluorescence decay of **C5-[8]HDI** in different solvents (c ~ 10^{-6} M).

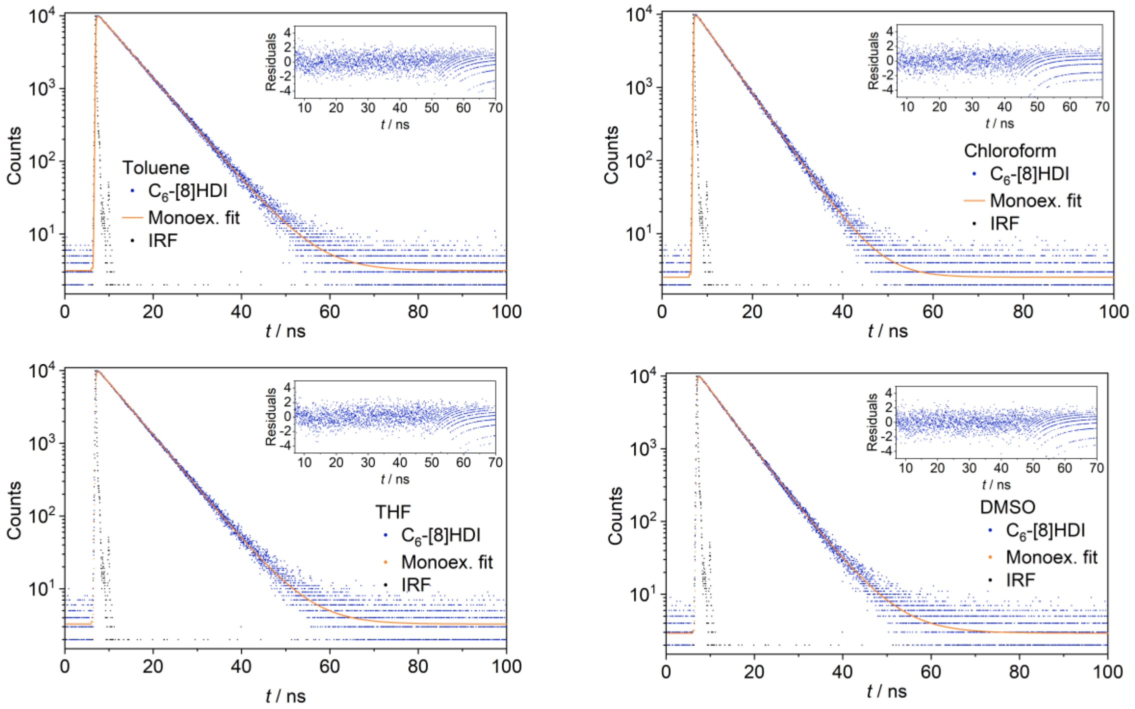

Figure S6. Time-resolved fluorescence decay of **C6-[8]HDI** in different solvents (c ~ 10^{-6} M).

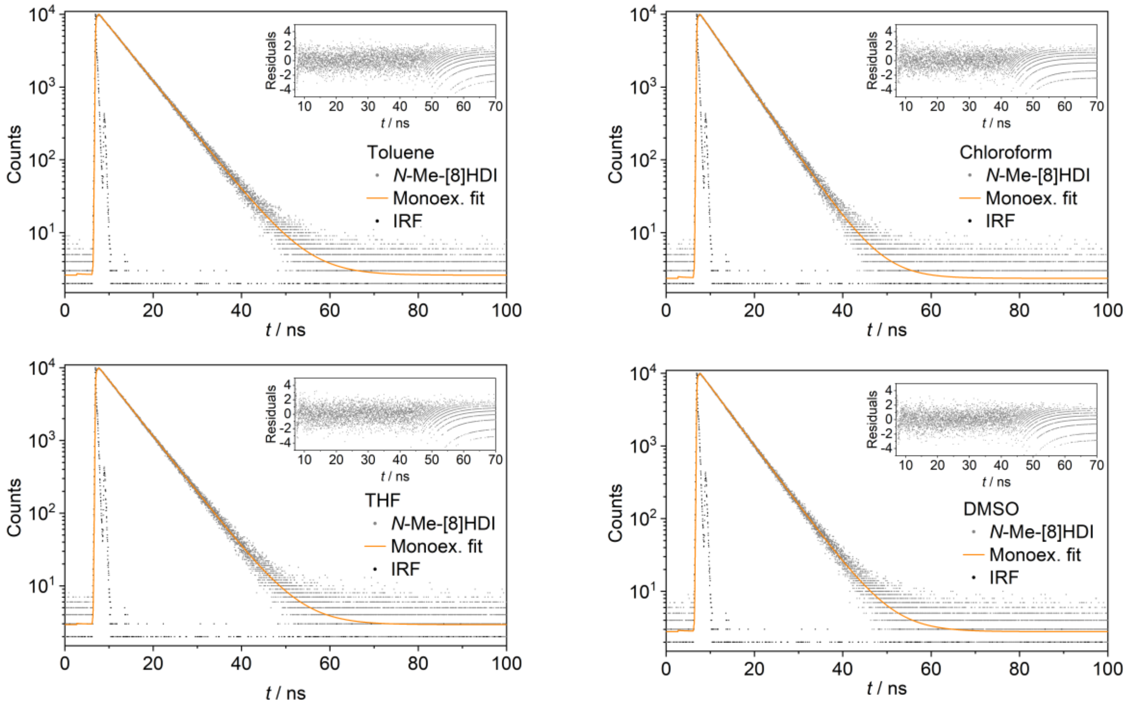

Figure S7. Time-resolved fluorescence decay of ***N*-Me-[8]HDI** in different solvents ($c \sim 10^{-6}$ M).

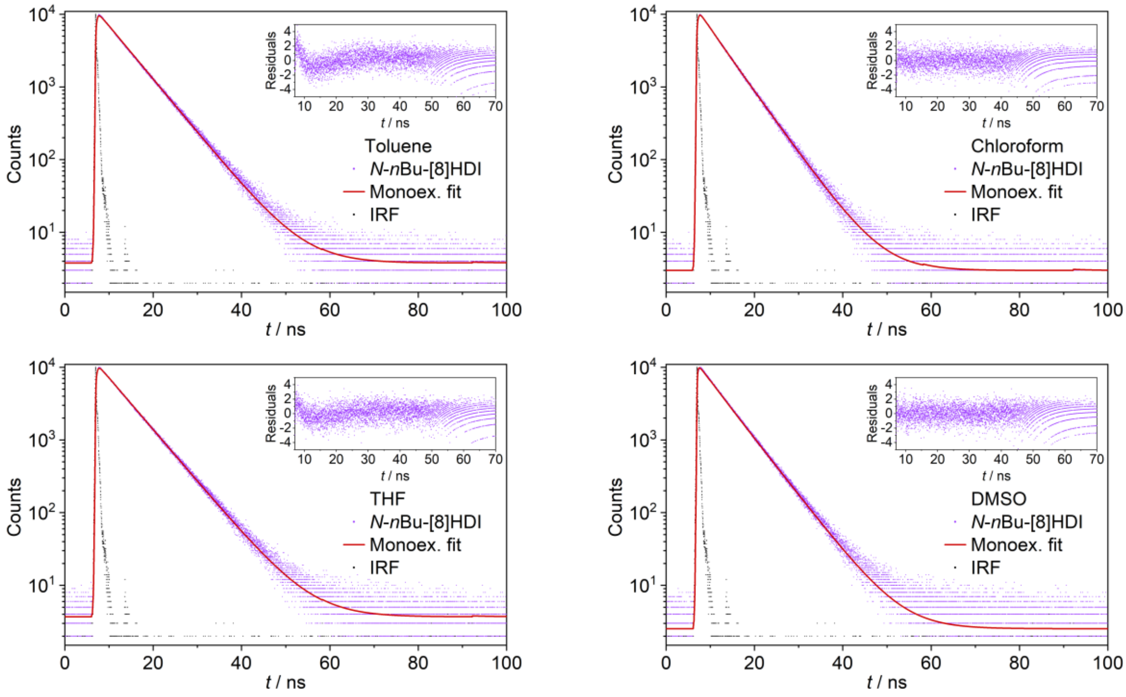

Figure S8. Time-resolved fluorescence decay of ***N*-*n*Bu-[8]HDI** in different solvents ($c \sim 10^{-6}$ M).

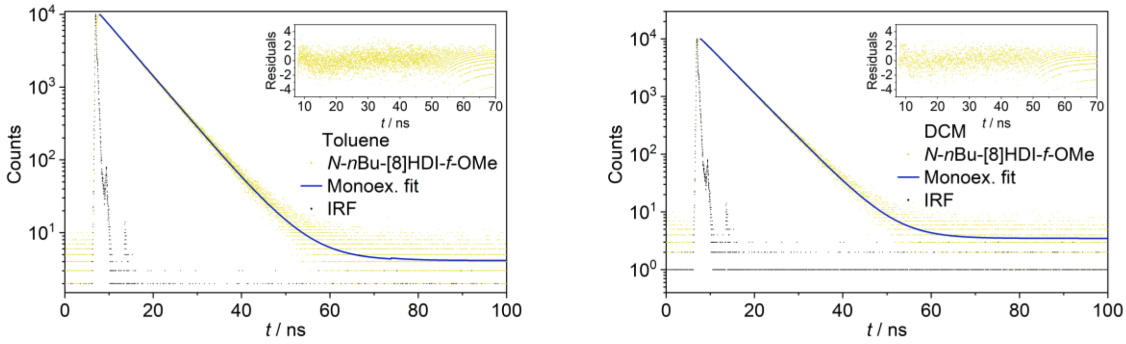

Figure S9. Time-resolved fluorescence decay of ***N*-*n*Bu-[8]HDI-*f*-OMe** in different solvents ($c \sim 10^{-6}$ M).

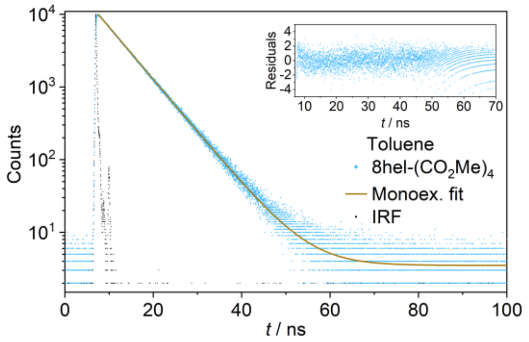

Figure S10. Time-resolved fluorescence decay of **6** in toluene ($c \sim 10^{-6}$ M).

S3.2. Optical and Chiroptical Spectroscopy

The **[8]HDI**s are reasonably soluble in organic solvents of medium polarity, such as DCM and chloroform, and aromatic solvents like toluene, but nearly insoluble in protic (MeOH) or non-polar (hexane) solvents. As expected, solubility generally increases with the length of the alkyl bridge, consequentially ***N*-*n*Bu-[8]HDI** is more soluble than the bridged helicenes or ***N*-Me-[8]HDI**, which does not have the benefit of solubilizing alkyl chains. Enantiopure samples universally show much better solubility compared with their racemic counterparts.

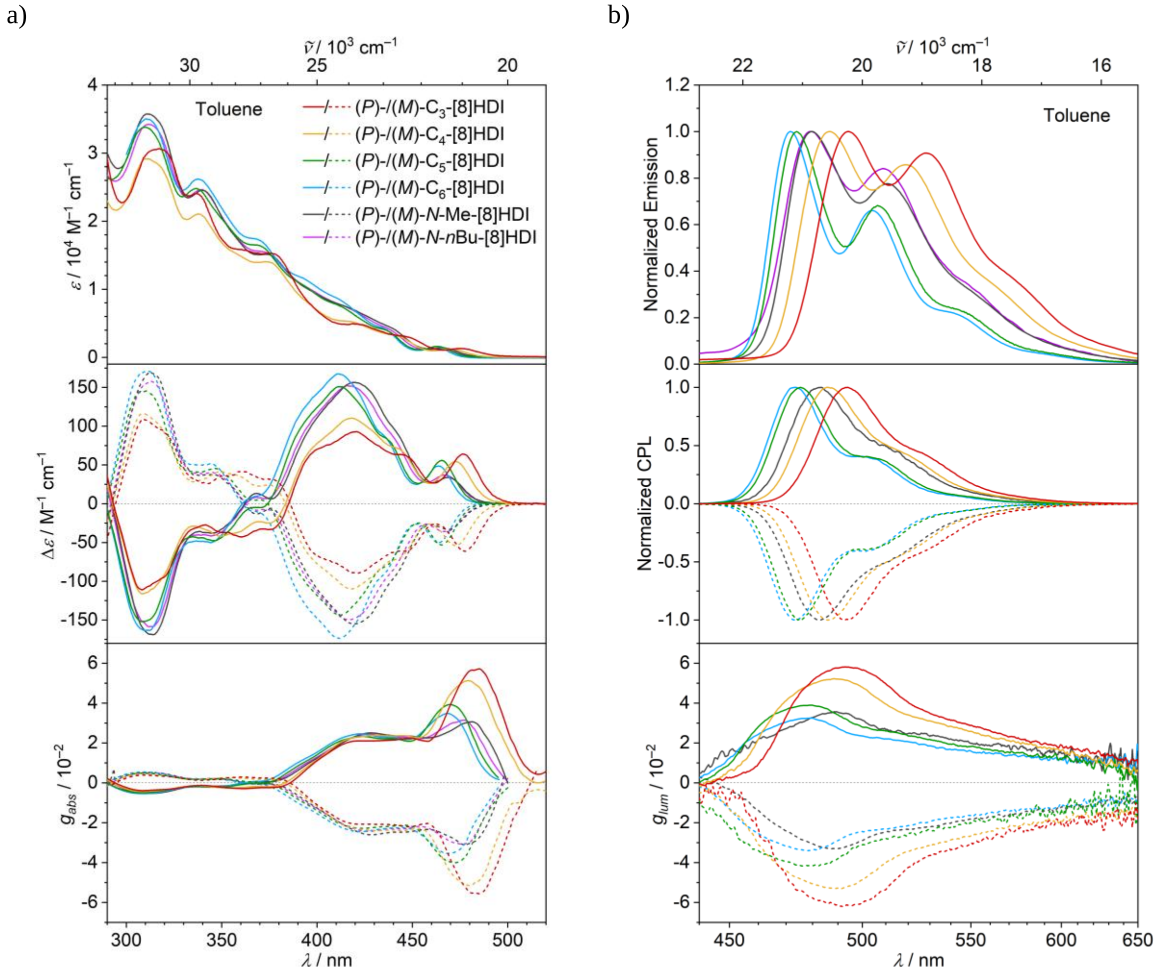

Figure S11. Comparison of (a) (top to bottom) absorption spectra ($c \sim 10^{-5}$ M), circular dichroism ($c \sim 10^{-5}$ M) and absorption dissymmetry factor, and (b) (top to bottom) emission spectra ($c \sim 10^{-6}$ M), CPL spectra ($c \sim 10^{-5}$ M) and luminescence dissymmetry factor of the discussed **[8]HDI**s in toluene.

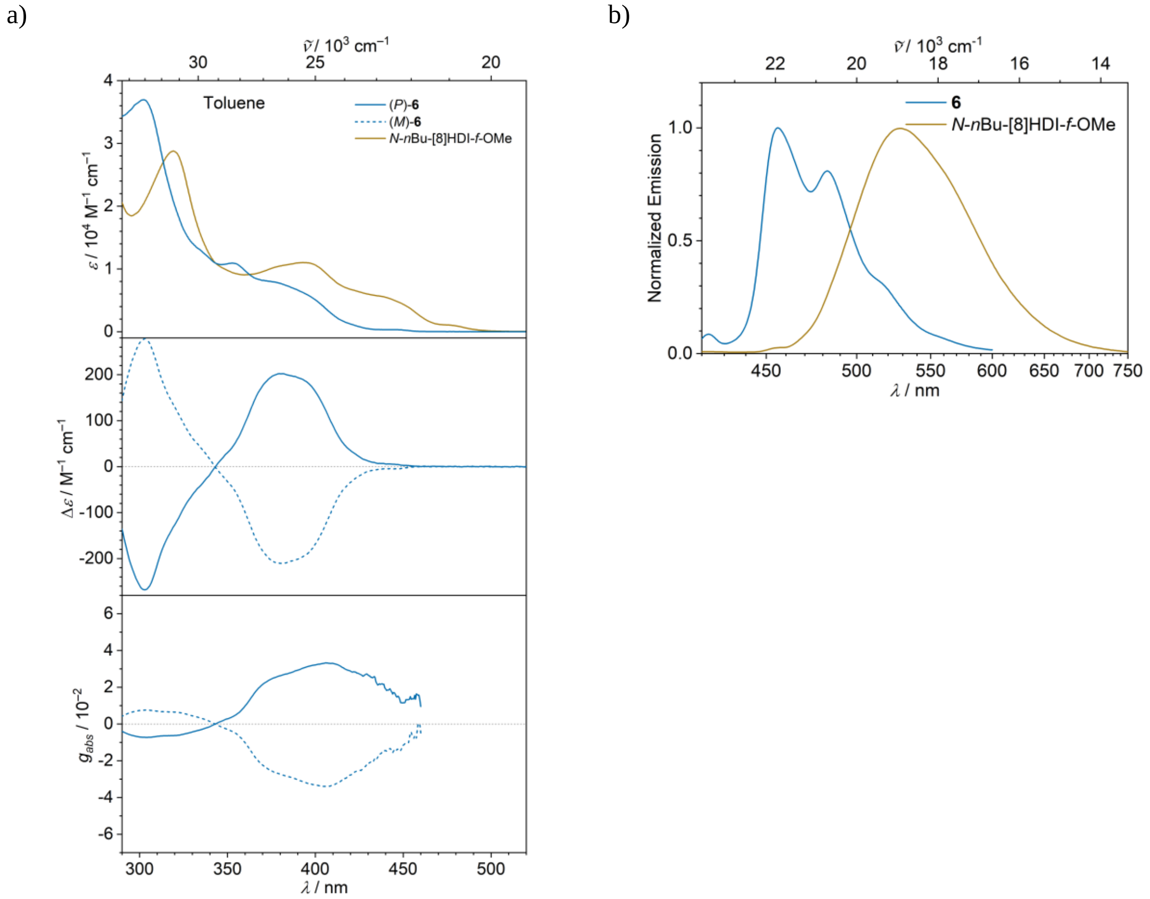

Figure S12. Comparison of (a) (top to bottom) absorption spectra ($c \sim 10^{-5}$ M) for **6** and ***N*-*n*Bu-[8]HDI-*f*-OMe**, and circular dichroism ($c \sim 10^{-5}$ M) and absorption dissymmetry factor for **6** in toluene; and (b) emission spectrum ($c \sim 10^{-6}$ M) of these compounds in toluene.

Table S1. Summary of the optical and chiroptical properties of the discussed compounds and some reference compounds in toluene. The values for **[5]HDI-OMe**, **[6]HDI-OMe** and **[7]HDI-OMe** were measured in DCM and those for carbo-[8]helicene in $CHCl_3$[10] or dioxane.[11]

Compound	λ_{abs} / nm[a)]	ε[b)]	λ_{em} / nm[c)]	Φ_{FL} / %	t_{FL} / ns	k_{FL} / 10^6 s^{-1} [d)]	k_{NR} / 10^8 s^{-1} [e)]	Stokes shift / cm^{-1}	$E_{g(opt)}$ / eV[f)]	Δε / M^{-1} cm^{-1} / nm	$10^2 \times g_{abs}$[g)]	$10^2 \times g_{lum}$[g)]
**C_3-[8]HDI**	477	1200	495	5.8	6.96	8.33	1.35	762	2.56	90 / 420	5.6	6.0
**C_4-[8]HDI**	471	1200	487	5.7	6.52	8.74	1.45	698	2.60	110 / 418	5.1	5.3
**C_5-[8]HDI**	464	1600	474	5.7	6.11	9.33	1.54	455	2.65	147 / 412	4.0	4.0
**C_6-[8]HDI**	463	1600	472	6.5	6.20	10.5	1.51	412	2.66	170 / 412	3.5	3.3
***N*-Me-[8]HDI**	468	1400	480	4.3	5.72	7.52	1.67	534	2.63	155 / 420	3.1	3.4
***N*-*n*Bu-[8]HDI**	466	1300	479	5.6	5.97	9.38	1.58	582	2.63	151 / 417	3.1	–
***N*-*n*Bu-[8]HDI-*f*-OMe**	479	1000	529	5.8	6.15	9.43	1.53	1973	2.54	–	–	–
**[5]HDI-OMe**[1, 12]	440	–	472	22	3.21	68.5	2.43	1541	2.62	107 / 325	0.67	0.14
**[6]HDI-OMe**[1, 12]	460	–	470	2	2.39	8.36	4.10	463	2.66	146 / 319	1.8	0.70
**[7]HDI-OMe**[1, 12]	469	–	510	12	2.30	60.9	3.74	1714	2.49	150 / 405	1.1	0.11
**6**	448	300	456	1.6	6.02	2.66	1.63	392	2.76	207 / 381	3.3	–
**carbo-[8]helicene**[10-11]	~435	~250	~455	1.4	10.0	1.86	0.99	1010	~2.79	~255 / 375	3.4	–

[a)]Due to low extinction coefficients for the lowest-energy transitions, the wavelengths thereof were determined by looking for the first negative peak in the 2nd derivative of the respective absorption spectra. [b)]Extinction coefficient at the first observed absorption maximum, in M^{-1} cm^{-1}. [c)]Emission maximum upon excitation at

350 nm. [d)]$k_R = \Phi_{FL}\ \tau^{-1}$. [e)] $k_{NR} = (1 - \Phi_{FL})\ \tau^{-1}$. [f)]Optical energy gaps were calculated using the intersection of the normalized absorption and emission spectra. [g)]The maximum dissymmetry factors are given. The shown values are the average of the values found for both enantiomers. –: Not measured / not available in the literature.

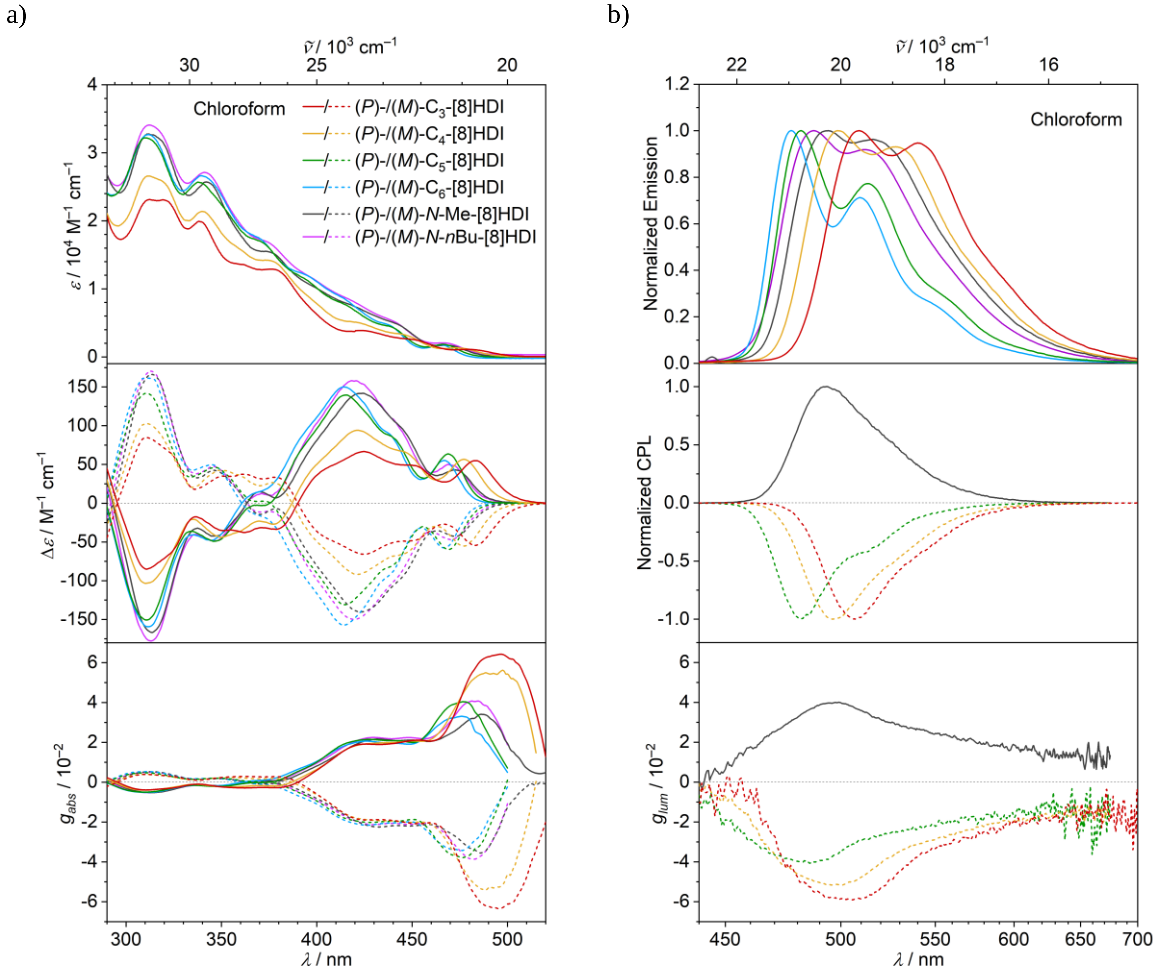

Figure S13. Comparison of (a) (top to bottom) absorption spectra ($c \sim 10^{-5}$ M), circular dichroism ($c \sim 10^{-5}$ M) and absorption dissymmetry factor, and (b) (top to bottom) emission spectra ($c \sim 10^{-6}$ M) , CPL spectra ($c \sim 10^{-5}$ M), and luminescence dissymmetry factor of the discussed **[8]HDI**s in chloroform. CPL was measured only for the (*M*) enantiomer for the **C*m*-[8]HDI**s and the (*P*) enantiomer for ***N*-Me-[8]HDI**.

Table S2. Summary of the optical and chiroptical properties of the discussed compounds in chloroform.

Compound	λ_{abs} / nm[a)]	ε [b)]	λ_{em} / nm [c)]	Φ_{FL} / %	t_{FL} / ns	k_{FL} / 10^6 s^{-1} [d)]	k_{NR} / 10^8 s^{-1} [e)]	Stokes shift / cm^{-1}	$E_{g(opt)}$ / eV [f)]	$\Delta\varepsilon$ / M^{-1} cm^{-1} / nm	$10^2 \times g_{abs}$[g)]	$10^2 \times g_{lum}$[g)]
**C$_3$-[8]HDI**	483	1050	509	6.1	6.07	10.1	1.55	1058	2.52	66 / 425	6.4	5.9
**C$_4$-[8]HDI**	476	1280	499	5.4	5.66	9.54	1.67	968	2.56	93 / 422	5.4	5.2
**C$_5$-[8]HDI**	468	1770	482	5.6	4.81	11.6	1.96	621	2.62	135 / 416	3.9	4.0
**C$_6$-[8]HDI**	467	1680	478	6.1	4.97	12.3	1.89	493	2.64	153 / 414	3.4	–
***N*-Me-[8]HDI**	472	1570	494	5.3	4.91	10.8	1.93	944	2.59	141 / 424	3.4	4.0
***N*-*n*Bu-[8]HDI**	470	1760	488	4.5	–	–	–	785	2.61	154 / 420	4.0	–

[a)]Due to low extinction coefficients for the lowest-energy transitions, the wavelengths thereof were determined by looking for the first negative peak in the 2nd derivative of the respective absorption spectra. [b)]Extinction coefficient at the first observed absorption maximum, in M^{-1} cm^{-1}. [c)]Emission maximum upon excitation at 350 nm. [d)]$k_R = \Phi_{FL}\ \tau^{-1}$. [e)] $k_{NR} = (1 - \Phi_{FL})\ \tau^{-1}$. [f)]Optical energy gaps were calculated using the intersection of the normalized absorption and emission spectra. [g)]The maximum dissymmetry factors are given. Where applicable, the shown values are the average of the values found for both enantiomers. –: Not measured.

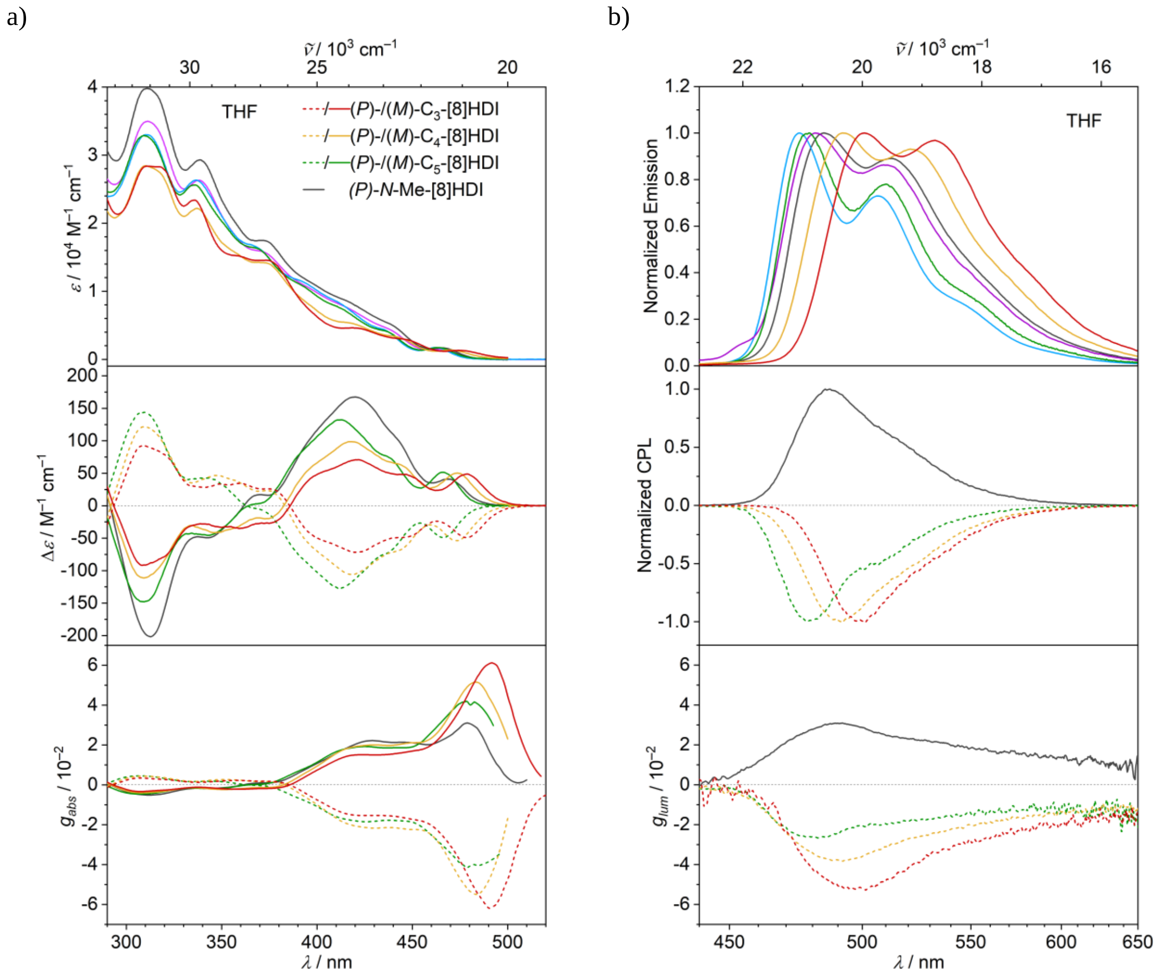

Figure S14. Comparison of (a) (top to bottom) absorption spectra ($c \sim 10^{-5}$ M), circular dichroism ($c \sim 10^{-5}$ M), and absorption dissymmetry factor, and (b) (top to bottom) emission spectra ($c \sim 10^{-6}$ M), CPL spectra ($c \sim 10^{-5}$ M) and luminescence dissymmetry factor of the discussed **[8]HDI**s in THF. CPL was measured only for the (*M*) enantiomer for the **C*m*-[8]HDI**s and the (*P*) enantiomer for ***N*-Me-[8]HDI**.

Table S3. Summary of optical and chiroptical properties of the discussed compounds in THF.

Compound	λ_{abs} / nm[a)]	ε [b)]	λ_{em} / nm [c)]	Φ_{FL} / %	t_{FL} / ns	k_{FL} / 10^6 s^{-1} [d)]	k_{NR} / 10^8 s^{-1} [e)]	Stokes shift / cm^{-1}	$E_{g(opt)}$ / eV [f)]	$\Delta\varepsilon$ / M^{-1} cm^{-1} / nm	$10^2 \times g_{abs}$ [g)]	$10^2 \times g_{lum}$ [g)]
**C₃-[8]HDI**	478	1200	500	4.3	7.01	6.13	1.37	921	2.55	71 / 421	6.2	5.2
**C₄-[8]HDI**	472	1300	493	5.3	6.51	8.14	1.45	902	2.59	102 / 419	5.3	3.8
**C₅-[8]HDI**	465	1700	479	5.3	5.86	9.04	1.62	629	2.64	130 / 413	4.1	2.6
**C₆-[8]HDI**	464	1700	475	5.5	6.03	9.12	1.57	499	2.65	–	–	–
***N*-Me-[8]HDI**	468	1600	485	4.5	5.58	8.06	1.71	749	2.62	167 / 420	3.1	3.1
***N*-*n*Bu-[8]HDI**	467	1300	481	4.5	–	–	–	623	2.63	–	–	–

[a)]Due to low extinction coefficients for the lowest-energy transitions, the wavelengths thereof were determined by looking for the first negative peak in the 2nd derivative of the respective absorption spectra. [b)]Extinction coefficient at the first observed absorption maximum, in M^{-1} cm^{-1}. [c)]Emission maximum upon excitation at 350 nm. [d)]$k_R = \Phi_{FL}\, \tau^{-1}$. [e)] $k_{NR} = (1 - \Phi_{FL})\, \tau^{-1}$. [f)]Optical energy gaps were calculated using the intersection of the normalized absorption and emission spectra. [g)]The maximum dissymmetry factors are given. Where applicable, the shown values are the average of the values found for both enantiomers. –: Not measured.

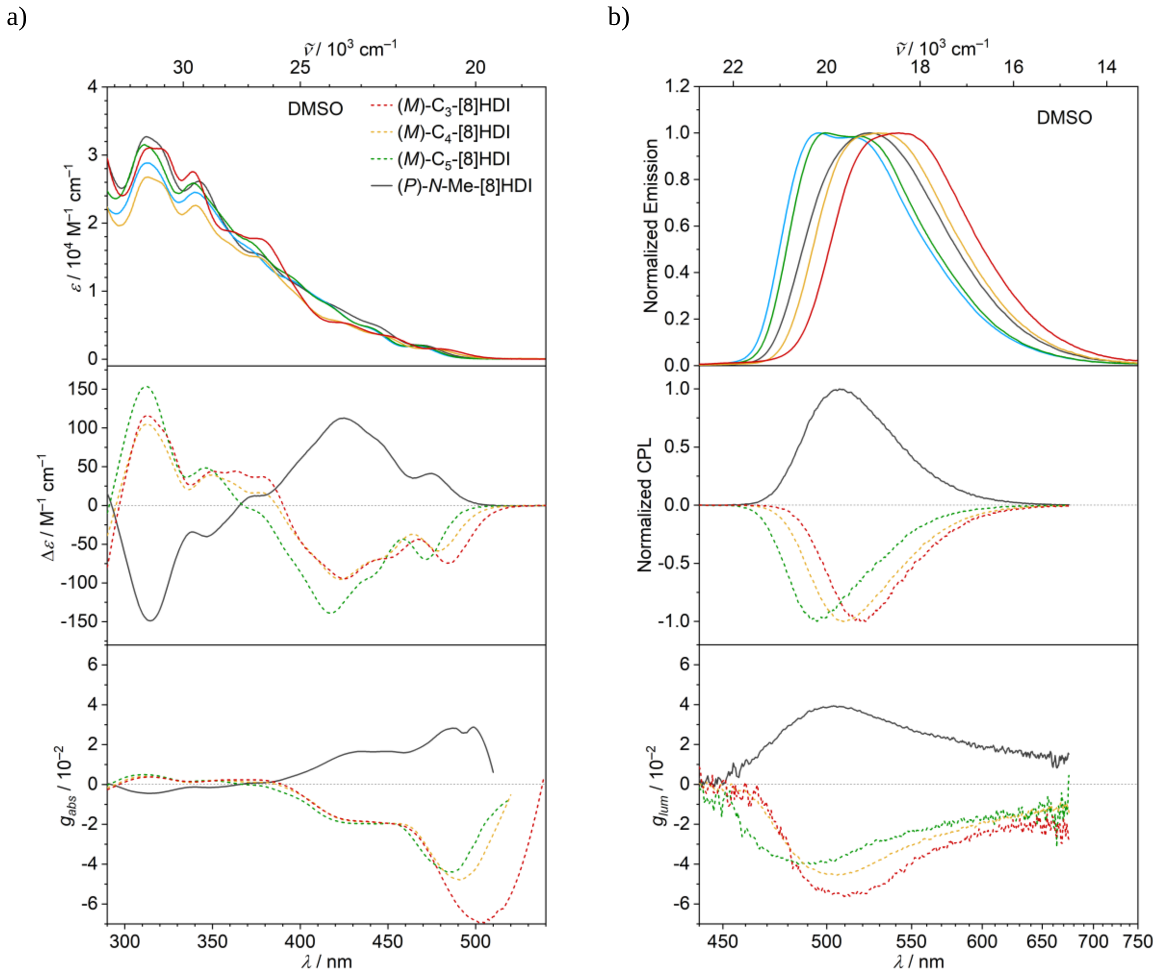

Figure S15. Comparison of (a) (top to bottom) absorption spectra ($c \sim 10^{-5}$ M), circular dichroism ($c \sim 10^{-5}$ M) and absorption dissymmetry factor and (b) (top to bottom) emission spectra ($c \sim 10^{-6}$ M), CPL spectra ($c \sim 10^{-5}$ M) and luminescence dissymmetry factor of the discussed **[8]HDI**s in DMSO. CD and CPL were measured only for the (*M*) enantiomer for the **C*m*-[8]HDI**s and the (*P*) enantiomer for ***N*-Me-[8]HDI**.

Table S4. Summary of optical and chiroptical properties of the discussed compounds in DMSO.

Compound	λ_{abs} / nm[a)]	ε [b)]	λ_{em} / nm [c)]	Φ_{FL} / %	t_{FL} / ns	k_{FL} / 10^6 s^{-1} [d)]	k_{NR} / 10^8 s^{-1} [e)]	Stokes shift / cm^{-1}	$E_{g(opt)}$ / eV [f)]	$\Delta\varepsilon$ / M^{-1} cm^{-1} / nm	$10^2 \times g_{abs}$[g)]	$10^2 \times g_{lum}$[g)]
**C_3-[8]HDI**	484	1400	542	5.3	5.96	8.89	1.59	2211	2.49	95 / 425	6.9	5.6
**C_4-[8]HDI**	477	1500	531	5.7	5.93	9.61	1.59	2132	2.53	95 / 423	4.8	4.5
**C_5-[8]HDI**	471	1900	500	5.3	5.25	10.10	1.80	1231	2.58	139 / 417	4.4	4.0
**C_6-[8]HDI**	469	1900	496	5.1	5.37	9.50	1.77	1161	2.60	–	–	–
***N*-Me-[8]HDI**	473	1900	524	5.6	5.27	10.63	1.79	2058	2.56	112 / 425	2.8	3.9

[a)]Due to low extinction coefficients for the lowest-energy transitions, the wavelengths thereof were determined by looking for the first negative peak in the 2nd derivative of the respective absorption spectra. [b)]Extinction coefficient at the first observed absorption maximum, in M^{-1} cm^{-1}. [c)]Emission maximum upon excitation at 350 nm. [d] $k_R = \Phi_{FL}\,\tau^{-1}$. [e)] $k_{NR} = (1 - \Phi_{FL})\,\tau^{-1}$. [f)]Optical energy gaps were calculated using the intersection of the normalized absorption and emission spectra. [g)]The maximum dissymmetry factors are given. –: Not measured.

S4. Electrochemistry

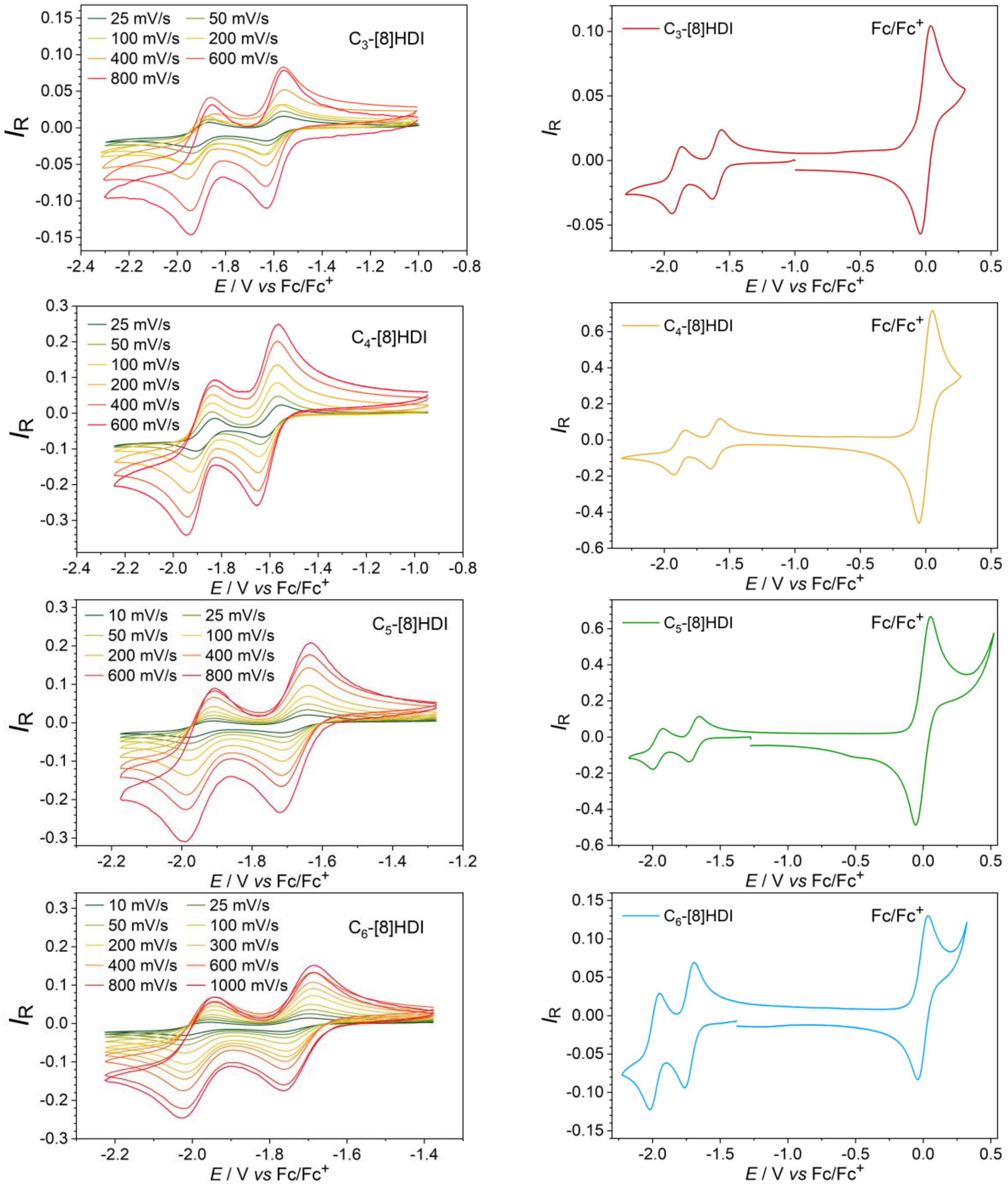

Figure S16. CV plots of the **C$_m$-[8]HDIs** in THF supported by a 0.2 M solution of $[Bu_4N][PF_6]$ as supporting electrolyte at various scan speeds (left) and the full CV spectrum including the Fc / Fc$^+$ reference at 200 mV s^{-1} (right).

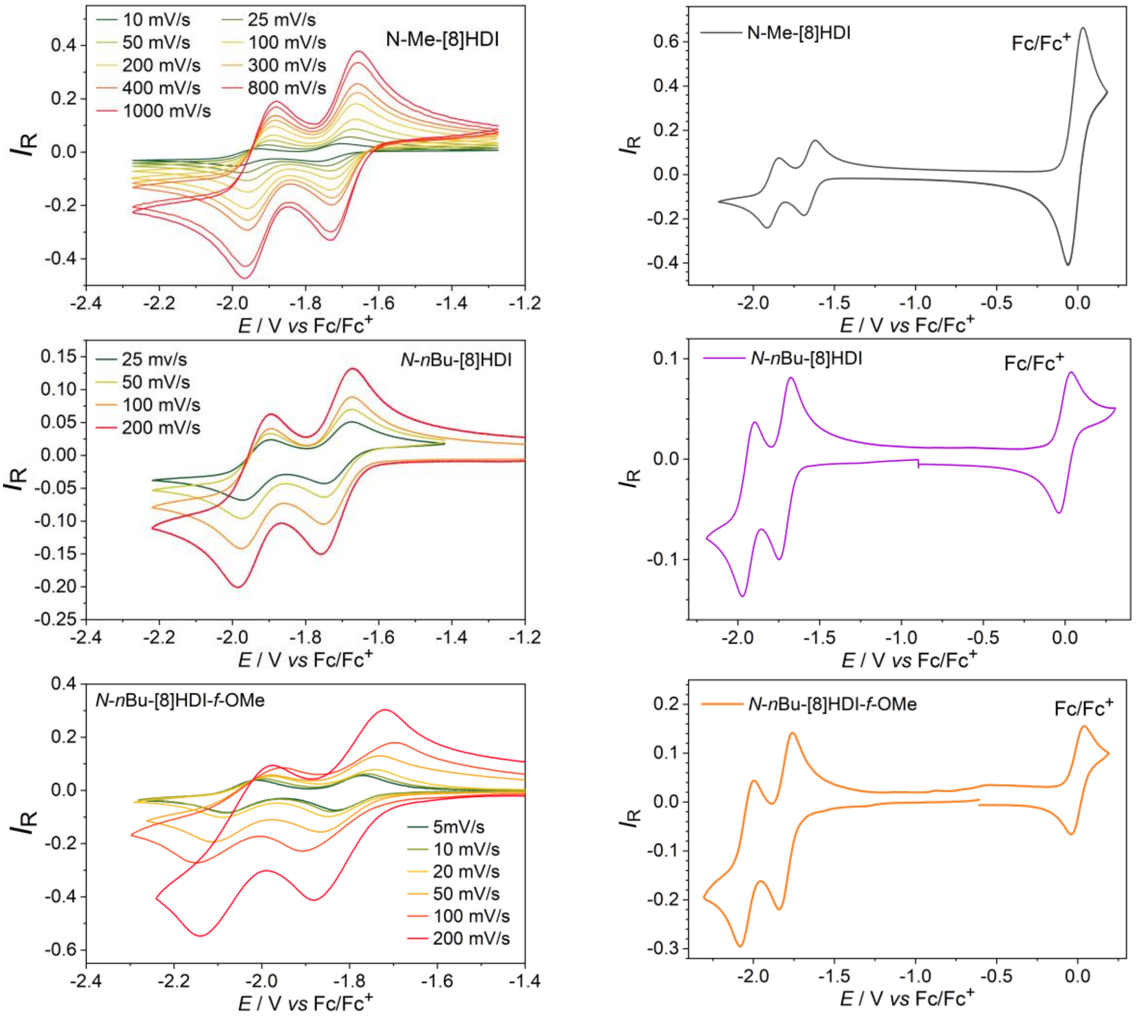

Figure S17. CV plots of ***N*-Me-[8]HDI**, ***N*-*n*Bu-[8]HDI**, and ***N*-*n*Bu-[8]HDI-*f*-OMe** in THF supported by a 0.2 M solution of $[Bu_4N][PF_6]$ as supporting electrolyte at various scan speeds (left) and the full CV plot including the Fc / Fc^+ reference at 200 mV s^{-1} (right).

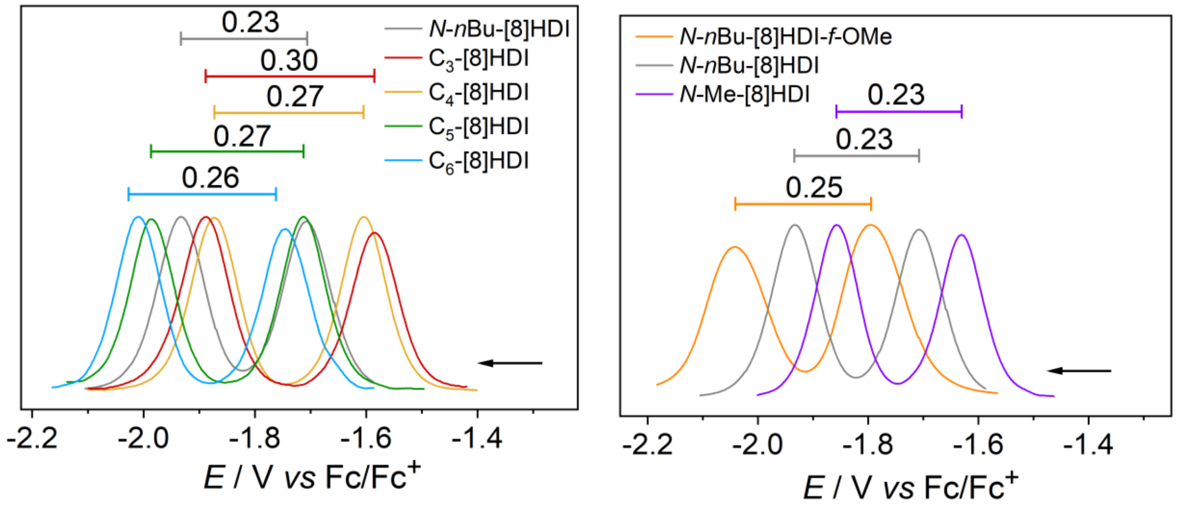

Figure S18. Comparison of the DPV plots of the **C_m-[8]HDI**s (left) and the open-chained **[8]HDI**s (right) with the difference between their reduction potentials given in V.

Table S5. Electrochemical properties of the **C$_m$-[8]HDI**s and the open-chained **[8]HDI**s in THF. a)LUMO = − (5.1 + $E_{red}1$) eV.[13] b)HOMO = LUMO − $E_{g\ (opt)}$, see Tab. S1

	$E_{red}1$ / V	$E_{red}2$ / V	ΔE_{red} / V	LUMO / eV[a)]	HOMO / eV[b)]
**C$_3$-[8]HDI**	−1.585	−1.887	0.302	−3.52	−6.06
**C$_4$-[8]HDI**	−1.605	−1.873	0.268	−3.50	−6.08
**C$_5$-[8]HDI**	−1.712	−1.986	0.274	−3.39	−6.03
**C$_6$-[8]HDI**	−1.744	−2.008	0.264	−3.36	−6.00
***N*-Me-[8]HDI**	−1.630	−1.857	0.227	−3.47	−6.09
***N*-nBu-[8]HDI**	−1.707	−1.933	0.226	−3.39	−6.03
***N*-*n*Bu-[8]HDI-*f*-OMe**	−1.796	−2.041	0.245	−3.30	−5.91

S5. Chiral Stationary Phase HPLC

Table S6. Overview of conditions for the HPLC separation of the enantiomers of the discussed compounds.

Compound	column^a	Eluent *n*-hex./CHCl$_3$/*i*PrOH	First fraction	Second fraction	α^b	R_s^c	*ee*
**6**	Lux i-Amylose-3	48/50/2	(*P*)	(*M*)	1.23	6.33	>99%
**C$_3$-[8]HDI**	Lux i-Amylose-3	25/72/3	(*M*)	(*P*)	1.29	9.48	>99%
**C$_4$-[8]HDI**	Lux i-Amylose-3	25/72/3	(*M*)	(*P*)	1.23	8.06	>99%
**C$_5$-[8]HDI**	Lux i-Amylose-3	25/72/3	(*M*)	(*P*)	1.22	7.66	>99%
**C$_6$-[8]HDI**	Lux i-Amylose-3	25/72/3	(*M*)	(*P*)	1.08	3.00	>99%
***N*-Me-[8]HDI**	Lux i-Amylose-3	25/72/3	(*M*)	(*P*)	1.23	8.68	>99%
***N*-*n*Bu-[8]HDI**	Lux i-Amylose-3	48/50/2	(*P*)	(*M*)	1.04	1.64	>99%

^{a}Phenomenex Lux i-Amylose-3 5 µm (250 × 4.6 mm). Sample injection: 10 µL of a 0.2 mg mL^{-1} solution in $CHCl_3$. Separation conditions: Analytical column, eluent flow rate: 0.5 mL min^{-1}, 20 °C. ^{b}Selectivity parameter: $\alpha = t_{R2} / t_{R1}$, where t_{R1}, and t_{R2} are elution times for first fraction, and second fraction, respectively. ^{c}Resolution parameter: $R_S = 2(t_{R2} - t_{R1}) / (w_2 + w_1)$, where w_1 and w_2 are half-height peak widths for first and second fraction, respectively.

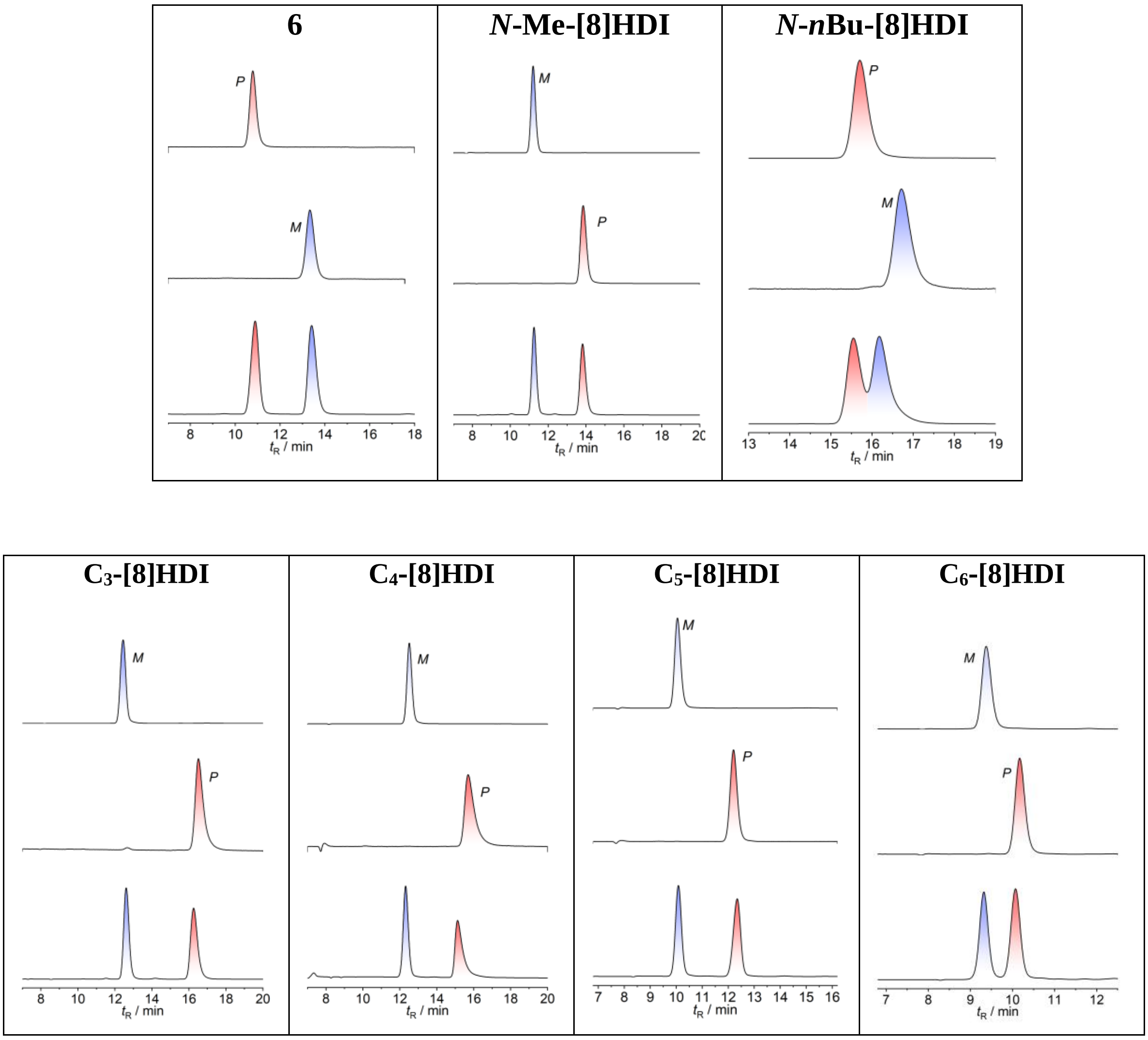

Figure S19. Analytical HPLC chromatograms of the racemic and enantiopure samples of **6, *N*-Me-[8]HDI,** and ***N*-*n*Bu-[8]HDI** (top), and **C*m*-[8]HDI** (bottom) using a chiral stationary phase column (Lux i-Amylose-3 5 µm 250 × 4.6 mm). The chromatogram detector was set at 350 nm with a bandwidth of 4 nm. Conditions of the separation: See Table S6. Larger amounts of **6** were separated on a semipreparative column of the same composition (250 × 21.2 mm). The enantiomeric purity of the final compounds that had been synthesized from enantiomerically pure **6** was checked in the same manner and the *ee* in all cases was >99%.

S6. Quantum Chemical Calculations

DFT calculations were performed using Gaussian 16 suite.[14] Geometries in ground and first excited state were optimized using the ωB97XD functional and 6-31g(d,p) basis set in the gas phase. All calculations are performed on global minimum conformations, obtained with the software package CREST.[15-16] TD–DFT calculations were performed on ωB97XD/6-31g(d,p) optimized geometries at the B3LYP/6-311G(2d,p)level. The effect of the solvent was accounted for using PCM (with toluene as the solvent). SpecDis and Avogadro software were used to analyze the TD–DFT calculated spectra, and POV–Ray was used to render graphical images of frontier molecular orbitals (FMOs).

Table S7. Calculated energies (in eV) of the frontier molecular orbitals of the discussed compounds.

Compound	HOMO−2	HOMO−1	HOMO	LUMO	LUMO+1	LUMO+2
**C_3-[8]HDI**	−8.176	−7.812	−7.683	−0.972	−0.671	−0.188
**C_4-[8]HDI**	−8.879	−8.354	−8.154	−0.925	−0.717	−0.218
**C_5-[8]HDI**	−8.115	−7.804	−7.738	−0.889	−0.756	−0.240
**C_6-[8]HDI**	−8.146	−7.818	−7.778	−0.875	−0.728	−0.259
***N*-Me-[8]HDI**	−8.152	−7.776	−7.748	−0.814	−0.812	−0.233
***N*-*n*Bu-[8]HDI**	−8.156	−7.784	−7.745	−0.835	−0.815	−0.241
**6**	−7.747	−7.393	−7.385	−0.216	−0.143	0.388

Table S8.1. TD–DFT (B3LYP/6-311G(2d,p)) calculated parameters defining g_{abs} for the $S_0 \rightarrow S_1$ transition of the discussed compounds in toluene.

Compound	λ [a]	f [b]	$\lvert\mu_e\rvert$ [c]	$\lvert\mu_m\rvert$ [d]	$\cos\theta$ [e]	R [f]	$10^2 \times g_{abs}$ [g] (calc.)	$10^2 \times g_{abs}$ [g] (obs.)
**C_3-[8]HDI**	476.6	0.0149	123.06	2.180	0.942	252.85	6.68	5.6
**C_4-[8]HDI**	467.1	0.0177	132.55	2.075	0.976	268.32	6.11	5.1
**C_5-[8]HDI**	461.1	0.0183	134.18	1.953	0.990	259.41	5.76	4.0
**C_6-[8]HDI**	452.2	0.0287	166.24	1.900	0.983	310.88	4.50	3.5
***N*-Me-[8]HDI**	453.9	0.0146	118.80	1.403	0.986	164.45	4.66	3.1
***N*-*n*Bu-[8]HDI**	456.0	0.0161	124.94	1.557	1.00	194.52	4.98	3.1
**6 (first transition)**	424.6	0.0021	43.61	0.122	0.155	−0.826	0.17	–
**6 (second transition)**	410.7	0.0648	238.07	3.499	0.686	571.58	4.03	3.4

[a]Wavelength in nanometers. [b]Oscillator strength in atomic units. [c]Electric transition dipole moments for the $S_0 \rightarrow S_1$ transition in 10^{-20} esu cm. [d]Magnetic transition dipole moments for the $S_0 \rightarrow S_1$ transition in 10^{-20} erg G^{-1}. [e]Angle between μ_e and μ_m in degrees. [f]Rotational strength in 10^{-40} erg · esu · cm · G^{-1}. [g]Dimensionless values.

Table S8.2. TD–DFT (B3LYP/6-311G(2d,p)) calculated parameters defining g_{lum} for the $S_1 \rightarrow S_0$ transition of the discussed compounds in toluene.

Compound	$\lvert\mu_e\rvert$ [a]	$\lvert\mu_m\rvert$ [b]	cos θ [c]	R [d]	$10^2 \times g_{\text{lum}}$ [e] (calc.)	$10^2 \times g_{\text{lum}}$ [e] (obs.)
**C3-[8]HDI**	221.85	2.459	0.533	290.97	2.36	6.0
**C3-[8]HDI**[f]	97.04	2.107	0.757	154.69	6.57	6.0
**C4-[8]HDI**	135.78	2.138	0.938	272.13	5.90	5.3
**C5-[8]HDI**	140.14	2.042	0.961	274.85	5.60	4.0
**C6-[8]HDI**	174.79	1.953	0.937	319.94	4.19	3.3
***N*-Me-[8]HDI**	125.28	1.632	0.997	203.96	5.20	3.4
***N*-*n*Bu-[8]HDI**	137.88	1.897	0.968	253.15	5.33	–

[a]Electric transition dipole moments for the $S_1 \rightarrow S_0$ transition in 10^{-20} esu cm. [b]Magnetic transition dipole moments for the $S_1 \rightarrow S_0$ transition in 10^{-20} erg G^{-1}. [c]Angle between μ_e and μ_m in degrees. [d] Rotational strength in 10^{-40} erg · esu · cm · G^{-1}. [e]Dimensionless values. [f]The geometry of the alkyl bridge of **C3-[8]HDI** was restrained in the C_2-symmetric state for the calculations.

Interestingly, with the exception of **C3-[8]HDI**, all molecules exhibit a consistent trend in the excited state $S_1 \rightarrow S_0$ transition in terms of μ_m, μ_e, and cos θ, mirroring the behavior observed in the ground-state $S_0 \rightarrow S_1$ transition. Furthermore, the calculated g_{lum} values for these molecules are in good agreement with the experimental measurements.

The deviation observed for **C3-[8]HDI** is likely attributable to limitations of DFT in accurately capturing the charge-transfer character between closely positioned naphthalene imide moieties in the excited state, or to symmetry-related factors. We tested several DFT ansatzes including different functionals and basis sets as well as the solvent effects which yielded the same trend. To evaluate the influence of molecular symmetry, we optimized the excited-state geometry of a C_2-symmetric conformer of **C3-[8]HDI**, which yielded a calculated g_{lum} value closely matching the experimental result. It is worth noting that the C_2-symmetric conformer lies much higher in energy relative to the lowest-energy conformer. We acknowledge that a more accurate description might be achievable through high-level methods such as coupled-cluster calculations; however, such investigations are beyond the scope of the present study.

Compound	**HOMO**	**LUMO**
**C_3-[8]HDI**	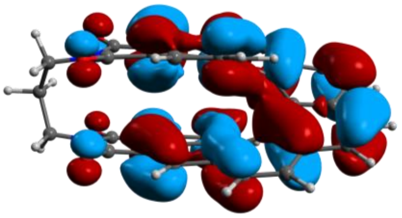	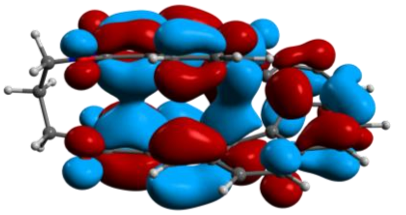
**C_4-[8]HDI**	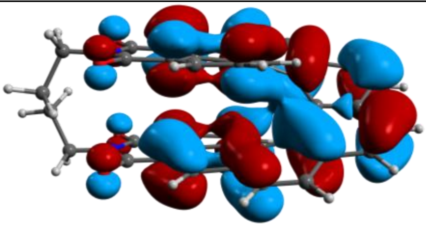	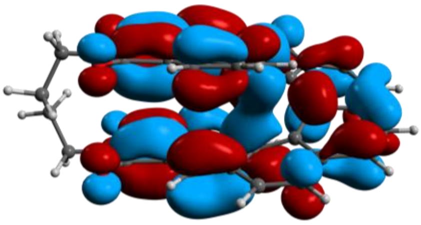
**C_5-[8]HDI**	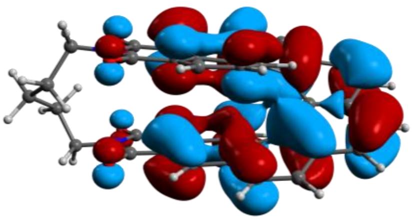	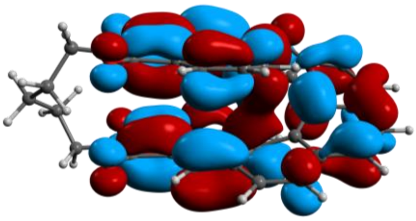
**C_6-[8]HDI**	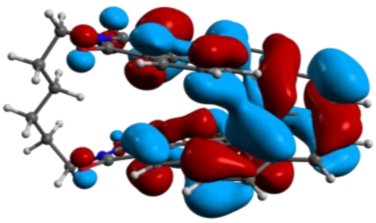	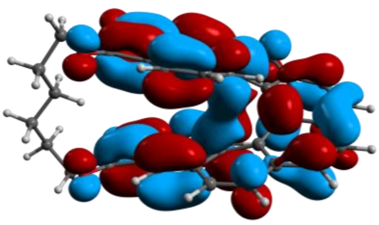
***N*-*n*Bu-[8]HDI**	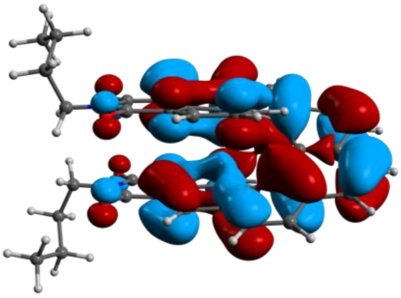	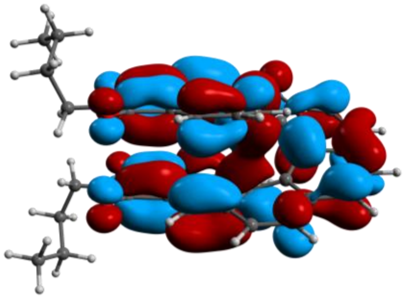
***N*-Me-[8]HDI**	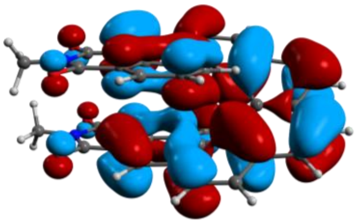	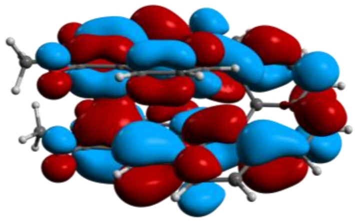
**6**	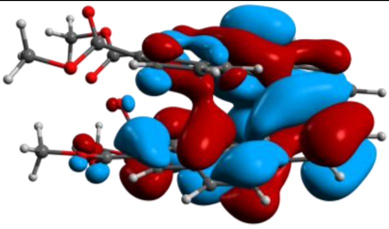	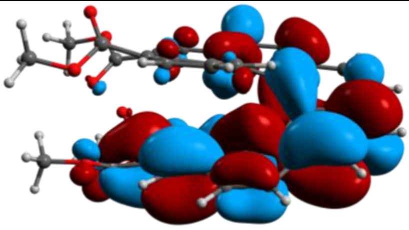

Figure S20. Frontier molecular orbitals of the discussed compounds.

Table S9. Summary of TD–DFT calculated key low-energy transitions.

Excited singlet state	Wavelength	Energy	Major transitions	Contribution	Oscillator strength (*f*)
**C_3-[8]HDI**					
1	476.57 nm	2.60 eV	HOMO → LUMO	0.69	0.0149
			HOMO−2 → LUMO	−0.11	
2	449.68 nm	2.76 eV	HOMO−1 → LUMO	0.68	0.0038
			HOMO → LUMO+1	0.11	
			HOMO → LUMO+2	−0.12	
**C_4-[8]HDI**					
1	467.06 nm	2.65 eV	HOMO → LUMO	0.69	0.0177
			HOMO−2 → LUMO	−0.12	
2	443.00 nm	2.80 eV	HOMO−1 → LUMO	0.68	0.0062
			HOMO → LUMO+1	−0.11	
			HOMO → LUMO+2	−0.13	
**C_5-[8]HDI**					
1	461.08 nm	2.69 eV	HOMO → LUMO	0.68	0.0183
			HOMO−1 → LUMO+1	−0.10	
			HOMO−2 → LUMO	−0.12	
2	438.39 nm	2.82 eV	HOMO−1 → LUMO	0.68	0.0089
			HOMO → LUMO+1	0.12	
			HOMO → LUMO+2	0.13	
**C_6-[8]HDI**					
1	452.24 nm	2.74 eV	HOMO → LUMO	0.40	0.0287
			HOMO−1 → LUMO	0.55	
			HOMO−2 → LUMO	0.12	
2	433.06 nm	2.86 eV	HOMO−1 → LUMO	−0.39	0.0096
			HOMO−1 → LUMO+2	0.10	
			HOMO → LUMO	0.55	
***N*-Me-[8]HDI**					
1	453.90 nm	2.73 eV	HOMO−1 → LUMO	0.65	0.0146
			HOMO → LUMO+1	−0.22	
2	438.56 nm	2.82 eV	HOMO−1 → LUMO	0.23	0.0322
			HOMO → LUMO+1	0.65	
			HOMO → LUMO+2	0.13	
***N*-*n*Bu-[8]HDI**					
1	456.04 nm	2.72 eV	HOMO−1 → LUMO	0.67	0.0161
			HOMO → LUMO+1	−0.19	
2	437.78 nm	2.83 eV	HOMO−1 → LUMO	0.19	0.0342
			HOMO → LUMO+1	0.65	
			HOMO → LUMO+2	0.14	
**6**					
1	424.62 nm	2.92 nm	HOMO−1 → LUMO	0.43	0.0021
			HOMO−1 → LUMO+1	−0.22	
			HOMO → LUMO	0.29	
			HOMO → LUMO+1	0.40	
2	410.73 nm	3.02 eV	HOMO−1 → LUMO	−0.45	0.0648
			HOMO−1 → LUMO+1	0.11	
			HOMO → LUMO	0.48	
			HOMO → LUMO+1	0.20	

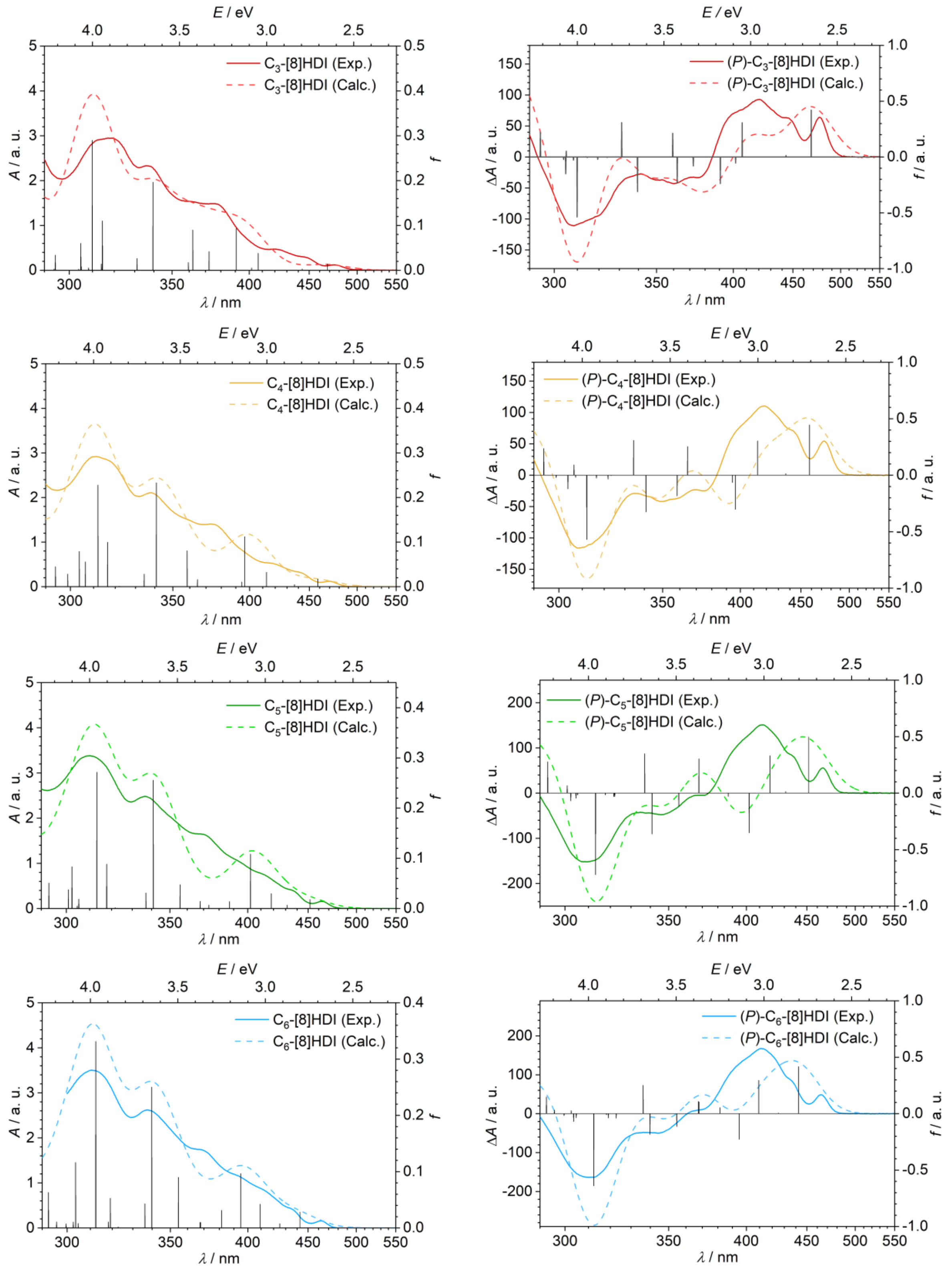

Figure S21. Comparison of experimental (solid line), TD–DFT calculated curve (dotted line), and bar (grey bar) UV–Vis spectra (left) and ECD spectra (right) of **C3-[8]HDI**, **C4-[8]HDI**, **C5-[8]HDI**, and **C6-[8]HDI** in toluene (the calculated spectra are blue shifted by 15 nm).

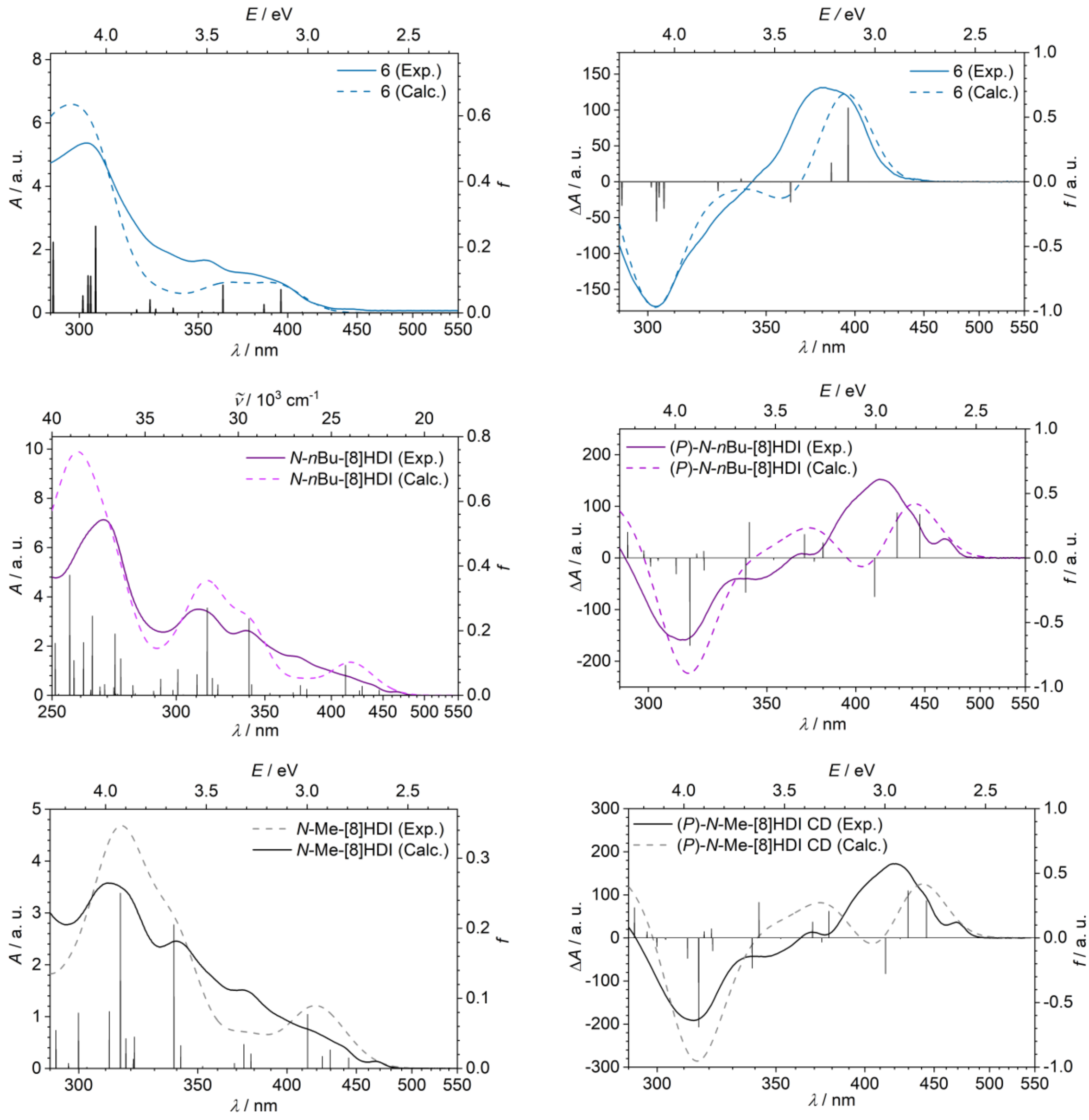

Figure S22. Comparison of experimental (solid line), TD–DFT calculated curve (dotted line), and bar (grey bar) UV–Vis spectra (left) and ECD spectra (right) of **6**, ***N*-*n*Bu-[8]HDI** and ***N*-*n*Bu-[8]HDI** in toluene (the calculated spectra are blue shifted by 15 nm).

Radical anion calculations

Table S10. IV–CT parameters calculated for the radical anions in THF using exact 35% HF-exchange contribution.[17-18]

Compound	*P* (neutral) / Å	*P* (radical anion) / Å	E_{ab} / cm^{-1}	$\Delta\mu_{ab}$ / D	μ_{ab} / D	V_{12} / cm^{-1}	ΔG_{ab} / cm^{-1}
**C_3-[8]HDI** (−1)	3.345	3.286	4899.9	5.48	3.38	1902	61
**C_4-[8]HDI** (−1)	3.406	3.359	4192.7	7.14	3.46	1458	97
**C_5-[8]HDI** (−1)	3.447	3.407	3703.1	8.98	2.82	986	202
**C_6-[8]HDI** (−1)	3.793	3.842	3608.4	11.1	3.31	923	215
***N*-Me-[8]HDI** (−1)	3.461	3.407	3451.6	9.83	2.03	658	330
***N*-Bu-[8]HDI** (−1)	–	–	3559.4	9.80	2.27	749	298

$\Delta\mu_{ab}$: Dipole moment difference between ground sate and IV–CT state, μ_{ab}: Projection of the transition moment on the dipole moment difference vector $\Delta\mu_{ab}$. V_{12}: Electronic coupling between the redox centers.

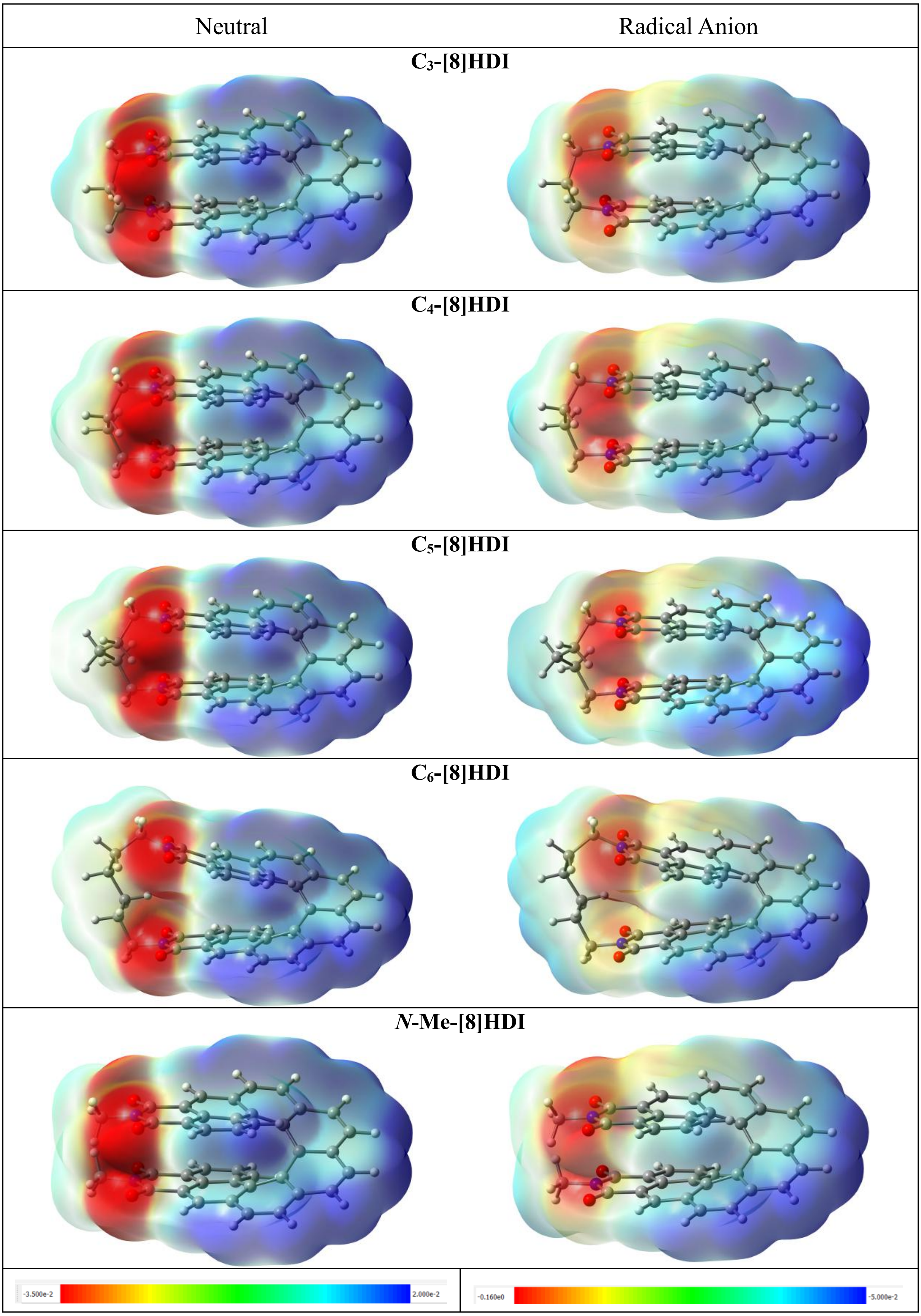

Figure S23. Calculated molecular electrostatic potential surfaces (MEPs) in the neutral molecule (left) and in the radical anion (right) for the discussed compounds. Note that the scaling of the color map is different between the left and right columns.

S7. Single Crystal Data

The crystal data of the compounds were collected on a RIGAKU XTALAB SYNERGY-R diffractometer with a HPA area detector and multi-layer mirror monochromated $Cu_{K\alpha}$ radiation. The structure was solved using intrinsic phasing method,[19] refined with the SHELXL program,[20] and expanded using Fourier techniques. All non-hydrogen atoms were refined anisotropically. ORTEP plots were calculated and rendered with the ORTEP–III (1.0.3) software.[21]

C3-[8]HDI

Single crystals of **(*M*)-C3-[8]HDI** were grown by slow diffusion of methanol into a saturated solution of the compound in chloroform in an NMR tube at room temperature. The measured crystal was obtained as a yellow block. The solvent molecule (chloroform) was disordered. The respective C–Cl and Cl–Cl distances were restrained during refinement to the same value to keep C_{3v} symmetry. The isotropic atomic displacement parameters of all atoms in both fragments were refined using a common free variable.

Single crystals of ***rac*-C3-[8]HDI** were grown by slow diffusion of methanol into a saturated solution of the compound in chloroform in an NMR tube at room temperature. The measured crystal was obtained as a clear, light-yellow plate. The propyl bridge was showing disorder (53:47%). The distances between α- and γ-carbon atoms in chains to the respective nitrogen atoms in helicene were restrained with SADI command. The atomic displacement parameters of disordered atoms were restrained with RIGU keyword ('enhanced rigid bond' restraint for all bonds in the connectivity list), a similarity restraint SIMU and their U_{ii} displacement parameters were restrained with ISOR keyword to approximate isotropic behavior. One reflection (0 3 5) was removed from the refinement as an outlier.

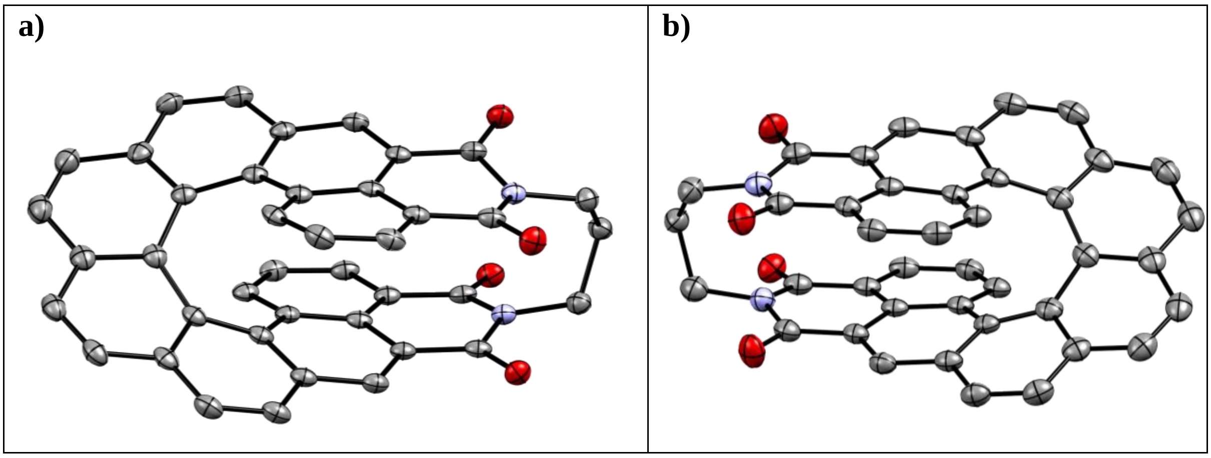

Figure S24. Thermal ellipsoids of (a) **(*M*)-C3-[8]HDI** and (b) ***rac*-C3-[8]HDI** at the 50% probability level. The hydrogen atoms are omitted for clarity.

Table S11. Crystal data and structure refinement for **C_3-[8]HDI**.

Compound	**(*M*)-C_3-[8]HDI**	***rac*-C_3-[8]HDI**
CCDC number	2434330	2434331
Chemical formula	$2(C_{41}H_{22}N_2O_4)\cdot CHCl_3$	$C_{41}H_{22}N_2O_4\cdot CHCl_3$
M_r	1332.58	725.97
Crystal system, space group	Tetragonal, $P4_12_12$	Triclinic, P_1
Temperature / K	100	100
a, b, c / Å	15.180 (14), 15.180(14), 25.88 (2)	6.992 (13), 15.45 (3), 15.82 (4)
α, β, γ / °	90, 90, 90	110.81 (12), 97.46 (12), 100.95 (12)
V / Å^3	5964 (12)	1532 (6)
Z	4	2
Radiation type	Cu $K\alpha$	Cu $K\alpha$
μ / mm^{-1}	1.97	3.14
Crystal size / mm	0.21 × 0.14 × 0.12	0.32 × 0.10 × 0.02
T_{min}, T_{max}	0.721, 1.000	0.345, 1.000
No. of measured, independent and observed [$I > 2\sigma(I)$] reflections	60515, 5765, 5639	26494, 5815, 4733
R_{int}	0.028	0.049
(sin θ / λ) max / Å^{-1}	0.617	0.620
$R[F^2 > 2\sigma(F^2)]$, $wR(F^2)$, S	0.043, 0.112, 1.03	0.060, 0.181, 1.10
No. of reflections	5765	5815
No. of parameters	450	488
No. of restraints	30	126
Flack parameter	0.019 (5)	–
$\Delta\rho_{max}$, $\Delta\rho_{min}$ / e Å^{-3}	0.70, −0.50	0.52, −0.62

C_4-[8]HDI

Single crystals of **(*M*)-C_4-[8]HDI** were grown by slow diffusion of methanol into a saturated solution of the compound in chloroform in an NMR tube at room temperature. The measured crystal was obtained as a clear, light-yellow needle. The butyl bridge was showing disorder (73:27%). The geometry of both chains was restrained (SADI and SAME commands). The atomic displacement parameters of the disordered atoms were restrained with RIGU keyword in ShelXL input ('enhanced rigid bond' restraint for all bonds in the connectivity list), similarity restraint SIMU and their U_{ii} displacement parameters were restrained with ISOR keyword to approximate isotropic behavior. The solvent (MeOH) showed disorder (78:22%). The C=O distance for both molecules was restrained with SADI command. Isotropic refinement was done using a single free-variable for all four U_{iso} parameters.

Single crystals of ***rac*-C_4-[8]HDI** were grown by slow diffusion of methanol into a saturated solution of the compound in chloroform in an NMR tube at room temperature. The measured crystal was obtained as a clear, light-yellow plate and refined as a 2-component twin. Component 2 was rotated by −180° around the [1 0 0] (direct) axis. The BASF parameter was refined to 46.9%. The butyl bridge was showing disorder (80:20%). The atomic displacement parameters of disordered atoms were restrained with RIGU keyword in ShelXL input ('enhanced rigid bond' restraint for all bonds in the connectivity list), similarity restraint SIMU and their U_{ii} displacement parameters were restrained with ISOR keyword to approximate isotropic behavior.

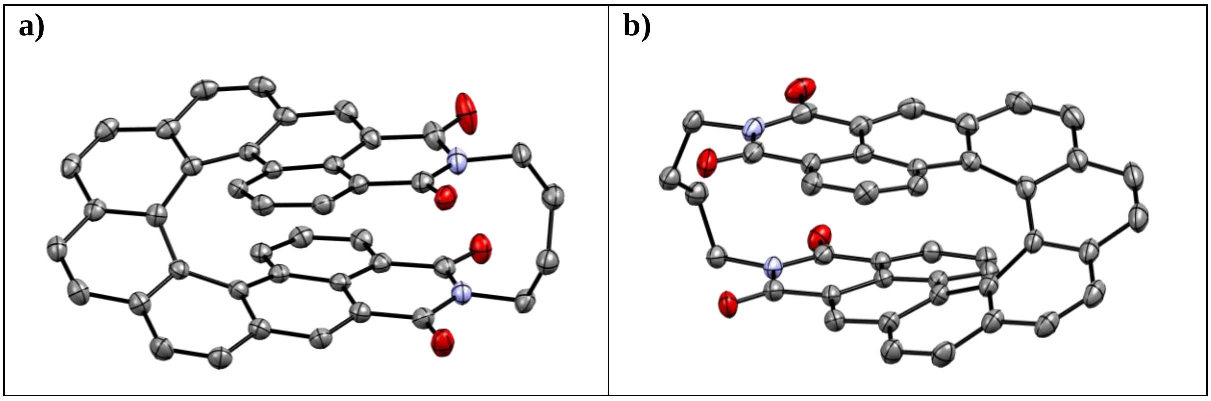

Figure S25. Thermal ellipsoids of (a) **(*M*)-C_4-[8]HDI** and (b) ***rac*-C_4-[8]HDI** at the 50% probability level. The hydrogen atoms are omitted for clarity.

Table S12. Crystal data and structure refinement for **C4-[8]HDI**.

Compound	**(*M*)-C4-[8]HDI**	***rac*-C4-[8]HDI**
CCDC number	2434332	2434333
Chemical formula	$C_{42}H_{24}N_2O_4 \cdot CH_4O$	$C_{42}H_{24}N_2O_4 \cdot CHCl_3$
M_r	652.67	740.00
Crystal system, space group	Orthorhombic, $P2_12_12_1$	Triclinic, P_1
Temperature / K	100	100
a, *b*, *c* / Å	7.287 (10), 17.07 (2), 24.07 (3)	7.209 (8), 15.649 (19), 15.83 (2)
α, β, γ / °	90, 90, 90	110.60 (8), 98.76 (9), 102.21 (7)
V / Å^3	2994 (7)	1583 (4)
Z	4	2
Radiation type	Cu *K*α	Cu *K*α
μ / mm^{-1}	0.77	3.05
Crystal size / mm	0.37 × 0.07 × 0.02	0.40 × 0.07 × 0.03
T_{min}, T_{max}	0.573, 1.000	0.604, 1.000
No. of measured, independent and observed [$I > 2\sigma(I)$] reflections	27929, 5633, 5244	12402, 12402, 11111
R_{int}	0.039	0.064
(sin θ / λ) max / Å^{-1}	0.614	0.620
$R[F^2 > 2\sigma(F^2)]$, $wR(F^2)$, S	0.046, 0.129, 0.91	0.069, 0.201, 1.02
No. of reflections	5633	12402
No. of parameters	485	507
No. of restraints	194	174
Flack parameter	−0.06 (13)	–
$\Delta\rho_{max}$, $\Delta\rho_{min}$ / e Å^{-3}	0.22, −0.24	0.73, −0.61

C_5-[8]HDI

Single crystals of ***rac*-C_5-[8]HDI** were grown by slow evaporation of a saturated solution of the compound in chloroform in an NMR tube at room temperature. The measured crystal was obtained as a yellow plate.

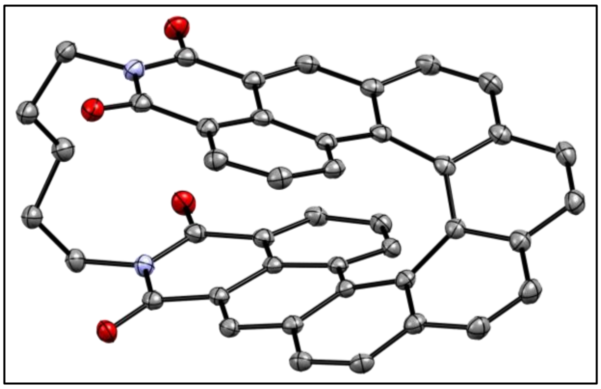

Figure S26. Thermal ellipsoids of ***rac*-C_5-[8]HDI** at the 50% probability level. The hydrogen atoms are omitted for clarity.

C_6-[8]HDI

Single crystals of ***rac*-C_6-[8]HDI** were grown by slow evaporation of a saturated solution of the compound in chloroform in an NMR tube at room temperature. The measured crystal was obtained as a clear, light-yellow needle. One of the chloroform molecules was disordered. The atomic displacement parameters of all atoms in both parts were restrained with similarity restraint SIMU, RIGU keyword in ShelXL input ('enhanced rigid bond' restraint for all bonds in the connectivity list) and their U_{ii} displacement parameters were restrained with ISOR keyword to approximate isotropic behavior. The C–Cl and Cl–Cl distances were restrained during refinement to the same value.

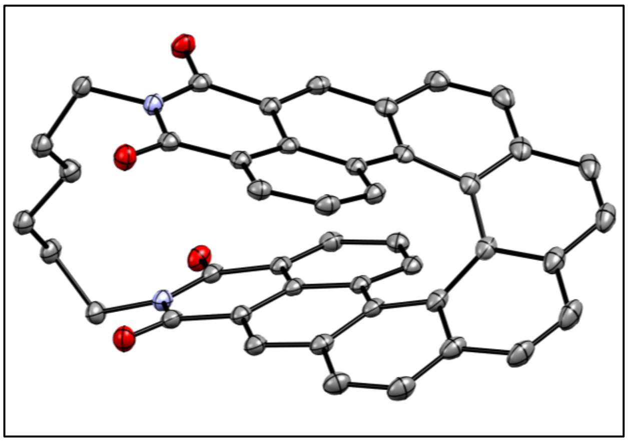

Figure S27. Thermal ellipsoids of ***rac*-C_6-[8]HDI** at the 50% probability level. The hydrogen atoms are omitted for clarity.

Table S13. Crystal data and structure refinement for **C_5-[8]HDI**.

Compound	***rac*-C_5-[8]HDI**
CCDC number	2434334
Chemical formula	$C_{40}H_{22}N_2O_4$
M_r	594.59
Crystal system, space group	Triclinic, P_1
Temperature / K	102
a, b, c / Å	7.77184 (11), 11.1036 (2), 15.4399 (3)
α, β, γ / °	82.2171 (15), 80.0312 (13), 86.2993 (13)
V / Å^3	1299.03 (4)
Z	2
Radiation type	Cu $K\alpha$
μ / mm^{-1}	0.80
Crystal size / mm	0.27 × 0.21 × 0.15
T_{min}, T_{max}	0.600, 1.000
No. of measured, independent and observed [$I > 2\sigma(I)$] reflections	24951, 5118, 4918
R_{int}	0.013
(sin θ / λ) max / Å^{-1}	0.627
$R[F^2 > 2\sigma(F^2)]$, $wR(F^2)$, S	0.041, 0.118, 1.09
No. of reflections	5118
No. of parameters	417
No. of restraints	0
Flack parameter	–
$\Delta\rho_{max}$, $\Delta\rho_{min}$ / e Å^{-3}	0.23, −0.21

Table S14. Crystal data and structure refinement for **C_6-[8]HDI**.

Compound	***rac*-C_6-[8]HDI**
CCDC number	2434335
Chemical formula	$C_{44}H_{28}N_2O_4 \cdot 2(CHCl_3)$
M_r	887.42
Crystal system, space group	Monoclinic, $P2_1/n$
Temperature / K	100
a, b, c / Å	14.804 (16), 14.132 (15), 19.56 (3)
α, β, γ / °	90, 109.70 (2), 90
V / Å^3	3853 (8)
Z	4
Radiation type	Cu $K\alpha$
μ / mm^{-1}	4.48
Crystal size / mm	0.31 × 0.07 × 0.05
T_{min}, T_{max}	0.384, 1.000
No. of measured, independent and observed [$I > 2\sigma(I)$] reflections	37435, 7418, 6793
R_{int}	0.020
(sin θ / λ) max / Å^{-1}	0.620
$R[F^2 > 2\sigma(F^2)]$, $wR(F^2)$, S	0.046, 0.118, 1.02
No. of reflections	7418
No. of parameters	560
No. of restraints	219
Flack parameter	–
$\Delta\rho_{max}$, $\Delta\rho_{min}$ / e Å^{-3}	1.02, −0.72

_N_-Me-[8]HDI

Single crystals of **(_P_)-_N_-Me-[8]HDI** were grown by slow diffusion of methanol into a saturated solution of the compound in chloroform in an NMR tube at room temperature. The measured crystal was obtained as an orange block.

Single crystals of **_rac_-_N_-Me-[8]HDI** were grown by slow diffusion of methanol into a saturated solution of the compound in DCM in an NMR tube at room temperature. These crystals could be assigned to two different polymorphs, and both structures were determined separately.

The measured crystal of polymorph 1 was obtained as an orange block.

The measured crystal of polymorph 2 was obtained as a yellow plate. The unit cell contains solvent molecules (mixture of DCM and methanol) which have been treated as a diffuse contribution to the overall scattering without specific atom positions by SQUEEZE/PLATON[22].

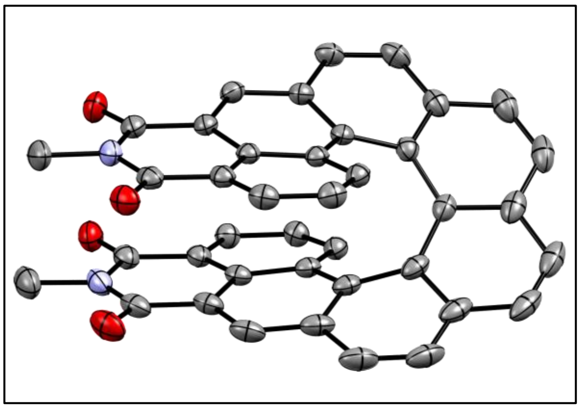

Figure S28. Thermal ellipsoids of **(_P_)-_N_-Me-[8]HDI** at the 50% probability level. The hydrogen atoms are omitted for clarity.

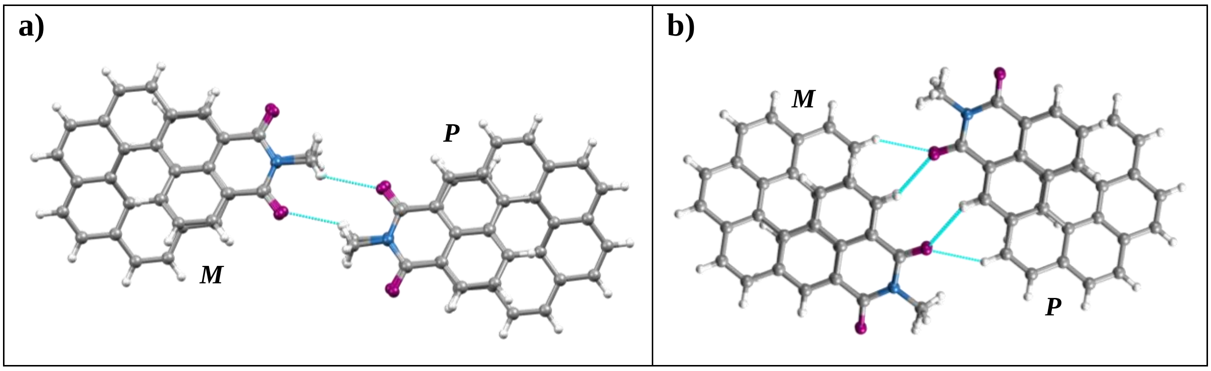

Figure S29. Arrangement of the _P_–_M_-dimers of **_rac_-_N_-Me-[8]HDI** within the crystal. (a) Polymorph 1. (b) Polymorph 2.

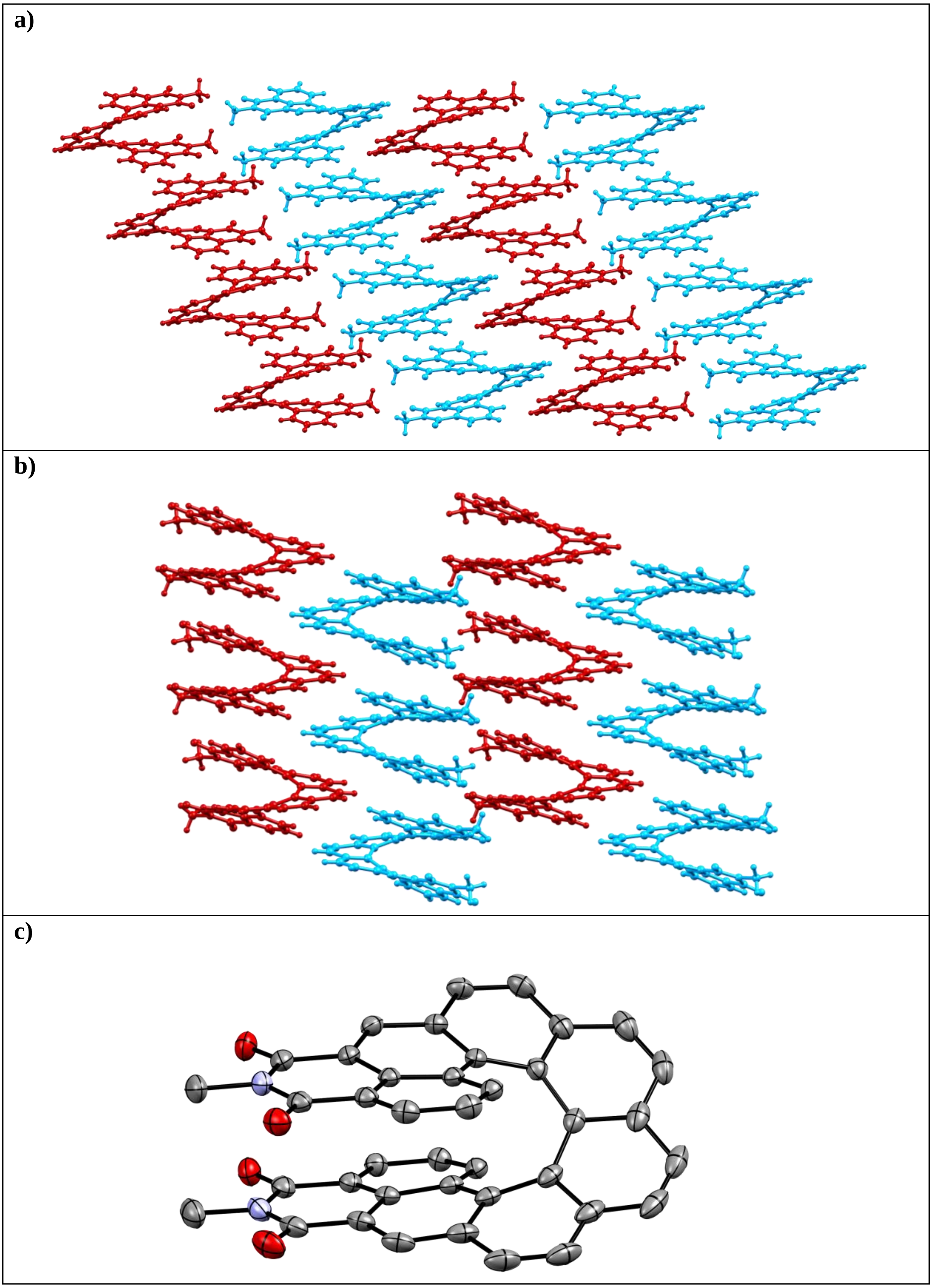

Figure S30. Crystal packing of (a) ***rac*-*N*-Me-[8]HDI** (Polymorph 1) and (b) ***rac*-*N*-Me-[8]HDI** (Polymorph 2). Dark red: *P* enantiomer, light blue: *M* enantiomer. Solvent molecules in the lattice were omitted for clarity. (c) Thermal ellipsoids of **(*P*)-*N*-Me-[8]HDI** at the 50% probability level. The hydrogen atoms are omitted for clarity.

Table S15. Crystal data and structure refinement for ***N*-Me-[8]HDI**.

Compound	**(*P*)-*N*-Me -[8]HDI**	***rac*-*N*-Me-[8]HDI** (Polymorph 1)	***rac*-*N*-Me-[8]HDI** (Polymorph 2)
CCDC number	2434336	2434337	2434338
Chemical formula	$C_{40}H_{22}N_2O_4$	$C_{40}H_{22}N_2O_4$	$C_{40}H_{22}N_2O_4$
M_r	594.59	594.59	594.59
Crystal system, space group	Tetragonal, $P4_32_12$	Triclinic, P_1	Triclinic, P_1
Temperature / K	100	102	100
a, *b*, *c* / Å	12.27117 (4), 12.27117(4), 18.53359 (11)	7.77184 (11), 11.1036 (2), 15.4399 (3)	7.30866 (12), 13.8136 (3), 15.0695 (3)
α, β, γ / °	90, 90, 90	82.2171 (15), 80.0312 (13), 86.2993 (13)	75.2830 (16), 78.1290 (15), 81.5093 (15)
V / Å^3	2790.82 (2)	1299.03 (4)	1432.69 (5)
Z	4	2	1
Radiation type	Cu $K\alpha$	Cu $K\alpha$	Cu $K\alpha$
μ / mm^{-1}	0.74	0.80	0.72
Crystal size / mm	0.51 × 0.41 × 0.30	0.27 × 0.21 × 0.15	0.34 × 0.18 × 0.07
T_{min}, T_{max}	0.712, 1.000	0.600, 1.000	0.449, 1.000
No. of meas., ind. and observed [$I > 2\sigma(I)$] reflections	35495, 2810, 2806	24951, 5118, 4918	28700, 5631, 5210
R_{int}	0.020	0.013	0.019
(sin θ / λ) max / Å^{-1}	0.623	0.627	0.625
$R[F^2 > 2\sigma(F^2)]$, $wR(F^2)$, S	0.028, 0.074, 1.08	0.041, 0.118, 1.09	0.043, 0.119, 1.04
No. of reflections	2810	5118	5631
No. of parameters	210	417	419
$\Delta\rho_{max}$, $\Delta\rho_{min}$ / e Å^{-3}	0.17, −0.16	0.23, −0.21	0.23, −0.32
Flack parameter	0.06 (3)	–	–

***N*-*n*Bu-[8]HDI**

Single crystals of ***rac*- *N*-*n*Bu-[8]HDI** were grown by slow evaporation of a saturated solution of the compound in chloroform in an NMR tube at room temperature. The measured crystal was obtained as a clear yellow block. Two butyl chains were disordered. The 1–2 and 1–3 distances between chain atoms were restrained to the theoretical value (1.55 and 2.54 respectively). Additionally, the distances between alpha Carbon and nitrogen atoms were restrained during refinement to the same value. The displacement parameters of disordered atoms were restrained to the same value with similarity restraint SIMU.

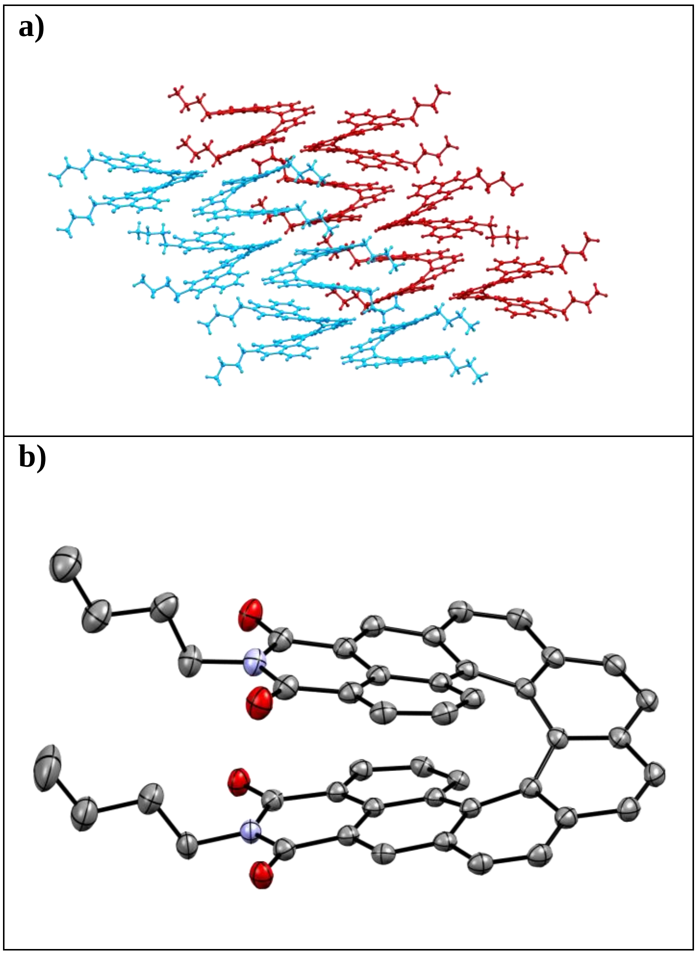

Figure S31. (a) Crystal packing of ***rac*-*N*-*n*Bu-[8]HDI** (dark red: *P* enantiomer, light blue: *M* enantiomer). (b) Thermal ellipsoids of ***rac*-*N*-*n*Bu-[8]HDI** at the 50% probability level. The hydrogen atoms are omitted for clarity.

Table S16. Crystal data and structure refinement for ***N*-*n*Bu-[8]HDI**.

Compound	***rac*-*N*-*n*Bu-[8]HDI**.
CCDC number	2434339
Chemical formula	$C_{46}H_{34}N_2O_4$
M_r	678.75
Crystal system, space group	Triclinic, *P*
Temperature / K	100
a, *b*, *c* / Å	16.035 (13), 20.753 (18), 22.235 (15)
α, β, γ / °	68.50 (4), 88.25 (4), 79.47 (4)
V / Å^3	6763 (9)
Z	8
Radiation type	Cu *K*α
μ / mm^{-1}	0.68
Crystal size / mm	0.23 × 0.18 × 0.10
T_{min}, T_{max}	0.642, 1.000
No. of measured, independent and observed [$I > 2\sigma(I)$] reflections	232803, 26447, 22639
R_{int}	0.033
(sin θ / λ) max / Å^{-1}	0.627
$R[F^2 > 2\sigma(F^2)]$, $wR(F^2)$, S	0.047, 0.132, 0.97
No. of reflections	26447
No. of parameters	1957
No. of restraints	196
Flack parameter	–
$\Delta\rho_{max}$, $\Delta\rho_{min}$ / e Å^{-3}	0.83, −0.50

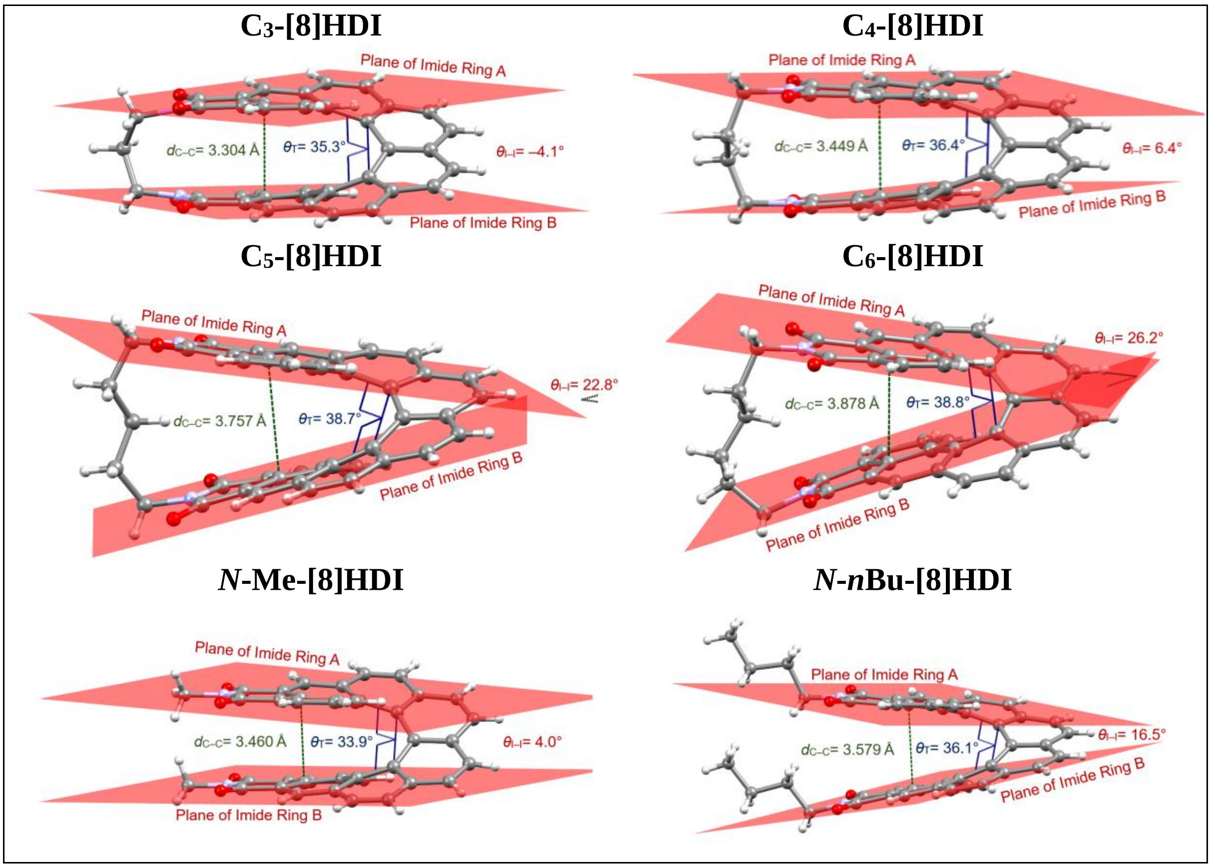

Figure S32. Key measurements from the crystallographic structures of the discussed **[8]HDI**s. $d_{C\text{–}C}$: Distance between the carbon atoms in the center of the imide ring and the terminal two aromatic rings of the helicene (4a and 16a carbon atoms in Figure S33). θ_T: Torsion angle of the [8]helicene backbone (between the 20b–20c and 20e–20f carbon-carbon bonds in Fig. S33), corresponding to 2α. $\theta_{I\text{–}I}$: Dihedral angle between the calculated planes of the six-membered imide rings. Positive values indicate that the imides are further apart than the helical pitch while negative values indicate imides that are closer together than the helical pitch (only the case for **C_3-[8]HDI)**. The unit cell of ***N*-*n*Bu-[8]HDI** contains four molecules with slightly different values of $d_{C\text{–}C}$. The values given above and in Table S17 represent the average value.

Table S17. Measured values for the helical pitch p, as approximated by the distance between the 4a and 16a carbon atoms of the [8]helicene backbone ($d_{C\text{–}C}$) in the crystal structures of the racemic samples, as well as the values from DFT-optimized geometries in the ground state ($d_{C\text{–}C}$ (GS)) and excited state ($d_{C\text{–}C}$ (ES)), the torsion angle θ_T in the crystal and in the DFT-optimized geometry, and the imide-imide angle $\theta_{I\text{–}I}$ in the crystal structures.

Compound	$d_{C\text{–}C}$ (Cryst.) / Å	$d_{C\text{–}C}$ (GS) / Å	$d_{C\text{–}C}$ (ES) / Å	θ_T (Cryst.) / °	θ_T (Calc.) / °	$\theta_{I\text{–}I}$ / °
**C_3-[8]HDI**	3.304	3.345	3.112	35.3	36.5	−4.1
**C_4-[8]HDI**	3.449	3.406	3.313	36.4	37.0	6.4
**C_5-[8]HDI**	3.757	3.447	3.385	38.7	37.3	22.8
**C_6-[8]HDI**	3.878	3.793	3.737	38.8	39.0	26.2
***N*-Me-[8]HDI**	3.460	3.461	3.425	33.9	38.2	4.0
***N*-nBu-[8]HDI**	3.579	3.447	3.393	36.1	38.0	16.5

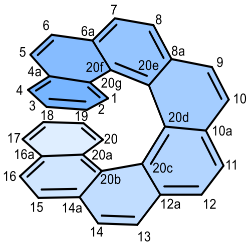

Figure S33. Numbering of the carbon atoms of the [8]helicene backbone in accordance with the IUPAC regulations.[23]

***rac*-C_3-[8]HDI**<br>Homochiral stacks, same orientation<br>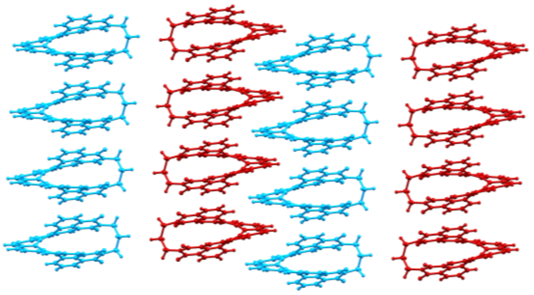	***(M)*-C_3-[8]HDI**<br>Homochiral stacks, 90° offset<br>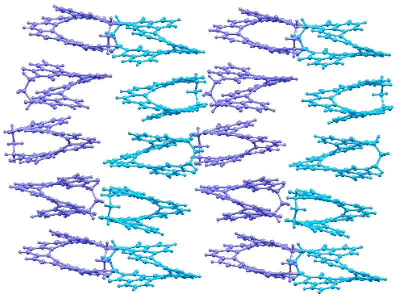
***rac*-C_4-[8]HDI**<br>Homochiral stacks, same orientation<br>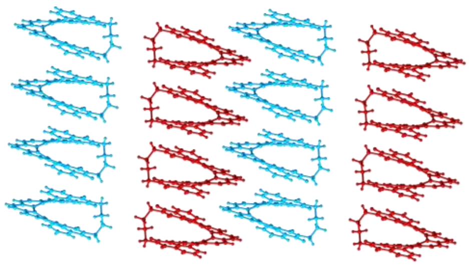	***(M)*-C_4-[8]HDI**<br>Homochiral stacks, same orientation<br>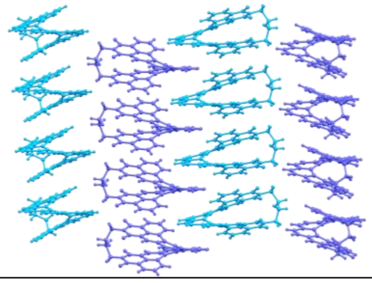
***rac*-C_5-[8]HDI**<br>Stacks of alternating enantiomers, 180° offset<br>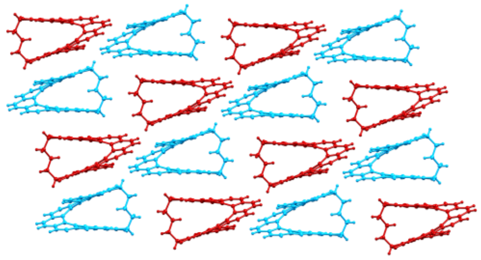	***rac*-C_6-[8]HDI**<br>Homochiral stacks, 180° offset<br>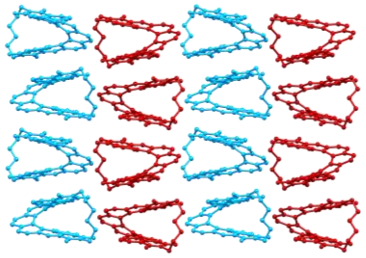
***rac*-*N*-Me-[8]HDI**<br>Homochiral stacks, same orientation<br>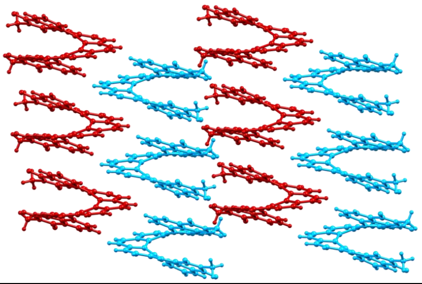	***(P)*-*N*-Me-[8]HDI**<br>Herringbone-like arrangement<br>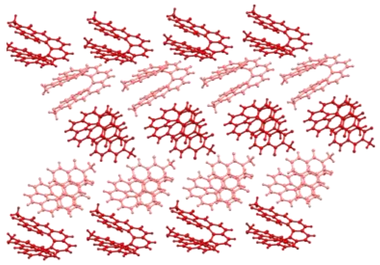

Figure S34. Comparison of the crystal structures of the racemic and enantiopure **[8]HDI**s. (*P*) enantiomers are indicated in red and (*M*) enantiomers in blue in racemic samples.

S8. NMR Spectroscopy

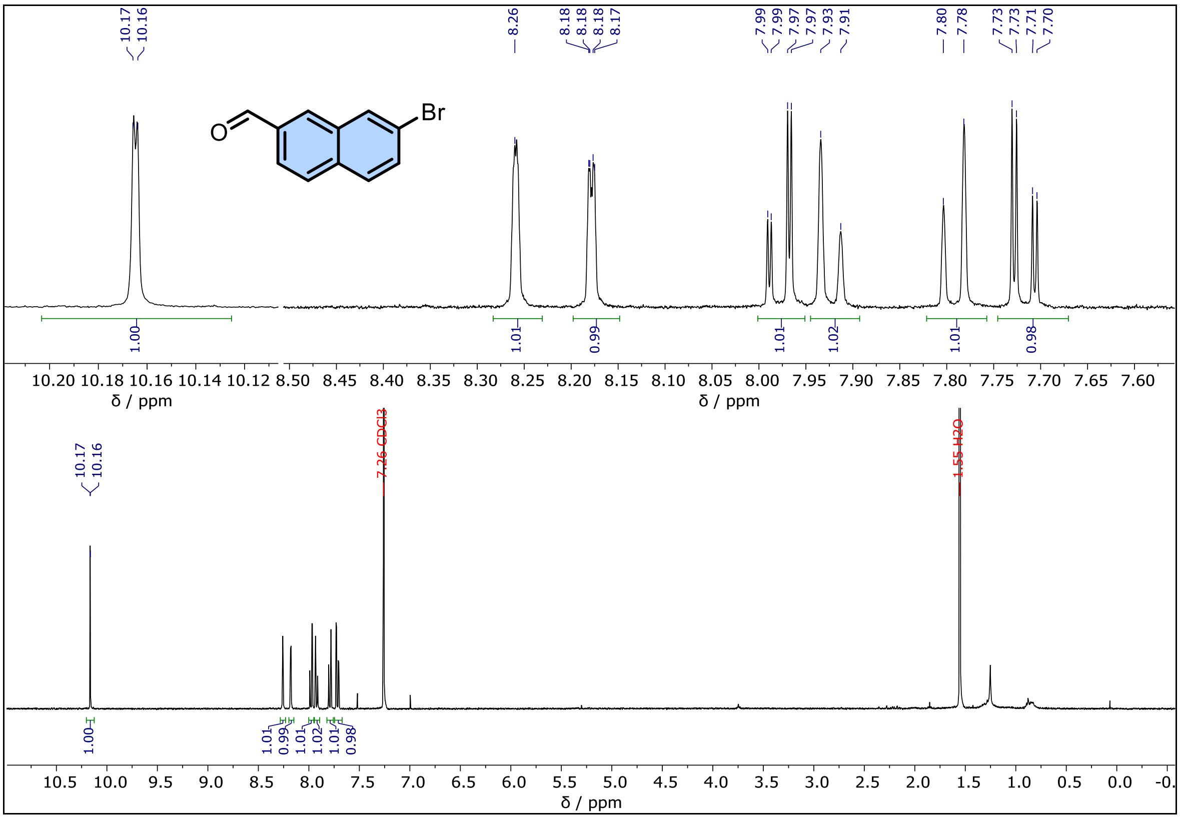

Figure S35. ^{1}H (400 MHz, $CDCl_3$) NMR spectrum of **7-bromo-2-naphthaldehyde**.

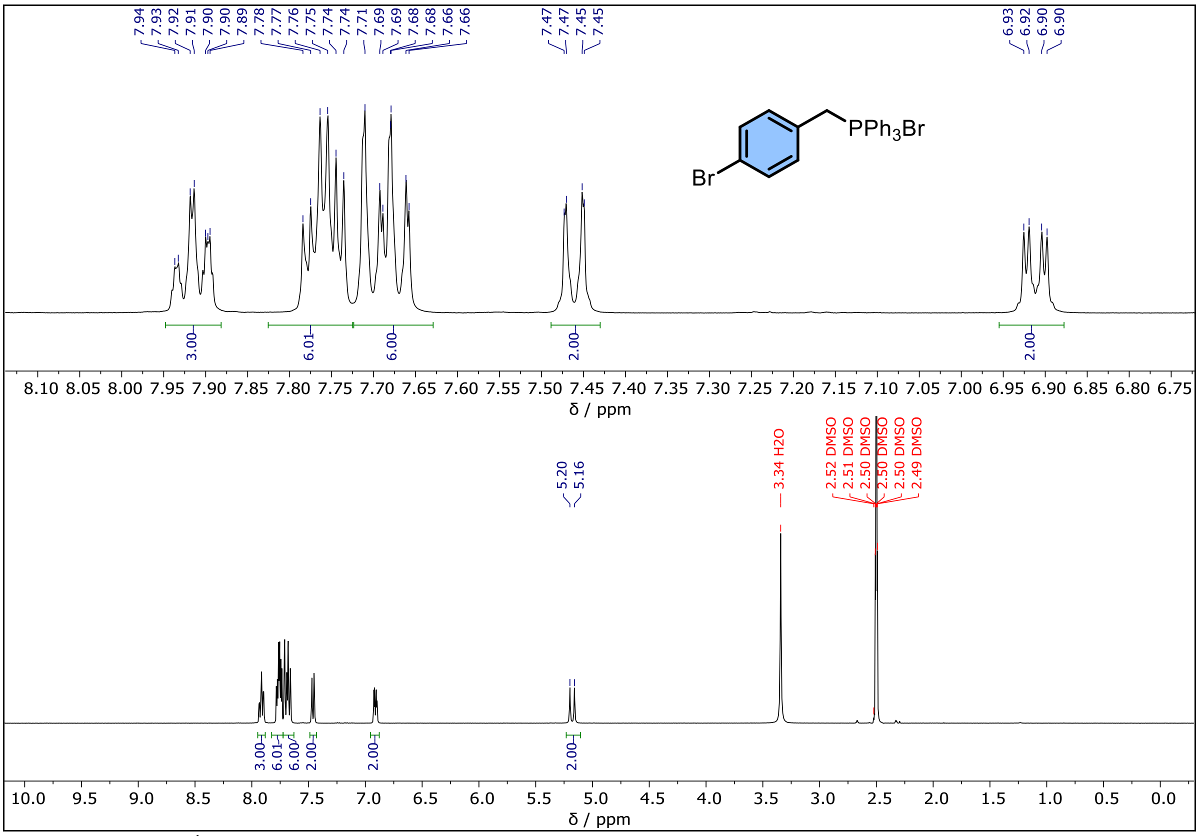

Figure S36. ^{1}H (400 MHz, DMSO-d_6) NMR spectrum of **(4-bromobenzyl)triphenyl-phosphonium bromide**.

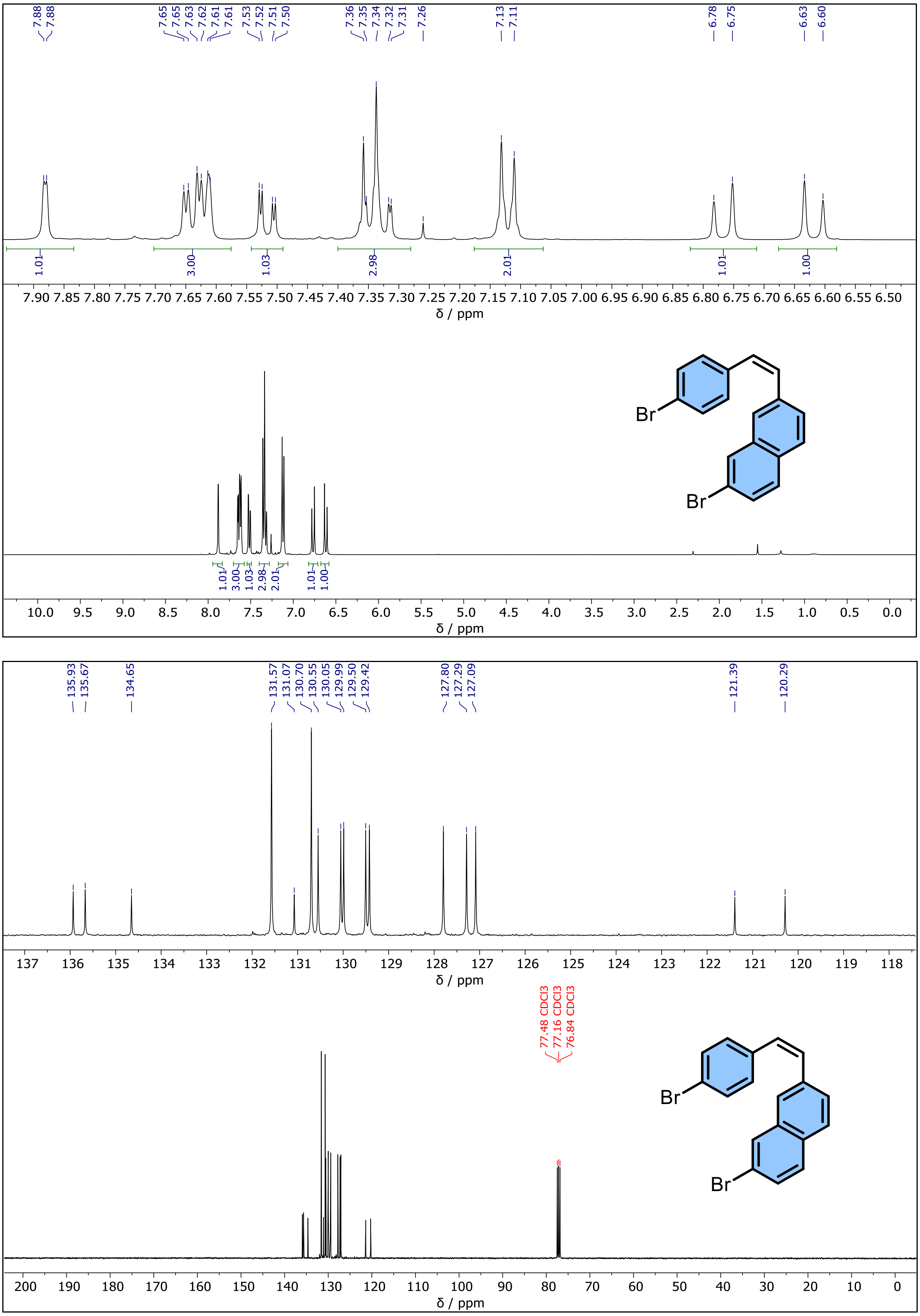

Figure S37. ^{1}H (top, 400 MHz, $CDCl_3$) and ^{13}C{^{1}H} NMR spectra (bottom, 101 MHz, $CDCl_3$) of **(*Z*)-2-bromo-7-(4-bromostyryl)naphthalene**.

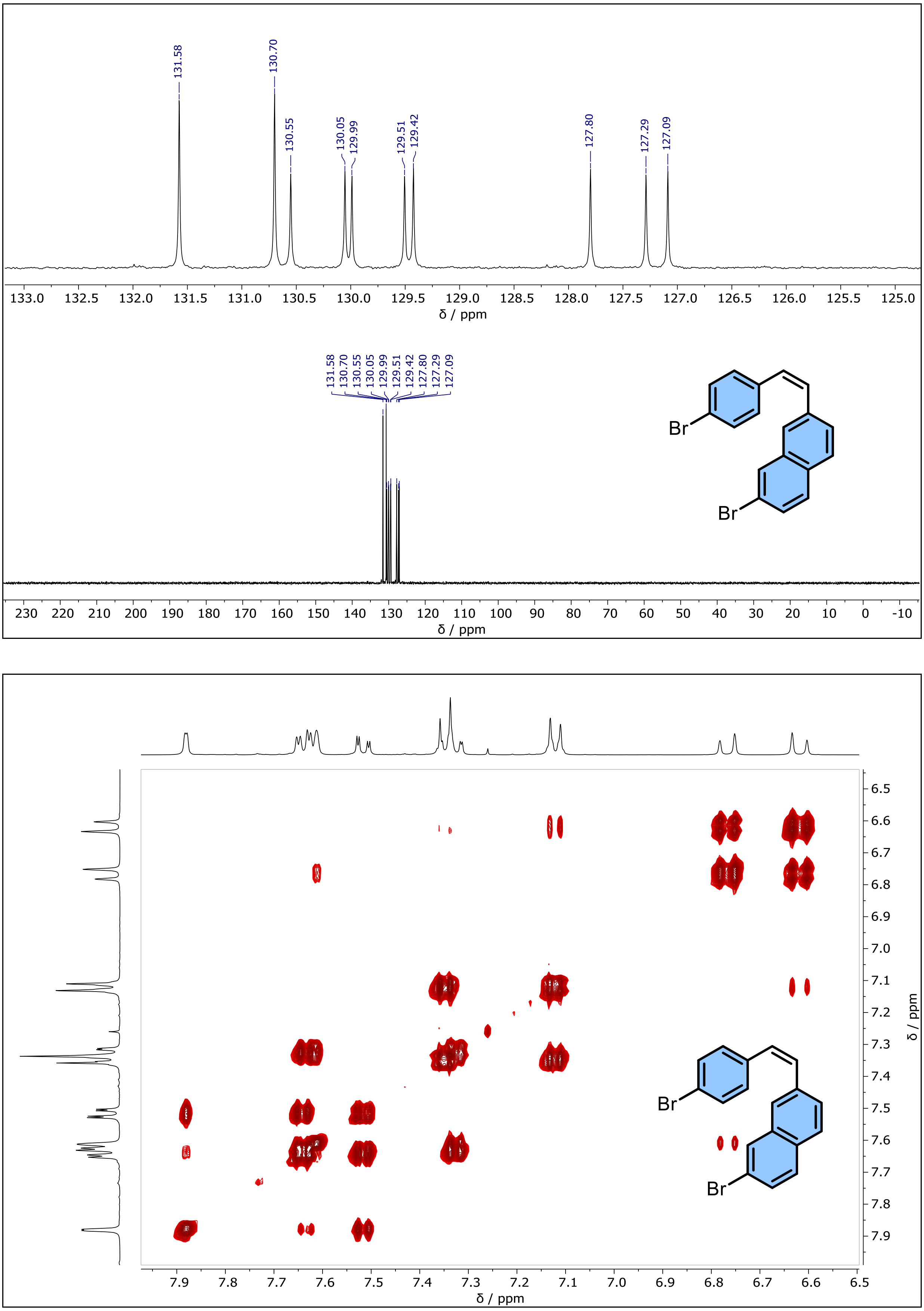

Figure S38. ^{13}C DEPT 135 NMR spectrum (top, 101 MHz, $CDCl_3$) and ^{1}H–^{1}H COSY NMR spectrum (bottom, $CDCl_3$) of **(*Z*)-2-bromo-7-(4-bromostyryl)naphthalene**.

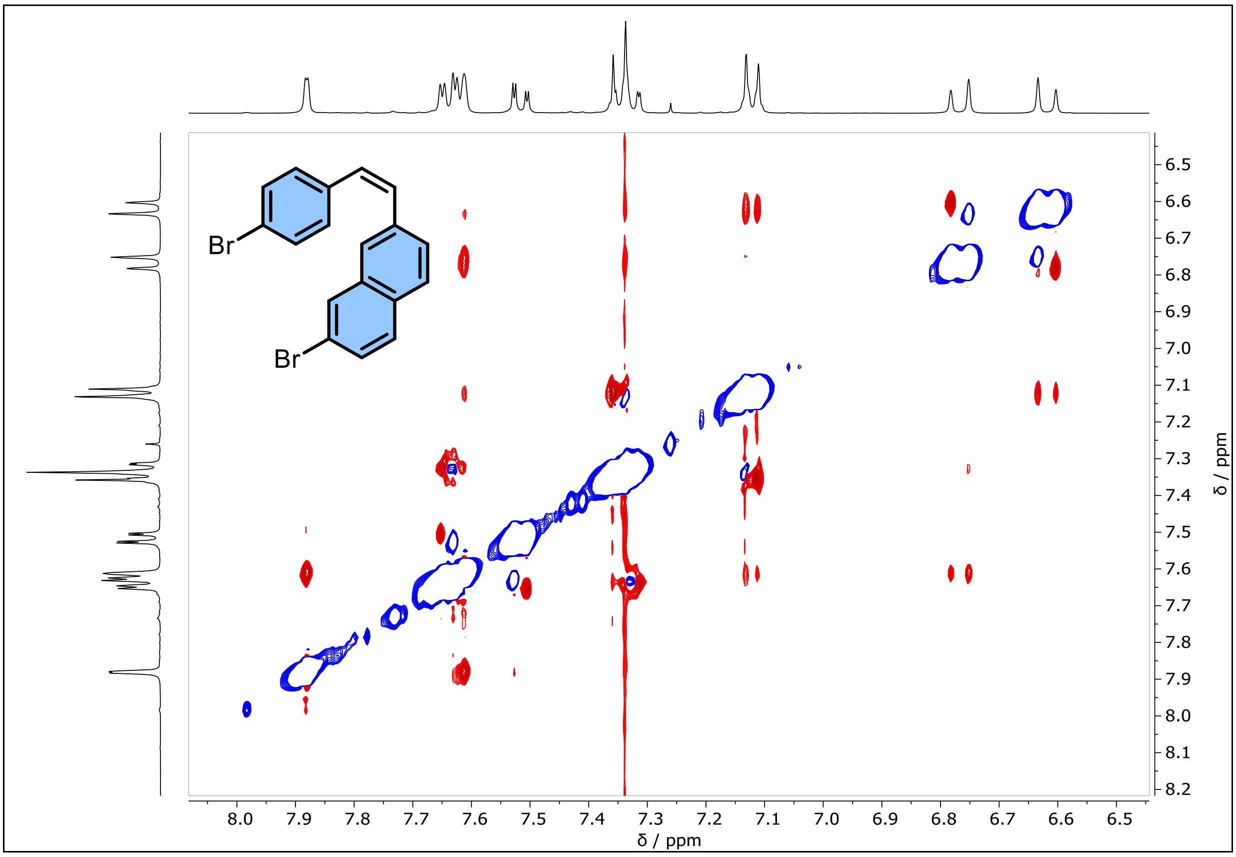

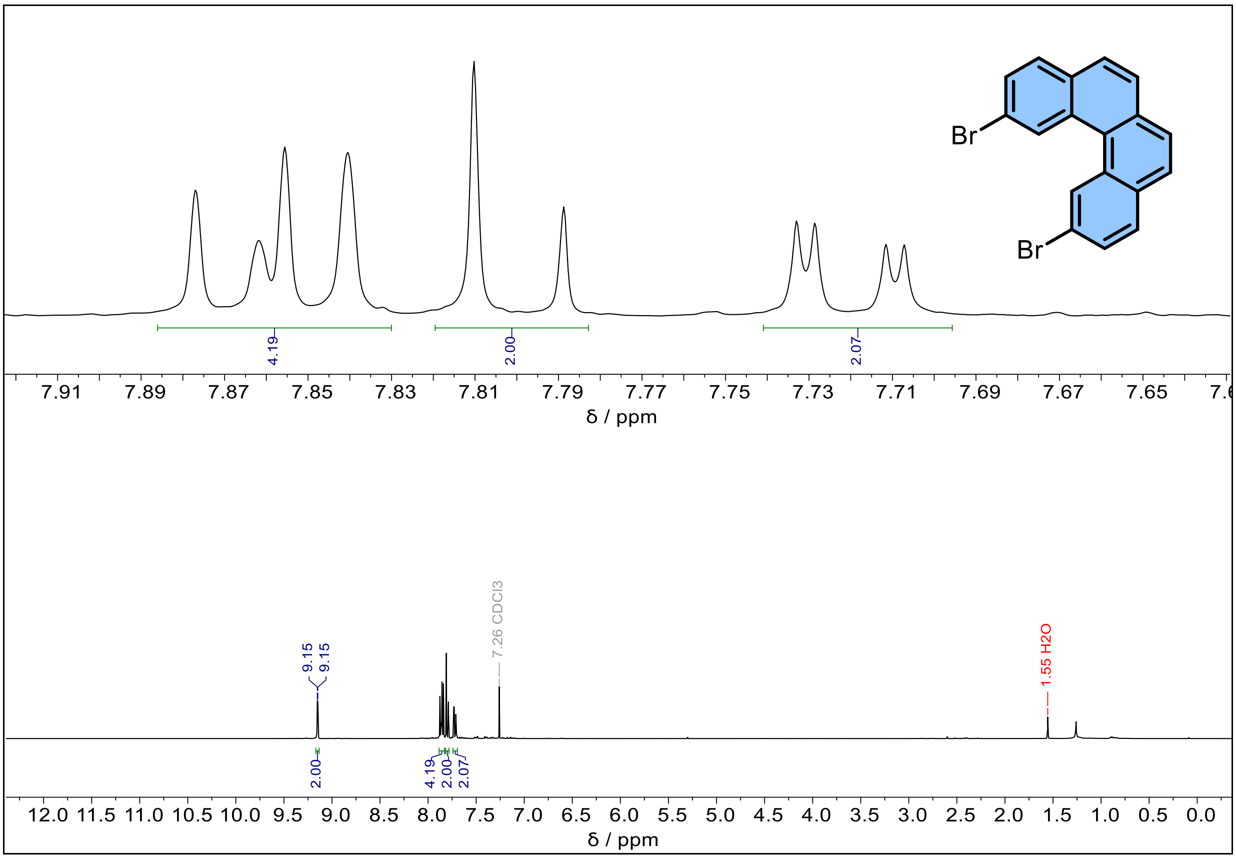

Figure S39. ^{1}H–^{1}H NOESY NMR spectrum (top, 400 MHz, $CDCl_3$) of **(*Z*)-2-bromo-7-(4-bromo-styryl)naphthalene** and ^{1}H (bottom, 400 MHz, $CDCl_3$) NMR spectrum of **2,11-dibromobenzo[c]phenanthrene**.

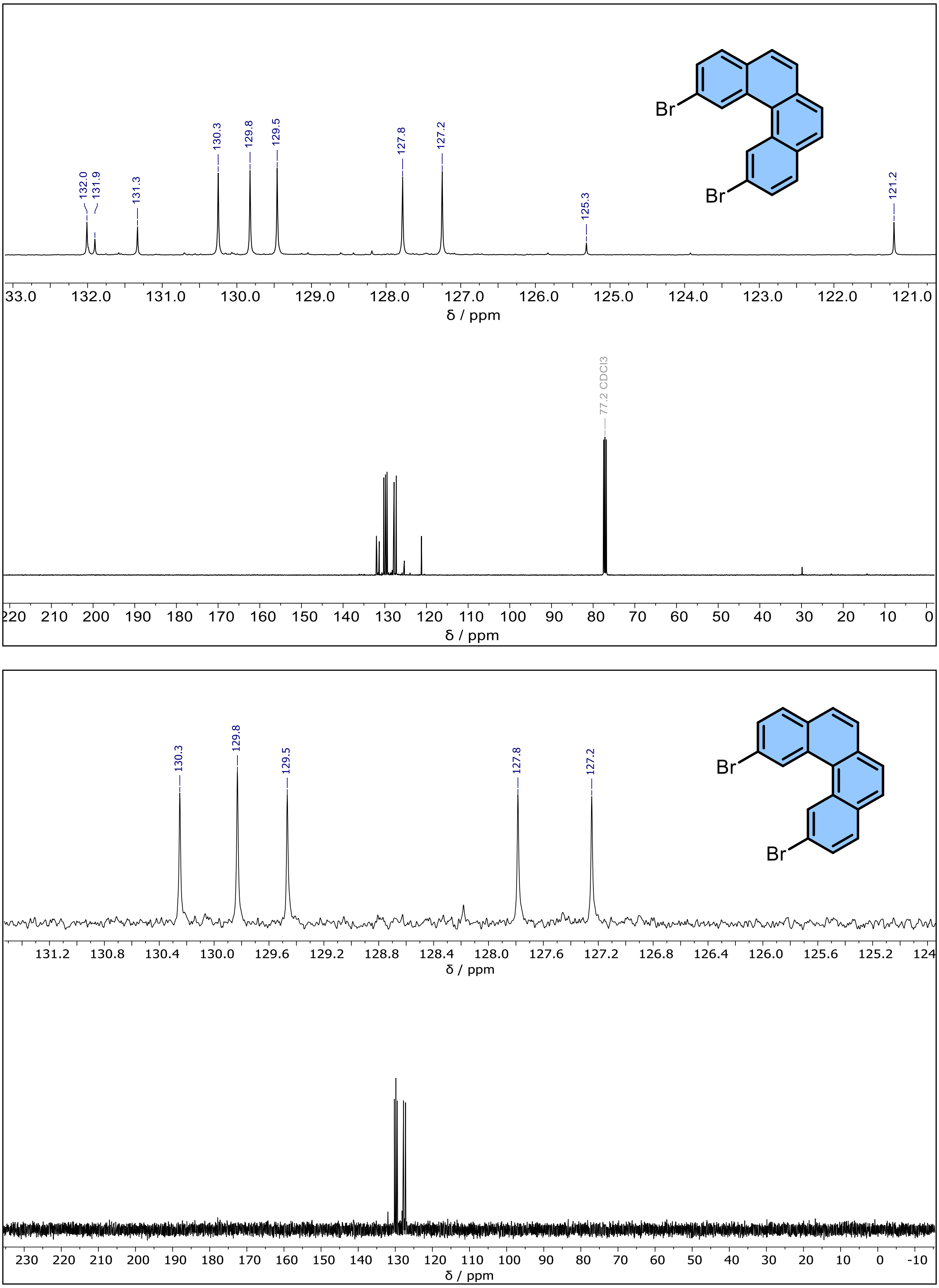

Figure S40. $^{13}C\{^{1}H\}$ NMR spectrum (top, 101 MHz, $CDCl_3$) and ^{13}C DEPT 135 NMR spectrum (bottom, 101 MHz, $CDCl_3$) of **2,11-dibromobenzo[c]phenanthrene**.

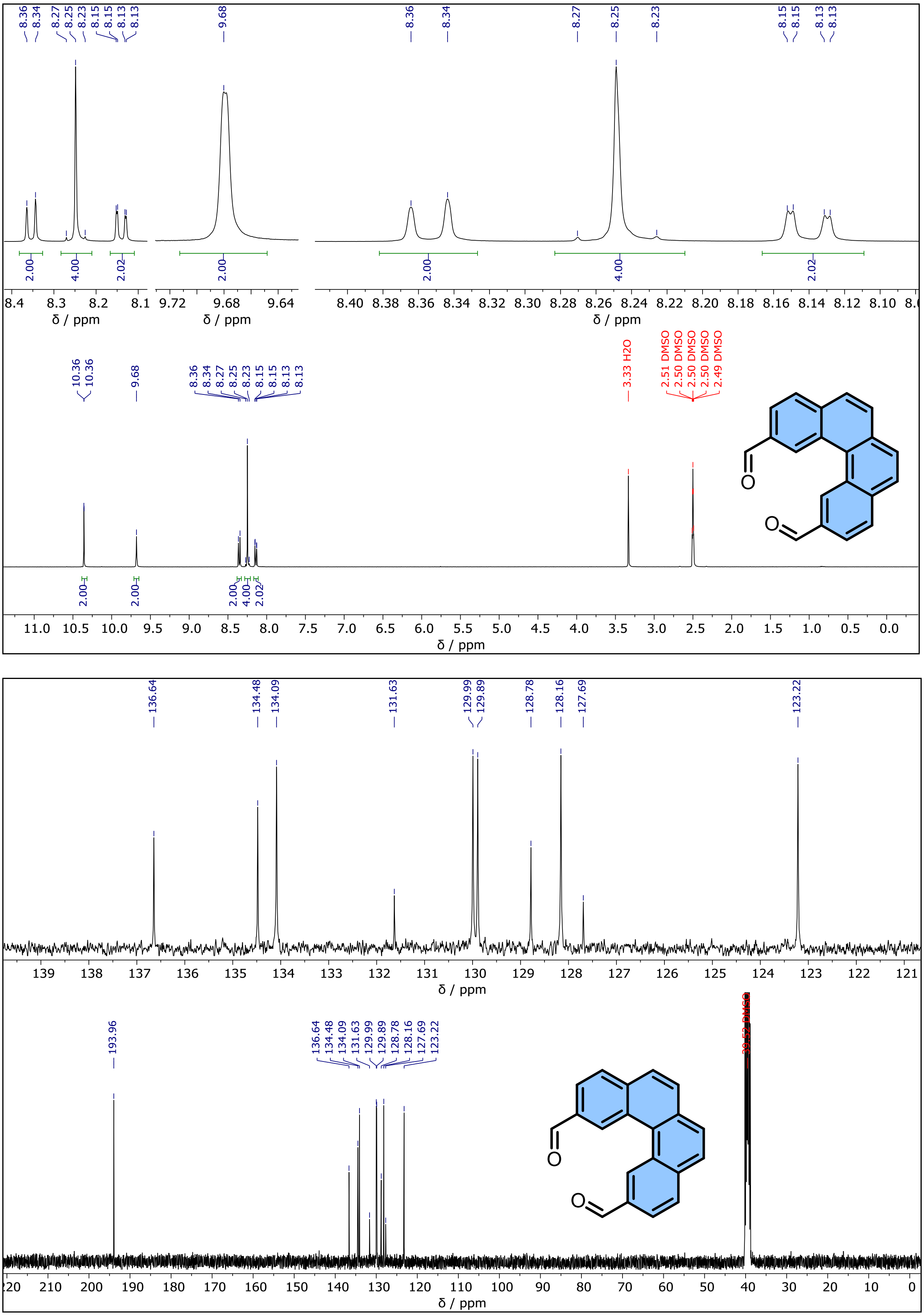

Figure S41. ^{1}H (top, 400 MHz, DMSO-d$_6$) and $^{13}C\{^{1}H\}$ NMR spectra (bottom, 101 MHz, DMSO–d$_6$) of **benzo[c]phenanthrene-2,11-dicarbaldehyde** (**1**).

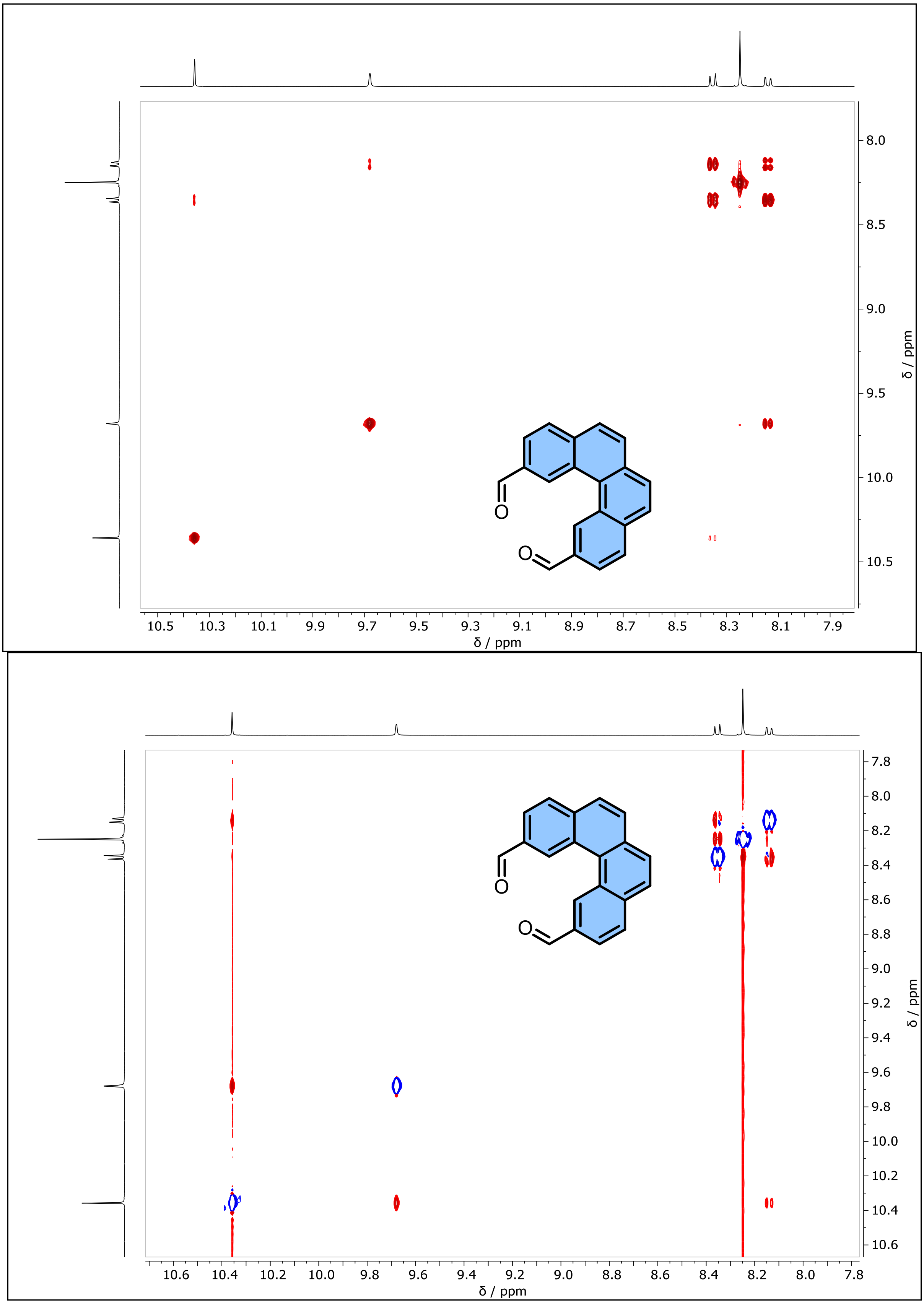

Figure S42. ^{1}H–^{1}H COSY NMR spectrum (DMSO-d$_6$, top) and ^{1}H–^{1}H NOESY NMR spectrum (DMSO–d$_6$, bottom) of **benzo[c]phenanthrene-2,11-dicarbaldehyde** (**1**).

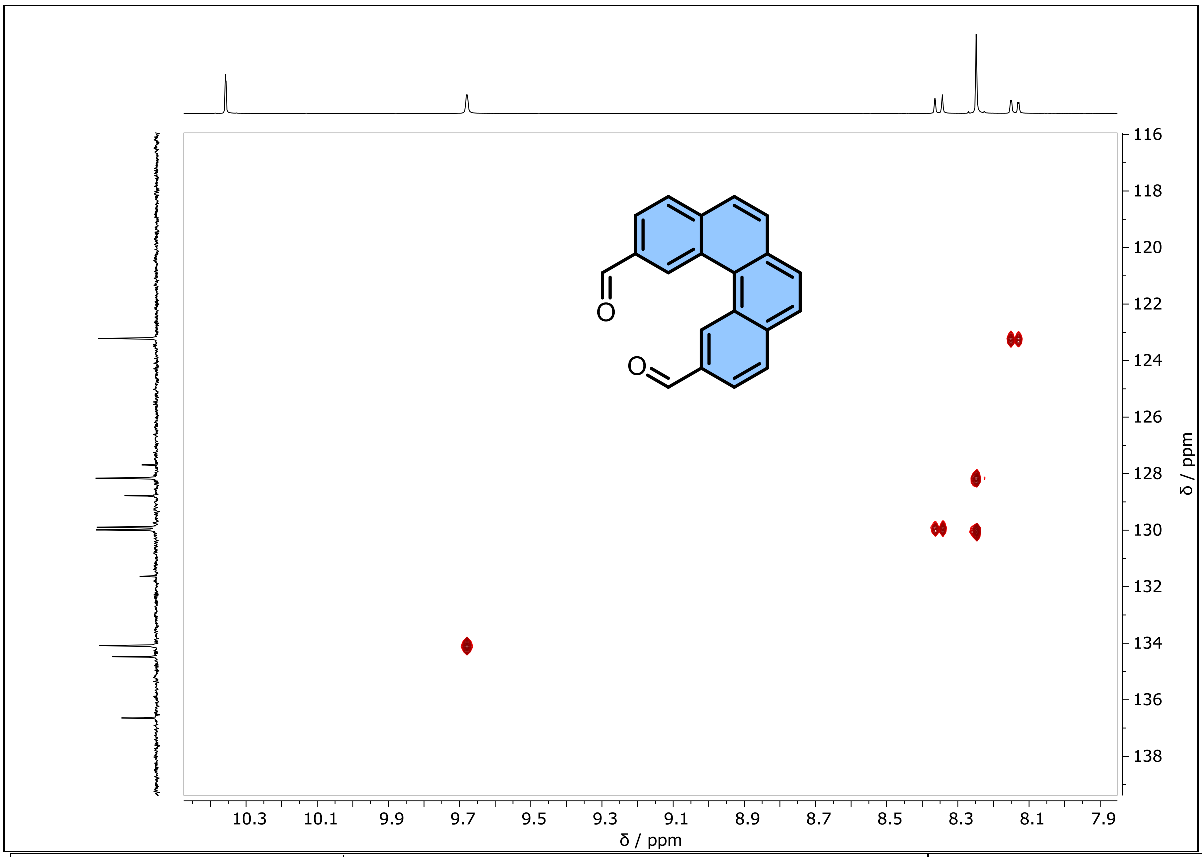

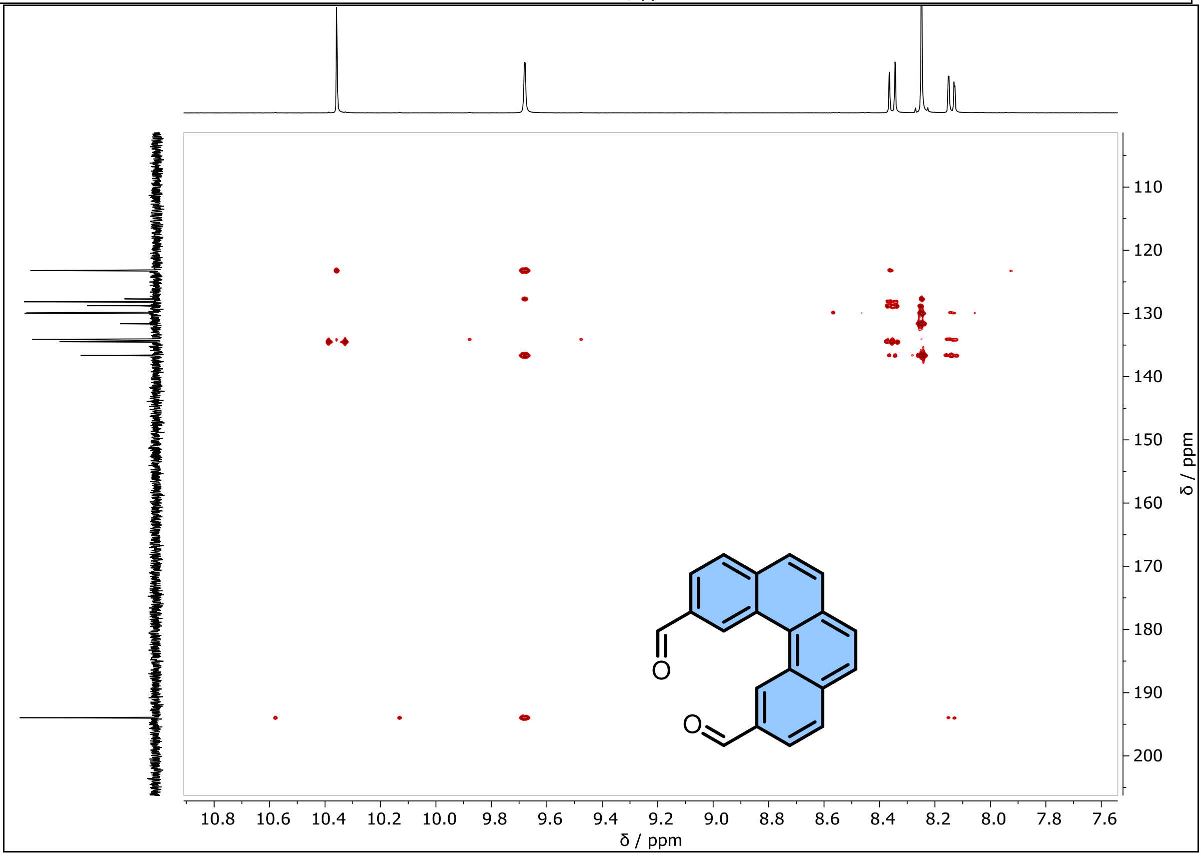

Figure S43. $^{1}H–^{13}C$ HSQC NMR spectrum (DMSO-d$_6$, top) and $^{1}H–^{13}C$ HMBC NMR spectrum (DMSO–d$_6$, bottom) of **benzo[c]phenanthrene-2,11-dicarbaldehyde** (**1**).

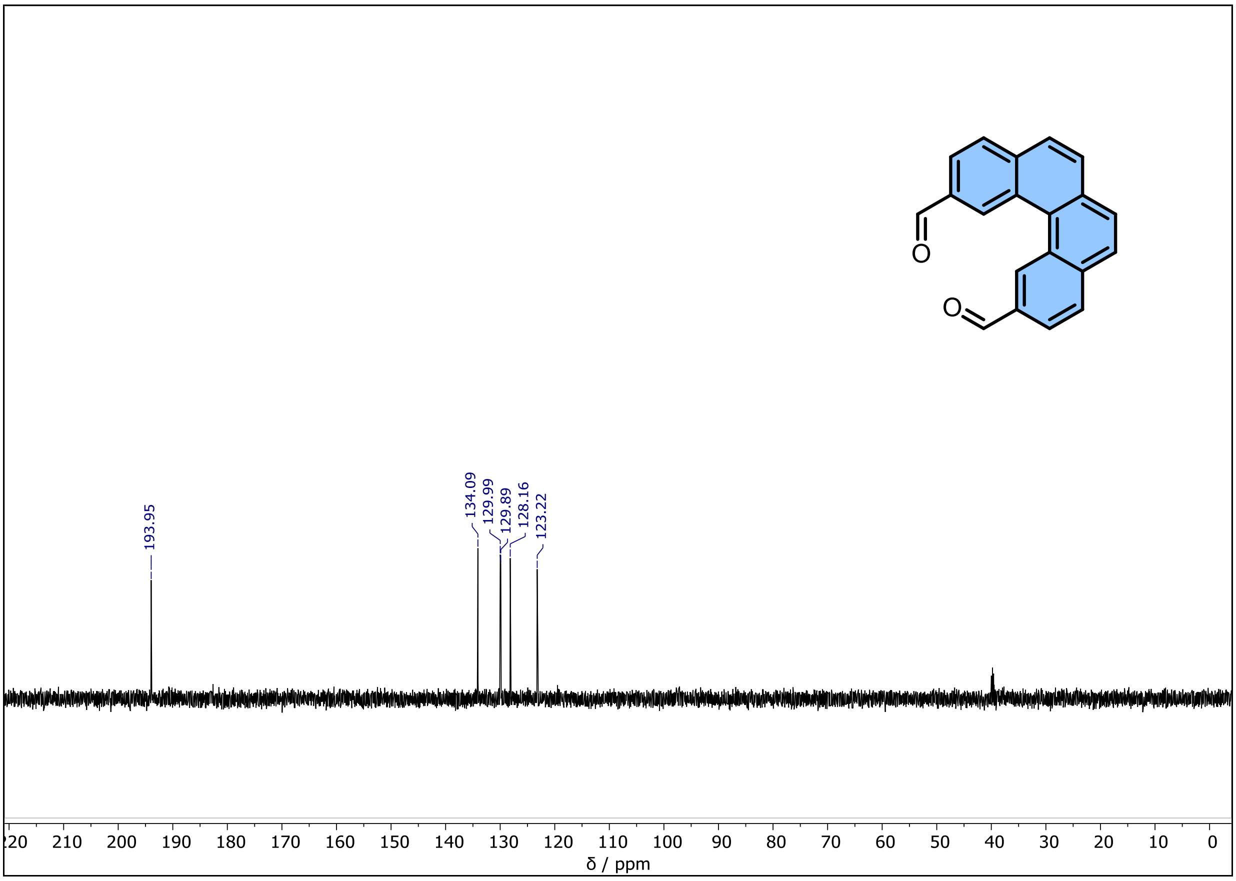

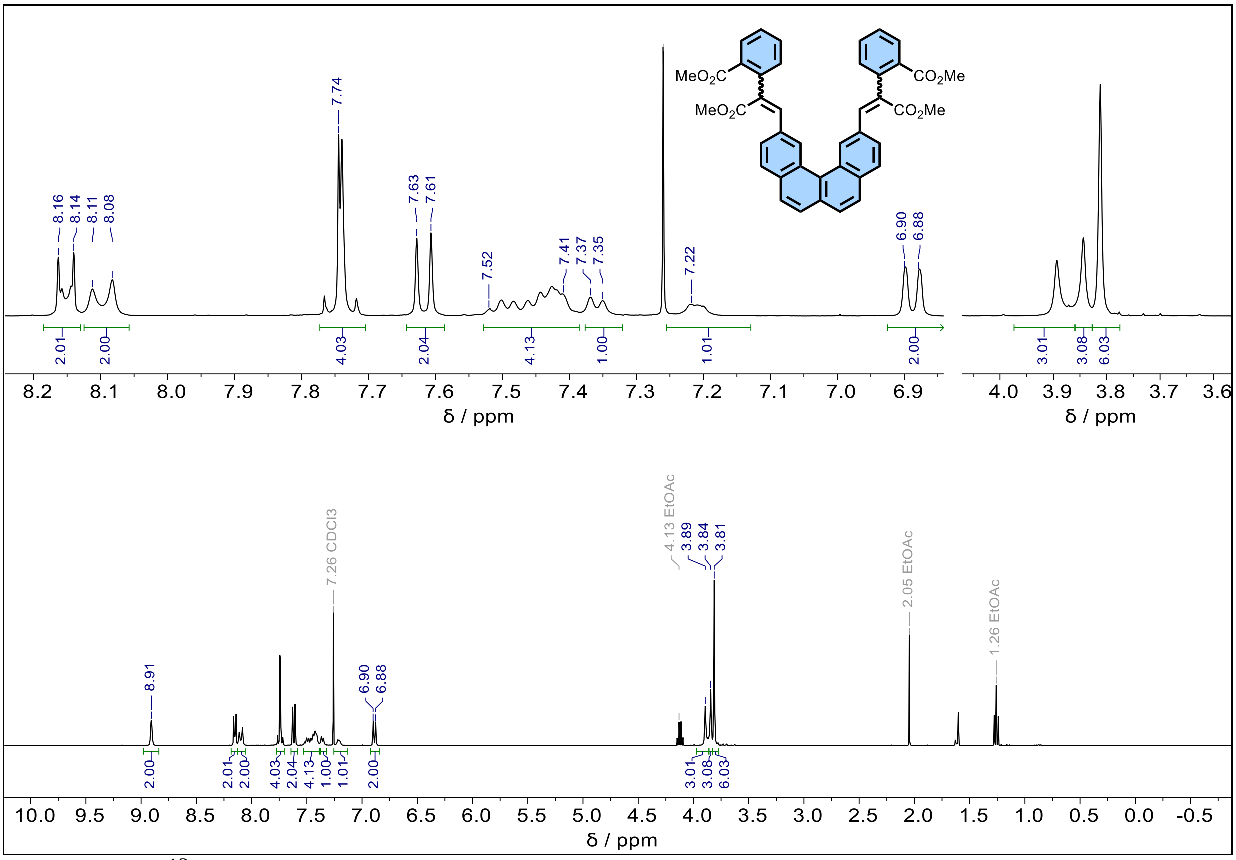

Figure S44. ^{13}C DEPT 135 NMR spectrum (top, 101 MHz, DMSO–d$_6$) of **benzo[c]phenanthrene-2,11-dicarbaldehyde** (**1**) and ^{1}H NMR spectrum (bottom, 400 MHz, CDCl$_3$) of **5**.

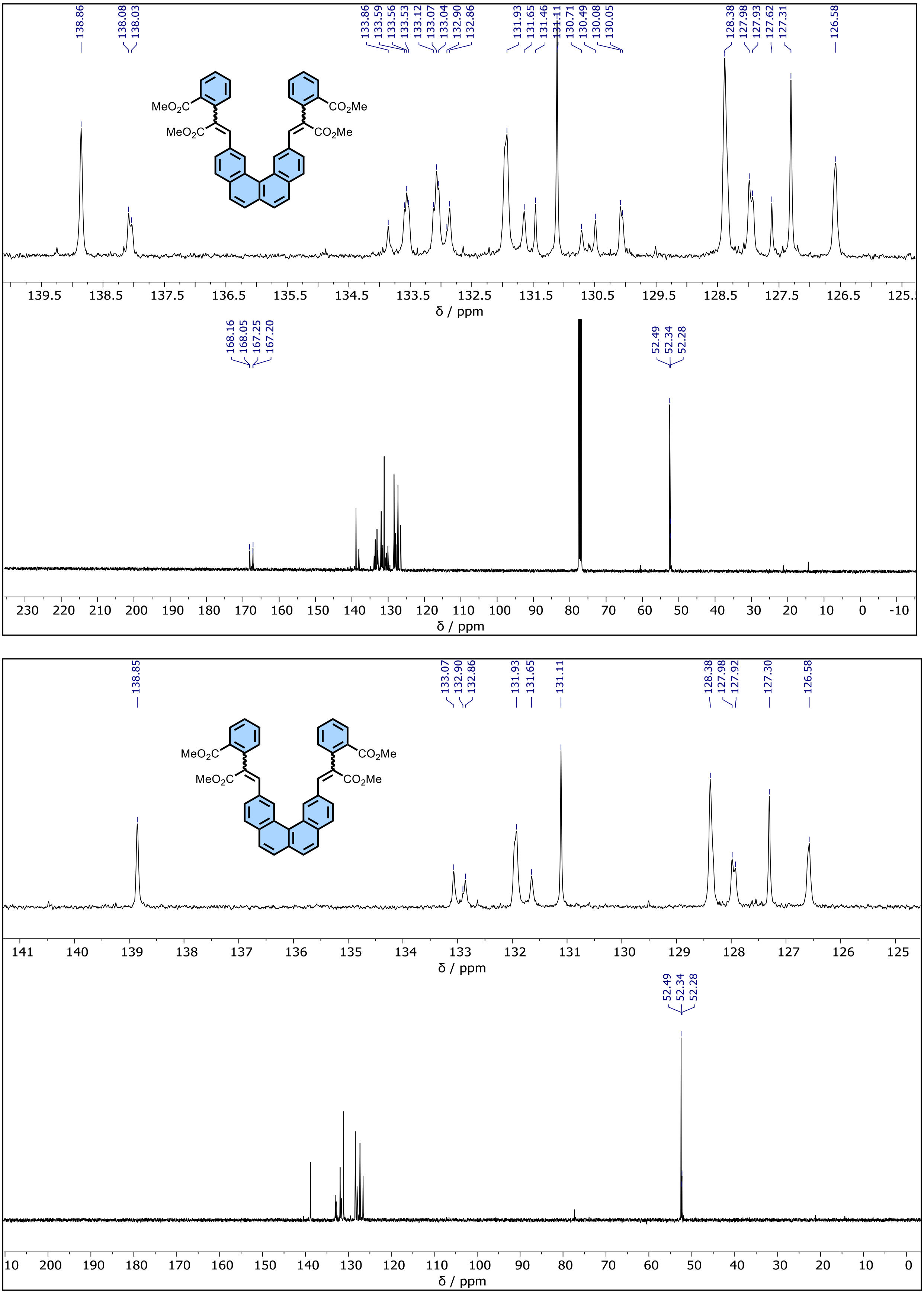

Figure S45. $^{13}C\{^{1}H\}$ NMR spectrum (top, 400 MHz, $CDCl_3$) and ^{13}C DEPT 135 NMR spectrum (bottom, 101 MHz, $CDCl_3$) of **5**.

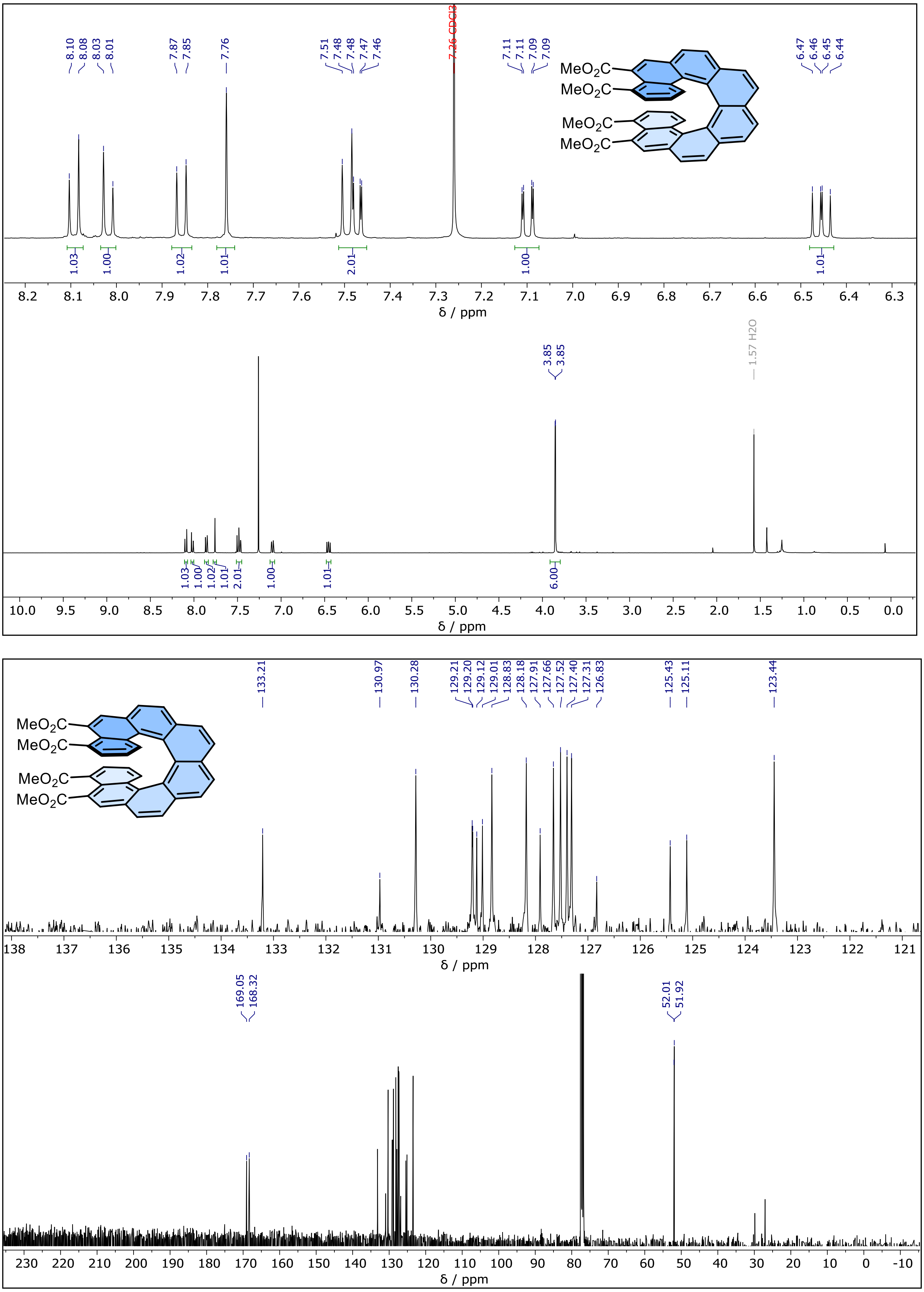

Figure S46. ^{1}H (top, 400 MHz, $CDCl_3$) and $^{13}C\{^1H\}$ NMR spectra (bottom, 101 MHz, $CDCl_3$) of **6**.

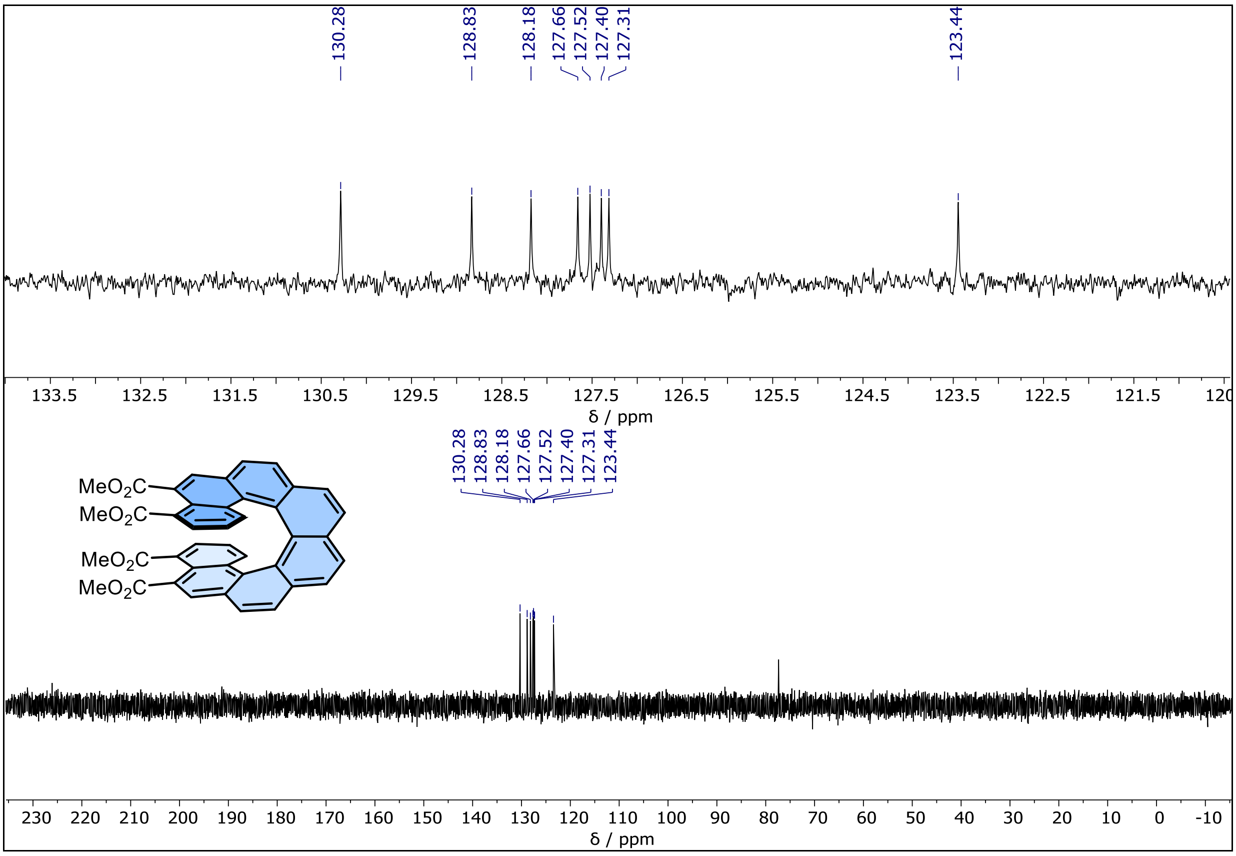

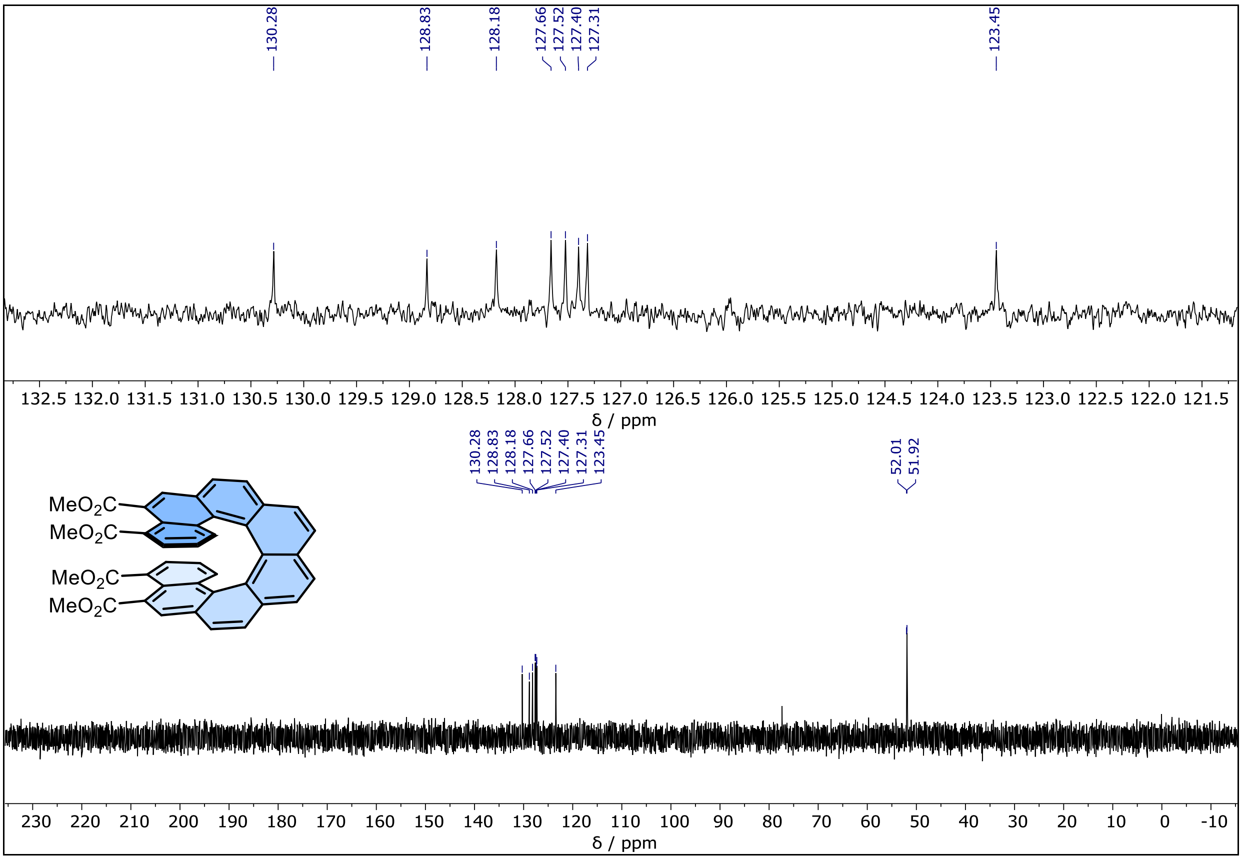

Figure S47. ^{13}C DEPT 90 (top, $CDCl_3$) and DEPT 135 (bottom, $CDCl_3$) NMR spectra of **6**.

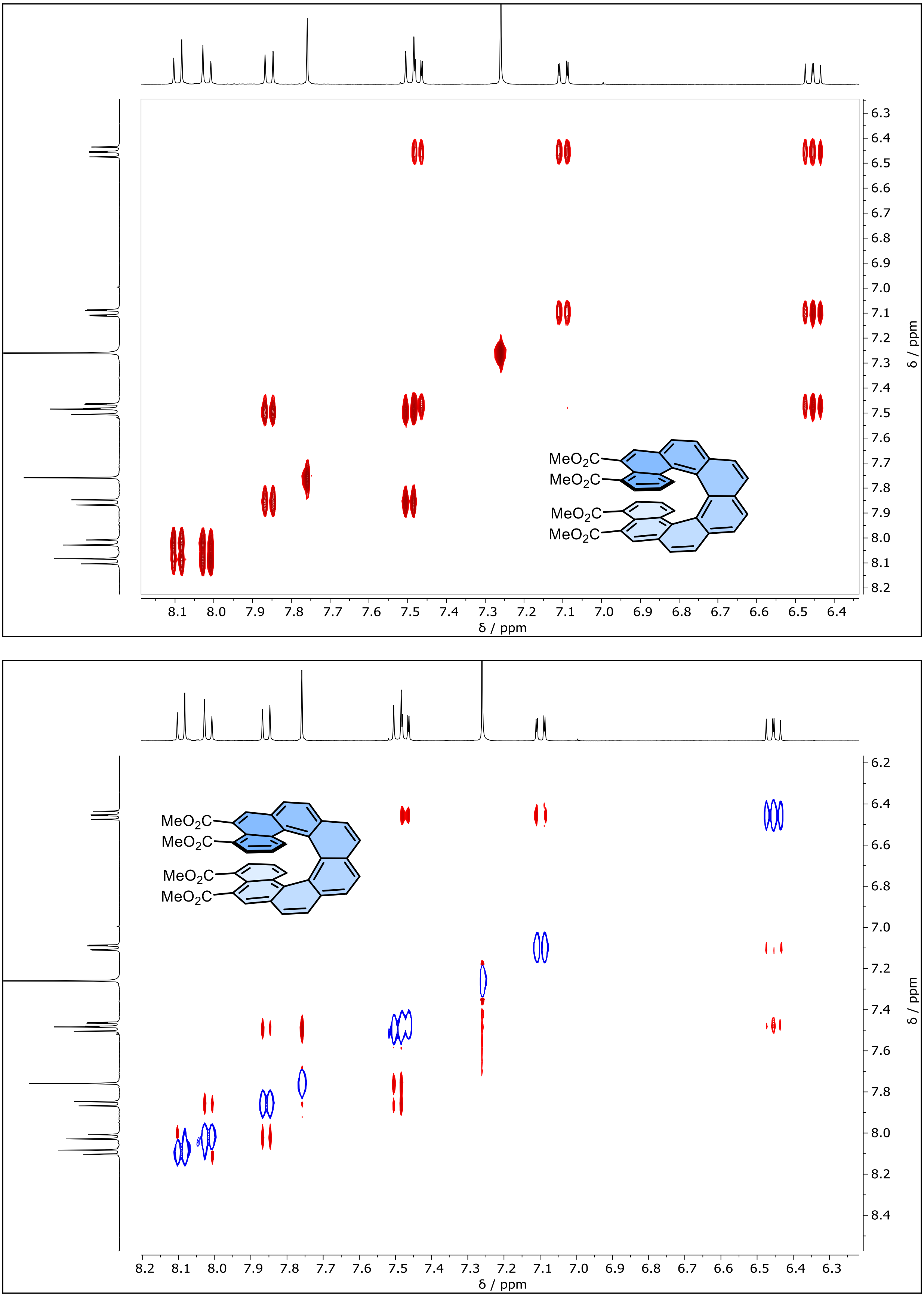

Figure S48. ^{1}H–^{1}H COSY NMR spectrum (top, 400 MHz, $CDCl_3$) and ^{1}H–^{1}H NOESY NMR spectrum (bottom, 400 MHz, $CDCl_3$) of **6**.

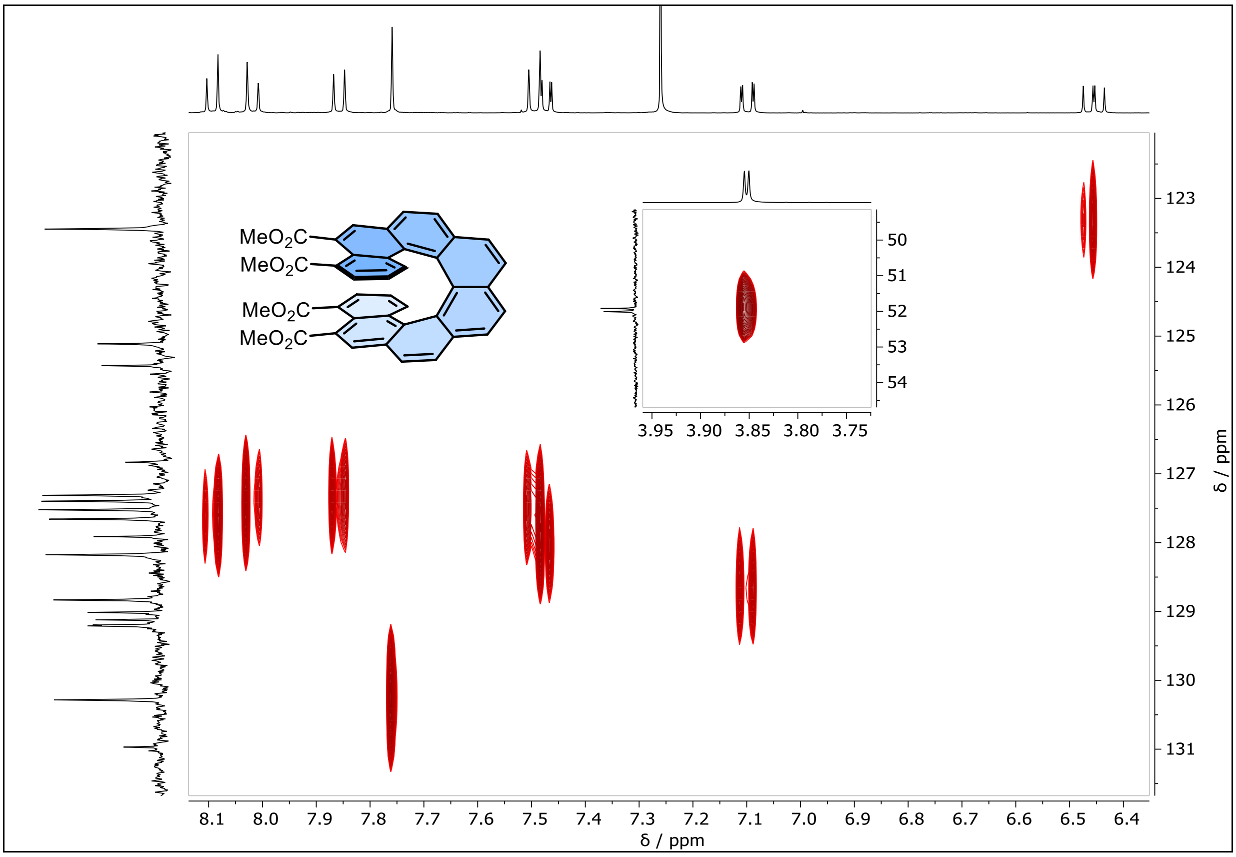

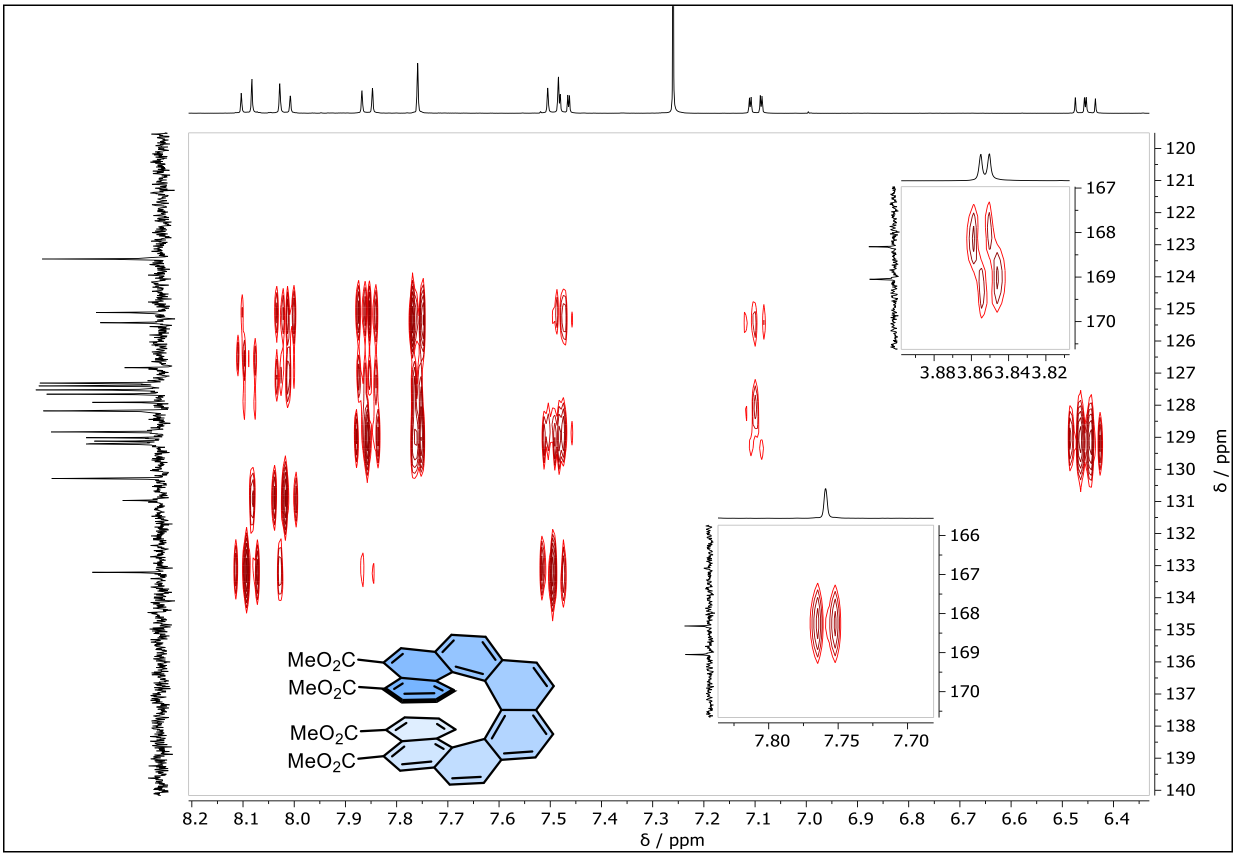

Figure S49. ^1H–^{13}C HSQC NMR spectrum (top, $CDCl_3$) and ^1H–^{13}C HMBC NMR spectrum (bottom, $CDCl_3$) of **6**.

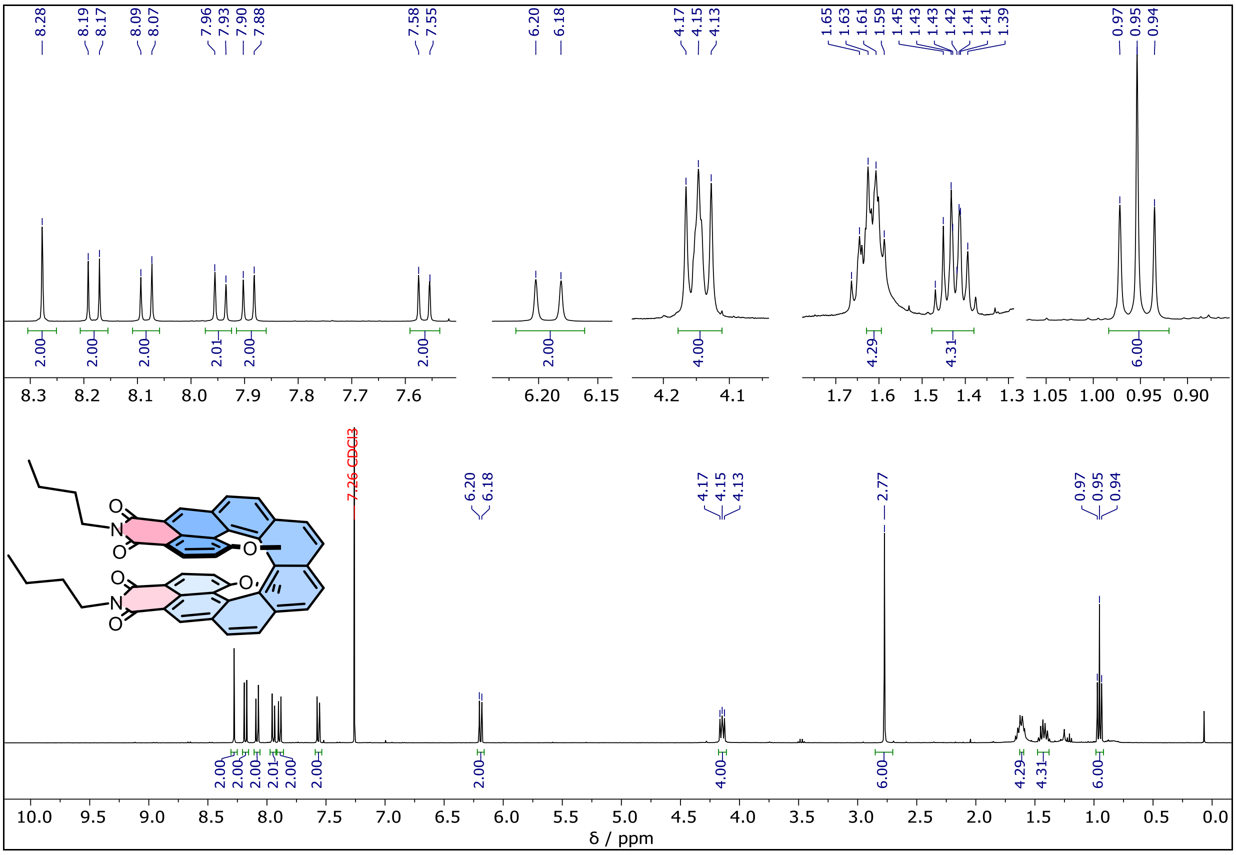

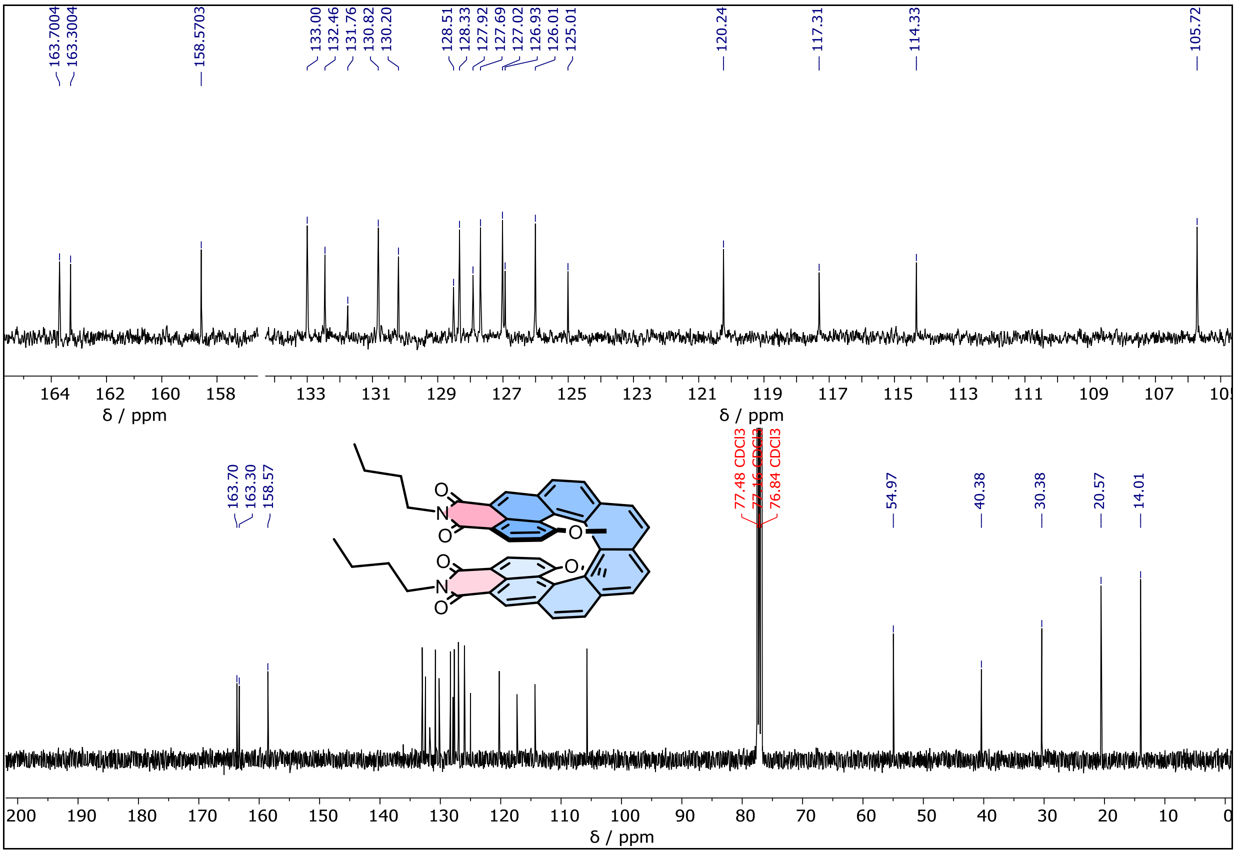

Figure S50. ^{1}H (top, 400 MHz, $CDCl_3$) and $^{13}C\{^{1}H\}$ NMR spectra (bottom, 101 MHz, $CDCl_3$) of ***N*-*n*Bu[8]HDI-*f*-OMe.**

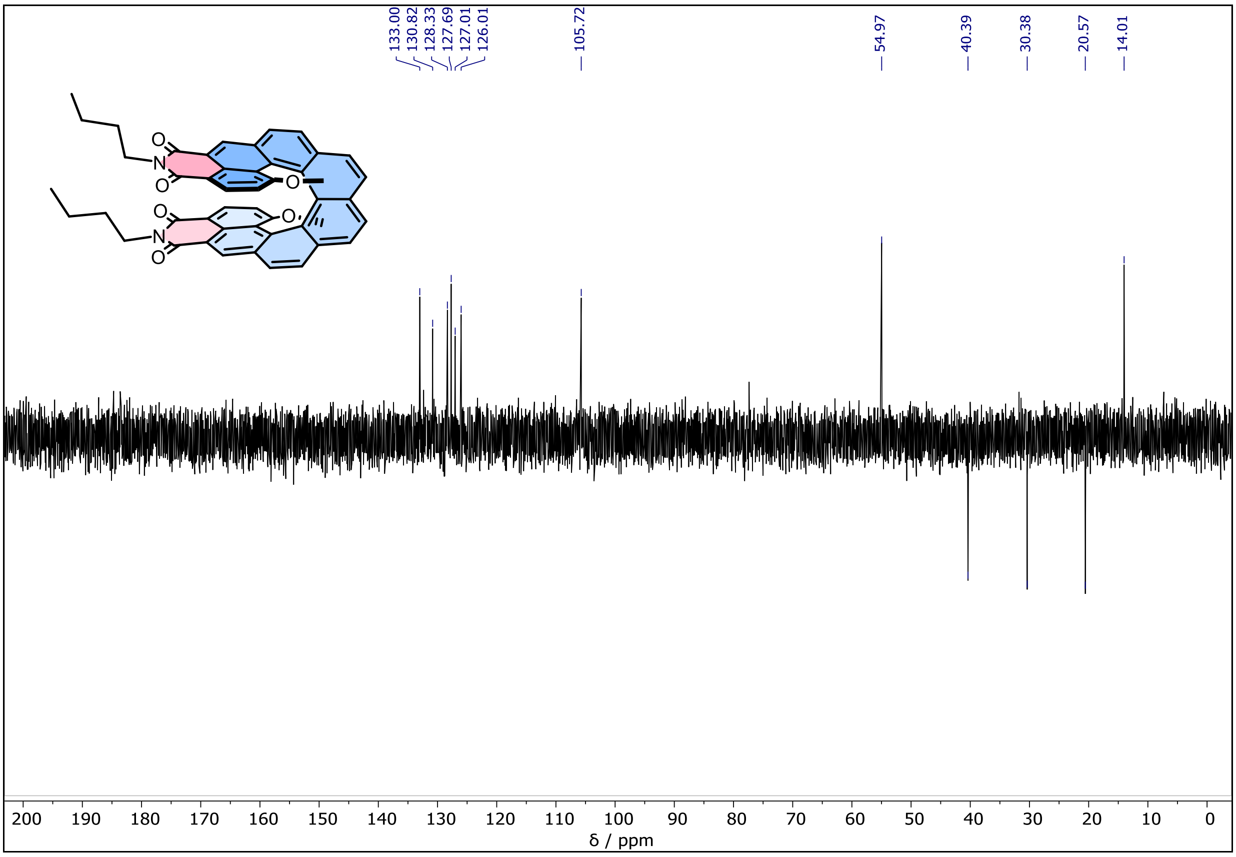

Figure S51. ^{13}C DEPT 135 NMR spectrum (101 MHz, $CDCl_3$) of ***N*-*n*Bu[8]HDI-*f*-OMe.**

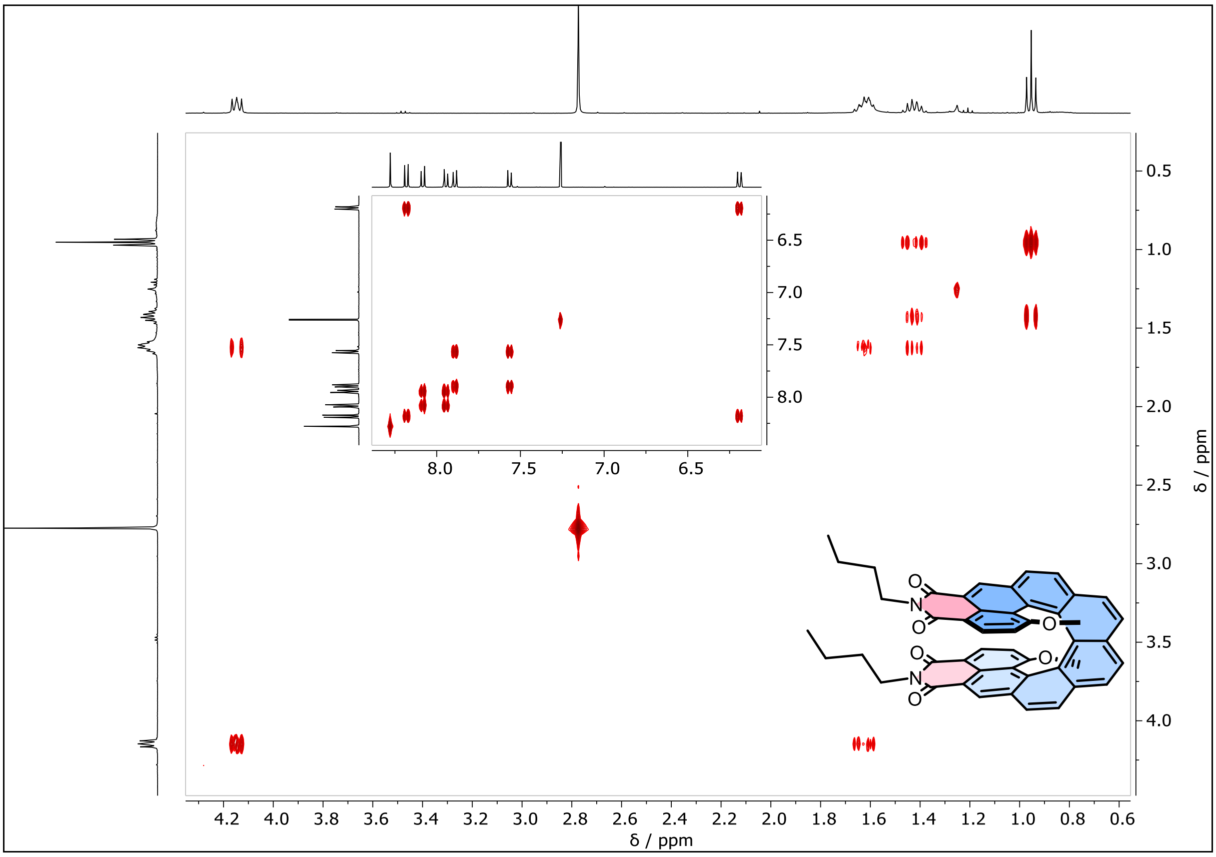

Figure S52. ^{1}H–^{1}H COSY NMR spectrum (400 MHz, $CDCl_3$) of ***N*-*n*Bu[8]HDI-*f*-OMe.**

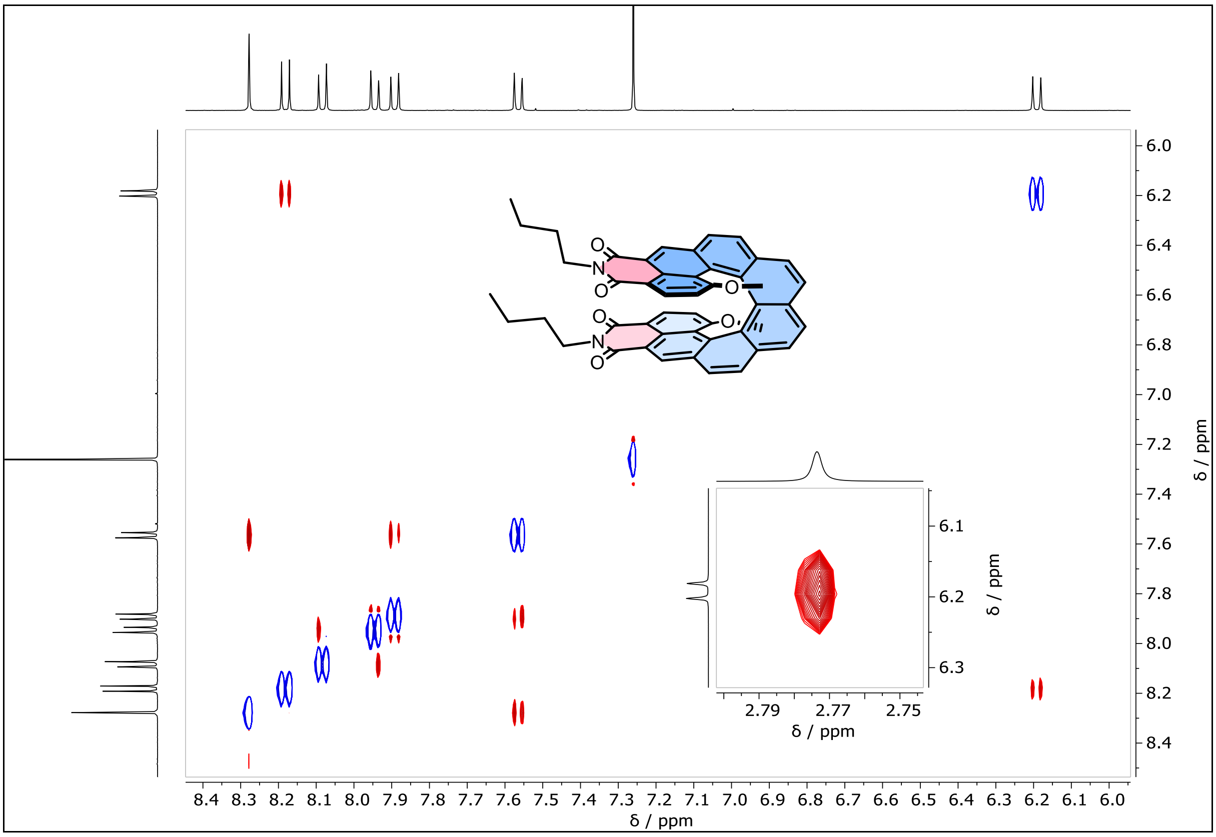

Figure S53. ¹H–¹H NOESY NMR spectrum (400 MHz, $CDCl_3$) of ***N*-*n*Bu[8]HDI-*f*-OMe**.

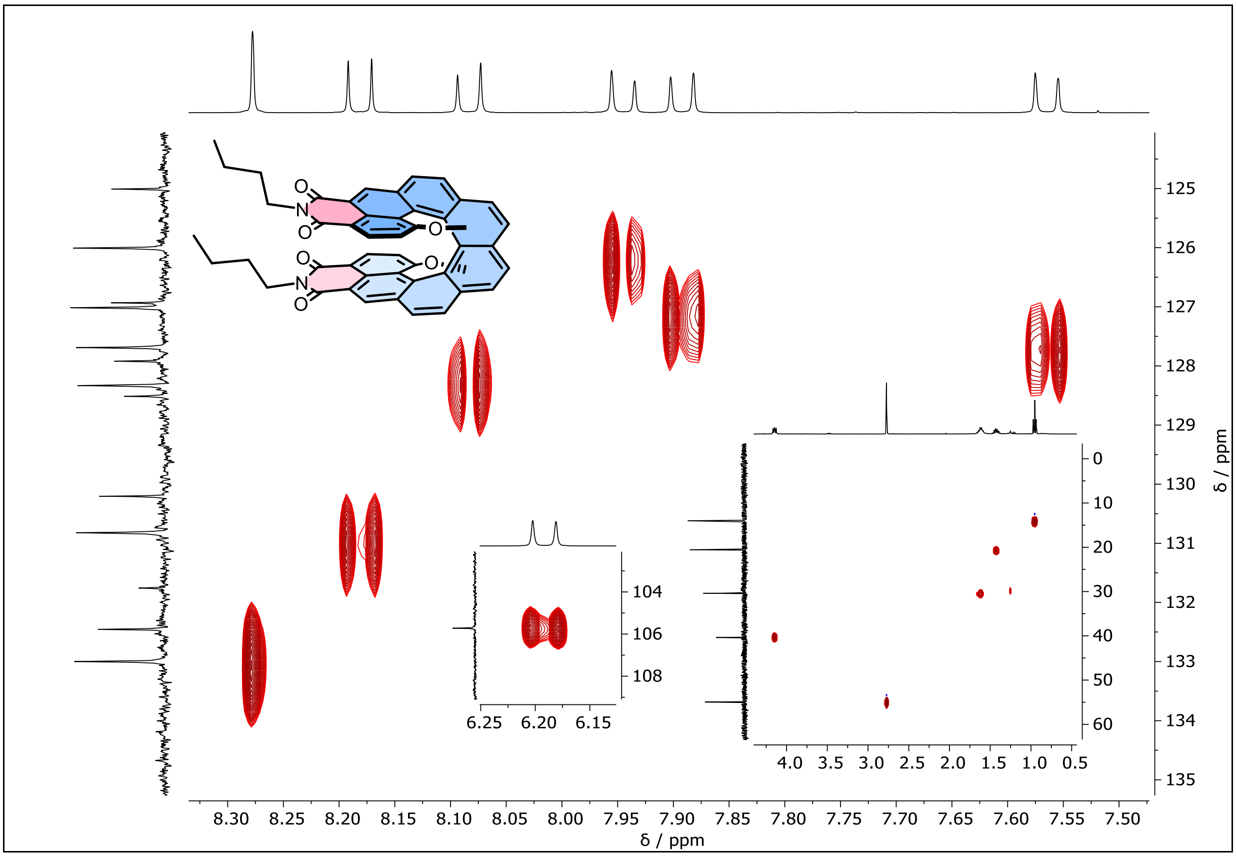

Figure S54. ¹H–¹³C HSQC NMR spectrum ($CDCl_3$) of ***N*-*n*Bu[8]HDI-*f*-OMe**.

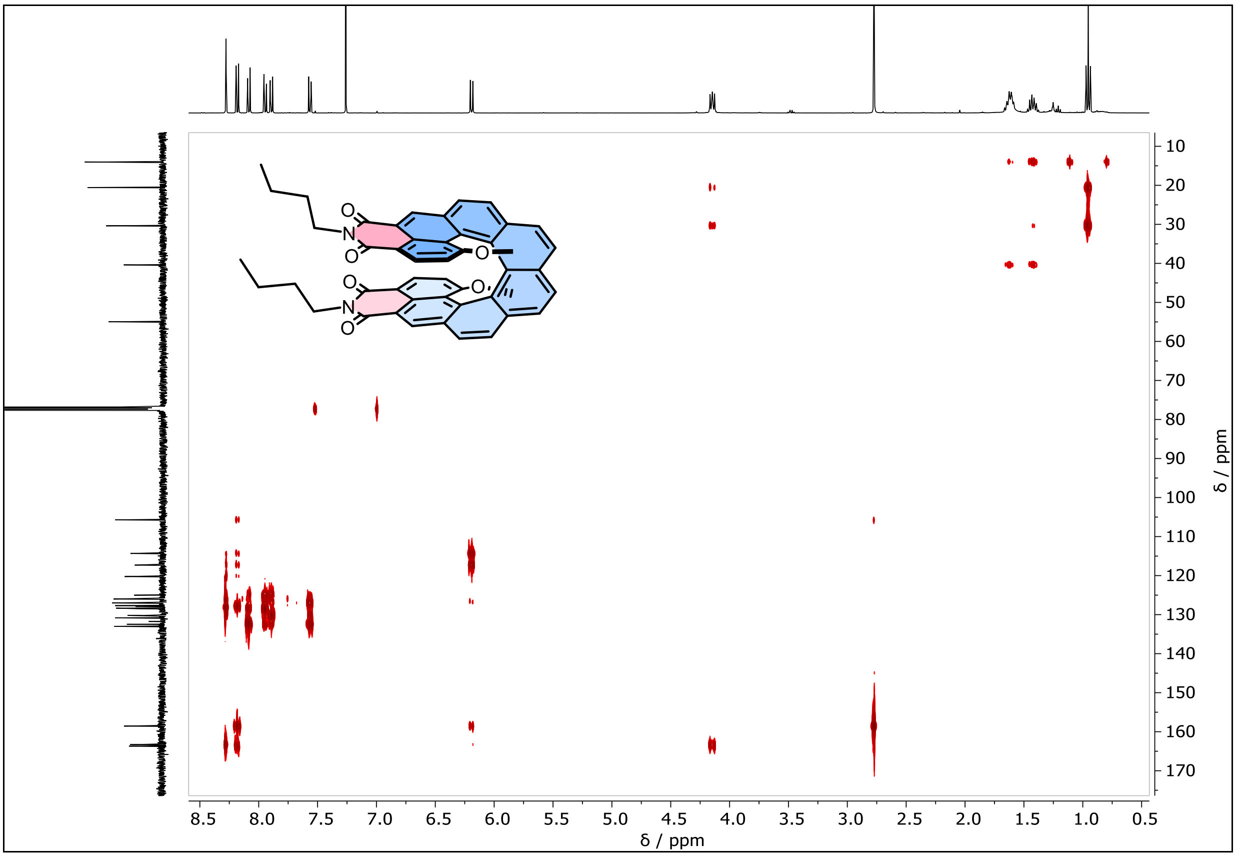

Figure S55. ^{1}H–^{13}C HMBC NMR spectrum of ***N*-*n*Bu[8]HDI-*f*-OMe** ($CDCl_3$, full spectrum).

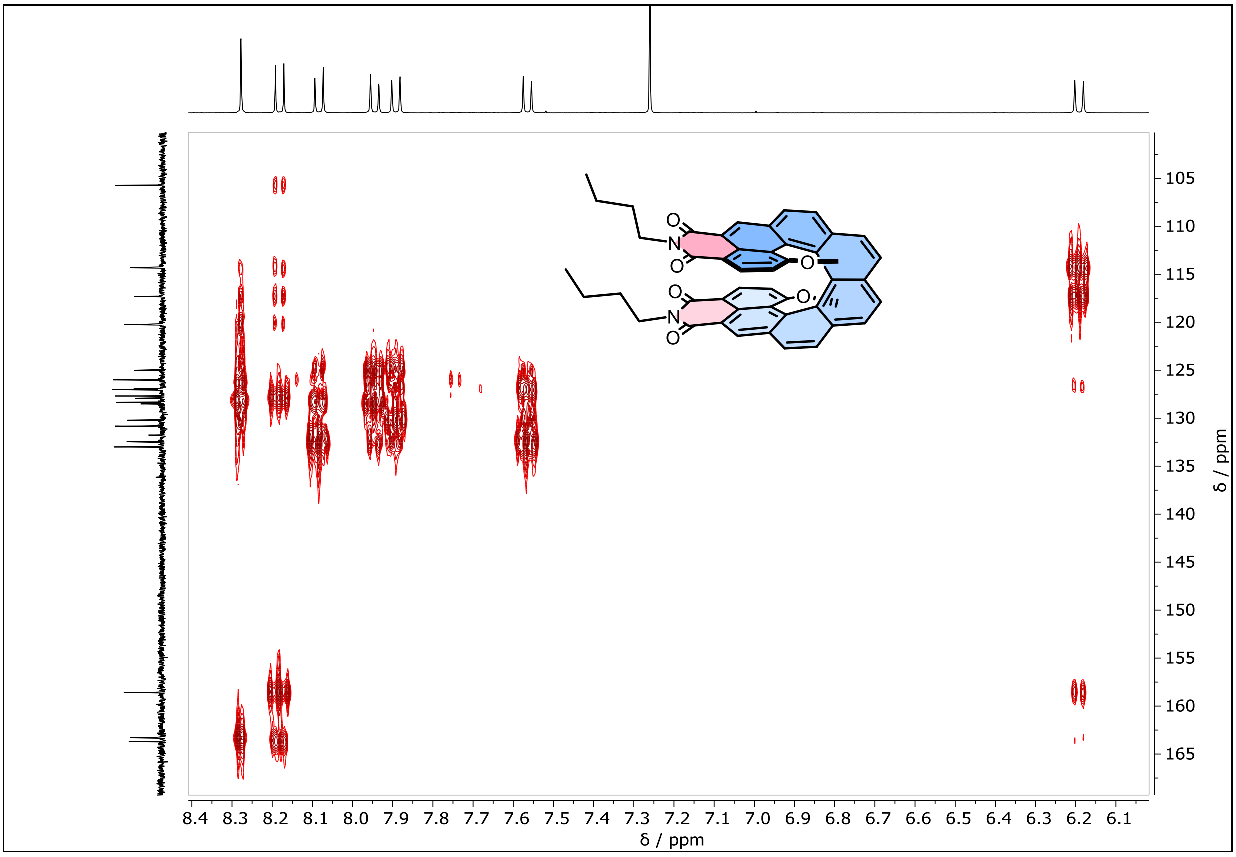

Figure S56. ^{1}H–^{13}C HMBC NMR spectrum ($CDCl_3$) of ***N*-*n*Bu[8]HDI-*f*-OMe** (aromatic section).

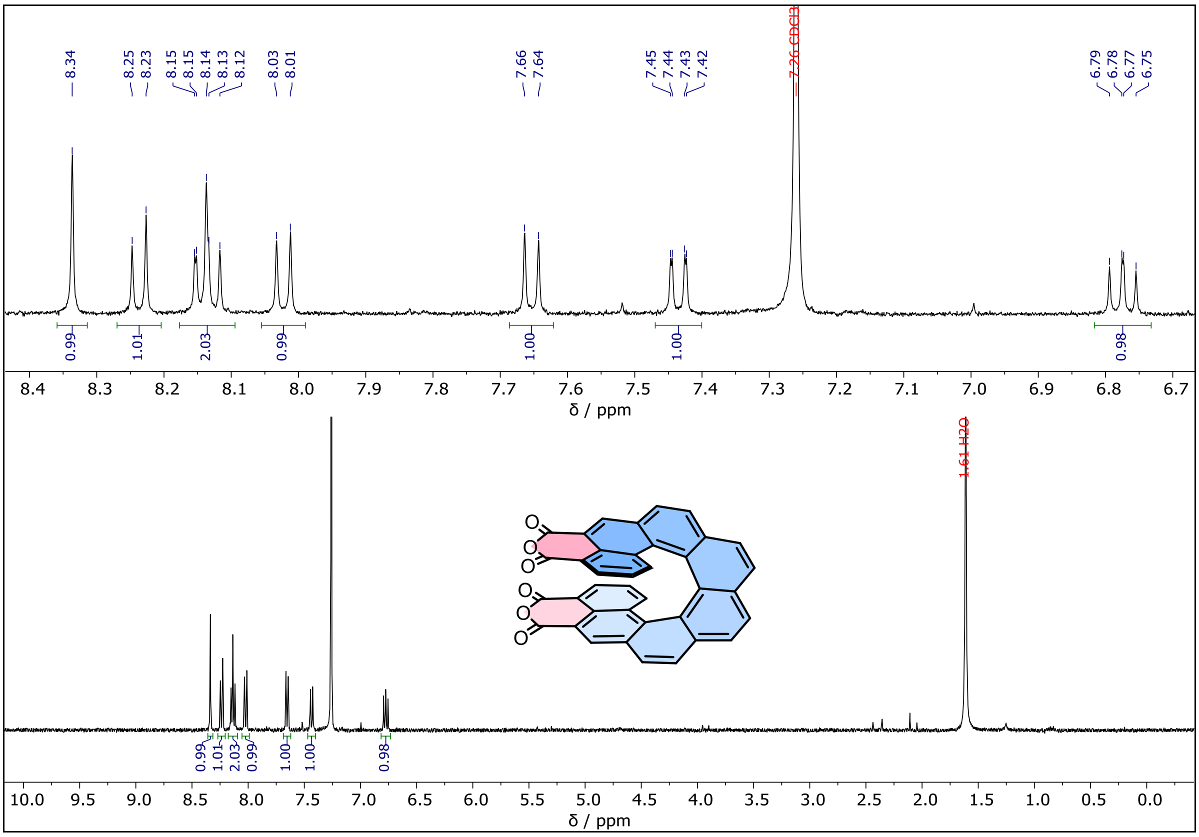

Figure S57. ^{1}H (400 MHz, $CDCl_3$) NMR spectrum of **7**.

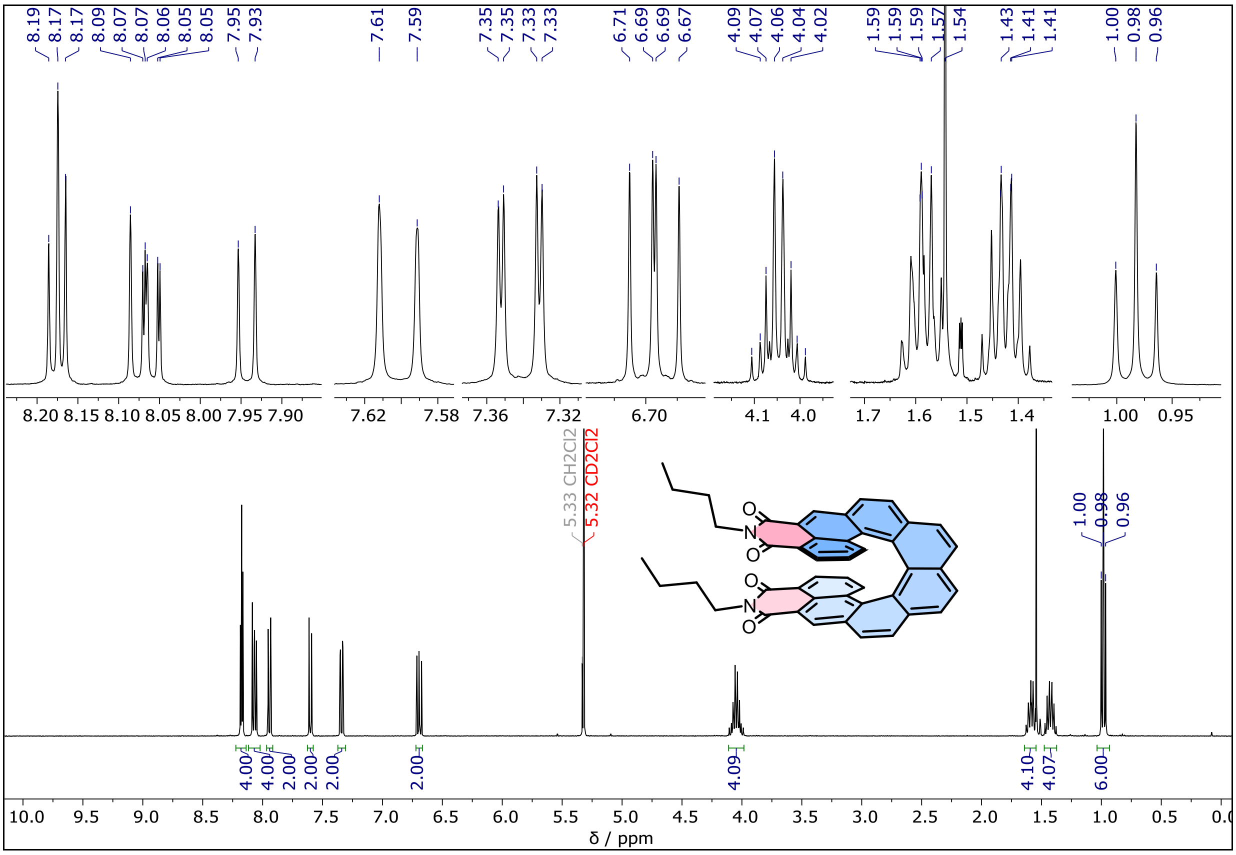

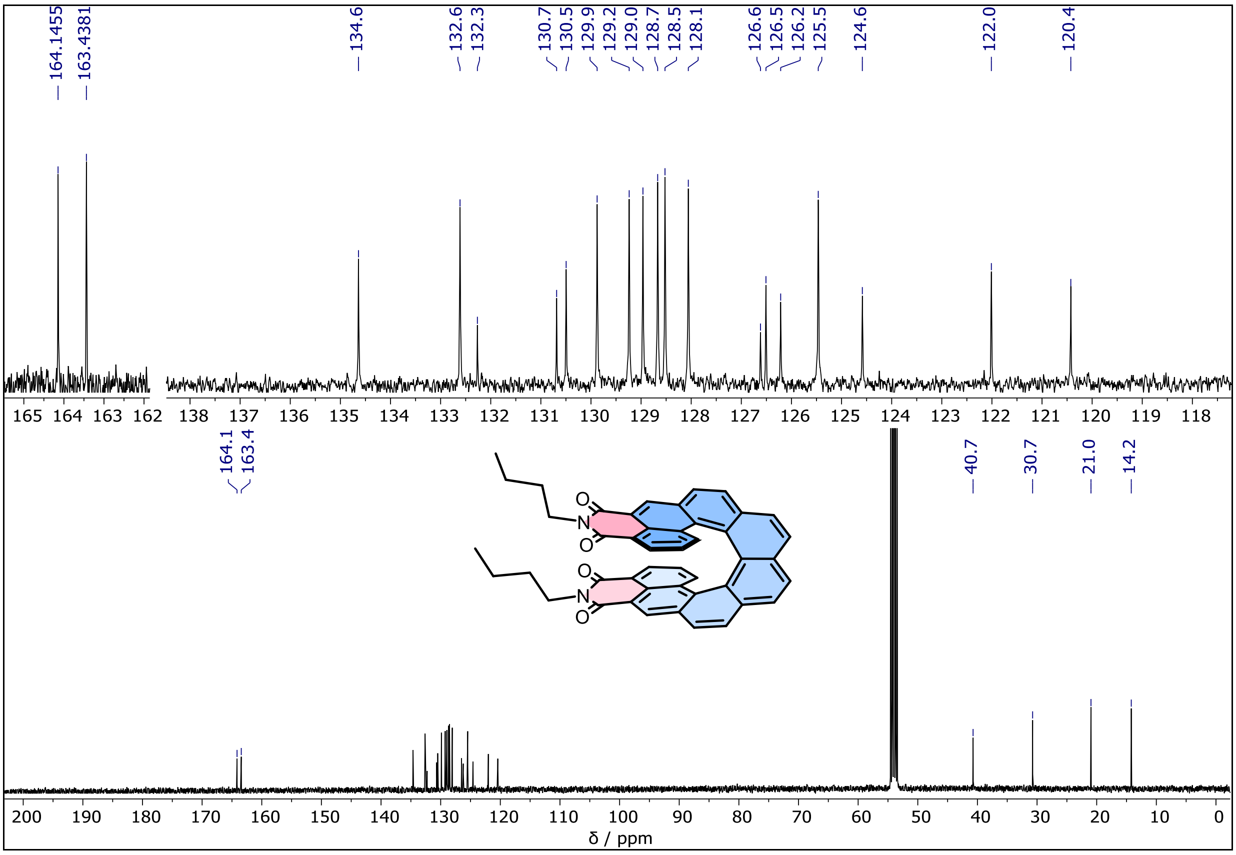

Figure S58. ^{1}H (top, 400 MHz, CD_2Cl_2) and $^{13}C\{^{1}H\}$ NMR spectra (bottom, 101 MHz, CD_2Cl_2) of ***N*-*n*Bu-[8]HDI**.

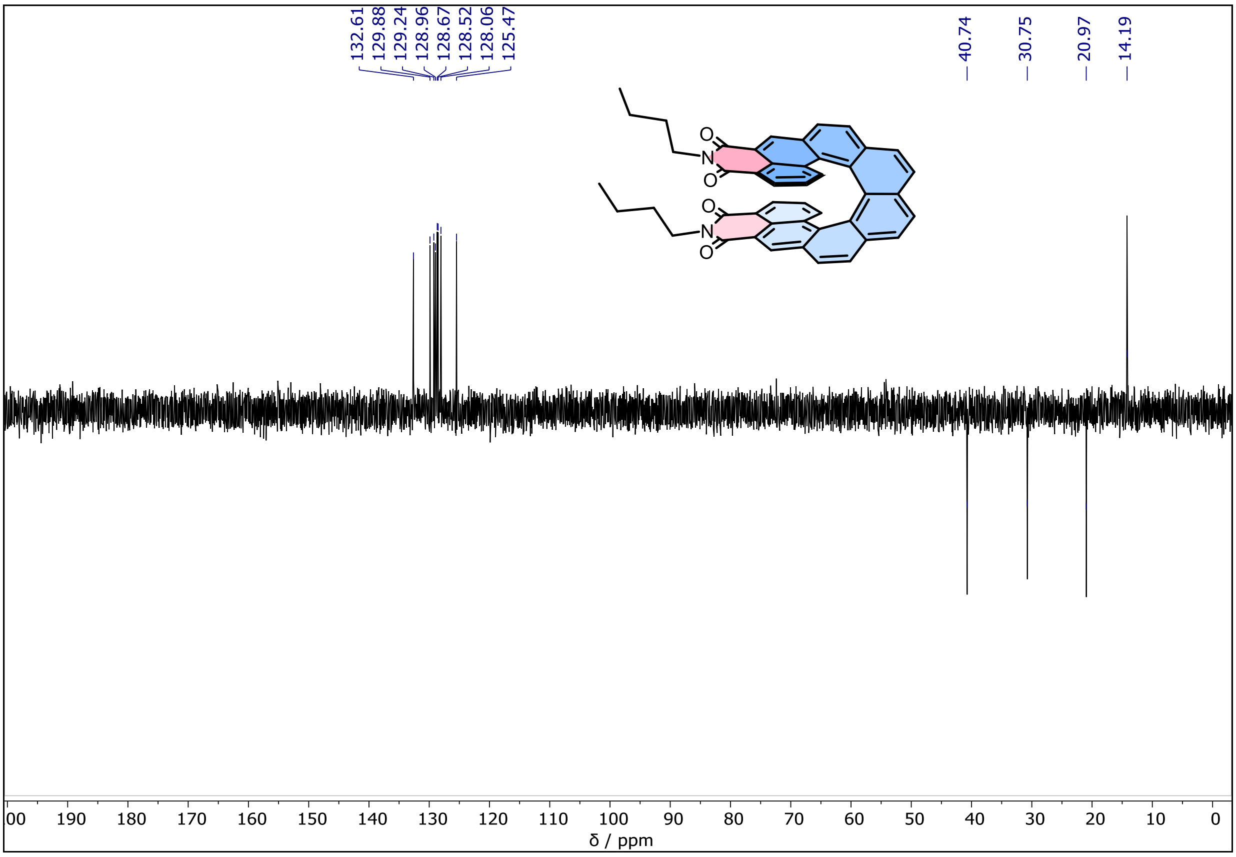

Figure S59. ^{13}C DEPT 135 NMR spectrum (101 MHz, CD_2Cl_2) of ***N*-*n*Bu-[8]HDI**.

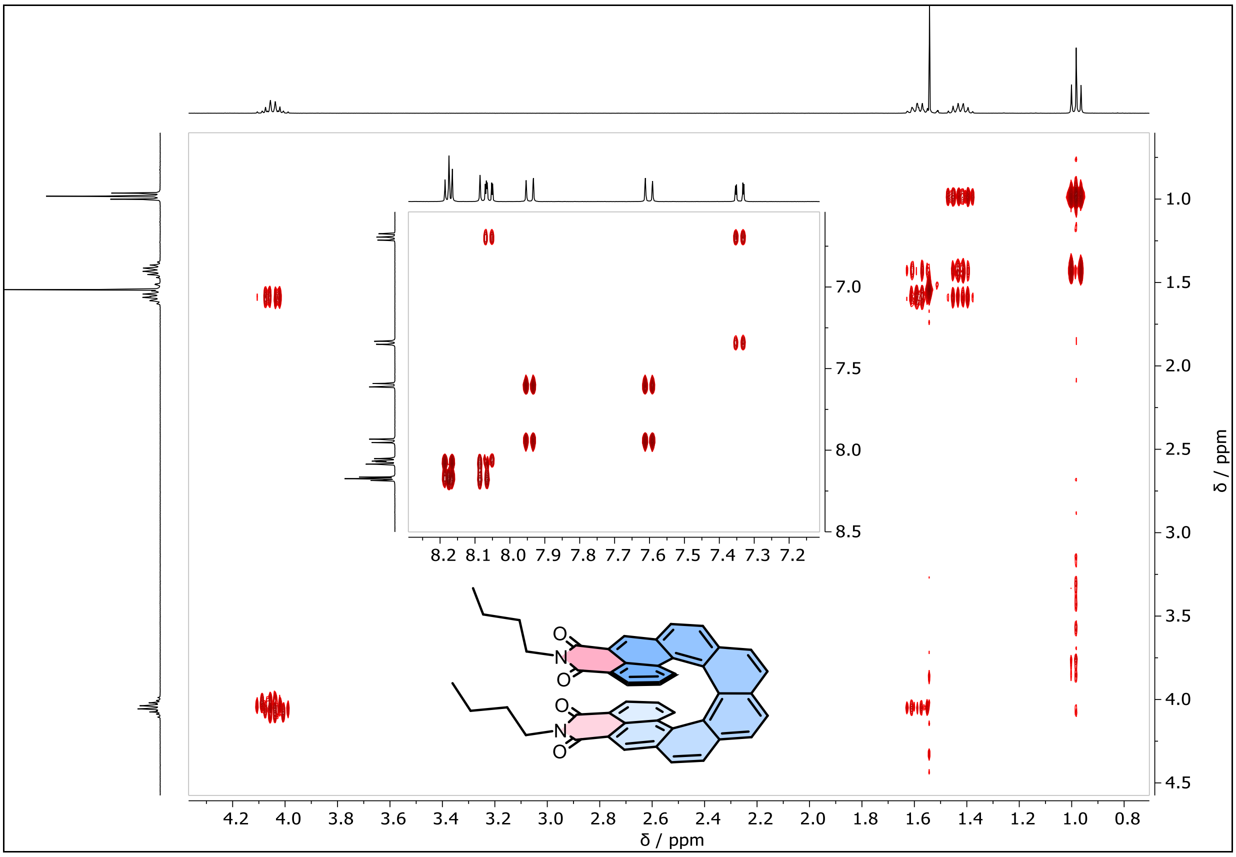

Figure S60. ^{1}H–^{1}H COSY NMR spectrum of ***N*-*n*Bu-[8]HDI** (400 MHz, CD_2Cl_2). Main spectrum: Aliphatic region. Insert: Aromatic region.

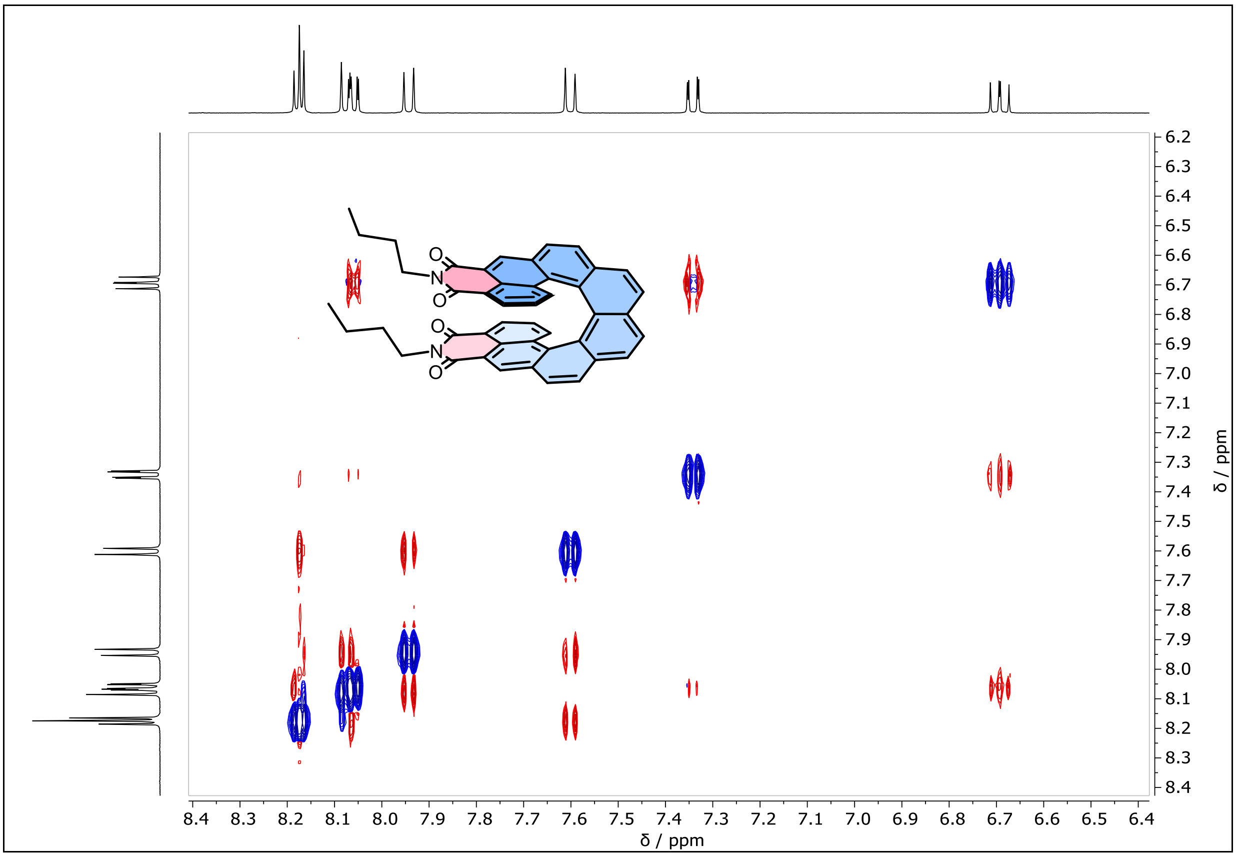

Figure S61. $^{1}H–^{1}H$ NOESY NMR spectrum (400 MHz, CD_2Cl_2) of ***N*-*n*Bu-[8]HDI**.

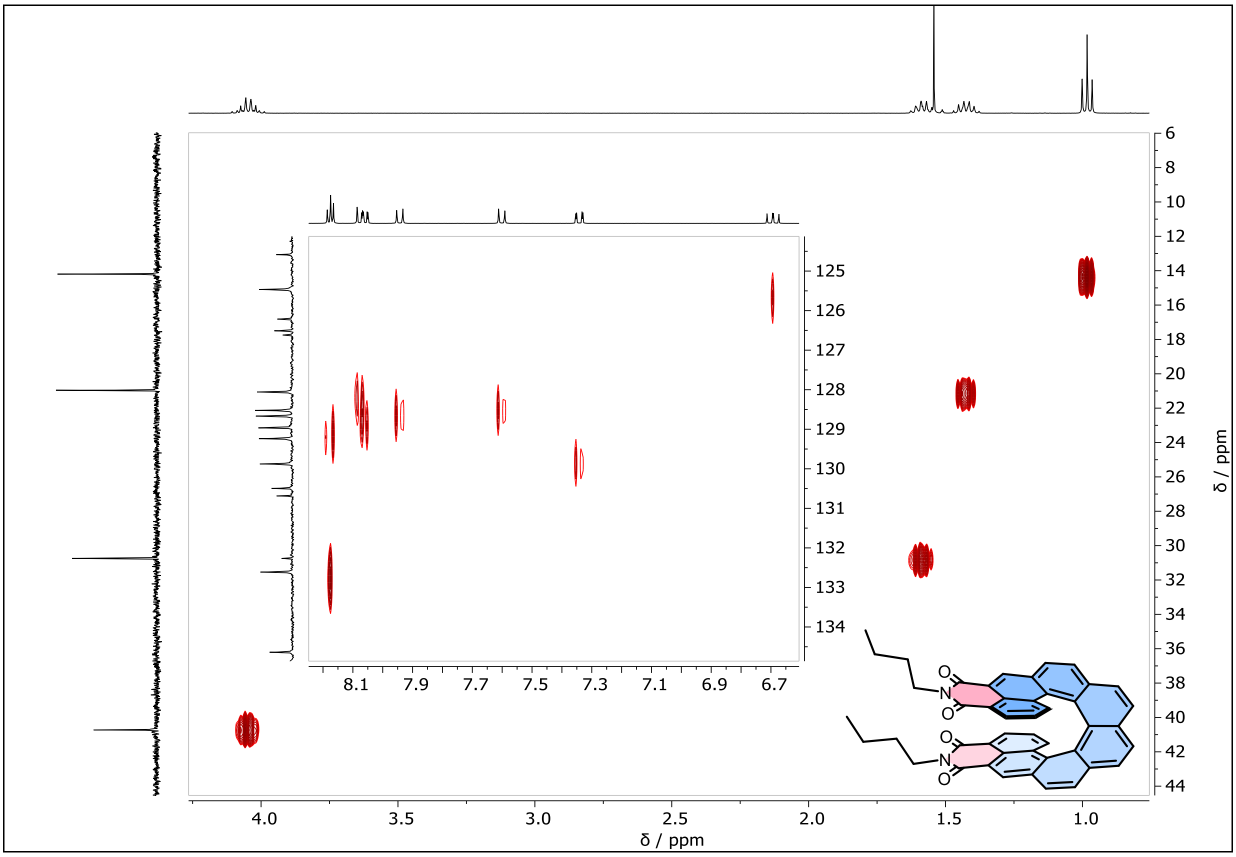

Figure S62. $^{1}H–^{1}H$ HSQC NMR spectrum (CD_2Cl_2) of ***N*-*n*Bu-[8]HDI**. Main spectrum: Aliphatic region. Insert: Aromatic region.

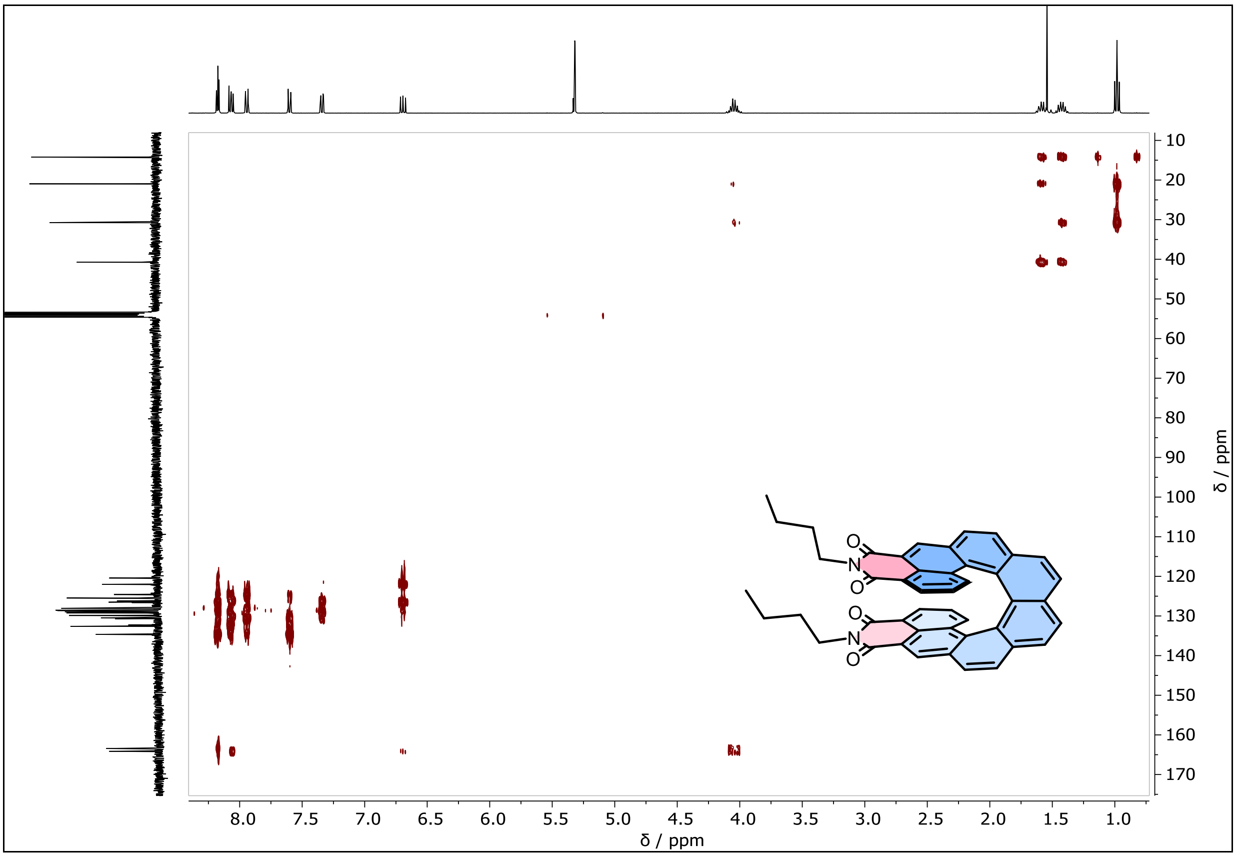

Figure S63. ^{1}H–^{13}C HMBC NMR spectrum of ***N-n*Bu[8]-HDI** (CD_2Cl_2, full spectrum).

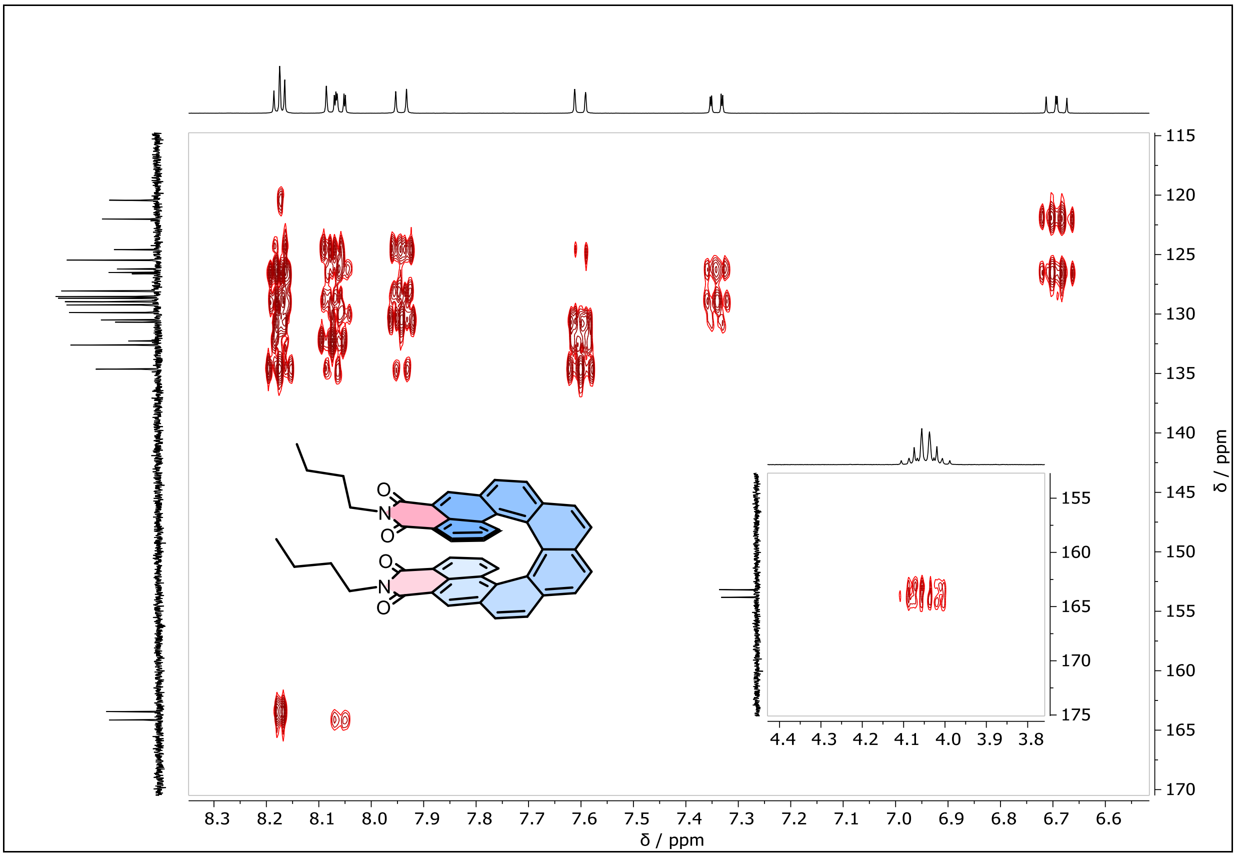

Figure S64. ^{1}H–^{13}C HMBC NMR spectrum of ***N-n*Bu[8]-HDI** (CD_2Cl_2, aromatic section).

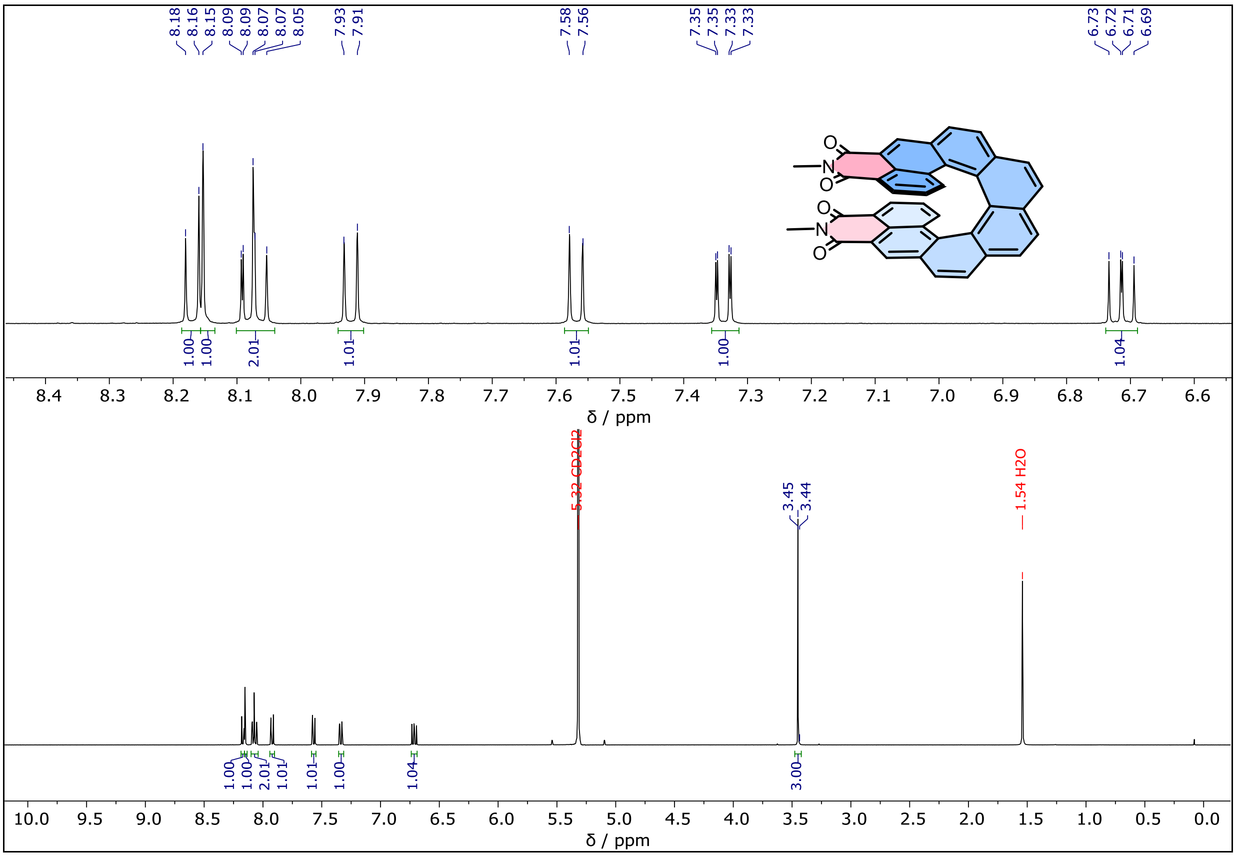

Figure S65. ^{1}H (400 MHz, CD_2Cl_2) NMR spectrum of ***N*-Me-[8]HDI**.

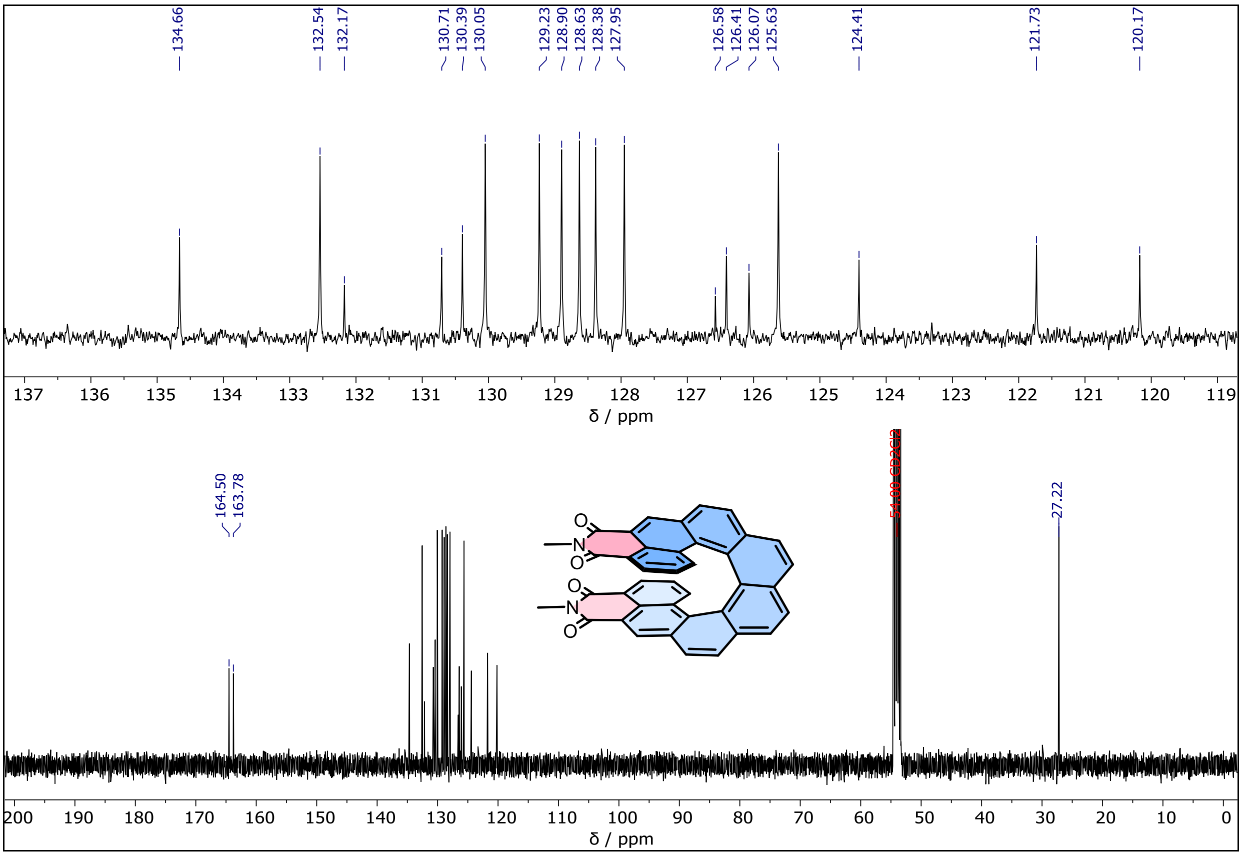

Figure S66.^{13}C{^{1}H} NMR spectrum (101 MHz, CD_2Cl_2) of ***N*-Me-8HDI**.

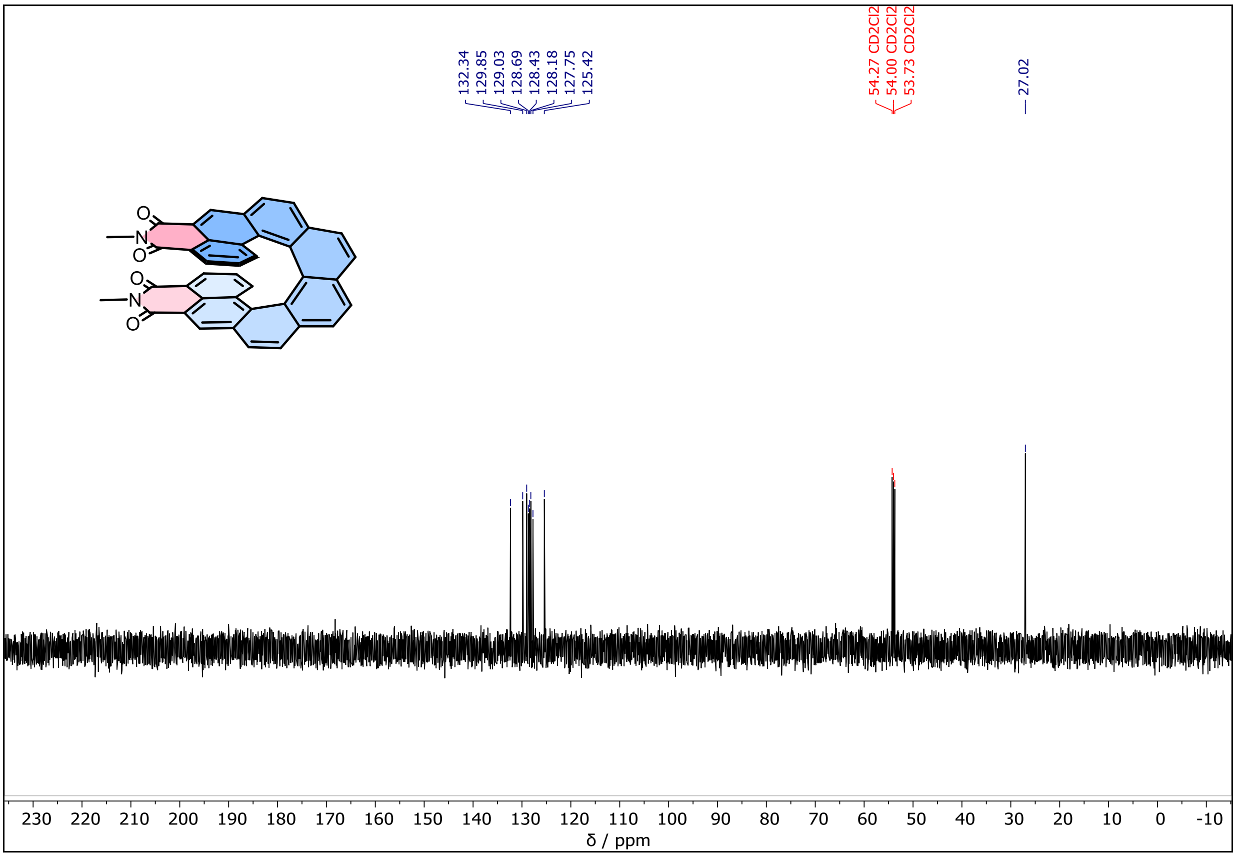

Figure S67. ^{13}C DEPT 135 NMR spectrum (101 MHz, CD_2Cl_2) of ***N*-Me-8HDI**.

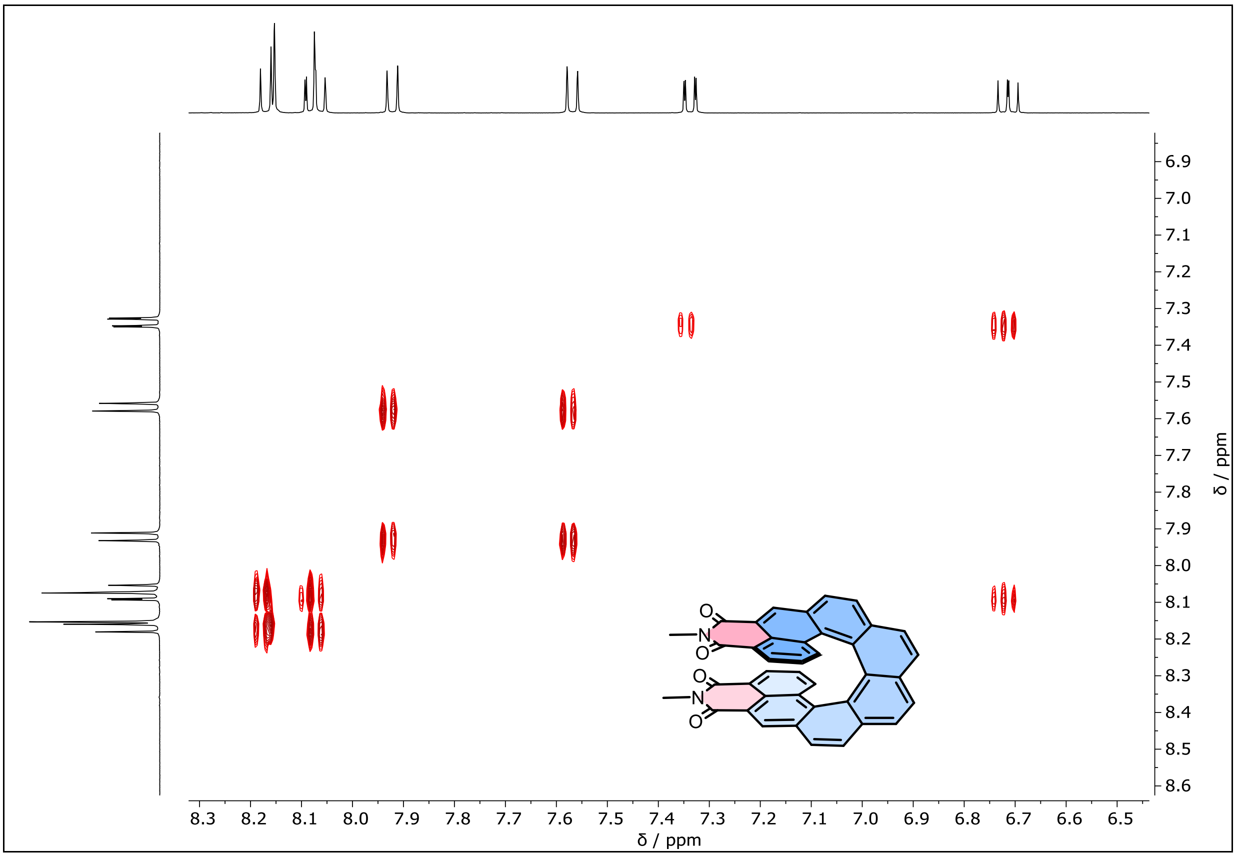

Figure S68. ^{1}H–^{1}H COSY NMR spectrum (400 MHZ, CD_2Cl_2) of ***N*-Me-8HDI** (aromatic section).

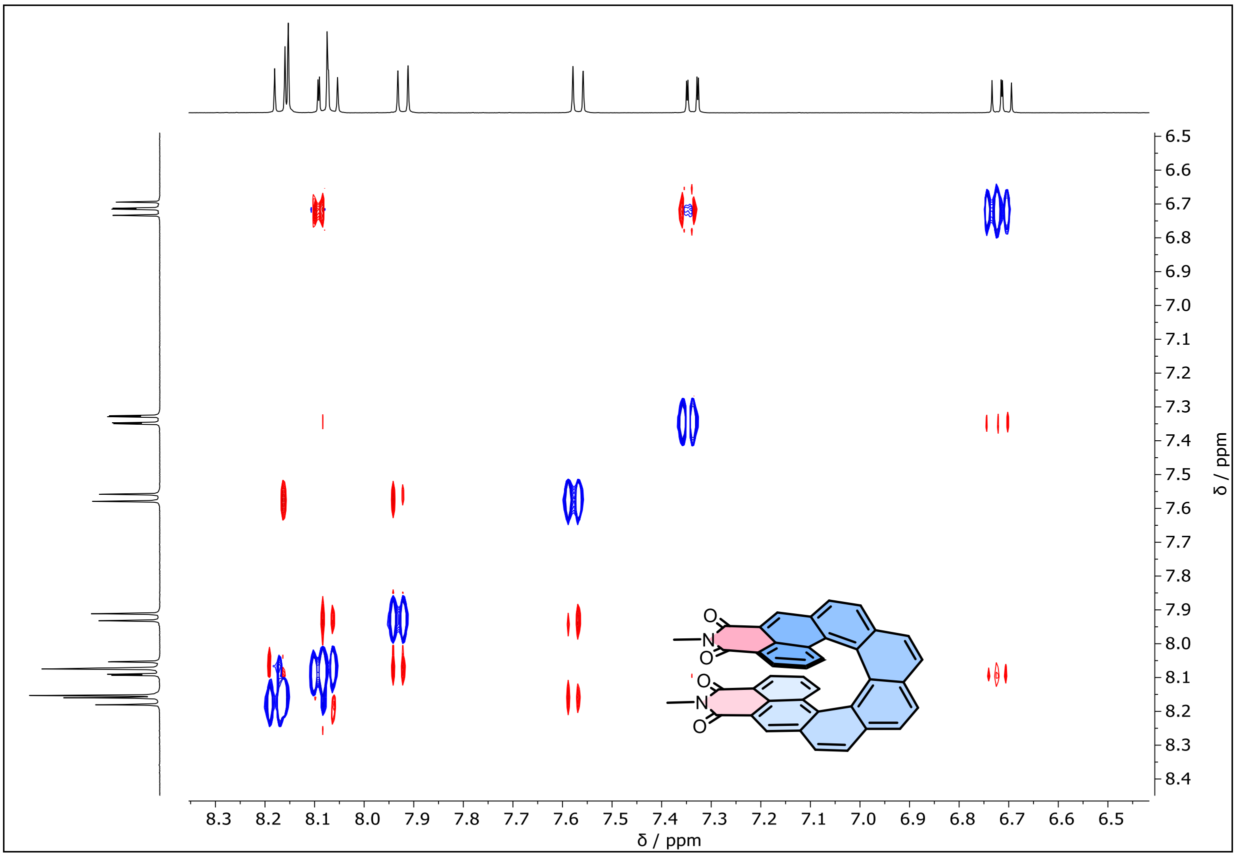

Figure S69. ^{1}H–^{1}H NOESY NMR spectrum (400 MHz, CD_2Cl_2) of ***N*-Me-8HDI.**

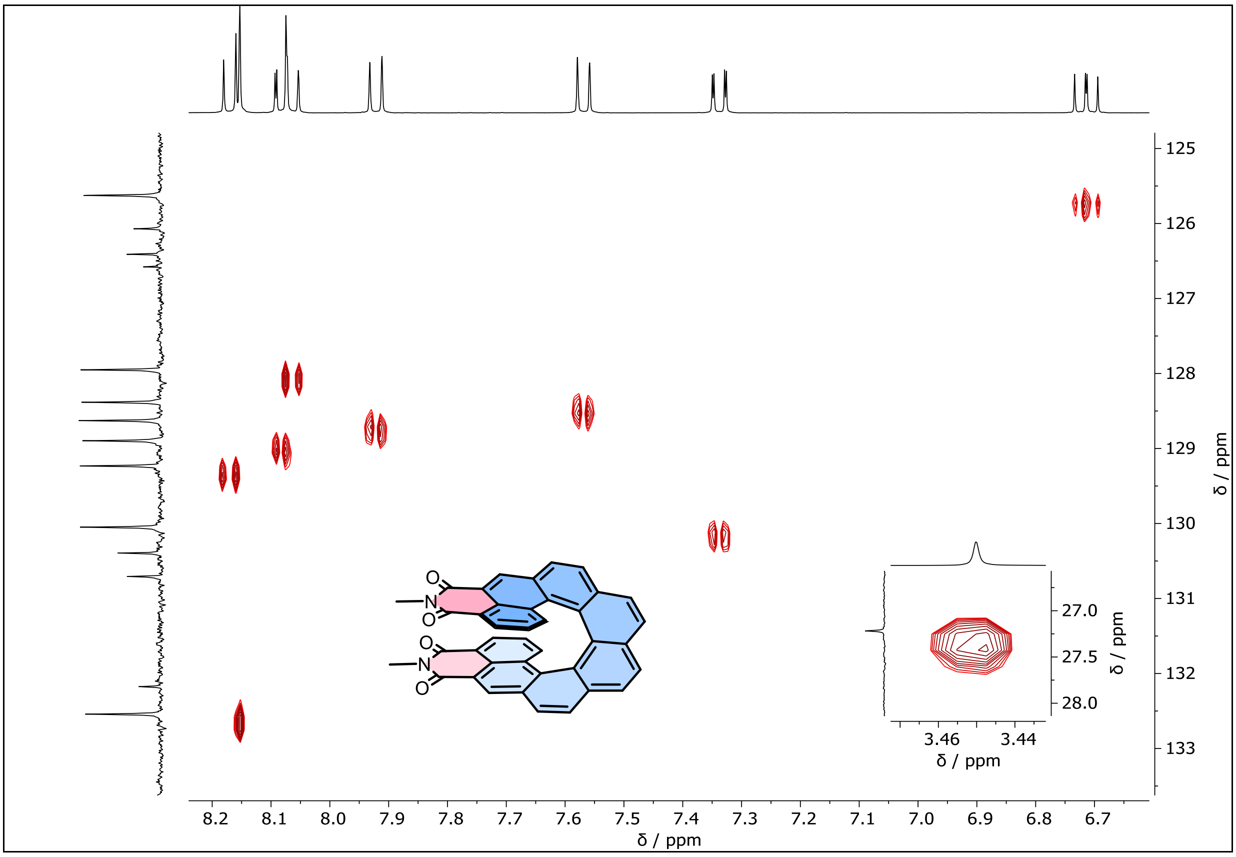

Figure S70. ^{1}H–^{13}C HSQC NMR spectrum (CD_2Cl_2) of ***N*-Me-8HDI**.

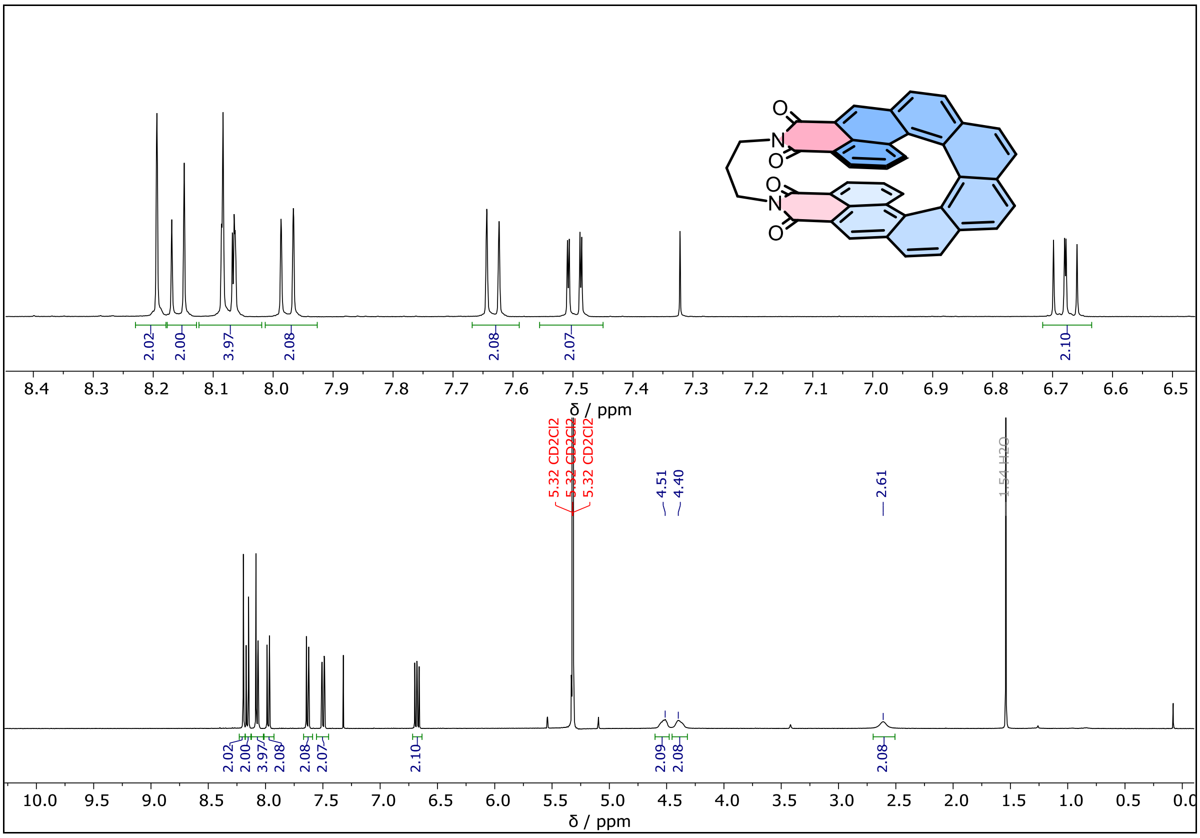

Figure S71. ^{1}H (400 MHz, CD_2Cl_2) NMR spectrum of **C3-8HDI**.

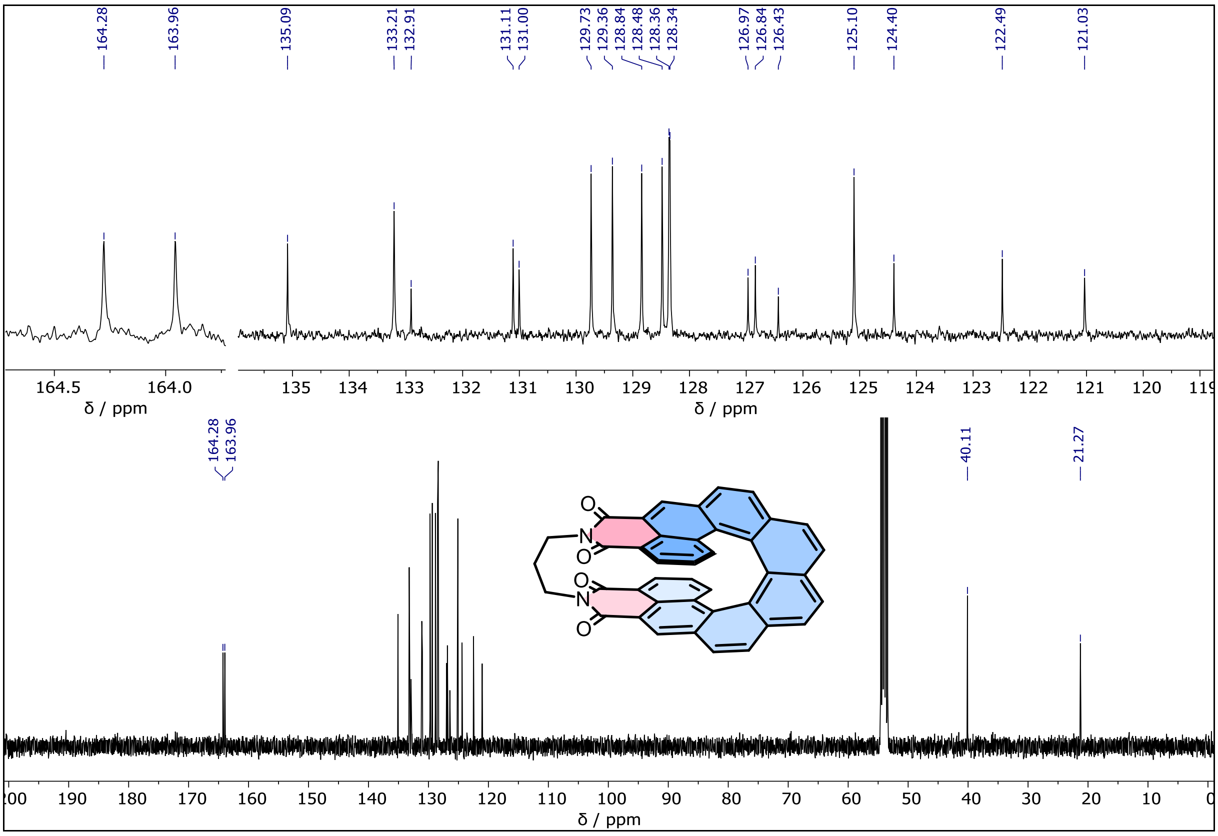

Figure S72. $^{13}C\{^{1}H\}$ NMR spectrum (101 MHz, CD_2Cl_2) of **C3-8HDI**

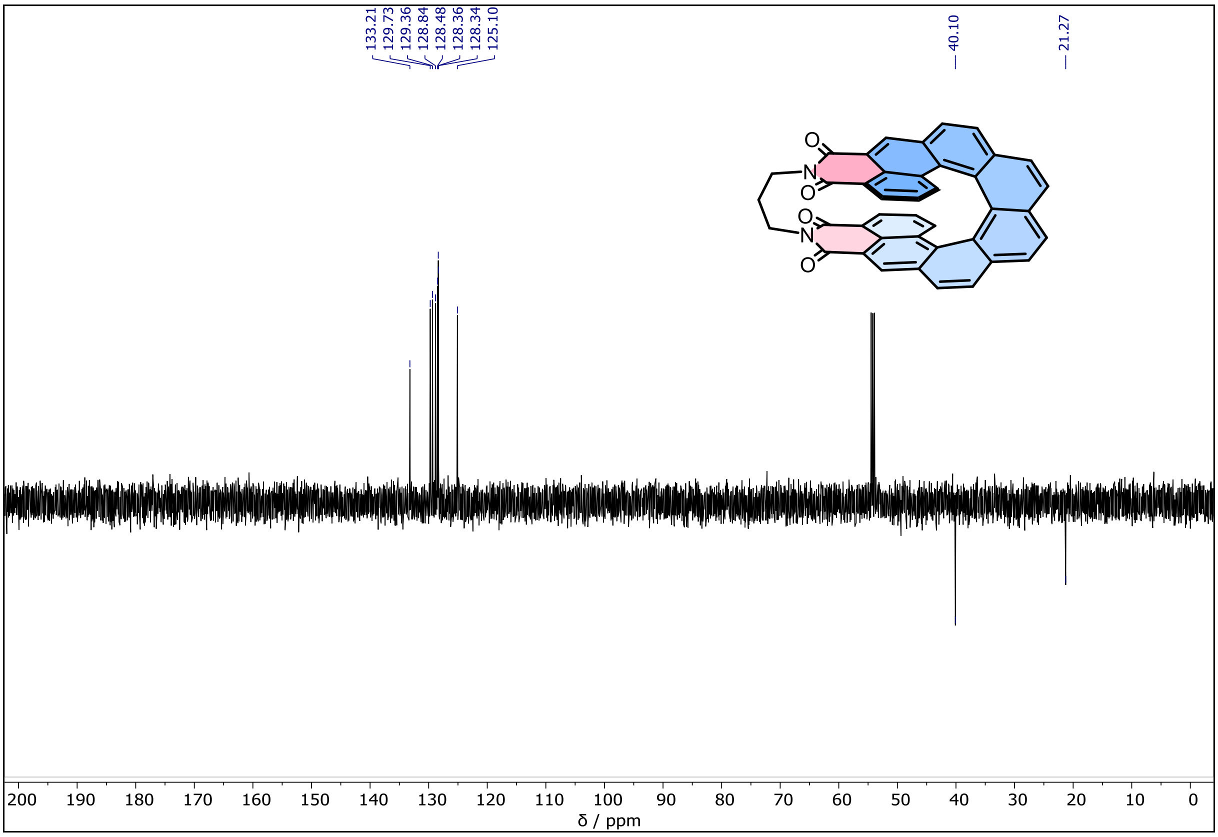

Figure S73. ^{13}C DEPT 135 NMR spectrum (101 MHz, CD_2Cl_2) of **C_3-8HDI**.

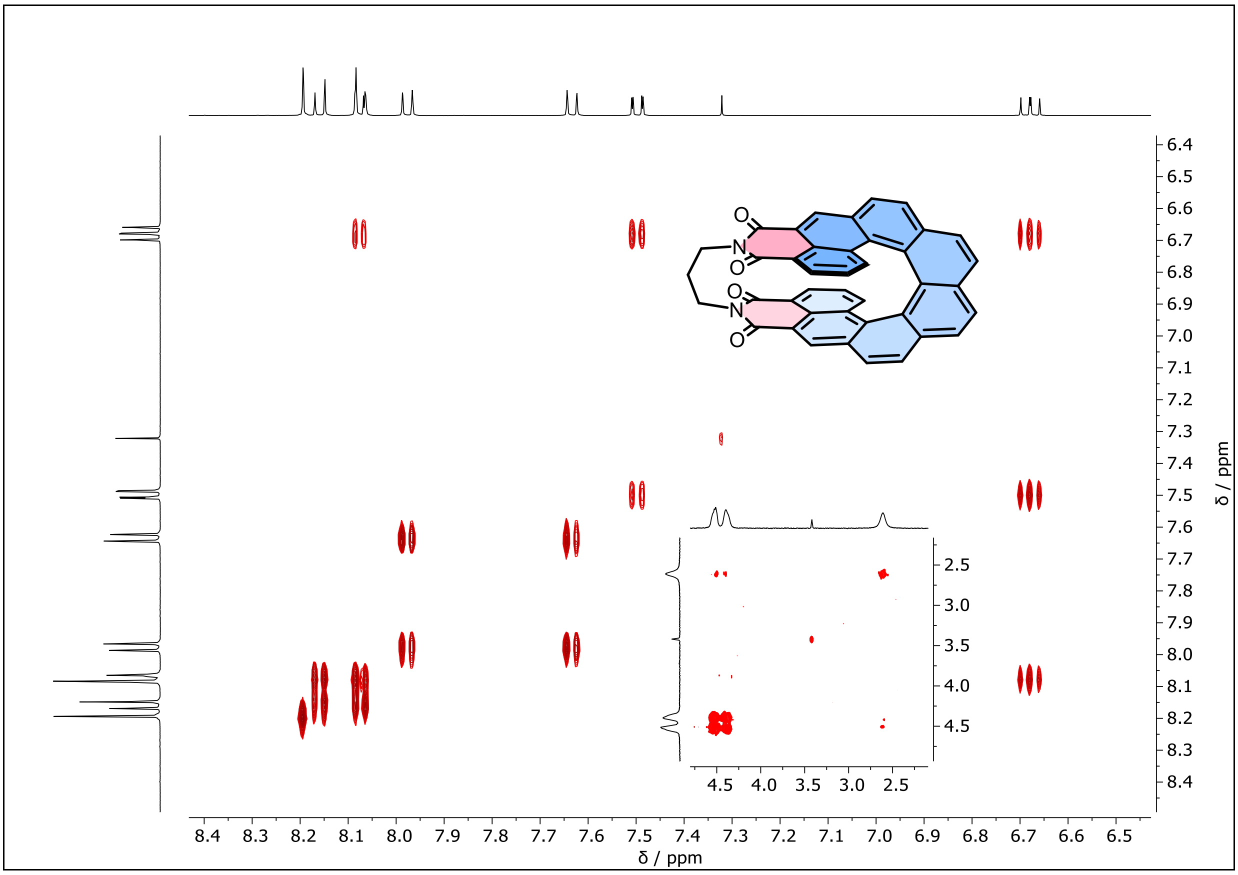

Figure S74. ^{1}H–^{1}H COSY NMR spectrum (101 MHz, CD_2Cl_2) of **C_3-8HDI**.

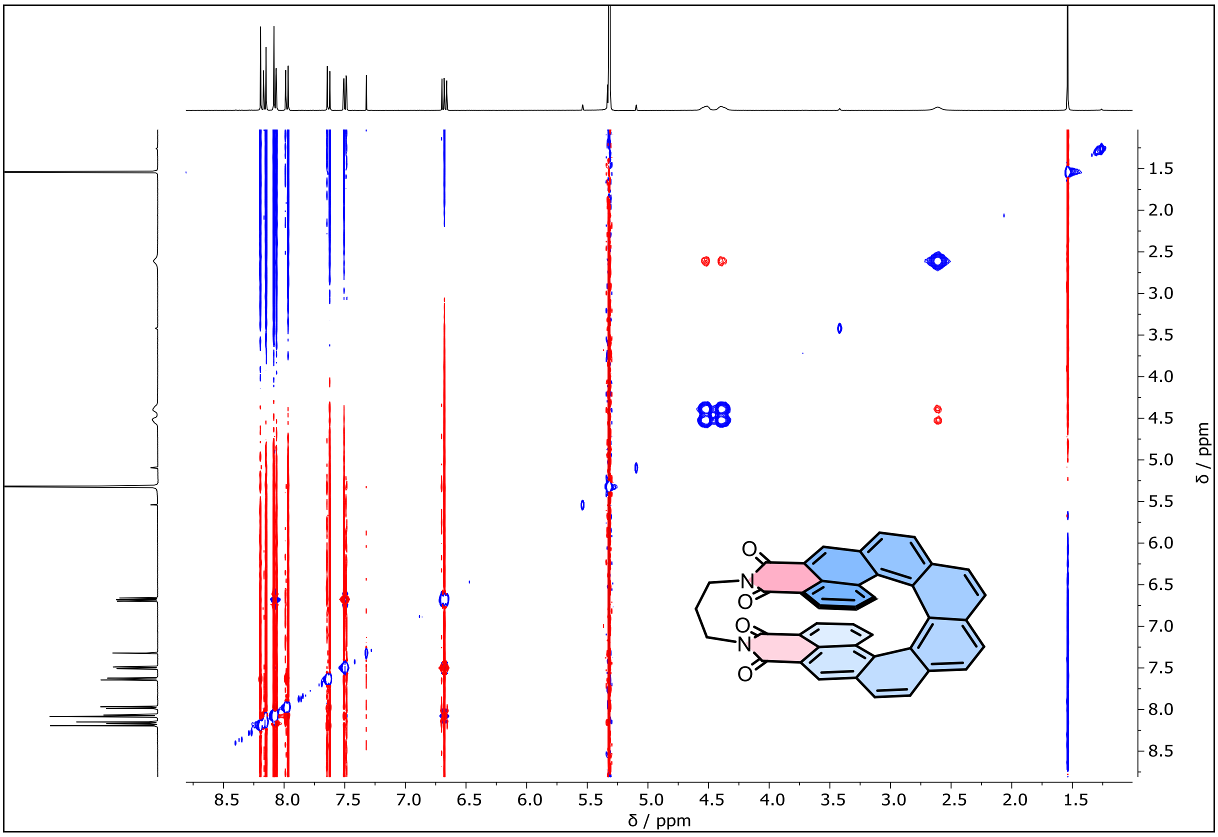

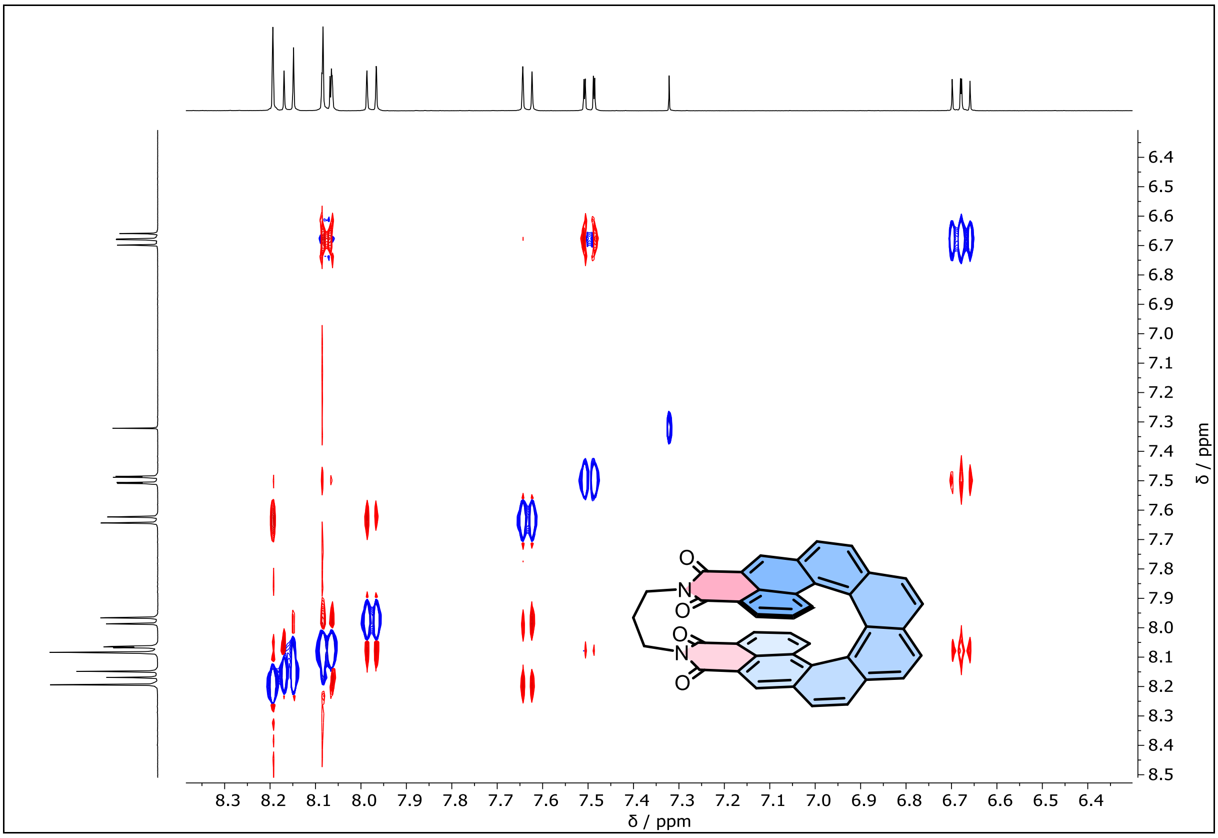

Figure S75. ^{1}H–^{1}H NOESY NMR spectrum (101 MHz, CD_2Cl_2) of **C_3-8HDI** (top), magnified aromatic section (bottom)

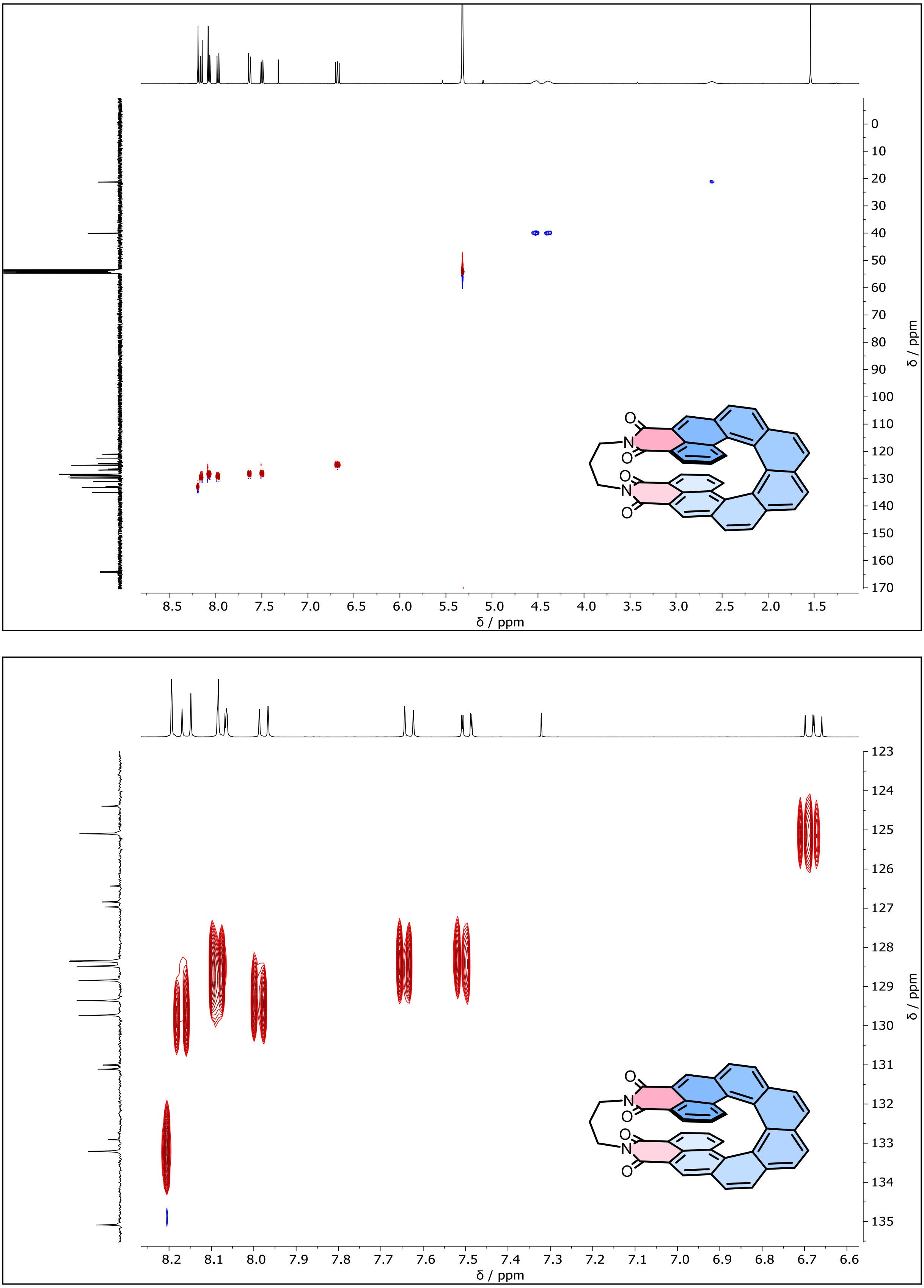

Figure S76. ^{1}H–^{13}C HSQC NMR spectrum (CD_2Cl_2) of **C_3-8HDI** (top), magnified aromatic section (bottom).

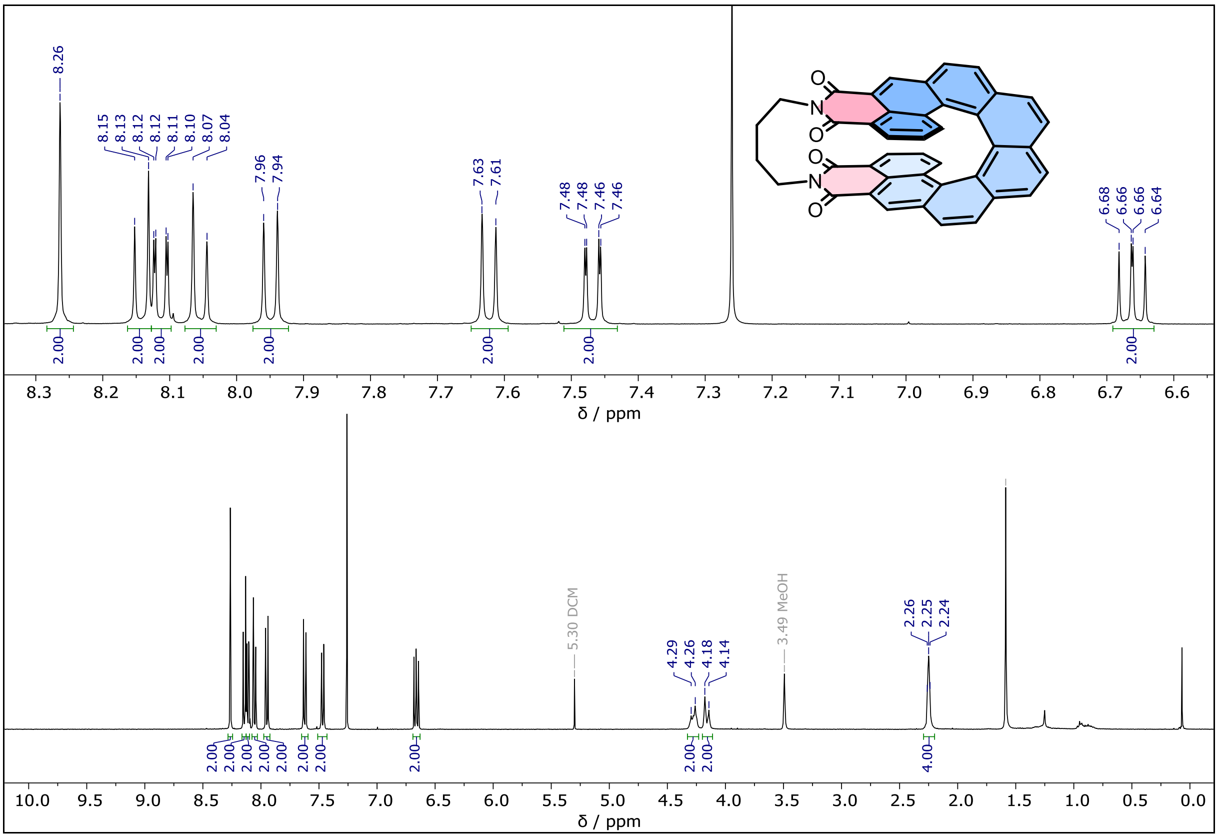

Figure S77. ^{1}H (400 MHz, $CDCl_3$) NMR spectrum of **C4-[8]HDI**.

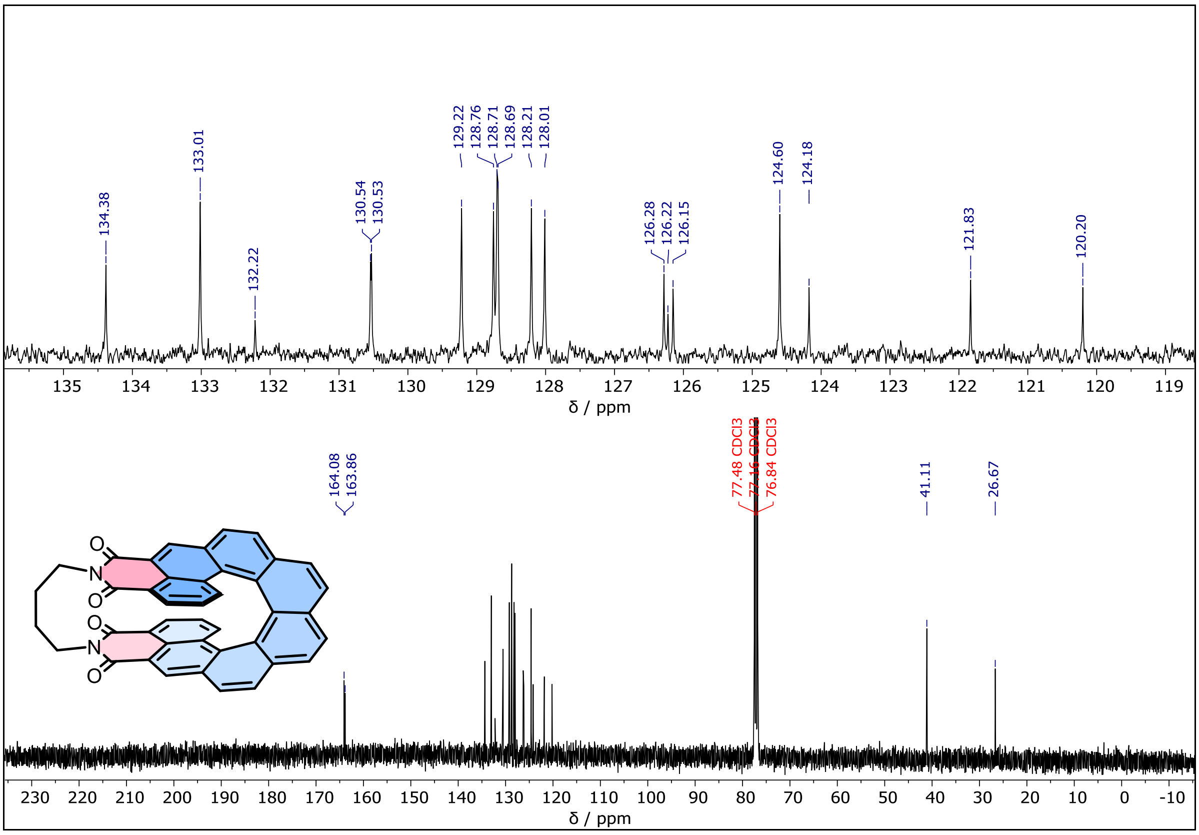

Figure S78. ^{13}C{^{1}H} NMR spectrum (101 MHz, $CDCl_3$) of **C4-[8]HDI**.

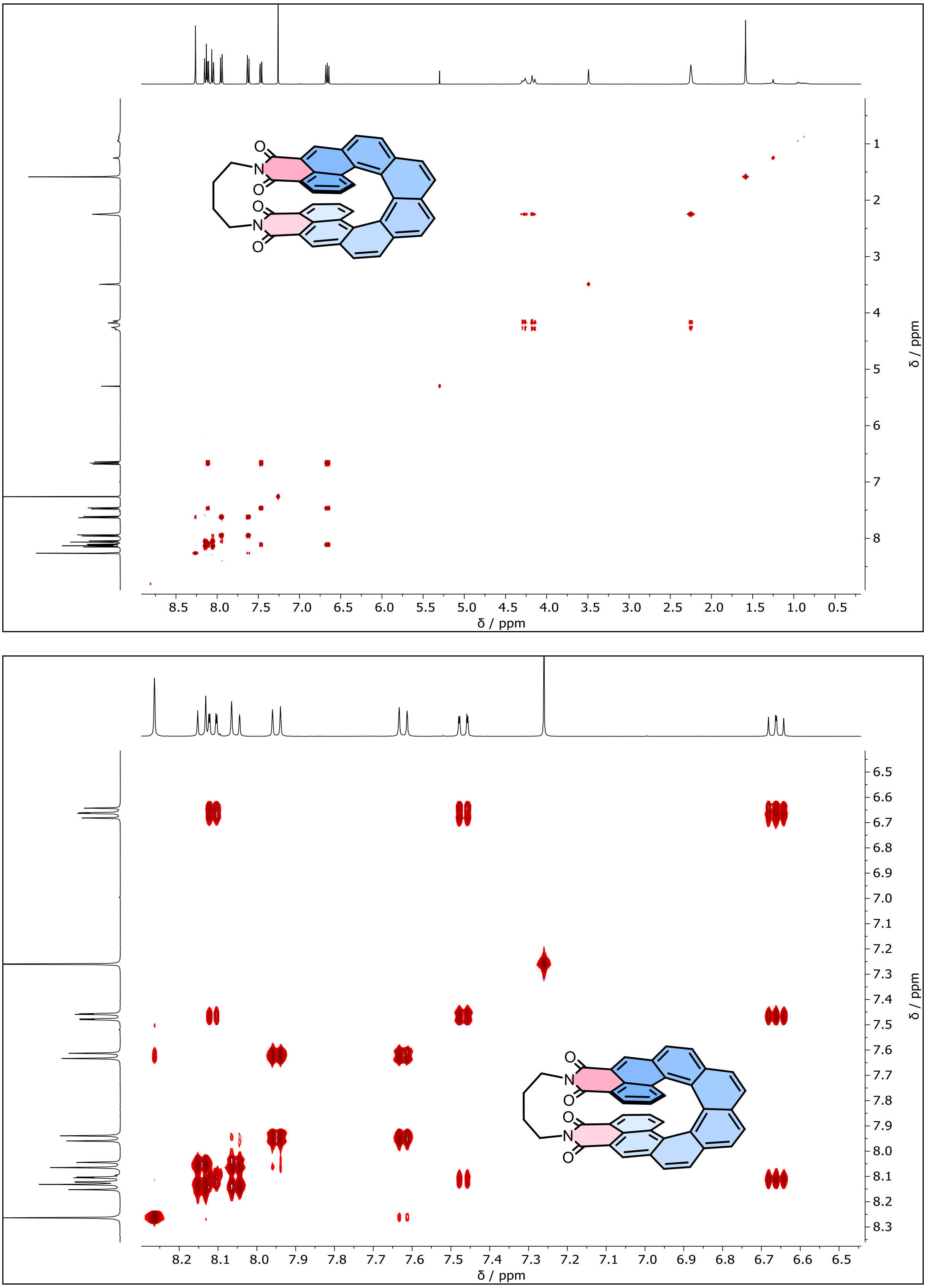

Figure S79. ^1H–^1H COSY NMR spectrum (400 MHz, $CDCl_3$) of **C_4-[8]HDI** (top), magnified aromatic section (bottom).

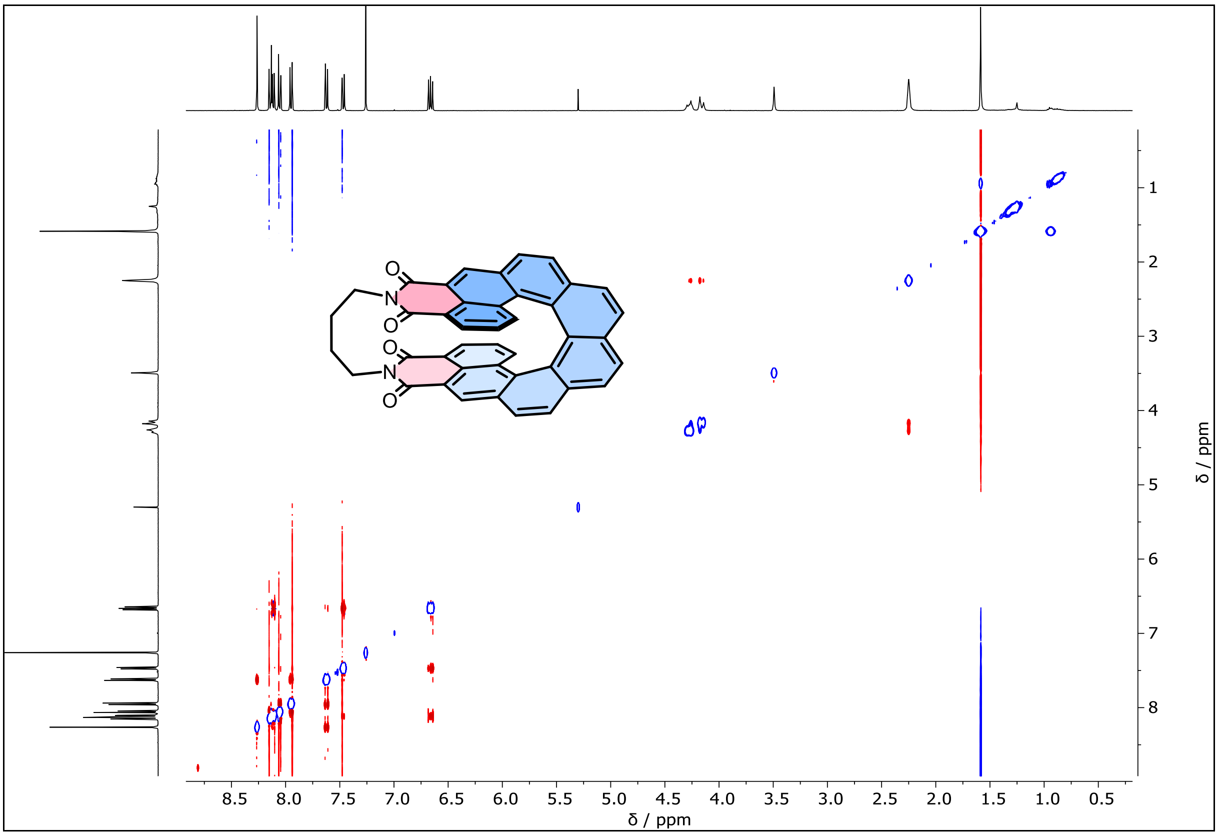

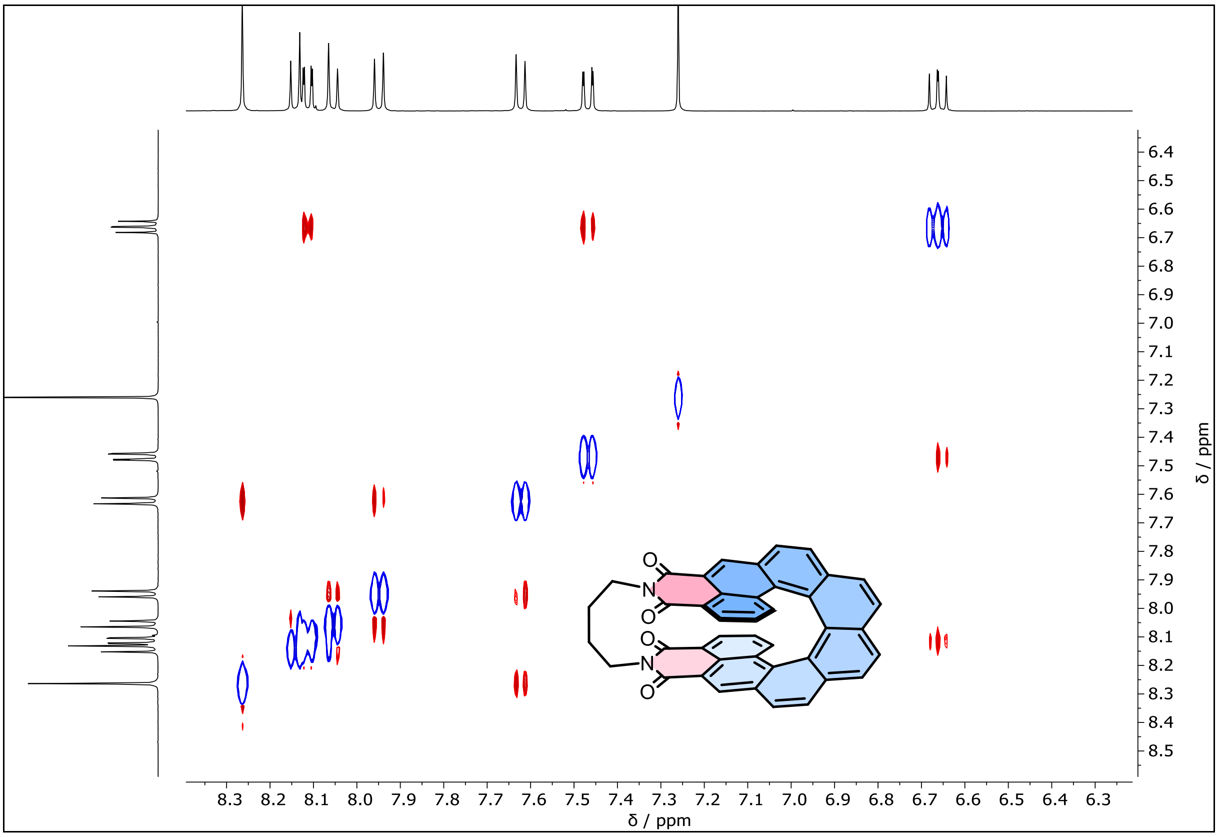

Figure S80. ^{1}H–^{1}H NOESY NMR spectrum (400 MHz, $CDCl_3$) of **C_4-8HDI** (top), magnified aromatic section (bottom).

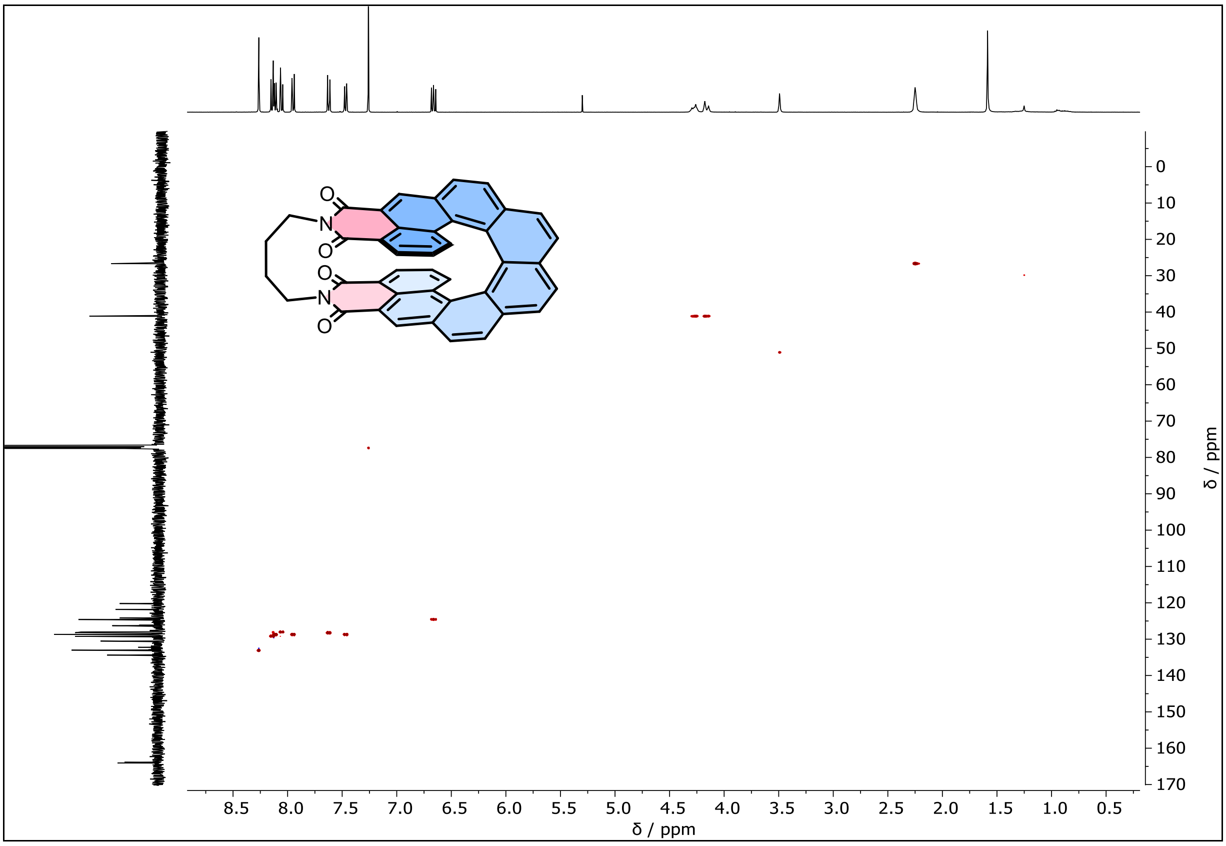

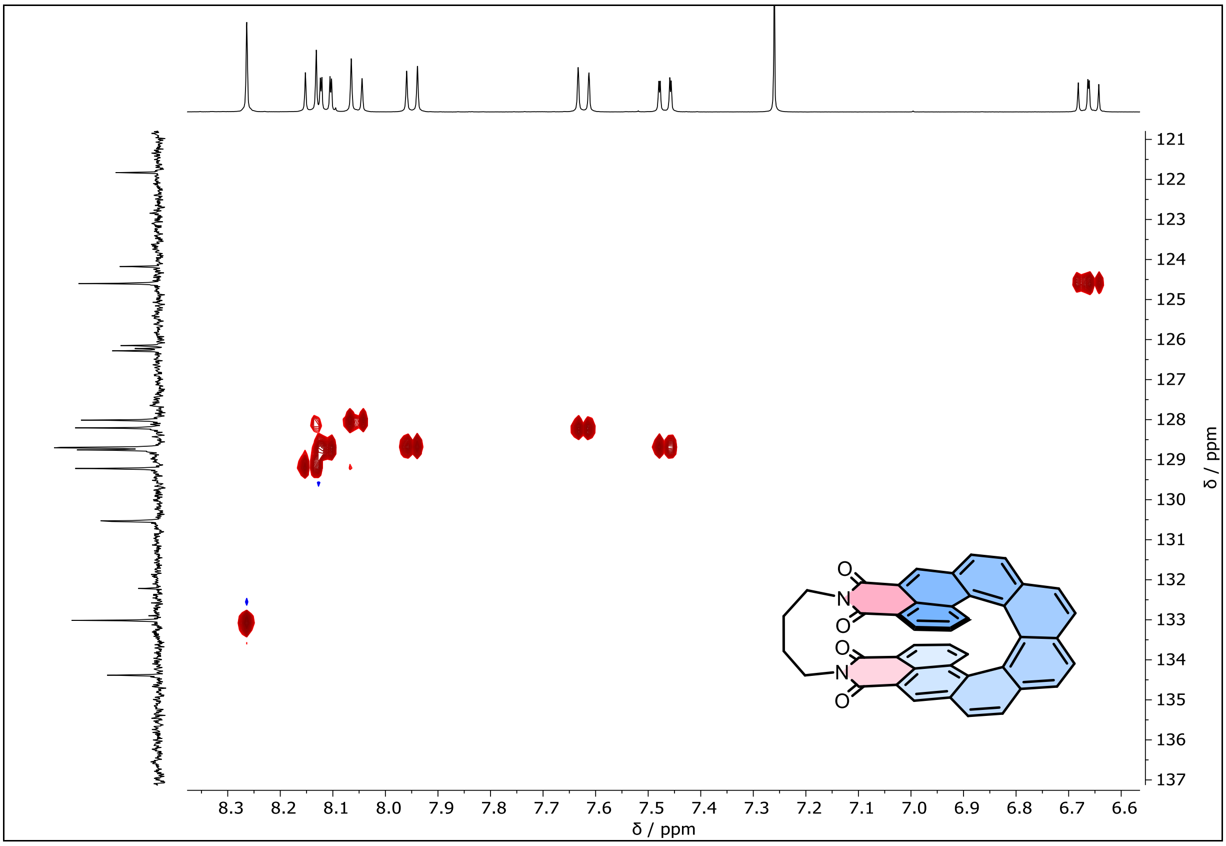

Figure S81. ^{1}H–^{13}C HSQC NMR spectrum ($CDCl_3$) of **C4-[8]HDI** (top), magnified aromatic section (bottom).

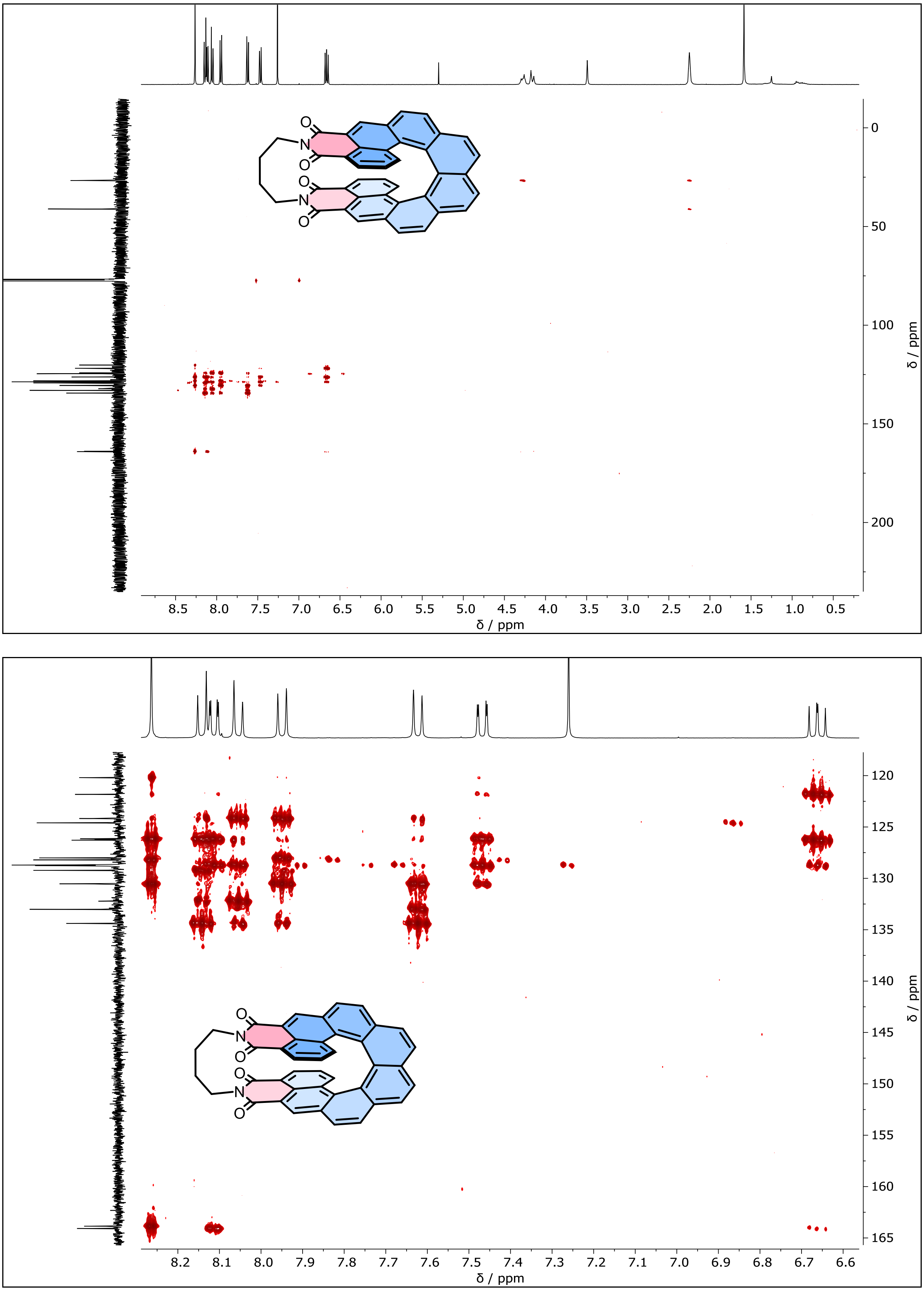

Figure S82. ^{1}H–^{13}C HMBC NMR spectrum ($CDCl_3$) of **C_4-[8]HDI**, magnified aromatic section (bottom).

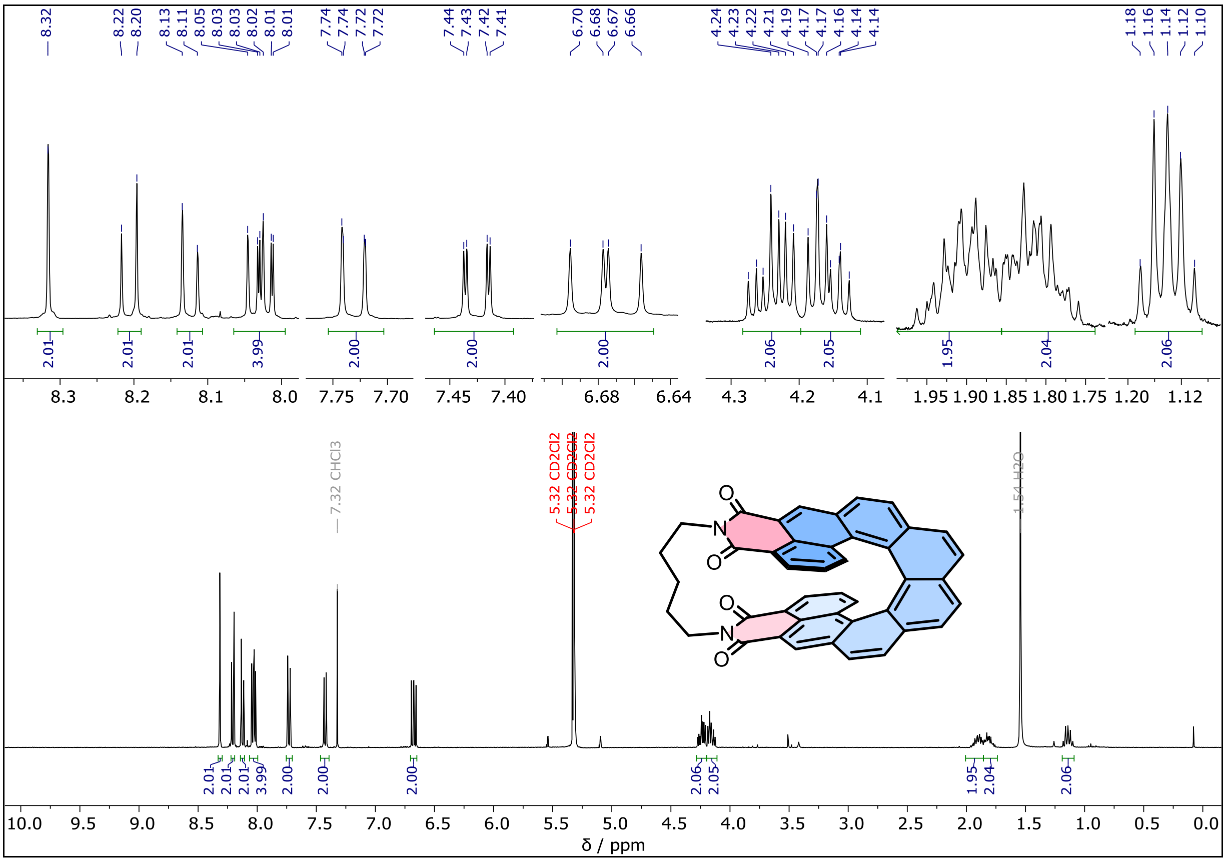

Figure S83. ^{1}H (400 MHz, CD_2Cl_2) NMR spectrum of **C₅-[8]HDI**.

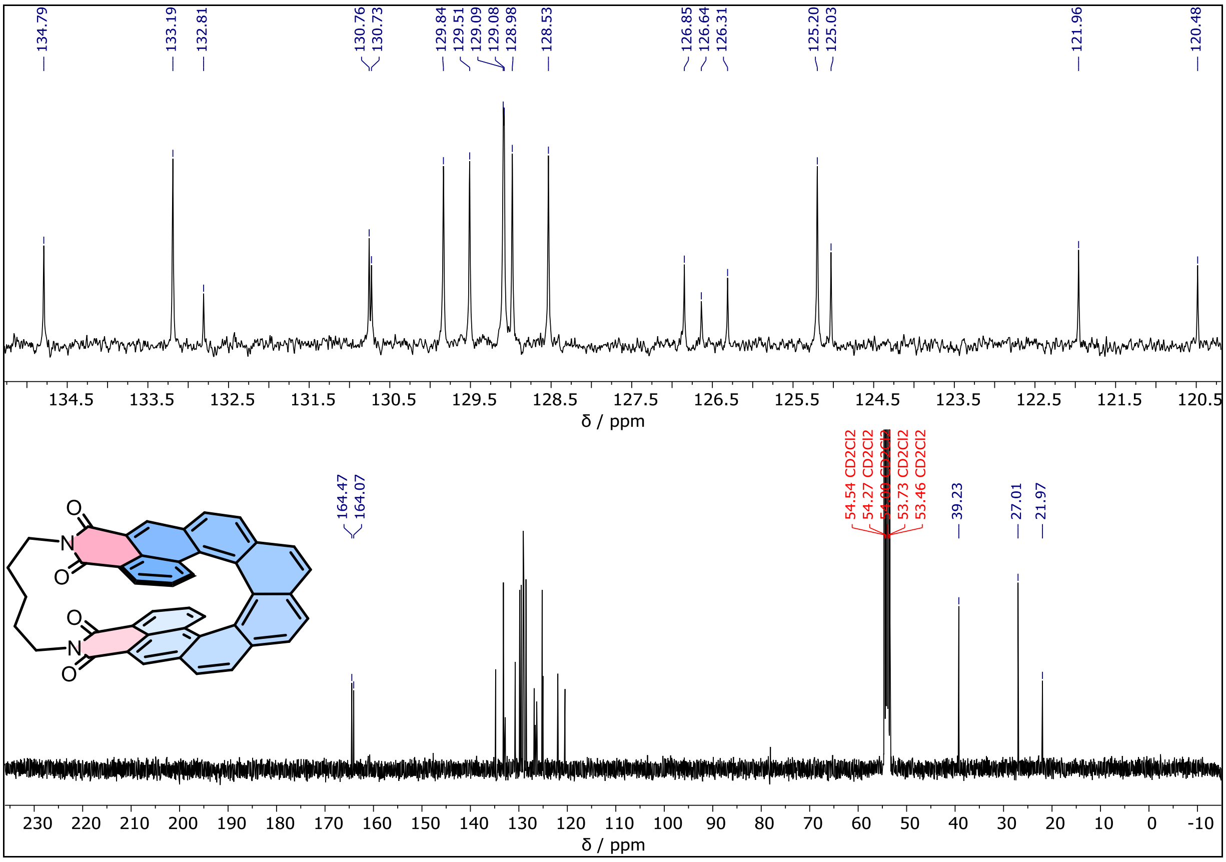

Figure S84. ^{13}C{^{1}H} NMR spectrum (101 MHz, CD_2Cl_2) of **C₅-[8]HDI**.

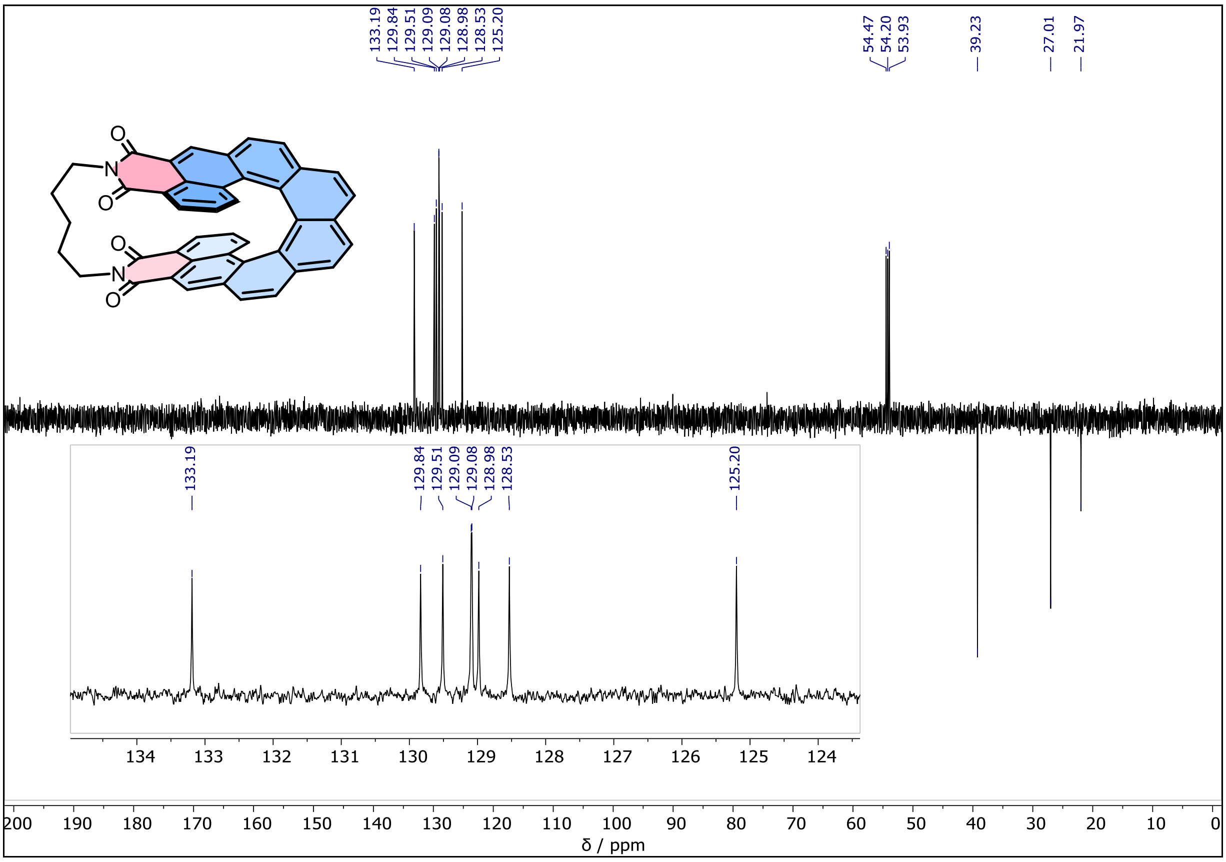

Figure S85. ^{13}C DEPT 135 NMR spectrum (101 MHz, CD_2Cl_2) of **C5-[8]HDI**.

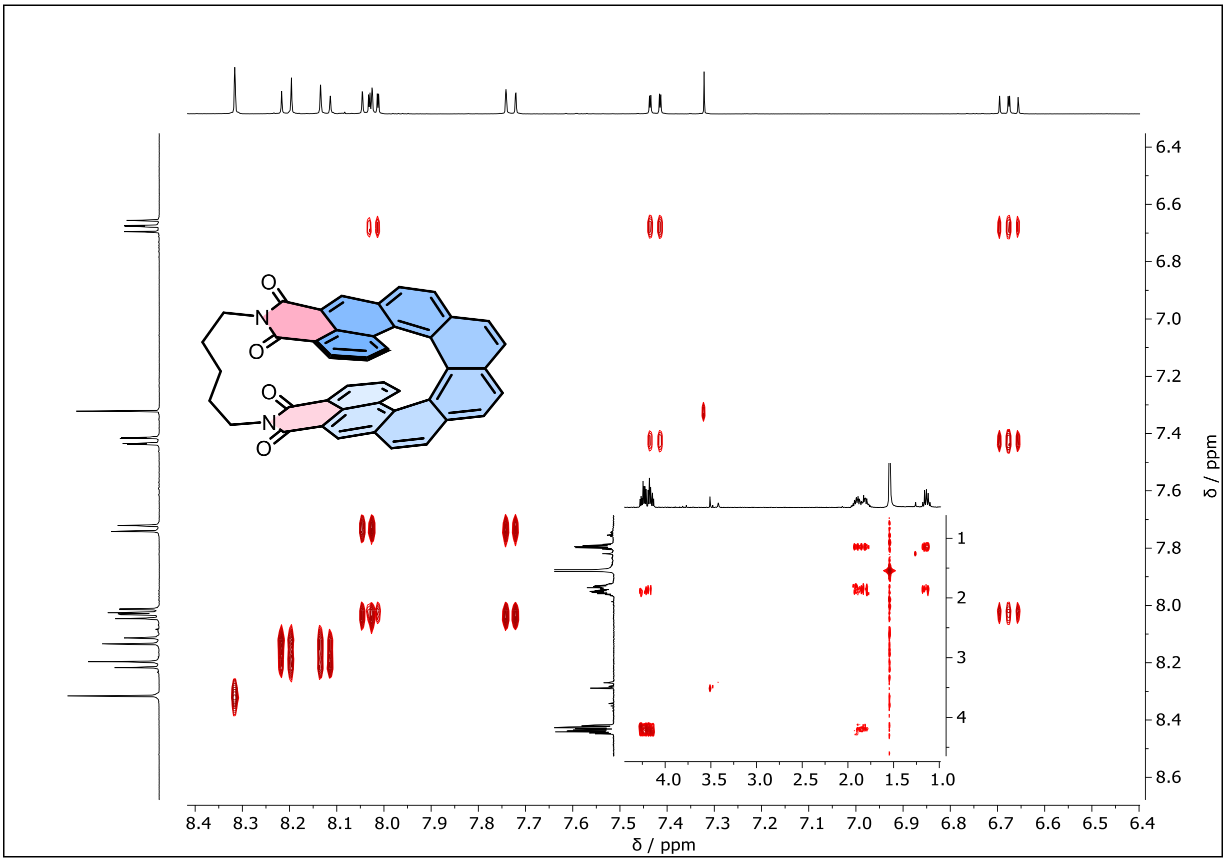

Figure S86. ^{1}H–^{1}H COSY NMR spectrum (400 MHz, CD_2Cl_2) of **C5-[8]HDI**.

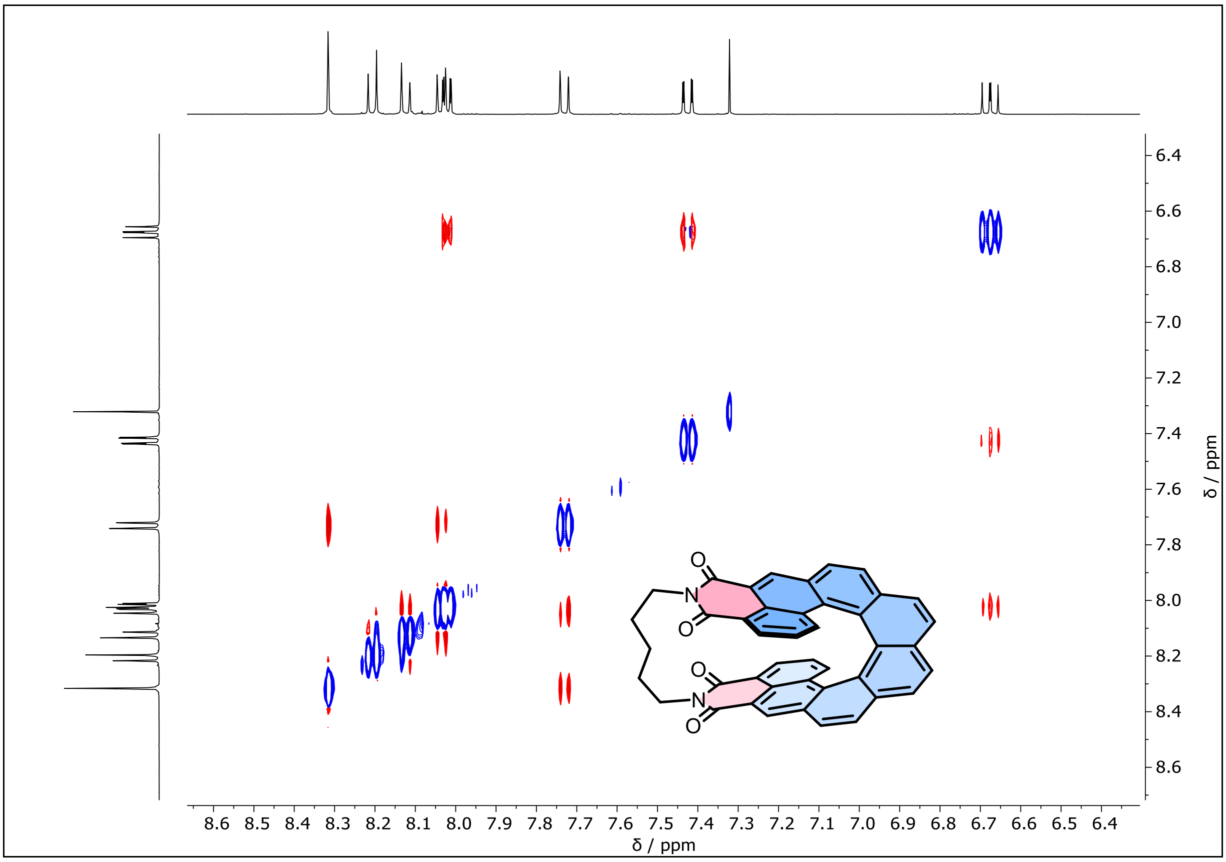

Figure S87. ^{1}H–^{1}H NOESY NMR spectrum (400 MHz, CD_2Cl_2) of **C5-8HDI**.

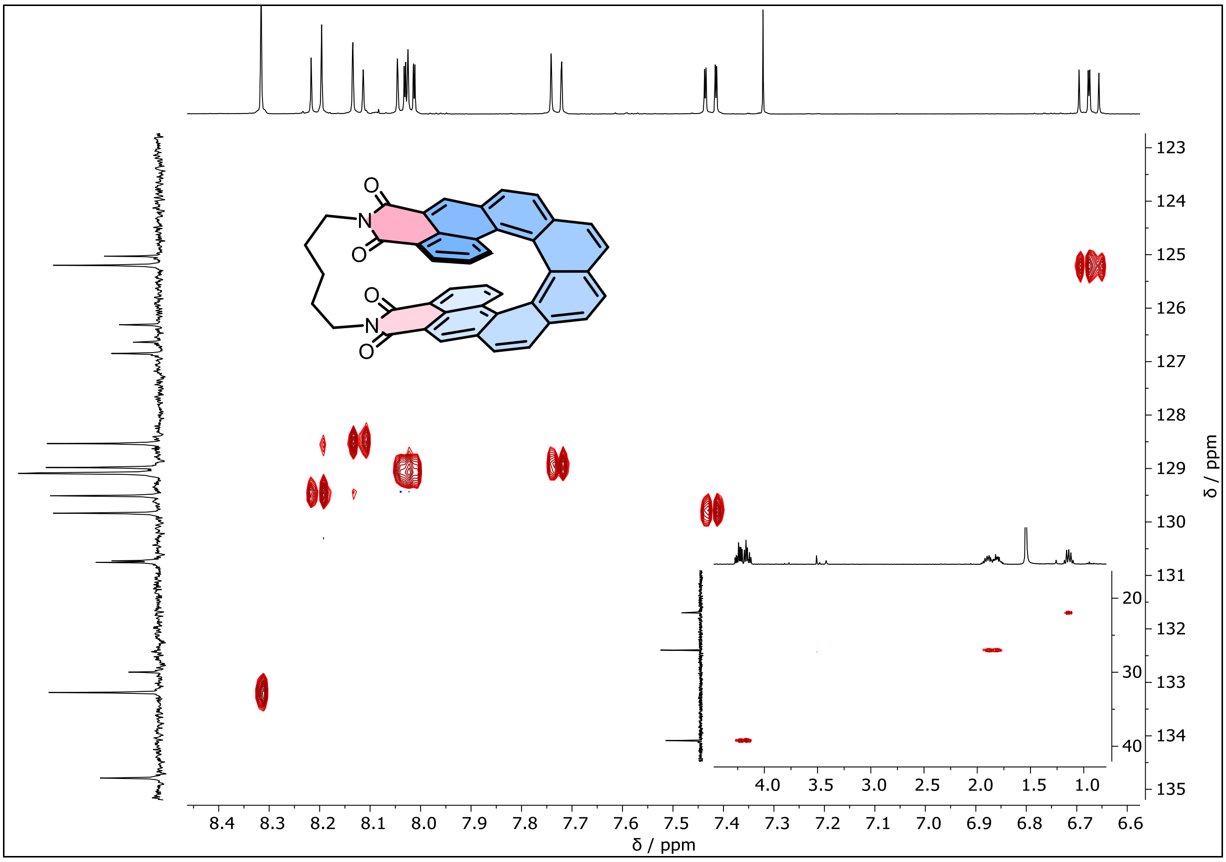

Figure S88. ^{1}H–^{13}C HSQC NMR spectrum (CD_2Cl_2) of **C5-[8]HDI**.

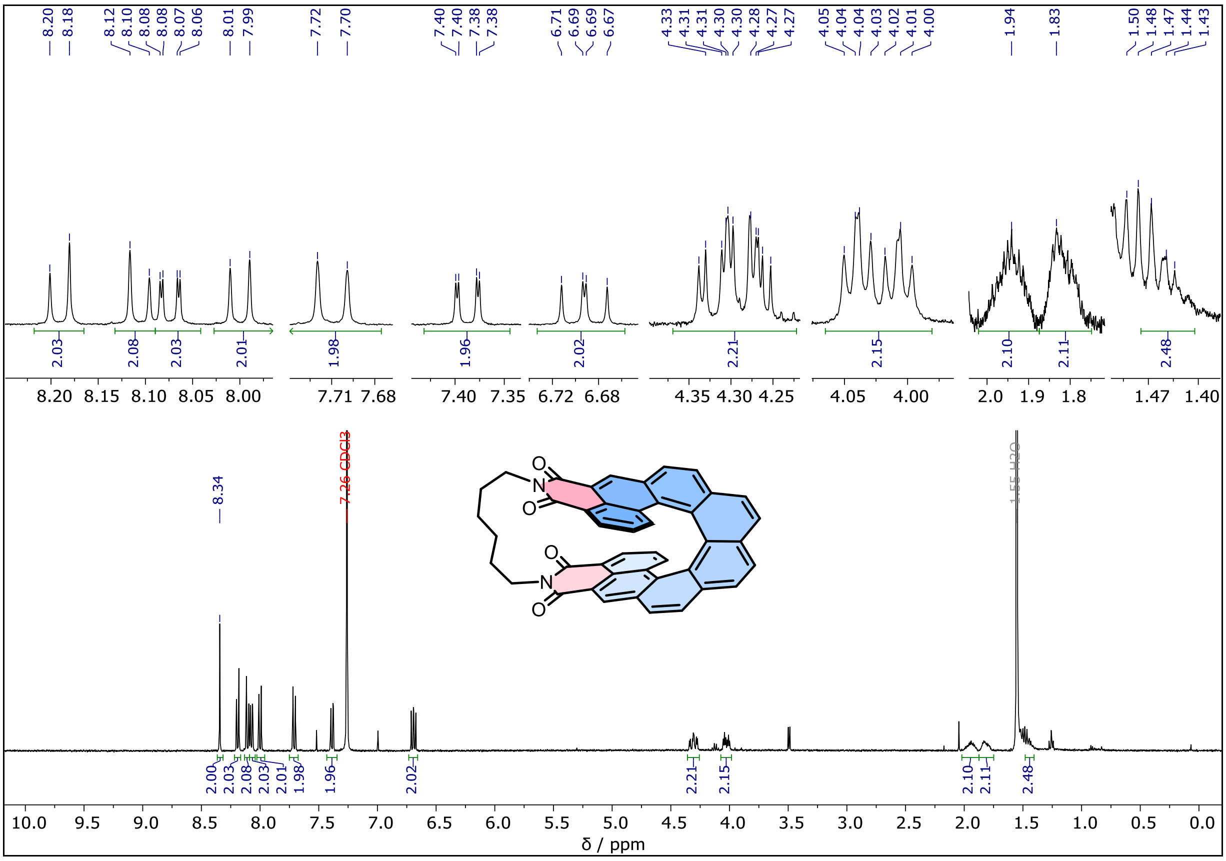

Figure S89. ^{1}H (400 MHz, $CDCl_3$) NMR spectrum of **C_6-[8]HDI**.

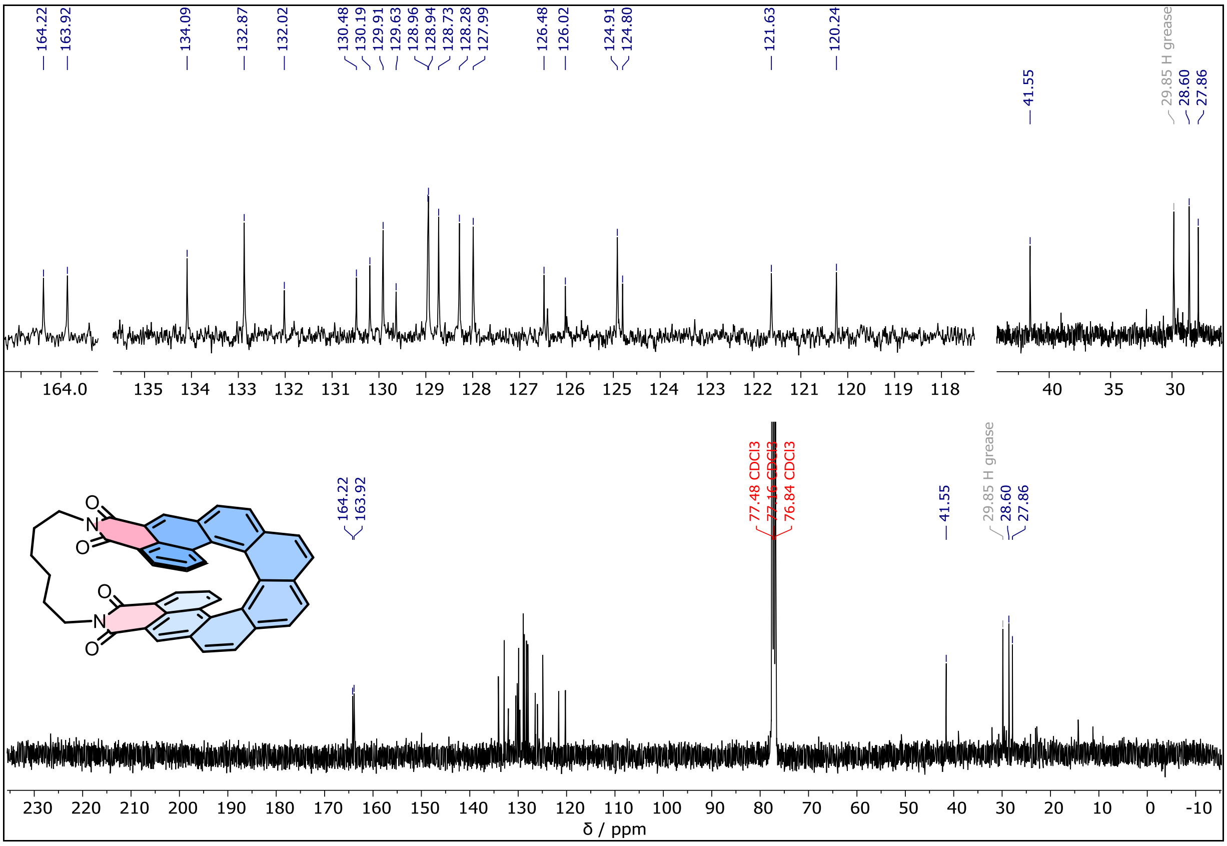

Figure S90. ^{13}C{^{1}H} NMR spectrum (101 MHz, $CDCl_3$) of **C_6-[8]HDI**.

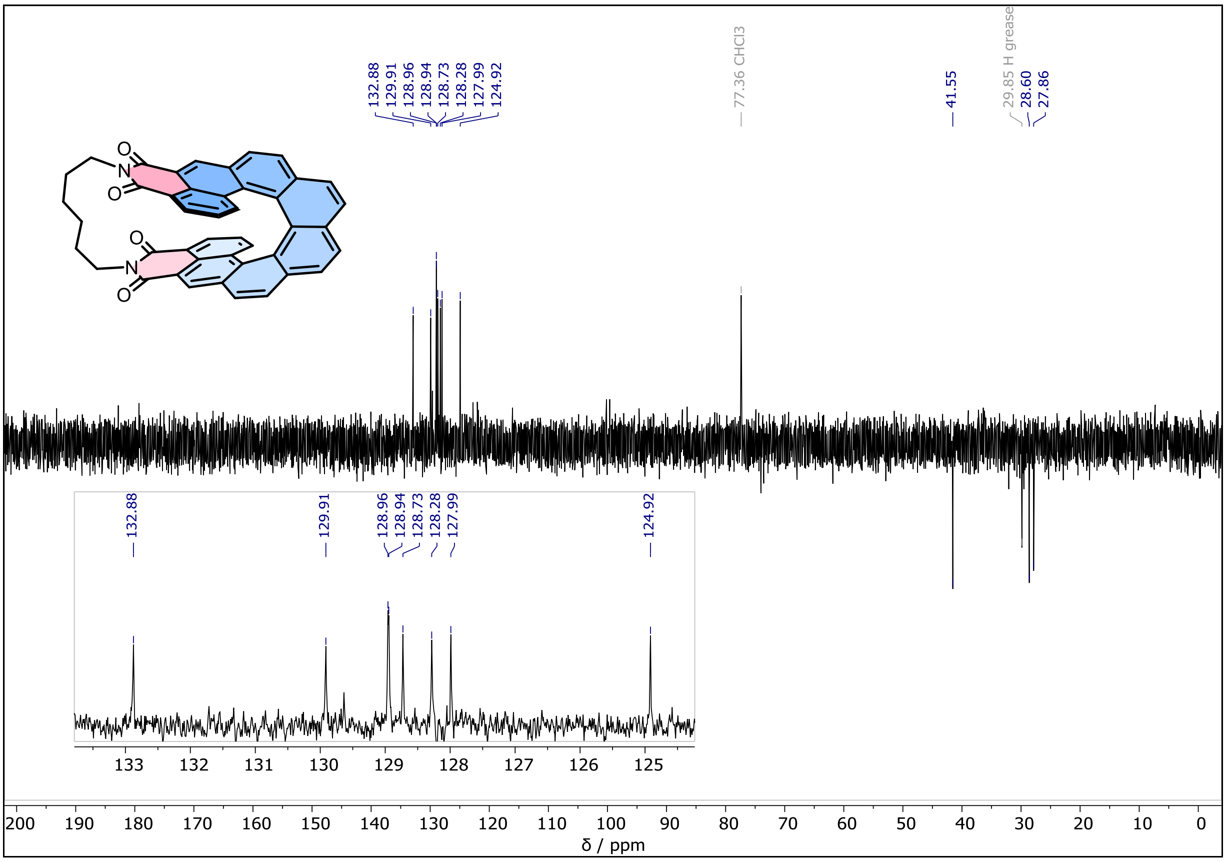

Figure S91. ^{13}C DEPT 135 NMR spectrum (101 MHz, $CDCl_3$) of **C6-[8]HDI**.

S9. High-Resolution Mass Spectrometry (HRMS)

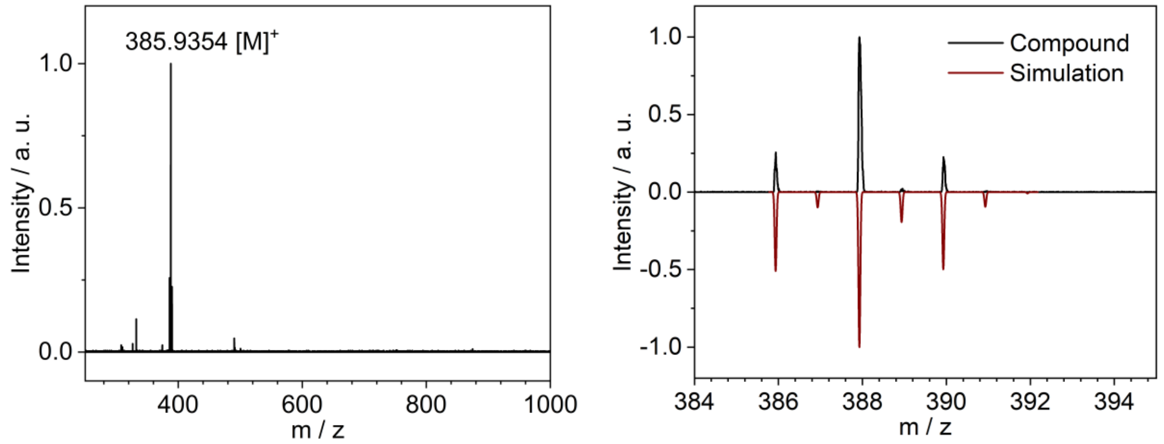

Figure S92. MALDI–TOF HRMS of **(*Z*)-2-bromo-7-(4-bromostyryl)naphthalene**.

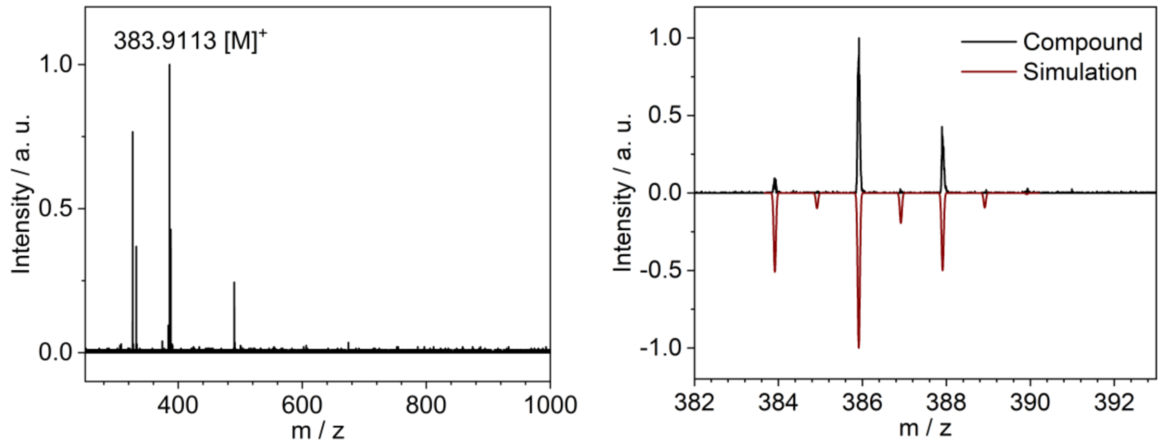

Figure S93. MALDI–TOF HRMS of **2,11-dibromobenzo[c]phenanthrene**.

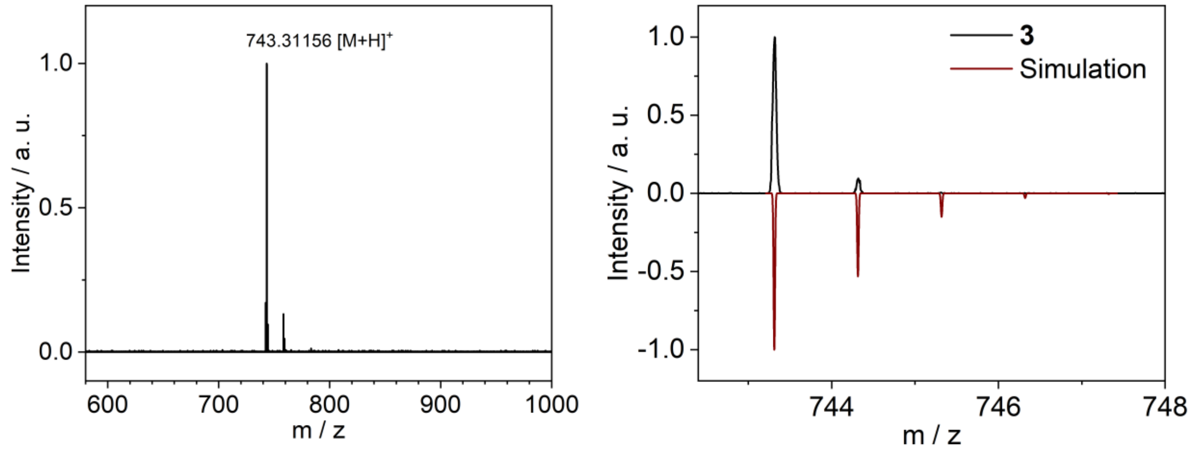

Figure S94. MALDI–TOF HRMS of **3**.

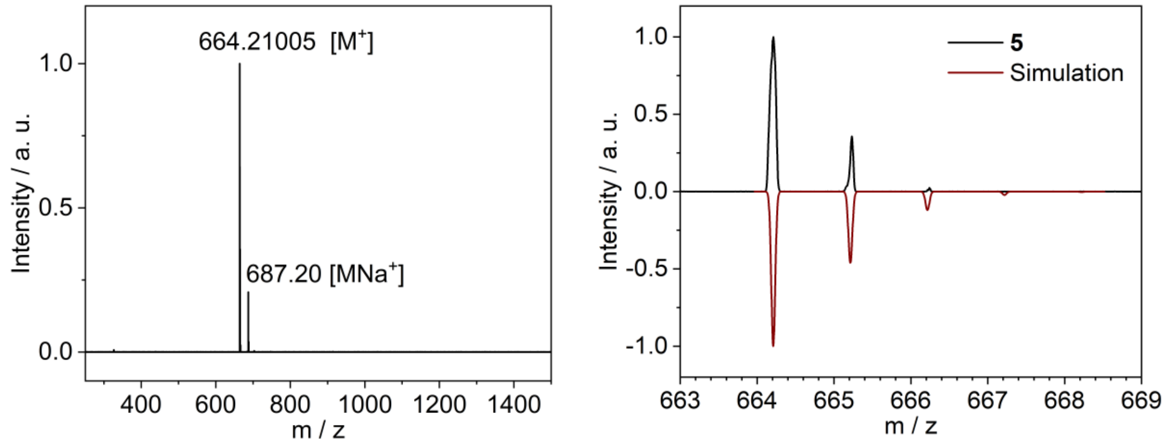

Figure S95. MALDI–TOF HRMS of **5**.

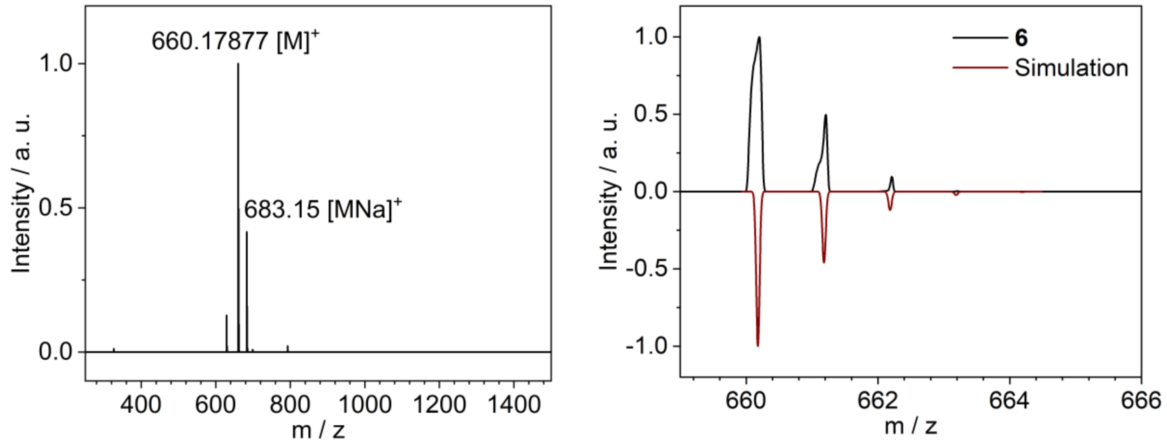

Figure S96. MALDI–TOF HRMS of **6**.

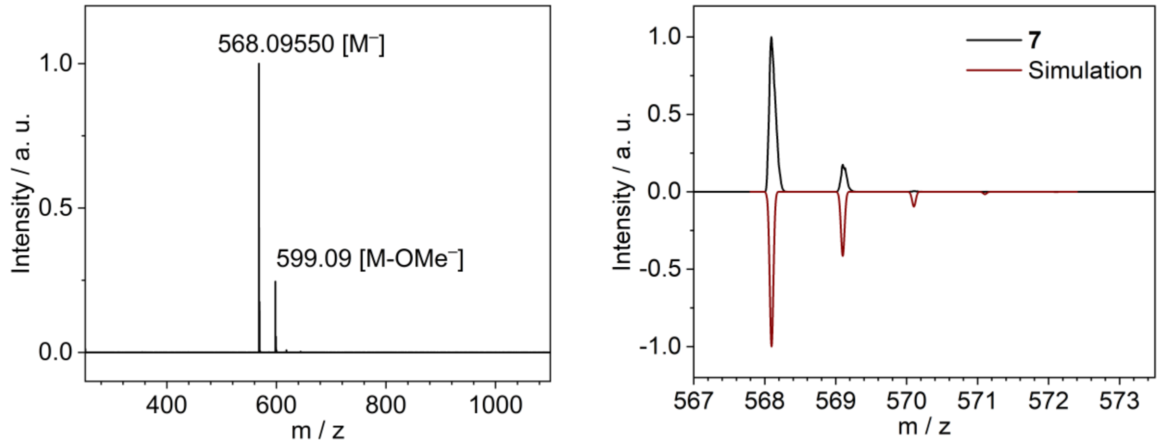

Figure S97. MALDI–TOF HRMS of **7**.

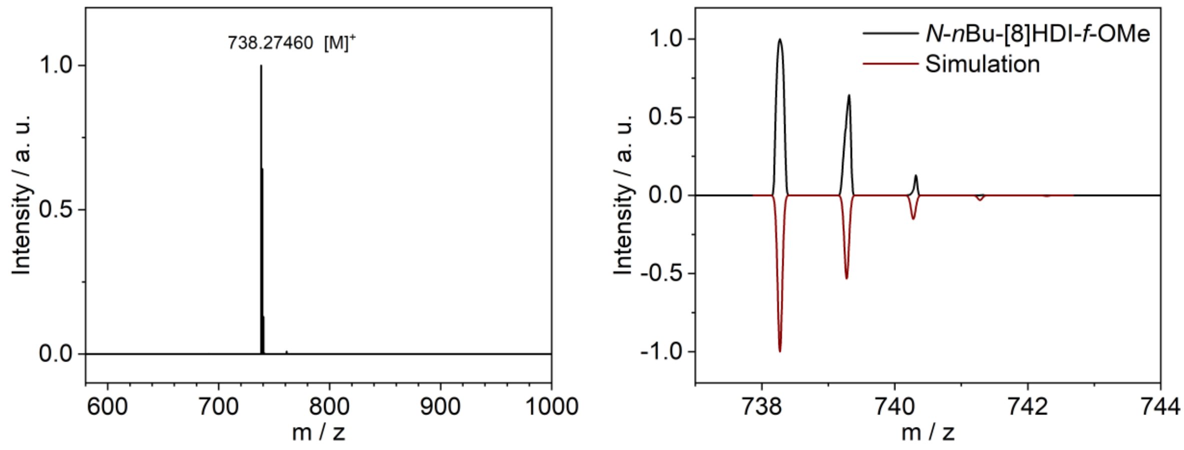

Figure S98. MALDI–TOF HRMS of ***N-n*Bu-[8]HDI-*f*-OMe**.

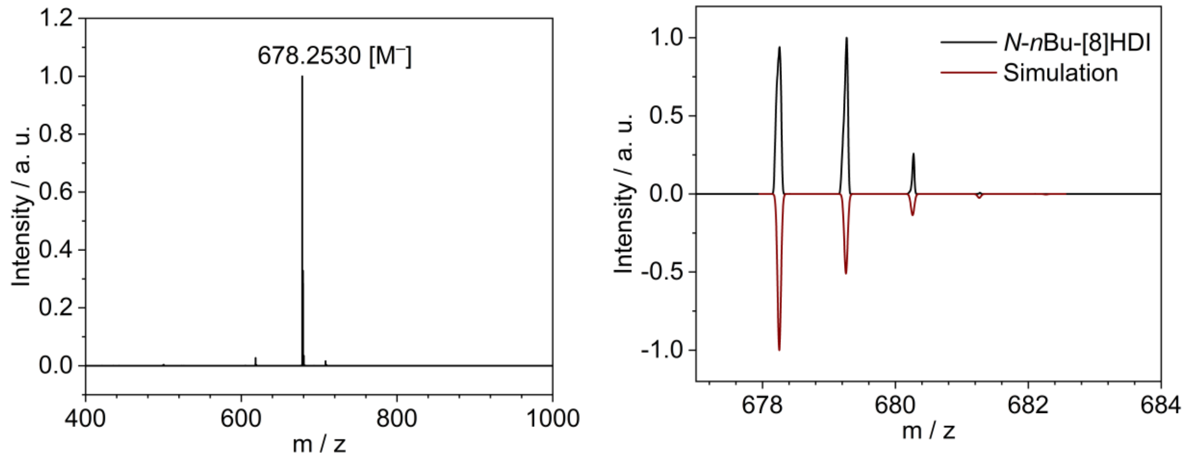

Figure S99. MALDI–TOF HRMS of ***N-n*Bu-8HDI**.

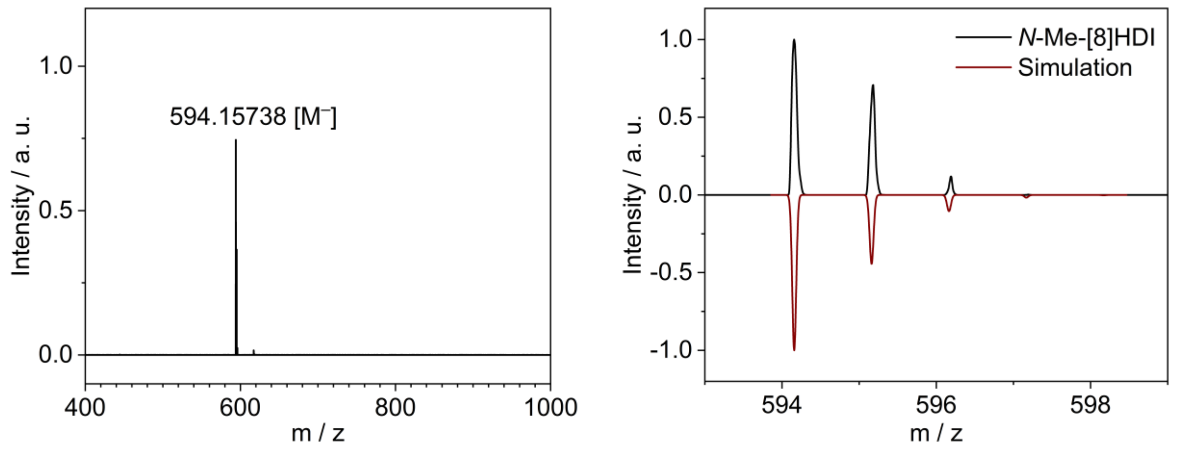

Figure S100. MALDI–TOF HRMS of ***N*-Me-8HDI**.

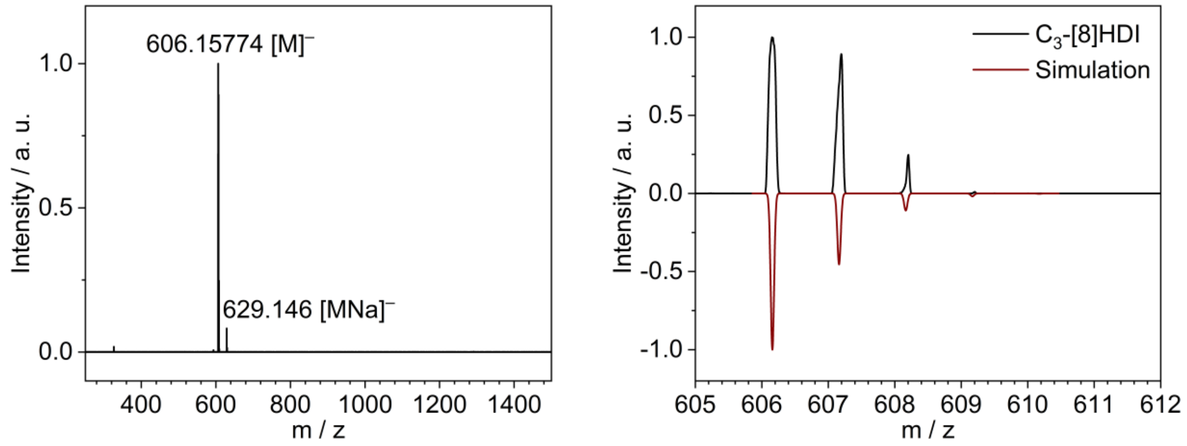

Figure S101. MALDI–TOF HRMS of **C3-8HDI**.

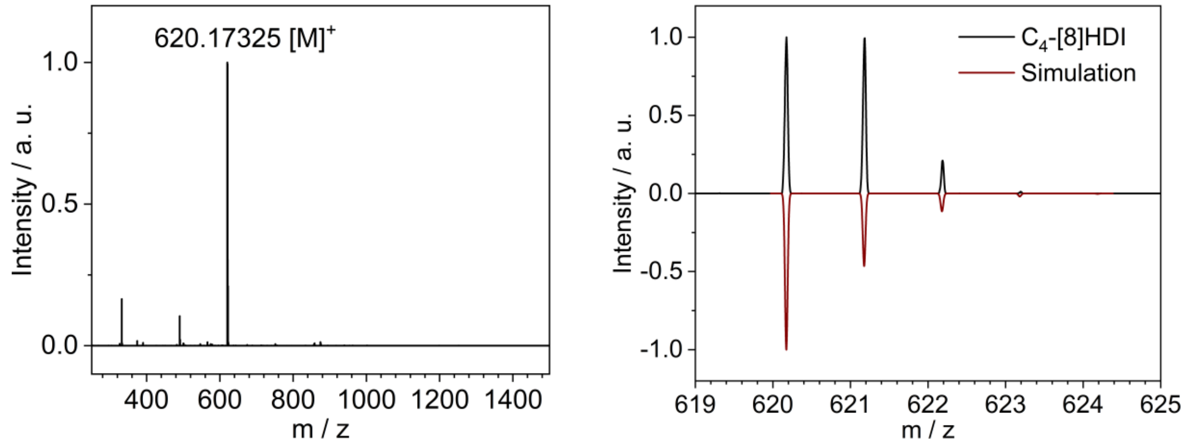

Figure S102. MALDI–TOF HRMS of **C4-8HDI**.

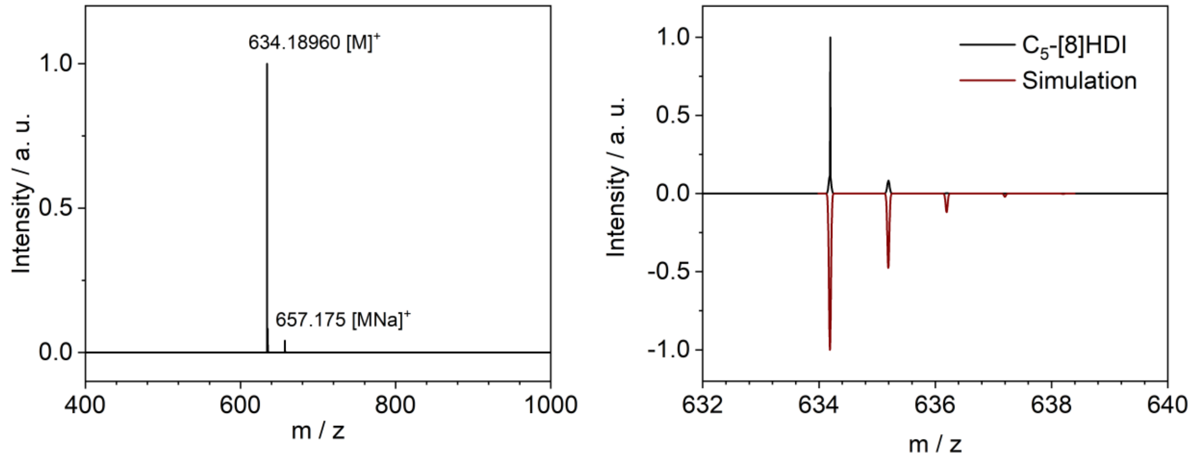

Figure S103. MALDI–TOF HRMS of **C5-8HDI**.

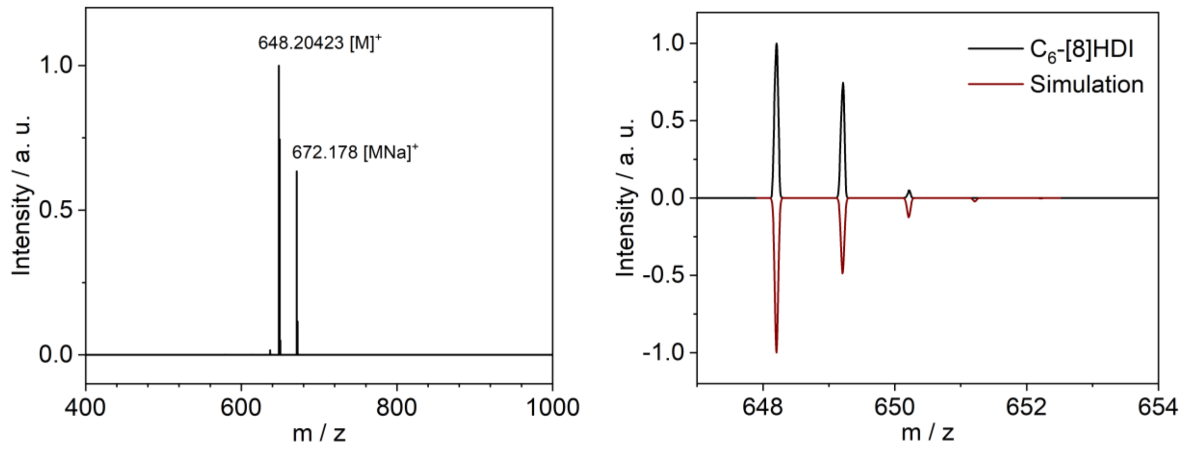

Figure S104. MALDI–TOF HRMS of **C_6-8HDI**.

S10. Cartesian Coordinates

The cartesian coordinates for the discussed compounds in the neutral state and as the radical anions have been uploaded as separate files.

S11. References

[1] F. Saal, F. Zhang, M. Holzapfel, M. Stolte, E. Michail, M. Moos, A. Schmiedel, A.-M. Krause, C. Lambert, F. Würthner, P. Ravat, "[*n*]Helicene Diimides (n = 5, 6, and 7): Through-Bond versus Through-Space Conjugation" *J. Am. Chem. Soc.* **2020**, *142*, 21298–21303.

[2] M. Strohalm, M. Hassman, B. Košata, M. Kodíček, "mMass data miner: an open source alternative for mass spectrometric data analysis" *Rapid Commun. Mass Spectrom.* **2008**, *22*, 905–908.

[3] M. Strohalm, D. Kavan, P. Novák, M. Volný, V. Havlíček, "mMass 3: A Cross-Platform Software Environment for Precise Analysis of Mass Spectrometric Data" *Anal. Chem.* **2010**, *82*, 4648–4651.

[4] T. H. Niedermeyer, M. Strohalm, "mMass as a Software Tool for the Annotation of Cyclic Peptide Tandem Mass Spectra" *PLOS ONE* **2012**, *7*, e44913.

[5] K. Antien, L. Pouysegu, D. Deffieux, S. Massip, P. A. Peixoto, S. Quideau, "Synthesis of [7]Helicene Enantiomers and Exploratory Study of Their Conversion into Helically Chiral Iodoarenes and Iodanes" *Chem. Eur. J.* **2019**, *25*, 2852–2858.

[6] M. Linseis, S. Zalis, M. Zabel, R. F. Winter, "Ruthenium Stilbenyl and Diruthenium Distyrylethene Complexes: Aspects of Electron Delocalization and Electrocatalyzed Isomerization of the Z-Isomer" *J. Am. Chem. Soc.* **2012**, *134*, 16671–16692.

[7] R. S. Thombal, A. R. Jadhav, V. H. Jadhav, "Biomass derived β-cyclodextrin-SO3H as a solid acid catalyst for esterification of carboxylic acids with alcohols" *RSC Adv.* **2015**, *5*, 12981–12986.

[8] M. R. Mahmoud, M. M. El-Shahawi, E. A. A. El-Bordany, F. S. M. Abu El-Azm, "Synthesis of Novel Indeno[1,2-c]isoquinoline Derivatives" *Synth. Commun.* **2010**, *40*, 666–676.

[9] E. H. H. Hasabeldaim, H. C. Swart, R. E. Kroon, "Luminescence and stability of Tb doped CaF(2) nanoparticles" *RSC Adv.* **2023**, *13*, 5353–5366.

[10] R. Martin, M.-J. Marchant, "Resolution and optical properties ([α] max, ORD and CD) of hepta-, octa-and nonahelicene" *Tetrahedron* **1974**, *30*, 343–345.

[11] E. Vander Donckt, J. Nasielski, J. Greenleaf, J. Birks, "Fluorescence of the Helicenes" *Chem. Phys. Lett.* **1968**, *2*, 409–410.

[12] F. Saal, A. K. Swain, A. Schmiedel, M. Holzapfel, C. Lambert, P. Ravat, "Push-pull [7]helicene diimide: excited-state charge transfer and solvatochromic circularly polarised luminescence" *Chem. Commun.* **2023**, *59*, 14005–14008.

[13] C. M. Cardona, W. Li, A. E. Kaifer, D. Stockdale, G. C. Bazan, "Electrochemical Considerations for Determining Absolute Frontier Orbital Energy Levels of Conjugated Polymers for Solar Cell Applications" *Adv. Mater.* **2011**, *23*, 2367−2371.

[14] M. J. Frisch, G. W. Trucks, H. B. Schlegel, G. E. Scuseria, M. A. Robb, J. R. Cheeseman, G. Scalmani, V. Barone, G. A. Petersson, H. Nakatsuji, X. Li, M. Caricato, A. V. Marenich, J. Bloino, B. G. Janesko, R. Gomperts, B. Mennucci, H. P. Hratchian, J. V. Ortiz, A. F. Izmaylov, J. L. Sonnenberg, Williams, F. Ding, F. Lipparini, F. Egidi, J. Goings, B. Peng, A. Petrone, T. Henderson, D. Ranasinghe, V. G. Zakrzewski, J. Gao, N. Rega, G. Zheng, W. Liang, M. Hada, M. Ehara, K. Toyota, R. Fukuda, J. Hasegawa, M. Ishida, T. Nakajima, Y. Honda, O. Kitao, H. Nakai, T. Vreven, K. Throssell, J. A. Montgomery Jr., J. E. Peralta, F. Ogliaro, M. J. Bearpark, J. J. Heyd, E. N. Brothers, K. N. Kudin, V. N. Staroverov, T. A. Keith, R. Kobayashi, J. Normand, K. Raghavachari, A. P. Rendell, J. C. Burant, S. S. Iyengar, J. Tomasi, M. Cossi, J. M. Millam, M. Klene, C. Adamo, R. Cammi, J. W. Ochterski, R. L. Martin, K. Morokuma, O. Farkas, J. B. Foresman, D. J. Fox, *Gaussian 16 Rev. C.01*, Wallingford, CT, **2016**.

[15] P. Pracht, F. Bohle, S. Grimme, "Automated exploration of the low-energy chemical space with fast quantum chemical methods" *Physical Chemistry Chemical Physics* **2020**, *22*, 7169–7192.
[16] S. Grimme, "Exploration of Chemical Compound, Conformer, and Reaction Space with Meta-Dynamics Simulations Based on Tight-Binding Quantum Chemical Calculations" *J. Chem. Theory Comput.* **2019**, *15*, 2847–2862.
[17] C. Lambert, G. Nöll, "The Class II/III Transition in Triarylamine Redox Systems" *J. Am. Chem. Soc.* **1999**, *121*, 8434−8442.
[18] M. Renz, K. Theilacker, C. Lambert, M. Kaupp, "A Reliable Quantum-Chemical Protocol for the Characterization of Organic Mixed-Valence Compounds" *J. Am. Chem. Soc.* **2009**, *131*, 16292–162302.
[19] G. M. Sheldrick, "SHELXT - Integrated space-group and crystal-structure determination" *Acta Crystallogr. A* **2015**, *71*, 3–8.
[20] G. M. Sheldrick, "A short history of SHELX" *Acta Crystallogr. A* **2008**, *64*, 112–122.
[21] L. J. Farrugia, "WinGX and ORTEP for Windows: an update" *J. Appl. Crystallogr.* **2012**, *45*, 849–854.
[22] A. L. Spek, "PLATON SQUEEZE: a tool for the calculation of the disordered solvent contribution to the calculated structure factors" *Acta Crystallogr. C* **2015**, *71*, 9–18.
[23] H. A. Favre, W. H. Powell, *Nomenclature of Organic Chemistry: IUPAC Recommendations and Preferred Names 2013,* Royal Society of Chemistry, **2013**
